# Expanding the Frontier of Linear Drug Design: Cu‐Catalyzed C_sp_–C_sp_^3^‐Coupling of Electron‐Deficient SF_4_‐Alkynes with Alkyl Iodides

**DOI:** 10.1002/advs.202306554

**Published:** 2023-12-31

**Authors:** Srikanth Reddy Narra, Muhamad Zulfaqar Bacho, Masashi Hattori, Norio Shibata

**Affiliations:** ^1^ Department of Nanopharmaceutical Sciences Nagoya Institute of Technology Gokiso, Showa‐ku Nagoya 466‐8555 Japan; ^2^ Department of Life Science and Applied Chemistry Nagoya Institute of Technology Gokiso, Showa‐ku Nagoya 466‐8555 Japan

**Keywords:** alkyl iodide, alkyne, cross‐coupling, fluorine, tetrafluorosulfanyl

## Abstract

Despite the attractive properties of tetrafluorosulfanyl (SF_4_) compounds in drug discovery, medicinal research on SF_4_ molecules is hindered by the scarcity of suitable synthetic methodologies. Drawing inspiration from the well‐established Sonogashira cross‐coupling of terminal alkynes under Pd‐catalysis, it is envisioned that SF_4_‐alkynes can serve as effective coupling partners. To overcome the challenges associated with the electron‐deficient nature of SF_4_‐alkynes and the lability of the SF_4_ unit under transition‐metal catalysis, an aryl radical mediated C_sp_–C_sp_^3^ cross‐coupling reaction is successfully developed under Cu catalysis. This methodology facilitates the coupling of SF_4_‐alkynes with alkyl iodides, leading to the immediate synthesis of SF_4_‐attached drug‐like molecules. These findings highlight the potential impact of SF_4_‐containing molecules in the drug industry, paving the way for further research in this emerging field.

## Graphical abstract

Although SF_4_ compounds have attractive linear connection properties for drug design, there is a lack of suitable synthetic methods for these molecules. In this study, a C_sp_–C_sp_^3^ cross‐coupling reaction is reported between electron‐deficient SF_4_ alkynes and alkyl iodides under Cu catalysis, which have significant implications for the pharmaceutical industry. This finding opens the possibility for further research in this emerging field.

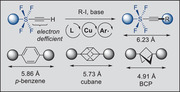

## 1 Introduction

Fluorine (F) atoms, with their high electronegativity and lipophilic nature, have proven to be game‐changers in modifying the physicochemical properties of organic molecules.^[^ ^1^ ^]^ This strategic incorporation of F into suitable positions has been instrumental in designing biologically active compounds, as evidenced by the remarkable success of fluoro‐agrochemicals and fluoro‐pharmaceuticals, which now dominate 70% and 30% of registered drugs, respectively, in the last five years.^[^ ^2^ ^]^ While carbon–fluorine (C–F) units are prevalent in fluorinated drugs on the market, the intriguing prospect of fluorinated drugs featuring an X–F unit, not C–F, remains untapped.^[^ ^2^ ^]^

**Figure 1:**
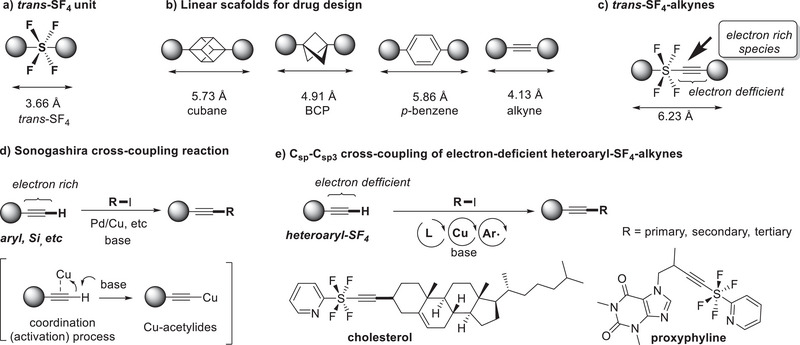
Structure and lengths (calculated, CH_3_) of a) trans‐tetrafluoro‐λ^6^‐sulfanyl(SF_4_) motif and b) linear connective motifs of cubane, BCP, *p*‐benzene, and alkyne. c) Structure and properties of trans‐SF_4_‐alkynes. d) General scheme of Sonogashira cross‐coupling reaction. e) Csp–Csp^3^ cross‐coupling of electron‐deficient heteroaryl‐SF_4_‐alkynes (this work).

Considering this awakening background, we focused on the potential of tetrafluorosulfanyl (SF_4_)‐containing organic molecules,^[^ ^3^, ^4^ ^]^ which harbor sulfur–fluorine (S–F) units, as promising and unconventional drug candidates for the future market.^[^ ^5^ ^]^ The combination of strong electron negativity and high lipophilicity induced by the four F atoms with a hypervalent sulfur atom gives rise to unique properties in the SF_4_ moiety. Notably, the *trans*‐type SF_4_ structure allows two individual substituents to align linearly (**Figure** **1**a), making it an attractive bio‐isostere of non‐conjugated linear motifs such as cubanes and bicyclopentanes (BCP), which mimic *p*‐substituted benzenes and alkynes (Figure 1b).^[^ ^6^, ^7^ ^]^

Despite the immense potential of SF_4_‐containing molecules, progress in their synthesis has been hindered by the scarcity of suitable methodologies.^[^ ^3^, ^4^ ^]^ A recent advance was made by discovering that *trans*‐SF_4_‐alkynes, especially those with heteroaryl groups, offer enhanced stability to SF_4_ molecules, which led to the exploration of the versatility of SF_4_‐alkynes in generating various SF_4_‐containing molecules (Figure 1c).^[^ ^4^ ^]^ These compounds, with their highly electron‐deficient nature in the alkyne portion, exhibit exceptional reactivity toward electron‐rich species. Moreover, SF_4_‐alkynes themselves would be an alternative bioisostere of a linear connection with a length of 6.23 Å, which is 0.37 Å longer than the *p*‐benzene system of 5.86 Å.

Drawing inspiration from the well‐established Sonogashira coupling (Figure 1d),^[^ ^8^ ^]^ a widely used cross‐coupling reaction of terminal alkynes with aryl or vinyl halides under Pd(0)/Cu(I) dual catalytic conditions or related transition‐metal catalysis, we envisioned the potential of employing SF_4_‐alkynes as coupling partners. Sonogashira coupling involves the coordination of Cu(I) to the triple bond of the alkyne, leading to activation of the alkyne moiety and subsequent formation of Cu(I) acetylides. Aryl alkynes and silyl alkynes are the most commonly employed. However, the electron‐deficient nature of the triple bond in SF_4_‐alkynes presents challenges for their coordination with Cu(I) during the catalytic process. Nevertheless, it was speculated that this property might enable the direct formation of Cu‐acetylides without alkyne activation via coordination. Additionally, the use of dual transition metal catalysts was anticipated due to the competitive activation of the S─F bond rather than the alkynes, allowing the breakdown of the critical SF_4_ group.^[^ ^9^ ^]^

Herein, we present an aryl radical‐mediated Cu‐catalyzed cross‐coupling reaction that overcomes the hurdles associated with the electron‐deficient nature of terminal SF_4_ alkynes without decomposition of the SF_4_‐moiety (Figure 1e). This approach successfully coupled SF_4_‐alkynes with primary, secondary, and tertiary alkyl iodides, further expanding the scope of this transformation. The late‐stage functionalization of drug molecules with alkyl iodides using this methodology resulted in instantaneous synthesis of SF_4_‐attached drug‐like molecules. Moreover, we demonstrated derivatization of the resulting inner SF_4_‐alkynes through 1,3‐dipolar cycloaddition with nitrones or click reactions with azides, enabling the linear linkage of two different heterocycles. The ability to incorporate SF_4_ moieties into drug‐like molecules through Sonogashira‐type cross‐coupling reactions allows for the exploration of novel chemical spaces and the potential discovery of promising drug candidates.

## 2 Results and Discussion

Among the numerous reaction conditions reported in the literature for Sonogashira‐ and Sonogashira‐type cross‐coupling reactions, we were intrigued by Liang's aryl radical‐mediated methodology^[^ ^10^ ^]^ for several reasons. First, unlike common Pd‐Cu dual‐transition‐metal systems, this method utilizes only Cu catalysis. Second, the SF_4_ moiety remains stable under radical conditions. Therefore, we investigated the use of 2‐(ethynyltetrafluoro‐λ^6^‐sulfaneyl)pyridine (**1a**, 1.0 equiv.), and iodocyclohexane (**2a**, 1.0 equiv.) as model substrates under Liang's conditions ([Cu(CH_3_CN)_4_]BF_4_ (25 mol%), 2‐mesityl‐1‐diazonium salt (2.0 equiv.), terpyridine (25 mol%), K_2_CO_3_ (3.0 equiv.) in DMSO, 50 °C, 30 min), yielding the desired product **3aa** in 70% yield (**Table** **1**, run 1). Encouraged by this success, we conducted further optimization studies. First, several reactions and catalyst variations were explored. Initial trials using Cu(I) as the catalyst (Table 1, runs 2–10) resulted in yields ranging from 42% to 78%. Subsequently, several Cu(II) catalysts were tested to obtain the desired products in moderate yields (Table 1, runs 11–14). After exploring various options, we found that [Cu(CH_3_CN)_4_]PF_6_ was the most efficient catalyst under these reaction conditions (Table 1, run 5). Considering the activation of the triple bond in **1a**, we recognized the significance of the bases in the formation of the Cu‐acetylide. Hence, we focused on reactions with different bases under [Cu(CH_3_CN)_4_]PF_6_ catalysis. Organic bases (*^i^* Pr_2_NEt and Et_3_N) and inorganic bases (Cs_2_CO_3_ and Na_2_CO_3_) were also investigated (Table 1, runs 15–18). *^i^* Pr_2_NEt exhibited better compatibility, presumably because of its enhanced solubility compared with inorganic bases (Table 1, runs 17 and 18). Next, we focused on various diazonium salts. However, variations in the diazonium salts were detrimental to the transformation, resulting in the formation of byproducts such as aryl coupling products (Table S3, Supporting Information). Our attempts to use different solvents, such as DMF and CH_3_CN, led to lower yields (Table 1, runs 19 and 20; Table S4, Supporting Information ). Reactions in THF and DCM failed to furnish the desired product, and DMSO was identified as the optimal solvent, providing the desired product in 84% yield. During the investigations, we observed that in some cases, starting material **1a** remained unconsumed and trace amounts of aryl coupling products were formed. To address this, we increased the equivalence of **2a**, leading to an improvement in the transformation yield of **3aa** by up to 98% (92% isolated yield; Table 1, run 21). Furthermore, reducing the amount of diazonium salt from 2.0 to 1.0 equiv. resulted in a decrease in yield from 98% to 46% (Table 1, run 22). Control experiments were performed to verify the significance of the various factors involved in the transformation. A reaction attempted in the absence of the diazonium salt yielded compromised results (Table 1, run 23). Additional control experiments without a base (Table 1, 10%, run 24), without a ligand (Table 1, 32%, run 25), and with temperature variation from 55 °C to room temperature (Table 1, 66%, run 26) further emphasized the importance of these factors (see Table S5, Supporting Information). The transformation was also tested by conducting the reaction on a gram‐scale with **1a** and iodocyclohexane **2a**, demonstrating a similar result (Table 1, run 27, 86% isolated yield).

**Table 1:** Optimization of the sonogashira cross‐coupling reaction condition for Py SF_4_ Alkyne^[^ ^a^ ^]^ and unactivated alkyl iodides.

Run	Catalyst	Base	Yield [%]^[^ ^b^ ^]^
1	[Cu(CH_3_CN)_4_]BF_4_	K_2_CO_3_	70
2	Cu(I)I	K_2_CO_3_	68
3	Cu(I)Br	K_2_CO_3_	42
4	Cu(I)Cl	K_2_CO_3_	74
5	[Cu(CH_3_CN)_4_]PF_6_	K_2_CO_3_	78
6	Cu(I)SCN	K_2_CO_3_	60
7	Cu(I)OAc	K_2_CO_3_	51
8	Cu(I)(CF_3_SO_3_)_2_/toluene	K_2_CO_3_	72
9	Cu(I)MeSal	K_2_CO_3_	68
10	CuF(PPh_3_)_3_	K_2_CO_3_	45
11	CuCl_2_	K_2_CO_3_	58
12	Cu(OAc)_2_	K_2_CO_3_	47
13	CuBr_2_	K_2_CO_3_	73
14	Bis(2,4‐pentanedionato)copper(II)	K_2_CO_3_	52
15	[Cu(CH_3_CN)_4_]PF_6_	*^i^* Pr_2_NEt	84
16	[Cu(CH_3_CN)_4_]PF_6_	Et_3_N	61
17	[Cu(CH_3_CN)_4_]PF_6_	Cs_2_CO_3_	52
18	[Cu(CH_3_CN)_4_]PF_6_	Na_2_CO_3_	Trace
19^[^ ^c^ ^]^	[Cu(CH_3_CN)_4_]PF_6_	*^i^* Pr_2_NEt	49
20^[^ ^d^ ^]^	[Cu(CH_3_CN)_4_]PF_6_	*^i^* Pr_2_NEt	57
21	[Cu(CH_3_CN)_4_]PF_6_	*^i^* Pr_2_NEt	98 (92)
22^[^ ^e^ ^]^	[Cu(CH_3_CN)_4_]PF_6_	*^i^* Pr_2_NEt	46
23^[^ ^f^ ^]^	[Cu(CH_3_CN)_4_]PF_6_	*^i^* Pr_2_NEt	0
24	[Cu(CH_3_CN)_4_]PF_6_	*^———‐^*	10
25^[^ ^g^ ^]^	[Cu(CH_3_CN)_4_]PF_6_	*^i^* Pr_2_NEt	32
26^[^ ^h^ ^]^	[Cu(CH_3_CN)_4_]PF_6_	*^i^* Pr_2_NEt	66
27^[^ ^i^ ^]^	[Cu(CH_3_CN)_4_]PF_6_	*^i^* Pr_2_NEt	87

^a)^ All reaction carried out 0.1 mmol of **1a**;

^b) 19^F yield;

^c)^ DMF as solvent;

^d)^ CH_3_CN as solvent instead of DMSO;

^e)^ 1.0 equiv. of diazonium salt instead of 2.0 equiv;

^f)^ Without diazonium salt;

^g)^ Without ligand;

^h)^ Reaction at rt;

^i)^ Gram scale reaction.

**Scheme 1:**
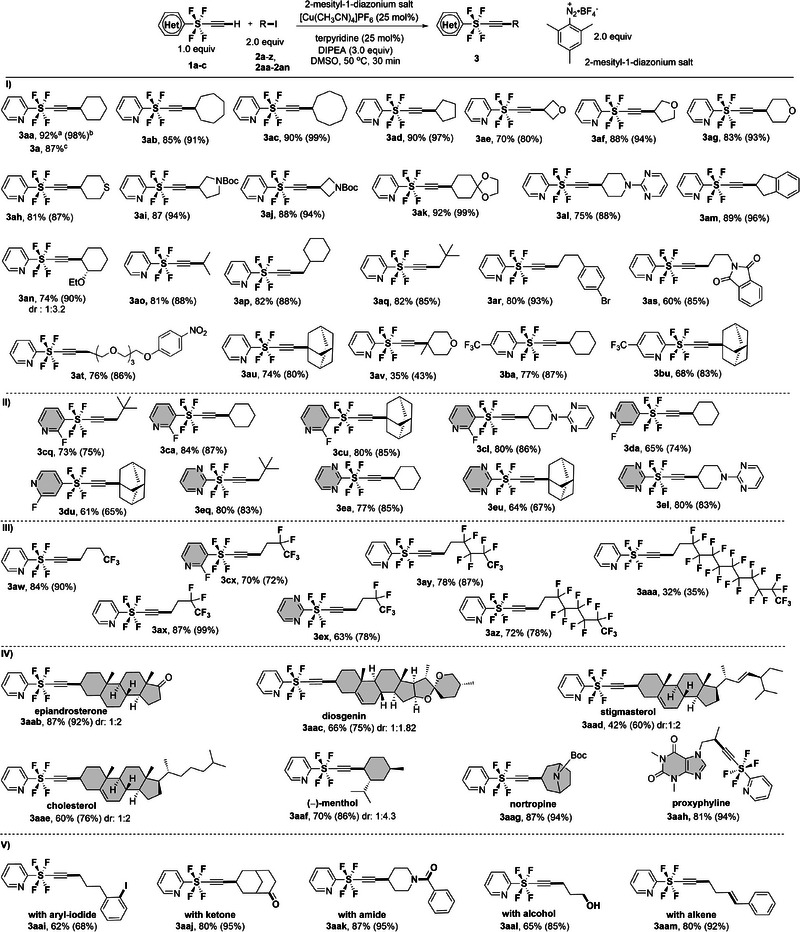
Substrate scope of **1** and **2**. I) Scope of different alkyl iodides. II) Scope of heterocyclic parts of alkynes. III) Scope of perfluorinated alkyl iodides. IV) Late‐stage cross‐coupling reactions of biologically attractive molecules. V) Functional group tolerance. All reactions were carried out with 0.2 mmol of SF_4_ terminal alkyne **1** except for IV and V) 0.1 mmol of **1**. [a] Isolated yields are shown, and [b] ^19^FNMR yields are shown in parentheses. [c] Gram scale reaction.

Initially, we explored the reaction of terminal alkyne **1a** with various secondary alkyl iodides **2a‐o** (**Scheme** **1** (I)) encompassing cyclic scaffolds **2a‐n** and acyclic groups **2o**. The reaction proceeded smoothly with secondary alkyl iodides **2a‐d** bearing 6‐, 7‐, 8‐, and 5‐membered rings, yielding the corresponding coupling products **3aa‐3ad** in high yields (85%–92%). Additionally, cyclic ethers **2e‐g**, cyclic thioether **2** **h**, pyrrolidine **2i**, and azetidine **2j** were accepted as the coupling partners, affording products **3ae‐3aj** in high yields. Furthermore, various functional groups, such as 1,3‐dioxolane **2k**, pyrimidine **2l**, indane **2m**, and ethoxy **2n**, were well tolerated under the reaction conditions, providing the desired products **3ak‐3an** in high yields. Moreover, the methodology was successfully extended to a coupling reaction with acyclic secondary alkyl iodide **2o**, delivering **3ao** in 81% yield. Next, we investigated the cross‐coupling reaction between terminal SF_4_‐alkyne **1a** and primary alkyl iodides. A diverse range of primary alkyl iodides **2p‐t**, containing both cyclic and acyclic alkyl groups, was well tolerated, yielding the desired coupling products **3ap–3at** (Scheme 1 (I)) in good to high yields. Notably, aryl‐Br **2r**, isoindoline **2s**, and polyethylene glycol **2t** moieties were also compatible with these reaction conditions. Tertiary alkyl iodides with adamantane **2u** and 4‐methyltetrahydro‐*2H*‐pyran **2v** were further attempted and reacted smoothly with **3a** to furnish the desired C_sp_–C_sp3_ coupling products **3au** and **3av** (Scheme 1 (I)) in moderate yields of 74% and 35% respectively. Furthermore, 4‐CF_3_ bearing SF_4_‐pyridine **1b** reacted steadily with secondary and tertiary cyclic alkyl iodides to afford the desired products **3ba** and **3bu**, respectively, in good yields.

Subsequently, we explored the variations in the heteroaromatic part of SF_4_‐terminal alkynes **1**. The cross‐coupling reaction of *meta*‐SF_4_‐pyridine **1c** and *para*‐SF_4_‐pyridine **1d** with various alkyl iodides **2**, including primary (**2q**), secondary (**2a**), and tertiary (**2u**) alkyl groups, as well as a heteroatom‐containing molecular architecture (**2l**), resulted in the formation of the corresponding products (**3cq, 3ca, 3cu, 3 cl, 3 da, and 3du**) (Scheme 1 (II)) in good yields of up to 84%. Furthermore, our protocol proved effective with pyrimidine SF_4_‐alkyne **1e** and various alkyl iodides, leading to the synthesis of the desired pyrimidine SF_4_‐alkenyl products (**3eq, 3ea, 3eu,** and **3el**) (Scheme 1 (II)) with good yields of up to 80%.

Building on our success with heteroaromatic substrates, we expanded the scope of this methodology to the synthesis of SF_4_‐alkynes **3** (Scheme 1 (III)) bearing diverse perfluorinated alkyl chains. While short fluoroalkyl groups, such as CF_3_ and C_2_F_5_, are often found in drug structures, longer perfluoroalkyl chains are also useful moieties for the design of agrochemicals and decoy molecules to deceive P450BM3.^[^ ^11^ ^]^ The reactions proceeded smoothly with various perfluoroalkyl iodides, providing the corresponding SF_4_‐alkyne products **3** (Scheme 1 (III)) in good yields up to 87%. Notably, while the majority of products exhibited high yields, the lower yield of the perfluorodecyl‐ethyl product **3aaa** (32%) could be attributed to the highly fluorous character of **2aa**.

Encouraged by these achievements, we applied our concept to the late‐stage cross‐coupling of terminal SF_4_‐alkyne **1a** with alkyl iodides possessing biologically attractive structures (Scheme 1 (IV)) Alkyl iodides derived from various steroid derivatives (epiandrosterone **2ab**, diosgenin **2ac**, stigmasterol **2ad**, and cholesterol **2ae**) were efficiently converted to the corresponding SF_4_‐alkyne coupling products **3aab‐3aae** in good‐to‐excellent yields (**87–42%**). Furthermore, the linear SF_4_ alkyne group was successfully incorporated into (−)‐menthol **2af** (70%), nortropine **2ag** (87%), and proxyphilene **2ah** (81%) via Sonagashira‐type coupling reaction in excellent yields. Next, we explored the functional group tolerance of alkyl iodides. The presence of functional moieties such as ArI (**2ai**), ketones (**2aj**), amides (**2ak**), alcohols (**2al**), and alkenes (**2am**) was well tolerated and delivered the appropriate products **3aai‐3aam** in good yields (up to 87%) (Scheme 1 (V)).

To determine the relevance of this Sonogashira cross‐coupling reaction, we performed robustness screening experiments in the presence of various heterocyclic pharmacophores. Interestingly, even in the presence of a range of nitrogen‐containing heterocycles, such as quinoline, isoquinoline, pyrazine, and pyrazole, the reaction proceeded well, and there was no significant loss in the yields of 3a, which clearly indicated the tolerance of common heterocyclic pharmacophores under the designed reaction conditions (**Table** **2**).

**Table 2:** Tolerance of various heterocyclic pharmacophores under Sonogashira cross‐coupling reaction conditions for 1a and 2a.

Run	Additive	Yield [%]^a^
1	non	98%
2	Quinoline	91%
3	Isoquinoline	80%
4	Pyrazine	96%
5	Pyrazole	92%

^a)19^F NMR yield.

**Scheme 2:**
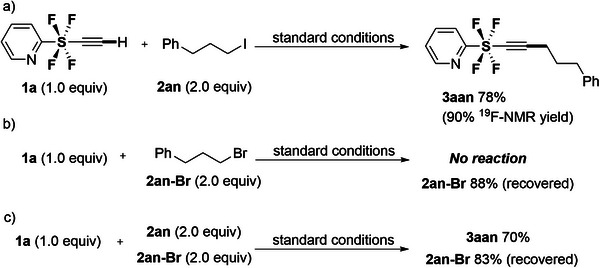
The comparisons of reactivity of alkyl iodide **2an** and bromide **2an‐Br**.

Motivated by this high functional tolerance, we further examined the reaction of **1a** with alkyl iodide **2an** in the presence or absence of alkyl bromide **2an‐Br** (**Scheme** **2**). Interestingly, the reaction proceeded solely with alkyl iodide **2an** (Scheme 2a) and not alkyl bromide **2an‐Br** (Scheme 2b), even in a mixture of **2an** and **2an‐Br** (Scheme 2c). Thus, performing the reaction with **2an** and **2an‐Br** in one pot furnished **3aan** in 70% yield, and **2an‐Br** was recovered in 83% yield. Therefore, these experiments suggest that the C─Br bonds are quite inactive toward the designed methodology and have high selectivity for C─I bonds. Further details of the reaction using alkyl bromides are provided in Supporting Information.

**Scheme 3:**
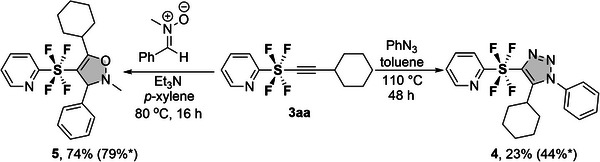
Synthetic application of PySF_4_‐Alkyne cross coupling product **3aa**. Reactions are carried out 0.1 mmol scale **3aa**.

While all SF_4_‐alkyne coupling products **3** are attractive as rod‐like linear compounds with a length of 6.23 Å, they offer even greater potential for further chemical transformations based on established alkyne chemical reactions. To demonstrate this, we performed downstream modifications of **3aa** as shown in **Scheme** **3**. First, alkyne click chemistry of the synthesized compound **3aa** with phenyl azide under metal‐free and heating conditions for two days resulted in the formation of triazole‐SF_4_‐pyridine **4** in a regioselective manner with a yield of 23%. Additionally, 1,3‐dipolar cycloaddition of alkyne **3aa** to nitrone in the presence of Et_3_N led to the formation of isoxazoline‐SF_4‐_pyridine **5** in 74% yield and regioselectivity.

**Figure 2:**
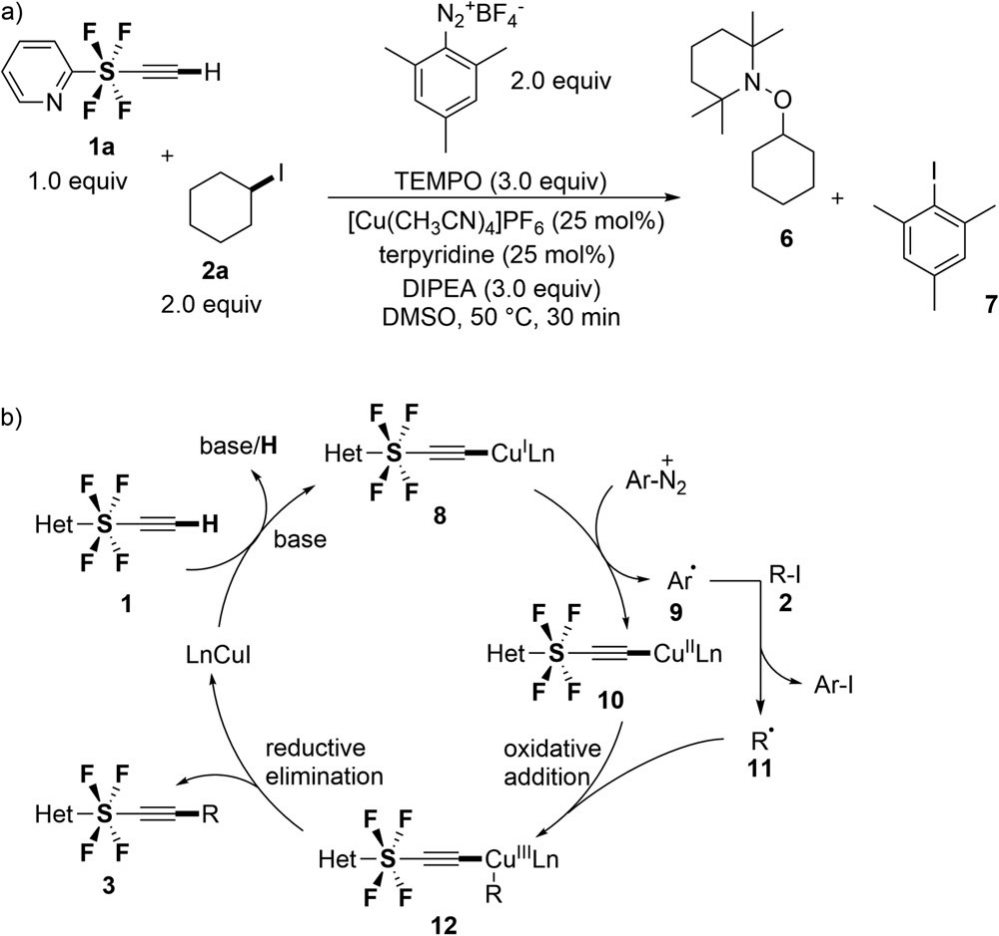
a) TEMPO experiment. b) Plausible pathway for Sonogashira‐type cross‐coupling reaction.

Mechanistic studies were performed to gain insights into the reaction mechanism. Radical trap experiment with 3.0 equiv. of TEMPO confirmed that the reaction proceeds via a radical pathway (**Figure** **2**a). TEMPO completely suppressed the formation of coupled products, and the formation of TEMPO adducts **6** and 2‐iodo‐1,3,5‐trimethylbenzene **7** was detected by GC‐MS (see Supporting Information), further supporting the radical mechanism.

Based on the results obtained from our experiments and relevant literatures,^[^ ^10^ ^]^ we propose a plausible mechanism outlined in Figure 2b. Initially, the terminal hydrogen of SF_4_‐alkyne **1** undergoes deprotonation by *^i^* Pr_2_NEt, followed by a reaction with the Cu(I) ligand, leading to the formation of LnCu(I)‐alkyne complex **8**. Subsequently, the aryl diazonium salt oxidizes the LnCu(I)‐alkyne complex **8** to Cu(II)‐alkyne **10**, generating the aryl radical **9**. The aryl radical **9** then reacts with alkyl iodide **2**, resulting in the formation of the alkyl radical **11** and aryl iodide. The alkyl radical **11** undergoes oxidative addition to Cu(II) complex **10** yielding a Cu(III) alkyne complex **12**. This Cu(III) complex **12** undergoes reductive elimination, leading to the formation of the coupled product **3**, accompanied by the release of the Cu(I) ligand complex. The Cu(I) complex then continues the catalytic cycle, promoting the desired reaction to furnish the product **3**.

## 3 Conclusion

In conclusion, we have successfully reported a Sonogashira‐type cross‐coupling reaction between electron‐deficient SF_4_‐attached alkynes and alkyl iodides, yielding excellent results under mild conditions. The reaction proceeds via a radical pathway facilitated by a diazonium salt and Cu catalysis, demonstrating its versatility by accepting various coupling partners, including primary, secondary, and tertiary alkyl iodides. This protocol enabled the synthesis of more than 50 novel pyridine/pyrimidine‐SF_4_‐alkyne compounds, some of which featured perfluorinated alkyl groups. Furthermore, we demonstrated late‐stage C_sp_–C_sp_^3^‐coupling of biologically relevant molecules, rendering this series of SF_4_‐compounds attractive bio‐isosteres of linear molecules such as p‐substituted benzenes, cubanes, and bicyclopentanes (BCP). We anticipate that this approach will significantly advance the field of linear drug design, opening new possibilities for the development of innovative drug candidates. The findings presented herein highlight the potential impact of SF_4_‐containing molecules in the pharmaceutical industry and pave the way for further research in this emerging field.

## Conflict of Interest

The authors declare no conflict of interest.

## Supporting information

Supporting Information

## Data Availability

The data that support the findings of this study are available in the supplementary material of this article.
