# Supplementary material for: Expanding the Frontier of Linear Drug Design: Cu‐Catalyzed Csp–Csp 3‐Coupling of Electron‐Deficient SF4‐Alkynes with Alkyl Iodides
Source: Adv Sci (Weinh). 2023 Dec 31;11(11):2306554. doi: 10.1002/advs.202306554 (PMC10953538; doi:10.1002/advs.202306554)
Supplement: Supplementary file 1 — Supporting Information [file ADVS-11-2306554-s001.pdf]

ADVANCED SCIENCE
Open Access

Supporting Information

for *Adv. Sci.*, DOI 10.1002/advs.202306554

Expanding the Frontier of Linear Drug Design: Cu-Catalyzed C_{sp}–$C_{sp}{}^3$-Coupling of Electron-Deficient SF_4-Alkynes with Alkyl Iodides

*Srikanth Reddy Narra, Muhamad Zulfaqar Bacho, Masashi Hattori and Norio Shibata**

Supporting Information
©Wiley-VCH 2021
69451 Weinheim, Germany

Expanding the Frontier of Linear Drug Design: Cu-Catalyzed Csp-Csp3-Coupling of Electron-Deficient SF_4-Alkynes with Alkyl Iodides

Srikanth Reddy Narra,[a] Muhamad Zulfaqar Bacho,[a] Masasi Hattori,[b] and Norio Shibata*[a,b]
[a] Department of Nano pharmaceutical Sciences, Nagoya Institute of Technology, Gokiso, Showa-ku, Nagoya 466-8555, Japan
[b] Department of Life Science and Applied Chemistry, Nagoya Institute of Technology, Gokiso, Showa-ku, Nagoya 466-8555, Japan

Abstract: Despite the attractive properties of tetrafluorosulfanyl (SF_4) compounds in drug discovery, medicinal research on SF_4 molecules has been hindered by the scarcity of suitable synthetic methodologies. Drawing inspiration from the well-established Sonogashira cross-coupling of terminal alkynes under Pd-catalysis, it was envisioned that SF_4-alkynes could serve as effective coupling partners. To overcome the challenges associated with the electron-deficient nature of SF_4-alkynes and the reactivity of the SF_4 group under transition-metal catalysis, we successfully developed an aryl radical mediated C_{sp}-C_{sp3} cross-coupling reaction under Cu-catalysis. This methodology facilitated the coupling of SF_4-alkynes with alkyl iodides, leading to the immediate synthesis of SF_4-attached drug-like molecules. These findings highlight the potential impact of SF_4-containing molecules in the drug industry, paving the way for further research in this emerging field.

Table of Contents

I. General information ………3
II. Reaction optimization ………4-6
III. General procedure and Characterizations of SF_4-alkyne (1c) 6-8
IV. General procedure and Characterizations of coupling products ……………………………………………………………………8-19
V. Experiments for determining the reactivity of C-Br bonds 20
VI. Experiment with TEMPO 21-22
VII. References 22
VIII. NMR spectra ………………………………………………………………………………………………..........................23-118

I. General information

All reactions were performed in oven-dried glassware under a positive pressure of nitrogen or argon. Solvents were transferred via syringe and were introduced into the reaction vessels through a rubber septum. All solvents were dried by standard method. All the reactions were monitored by thin-layer chromatography (TLC) carried out on 0.25 mm Merck silica gel (60-F254). The TLC plates were visualized with UV light. All the reaction products were purified by column chromatography and was carried out on a column packed with silica gel 60N spherical neutral size 50-63 mm. The ^{1}H NMR (300 MHz, 500 MHz and 700 MHz) and ^{19}F NMR (282 MHz) spectra as for solution in $CDCl_3$ were recorded on a Varian Mercury 300 and BRUKER 500 Ultra Shield TR. ^{13}C NMR (126.0, 176.0 MHz) spectra for solution in $CDCl_3$ was recorded on a BRUKER 500 Ultra Shield TR. The chemical shifts (δ) are expressed in ppm downfield from internal TMS (δ = 0.00) and coupling constants (*J*) are reported in hertz (Hz). The (trifluoromethyl)benzene ($C_6H_5CF_3$) [δ = −63.72 ($CDCl_3$)] and Hexafluorobenzen (C_6F_6) [δ = −162.2 ($CDCl_3$)] are used as internal standard for ^{19}F NMR. The following abbreviations were used to explain the multiplicities: s = singlet, d = doublet, t = triplet, q = quartet, m = multiplet, br = broad. Mass spectra were recorded on a SHIMADZU GCMS-QP5050A (EI-MS) and SHIMADZU LCMS-2020 (ESI-MS). High resolution mass spectrometry (HRMS) was carried out on an electron impact ionization mass spectrometer with a micro-TOF analyzer and recorded on a Waters, GCT Premier (EI-MS) with a TOF analyzer. Infrared spectra were recorded on a JASCO FT/IR-4100 spectrometer. Melting points were recorded on a BUCHI M-565. Commercially available chemicals were obtained from Aldrich Chemical Co., Alfa Aesar, TCI and used as received unless otherwise noted. Solvents acetonitrile, ethyl acetate, ethanol, Dioxane, DMF, DCM and THF were dried and distilled before use.
The Piridine-SF_4-alkynes **1a, 1c** were prepared according to literature[1] **1b**,**1d** and pyrimidine-SF_4-alkyne **1e** are prepared newly.

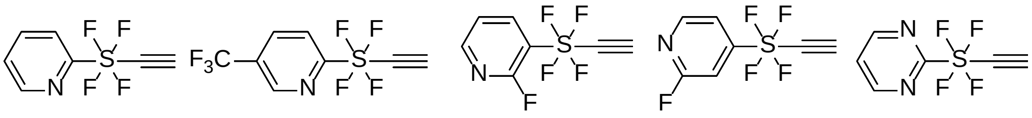

1a **1b** **1c** **1d** **1e**

Figure S1. SF_4-alkyne 1a-e.

Iodo compounds **2b, 2c, 2h, 2k, 2l, 2m, 2n, 2p, 2r, 2s, 2t, 2v** and **2ab-2an** were prepared from alcohol compounds [2] and remaining were obtained from commercially source.

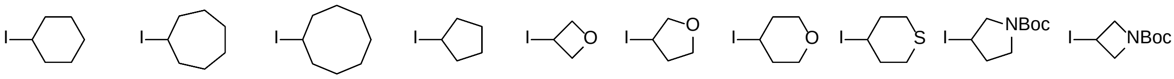

2a **2b** **2c** **2d** **2e** **2f** **2g** **2h** **2i** **2j**

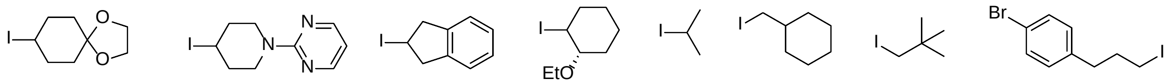

2k **2l** **2m** **2n** **2o** **2p** **2q** **2r**

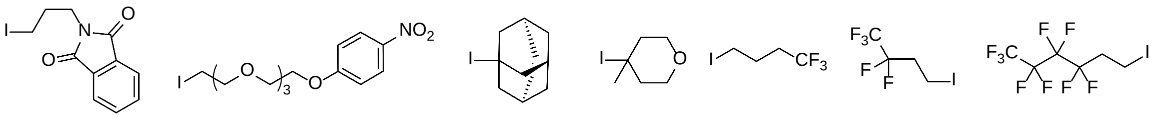

2s **2t** **2u** **2v** **2w** **2x** **2y**

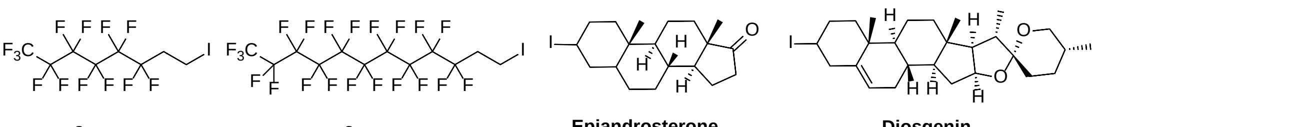

2z **2aa** **Epiandrosterone 2ab** **Diosgenin 2ac**

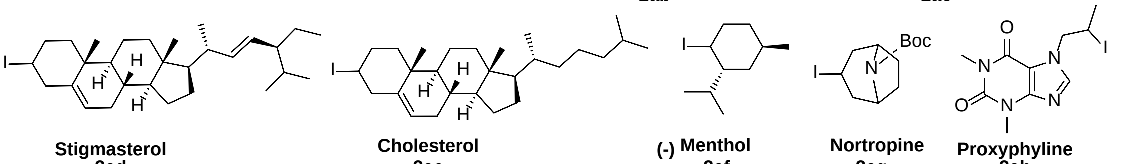

Stigmasterol 2ad **Cholesterol 2ae** **(-) Menthol 2af** **Nortropine 2ag** **Proxyphyline 2ah**

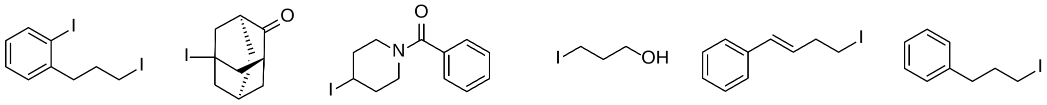

2ai **2aj** **2ak** **2al** **2am** **2an**

Figure S2. Iodo compounds 2a-2z and 2aa-2an.

II. Reaction optimization

Table S1: Optimization of the sonogashira cross-coupling reaction condition for Py SF_4 Alkyne and unactivated alkyl iodides. [a]

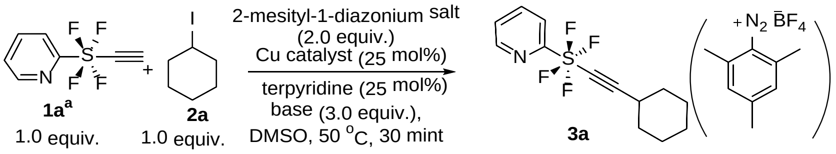

run	Catalyst	base	Yield(%)[b]	Run	Catalyst	base	Yield (%)[b]
1	$[Cu(CH_3CN)_4]BF_4$	K_2CO_3	70	15	$[Cu(CH_3CN)_4]PF_6$	$^iPr_2NEt$	84
2	Cu(I)I	K_2CO_3	68	16	$[Cu(CH_3CN)_4]PF_6$	Et_3N	61
3	Cu(I)Br	K_2CO_3	42	17	$[Cu(CH_3CN)_4]PF_6$	Cs_2CO_3	52
4	Cu(I)Cl	K_2CO_3	74	18	$[Cu(CH_3CN)_4]PF_6$	Na_2CO_3	Trace
5	$[Cu(CH_3CN)_4]PF_6$	K_2CO_3	78	19[c]	$[Cu(CH_3CN)_4]PF_6$	$^iPr_2NEt$	49
6	Cu(I)SCN	K_2CO_3	60	20[d]	$[Cu(CH_3CN)_4]PF_6$	$^iPr_2NEt$	57
7	Cu(I)OAc	K_2CO_3	51	21	$[Cu(CH_3CN)_4]PF_6$	$^iPr_2NEt$	98 (92)
8	$Cu(I)(CF_3SO_3)_2$/toluene	K_2CO_3	72	22[e]	$[Cu(CH_3CN)_4]PF_6$	$^iPr_2NEt$	46
9	Cu(I)MeSal	K_2CO_3	68	23[f]	$[Cu(CH_3CN)_4]PF_6$	$^iPr_2NEt$	0
10	$CuF(PPh_3)_3$	K_2CO_3	45	24	$[Cu(CH_3CN)_4]PF_6$	----------	10
11	$CuCl_2$	K_2CO_3	58	25[g]	$[Cu(CH_3CN)_4]PF_6$	$^iPr_2NEt$	32
12	$Cu(OAc)_2$	K_2CO_3	47	26[h]	$[Cu(CH_3CN)_4]PF_6$	$^iPr_2NEt$	66
13	$CuBr_2$	K_2CO_3	73	27[i]	$[Cu(CH_3CN)_4]PF_6$	$^iPr_2NEt$	87
14	Bis(2,4-pentanedionato)copper(II)	K_2CO_3	52				

[a] all reaction carried out 0.1 mmol of 1a. [b] ^{19}F yield. [c] DMF as solvent. [d] CH_3CN as solvent. [e]1.0 equiv of diazonium salt. [f] absences of diazoniumsalt. [g] without ligand. [h] reaction at rt. [i] gram scale reaction.

Table S2. Ligand screening, related to Table 1

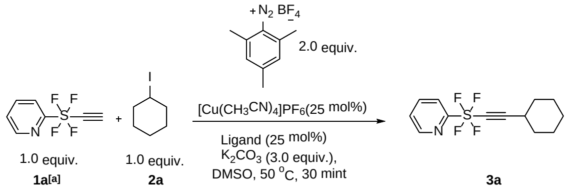

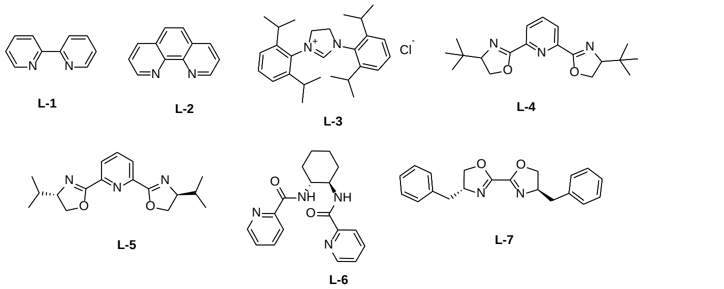

run	Ligand	[b]Yield (%)
**1.0**	L-1	27
**2.0**	L-2	30
**3.0**	L-3	17
**4.0**	L-4	32
**5.0**	L-5	29
**6.0**	L-6	22
**7.0**	L-7	63

[a] all reaction carried out 0.1 mmol of 1a. [b] ^{19}F yield.

Table S2. Base screening

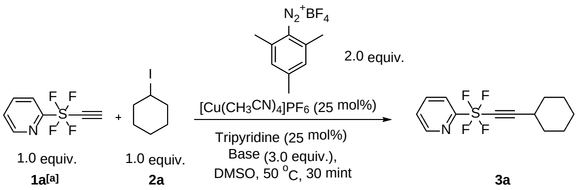

run	Base	[b]Yield (%)
**1.0**	DIPEA	84
**2.0**	Et_3N	61
**3.0**	CS_2CO_3	52
**4.0**	Na_2CO_3	trace

[a] all reaction carried out 0.1 mmol of 1a. [b]^{19}F yield.

Table S3. Diazonium salt screening.

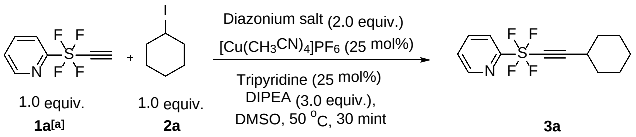

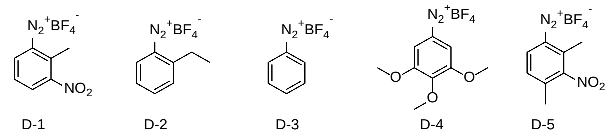

run	Diazonium salt	[b]]Yield (%)
**1.0**	D-1	49
**2.0**	D-2	52
**3.0**	D-3	54
**4.0**	D-4	63
**5.0**	D-5	48

[a] all reaction carried out 0.1 mmol of 1a. [b] ^{19}F yield.

Table S4. Solvent screening

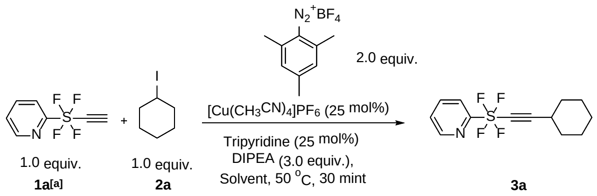

run	Solvent	[b]Yield (%)
**1.0**	ACN	57
**2.0**	DMF	49
**3.0**	THF	0
**4.0**	DCM	0

[a] all reaction carried out 0.1 mmol of 1a. [b] ^{19}F yield

Table S5. Controlled experiments

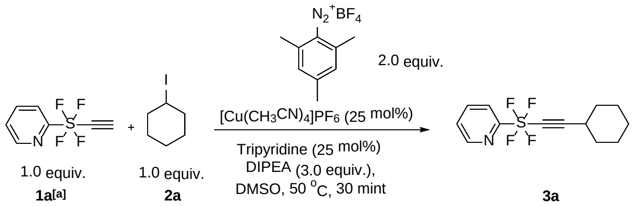

run	Variation	[b]Yield (%)
**1.0**	Without catalyst	0
**2.0**	Without base	10
**3.0**	Without diazonium salt	0
**4.0**	Without ligand	32
**5.0**	1.0 equiv. of diazonium salt	46
**6.0**	Room temperature	66

[a] all reaction carried out 0.1 mmol of 1a. [b] ^{19}F yield.

III. General procedure and Characterizations of SF_4-alkyne (1)

2-(ethynyltetrafluoro-λ^6-sulfaneyl)-5-(trifluoromethyl)pyridine (1b)

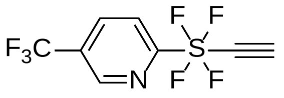

1b

In an argon-filled glovebox, a flame-dried round-bottom flask was charged with 2-(chlorotetrafluoro-λ^6-sulfaneyl)-5-(trifluoromethyl)pyridine (2.89 g, 10.0 mmol, 1.0 equiv) and trimethylsilylacetylene (1.17 g (1.16 mL), 12.0 mmol, 1.2 equiv). The reaction mixture was irradiated by 1W blue LED light and stirred at room temperature for 7 days. Thereafter, the reaction mixture was quenched by Na_2CO_3 aqueous solution followed by extraction with Et_2O. Combined organic layers were finally washed with brine solution, dried over anhydrous Na_2SO_4, then the solvent was removed under reduced pressure. The crude product was purified by flash column chromatography (using hexane/ethyl acetate) to obtain impure product **1b^1** (1.53 g) as a off white solid. Under an N_2 atmosphere, in a flame-dried round-bottom flask, a THF solution of b^1 (1.53 g, 3.95 mmol 1.0 equiv) was cooled to −78 °C. To this solution, MeLi (7.49 mL, 8.69 mmol, 2.2 equiv, 1.16 M in Et_2O) was added dropwise and the mixture was stirred at −78 °C for 12 h. After that, the reaction mixture was quenched by NH_4Cl aqueous solution followed by extraction with Et_2O. Combined organic layers were finally washed with brine solution, dried over anhydrous Na_2SO_4, and then the solvent was removed under reduced pressure. The crude product was purified by flash column chromatography (using hexane/ethyl acetate) to obtain pure product **1c** (190 mg 17.2%) .

(*E*)-2-((2-chloro-2-(trimethylsilyl)vinyl)tetrafluoro-λ^6-sulfaneyl)-5-(trifluoromethyl)pyridine (1b^1)

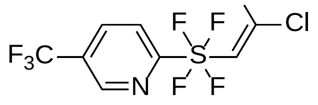

Color: off white solid. mp: 88.6 -89.3 °C (10% EtOAc/hexane). ^{1}H NMR (300 MHz, $CDCl_3$) δ 8.12 (d, *J* = 9.7 Hz, 1H), 7.88 (d, *J* = 8.6 Hz, 1H), 7.60 – 7.42 (m, 1H) 0.36 (s, 9H). ^{19}F NMR (282 MHz, CDCl3) δ 58.24 (d, *J* = 9.2 Hz, 4F), 63.02 (s, 3F). ^{13}C NMR (126 MHz, $CDCl_3$) δ 171.88 (p, *J* = 32.3 Hz), 153.06 (p, *J* = 27.2 Hz), 147.44 (t, *J* = 8.8 Hz),144.83, 135.89 (d, *J* = 3.6 Hz), 128.75 (q, *J* = 33.7 Hz), 122.59 (t, *J* = 272.9 Hz), 121.66 (p, *J* = 4.7 Hz), 0.43. ATR-FTIR (KBr): v = 3116, 3069, 2977, 2917, 1599, 1582, 1469, 1582, 1469, 1385, 1258, 1080, 8140, 705 cm-1.

2-(ethynyltetrafluoro-l6-sulfaneyl)-5-(trifluoromethyl)pyrimidine (1b)

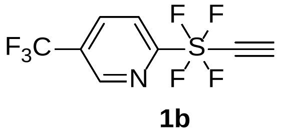

1b

Yield: (190 mg, 17.2%), Color: white solid. mp: 78.6 – 79.1 °C (100% hexane to 10% EtOAc/hexane) ^{1}H NMR (400 MHz, $CDCl_3$) δ 8.83 (s, 1H), 8.14 (d, *J* = 8.7 Hz, 1H), 7.89 (d, *J* = 8.5 Hz, 1H), 2.93 – 2.91 (m, 1H). ^{19}F NMR (282 MHz, $CDCl_3$) δ 74.83 (s, 4F), -63.05 (s,

3F).^{13}C NMR (126 MHz, $CDCl_3$) δ 177.42 (t, J = 29.9 Hz), 145.04, 136.11, 129.20 (q, J = 33.7 Hz), 122.47 (q, 272.9 Hz), 121.53 (t, J = 4.5 Hz), 91.96 – 83.68 (m), 68.30 – 57.44 (m). LCMS (ESI+) m/z: [M + H]+ Calcd for $C_8H_6F_7NS$ 280.00 found 280.0. ATR-FTIR (KBr): v = 3297, 3066, 2106, 1599, 1583, 1389, 1124, 1080, 1014, 854 cm^{-1}.

4-(ethynyltetrafluoro-λ^6-sulfaneyl)-2-fluoropyridine (1d)

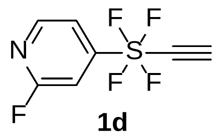

1d

In an argon-filled glovebox, a flame-dried round-bottom flask was charged with pyridine-tetrafluoro-λ^6-sulfanylchloride (1.3 g, 5.42 mmol, 1.0 equiv) and trimethylsilylacetylene (639 mg, 6.5 mmol, 1.2 equiv). The reaction mixture was irradiated by 40W blue LED light 450 nm and stirred at room temperature for 16 h. Thereafter, the reaction mixture was quenched by Na_2CO_3 aqueous solution followed by extraction with Et_2O. Combined organic layers were finally washed with brine solution, dried over anhydrous Na_2SO_4, then the solvent was removed under reduced pressure. The crude product was purified by flash column chromatography (using hexane/ethyl acetate) to obtain pure product **1d^1** (1.3 g 71%) as a colou less oil. Under an N_2 atmosphere, in a flame-dried round-bottom flask, a THF solution of d^1 (1.0 g, 2.96 mmol 1.0 equiv) was cooled to −78 °C. To this solution, MeLi (5.76 mL, 6.51 mmol, 2.2 equiv, 1.16 M in Et_2O) was added dropwise and the mixture was stirred at −78 °C for 12 h. After that, the reaction mixture was quenched by NH_4Cl aqueous solution followed by extraction with Et_2O. Combined organic layers were finally washed with brine solution, dried over anhydrous Na_2SO_4, and then the solvent was removed under reduced pressure. The crude product was purified by flash column chromatography (using hexane/5%ethyl acetate) to obtain pure product **1d (**87.0 mg 13%**)**.

(*E*)-4-((2-chloro-2-(trimethylsilyl)vinyl)tetrafluoro-λ^6-sulfaneyl)-2-fluoropyridine (1d^1)

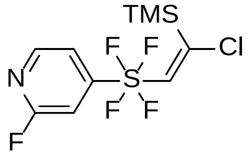

Yield: (1.3 g, 71%), Color: colorless oil (5% EtOAc/hexane). ^{1}H NMR (300 MHz, $CDCl_3$) δ 8.33 (d, *J* = 5.6 Hz, 1H), 7.51 (dd, *J* = 5.7, 1.7 Hz, 1H), 7.44 (p, 1H), 0.35 (s, 9H). ^{19}F NMR (282 MHz, CDCl3) δ 67.34 (d, *J* = 10.6 Hz, 4F) -65.01 (1F). ^{13}C NMR (126 MHz, $CDCl_3$) δ 170.30 (dd, *J* = 28.8, 7.9 Hz), 164.03 (d, *J* = 241.1 Hz), 153.81 (p, *J* = 27.4 Hz), 148.67 (d, *J* = 15.0 Hz), 148.12 (t, *J* = 8.9 Hz), 118.75 (q, *J* = 5.1 Hz), 108.13 (dt, *J* = 42.0, 5.5 Hz), 0.01. LCMS (ESI+) m/z: [M + Na]+ Calcd for $C_{10}H_{14}ClF_5NSSi$ 338.02; found 338.05. ATR-FTIR (KBr): v = 3113, 3071, 2964, 2905, 1587, 1473, 1398, 1296, 1256, 1231, 1095, 996, 695 cm-1.

4-(ethynyltetrafluoro-λ6-sulfaneyl)-2-fluoropyridine (1d)

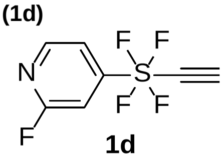

1d

Yield: (87 mg, 13%), Color: white solid. mp: 116.2 – 117.6 °C (5% EtOAc/hexane) ^{1}H NMR (500 MHz, $CDCl_3$) δ 8.36 (d, *J* = 5.5 Hz, 1H), 7.52 (d, J = 5.5 Hz, 1H), 7.27 (t, *J* = 2.0 Hz, 1H), 2.91 (p, J = 3.3 Hz 1H). ^{19}F NMR (282 MHz, $CDCl_3$) δ 83.91 (s, 4F), -64.46 (s, 1F). ^{13} C NMR (126 MHz, $CDCl_3$) δ 167.65 (td, *J* = 26.2, 7.9 Hz), 163.54 (d, *J* = 241.8 Hz), 148.50 (d, *J* = 15.0 Hz), 118.04 (q, *J* = 5.0 Hz), 107.56 (dt, *J* = 42.2, 5.4 Hz), 86.52 (p, *J* = 53.1 Hz), 63.20 – 62.70 (m). LCMS (ESI+) m/z: [M + H]+ Calcd for $C_7H_6F_5NS$ 230.0; found 230.15. ATR-FTIR (KBr): v = 3181, 2092, 1594, 1476, 1402, 1233, 1096, 911, 665 cm^{-1}.

pyrimidine-SF_4-alkyne (1e)

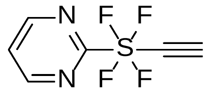

1e

In an argon-filled glovebox, a flame-dried round-bottom flask was charged with pyridine-tetrafluoro-λ^6-sulfanylchloride (6.8 g, 26.95 mmol, 1.0 equiv) and trimethylsilylacetylene (3.17 g (4.47 mL), 32.35 mmol, 1.5 equiv). The reaction mixture was irradiated by 1W blue LED light and stirred at room temperature for 7 days. Thereafter, the reaction mixture was quenched by Na_2CO_3 aqueous solution followed by extraction with Et_2O. Combined organic layers were finally washed with brine solution, dried over anhydrous Na_2SO_4, then the solvent was removed under reduced pressure. The crude product was purified by flash column chromatography (using hexane/ethyl acetate) to obtain pure product **1e^1** (3.33 g 39%) as a white solid. Under an N_2 atmosphere, in a flame-dried round-bottom flask, a THF solution of c^1 (2.61 g, 8.14 mmol 1.0 equiv) was cooled to −78 °C. To this solution, MeLi (15.44 mL, 17.91 mmol, 2.2 equiv, 1.16 M in Et_2O) was added dropwise and the mixture was stirred at −78 °C for 12 h. After that, the reaction mixture was quenched by NH_4Cl aqueous solution followed by extraction with Et_2O. Combined organic layers were finally washed with brine solution, dried over anhydrous Na_2SO_4, and then the solvent was removed under reduced pressure. The crude product was purified by flash column chromatography (using hexane/ethyl acetate) to obtain pure product **1e**.

(*E*)-2-((2-chloro-2-(trimethylsilyl)vinyl)tetrafluoro- λ^6-sulfaneyl)pyrimidine (1e^1)

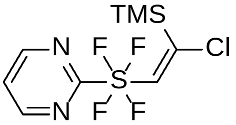

Yield: (3332 mg, 39%), Color: white solid. mp: 103.0–104.1 °C (10% EtOAc/hexane). ^{1}H NMR (300 MHz, $CDCl_3$) δ 8.89 (d, *J* = 4.8 Hz, 2H), 7.63 – 7.39 (m, 2H), 0.37 (d, *J* = 1.1 Hz, 9H). ^{19}F NMR (282 MHz, CDCl3) δ 53.42 (d, *J* = 9.1 Hz, 4F). ^{13}C NMR (126 MHz, $CDCl_3$) δ 173.88 (t, *J* = 36.4 Hz), 158.47, 152.85 (p, *J* = 27.6, 27.2 Hz), 147.22 (t, *J* = 9.0 Hz), 122.80, 0.40. LCMS (ESI+) m/z: [M + Na]+ Calcd for $C_9H_{13}ClF_4N_2SSiNa$ 343.0; found 343.0. ATR-FTIR (KBr): v = 3042, 3002, 2974, 2903, 2568, 2380, 1981, 1696, 1614, 1563, 1438, 1391, 1321, 1292 cm-1.

2-(ethynyltetrafluoro- λ^6-sulfaneyl)pyrimidine (1e)

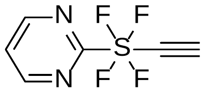

1e

Yield: (340 mg, 20%), Color: white solid. mp: 134.5–135.6 °C (100% hexane to 30% EtOAc/hexane) ^{1}H NMR (300 MHz, $CDCl_3$) δ 8.89 (d, *J* = 4.8 Hz, 2H), 7.50 (t, *J* = 4.8 Hz, 1H), 2.94 – 2.85 (m, 1H). ^{19}F NMR (282 MHz, $CDCl_3$) δ 69.80 (d, *J* = 3.5 Hz, 4F).^{13}C NMR (126 MHz, $CDCl_3$) δ 177.08 – 168.10 (m), 158.55, 123.18, 86.27 (t, *J* = 52.4 Hz), 66.91 – 56.68 (m). LCMS (ESI+) m/z: [M + H]+ Calcd for $C_6H_5F_4N_2S$ 213.0; found 213.0. ATR-FTIR (KBr): v = 3177, 3068, 2088, 1570, 1444, 1393, 1184, 995, 945, 804, 701, 626, 545, 497 cm^{-1}.

IV. General procedure and Characterizations of coupling products

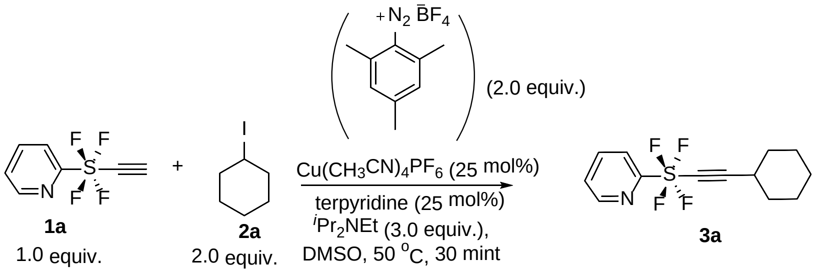

An oven-dried 4 mL vial equipped with a magnetic stir bar was charged with the alkyl iodide **2a** (84.0 mg, 0.4 mmol, 2.0 equiv.), $[Cu(CH_3CN)_4]PF_6$ (18.6 mg, 0.05 mmol, 0.25 equiv.), terpyridine (11.7 mg, 0.05 mmol, 0.25 equiv.), DIPEA (77.6 mg, 0.6 mmol, 3.0 equiv.) and Pyridine SF_4 alkyne **1a** (42.2 mg, 0.2 mmol, 1.0 equiv.) dissolved in 0.5 mL of dry DMSO, the dark brown solution was heated to 50 °C and was stirred for 5 min. Aryldiazonium salt (93.6 mg, 0.4 mmol, 2.0 equiv.) taken in dry DMSO (0.5 mL) slowly added to the vial within 5 minutes via syringe. The resultant mixture was stirred at 50 °C for an additional 10 minutes. The reaction mixture was then cooled down to room temperature, diluted with water (10 mL) and extracted with ethyl acetate (10 mL × 3). The organic layers were combined, washed with brine (10 mL) and concentrated under vacuo. The crude product was purified by silica gel column chromatography (5% EtOAc hexane use as eluent) to afford the desired product as a brick red colored solid **3aa** (54.0 mg 92% yield).

2-((cyclohexylethynyl)tetrafluoro-*λ*^{6}-sulfaneyl)pyridine (**3aa**)

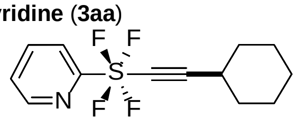

Prepared according to general procedure with **1a** (0.2 mmol) and **2a** (0.4 mmol, 2.0 equiv) at 50 °C for 5 mints to give **3a** (54.0 mg, 92% yield) as a brick red solid. mp: 63.0 – 64.0 °C (5% EtOAc/hexane). ^{1}H NMR (500 MHz, $CDCl_3$) *δ* 8.53 (dd, *J* = 5.0, 1.9 Hz, 1H), 7.85 (t, *J* = 8.2 Hz, 1H), 7.72 (d, *J* = 8.2 Hz, 1H), 7.41 (dd, *J* = 7.0, 4.3 Hz, 1H), 2.58-2.54 (m, 1H), 1.88-1.83 (m, 2H), 1.78-1.72 (m, 2H), 1.61-1.51 (m, 3H), 1.40-1.32 (m, 3H). ^{19}F NMR (282 MHz, $CDCl_3$) *δ* 76.09 (d, *J* = 3.7 Hz, 4F). ^{13}C NMR (126 MHz, $CDCl_3$) 169.92 (q, *J* = 29.5 Hz), 147.46, 138.43, 126.01, 121.31, 86.44 (p, *J* = 50.8 Hz), 79.20-79.05 (m), 31.35, 27.91, 25.68, 24.53. HRMS (ESI+) Calculated for $C_{13}H_{15}NF_4NaS$ $[M + H]^+$ 316.0759; found 316.0759. ATR-FTIR (KBr): v = 3062, 2935, 2858, 2230, 1578, 1457, 1427, 774, 642 cm^{-1}.

2-((cycloheptylethynyl)tetrafluoro-*λ*^{6}-sulfaneyl)pyridine (**3ab**)

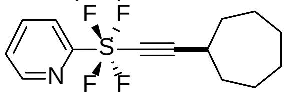

Prepared according to general procedure with **1a** (0.2 mmol) and **2b** (0.4 mmol, 2.0 equiv) at 50 °C for 5 mints to give **3ab** (52.2 mg, 85%), as a brick red color thick liquid. (5% EtOAc/hexane). ^{1}H NMR (500 MHz, $CDCl_3$) δ 8.53 (dd, *J* = 4.8, 1.9 Hz, 1H), 7.86 (t, *J* = 8.2 Hz, 1H), 7.72 (d, *J* = 8.3 Hz, 1H), 7.42 (dd, *J* = 7.4, 4.7 Hz, 1H), 2.78-2.73 (m, 1H), 1.92-1.86 (m, 2H), 1.81-1.72 (m, 4H), 1.63-1.51 (m, 6H). ^{19}F NMR (282 MHz, $CDCl_3$) *δ* 76.11 (d, *J* = 3.7 Hz, 4F). ^{13}C NMR (126 MHz, $CDCl_3$) 173.24-165.98 (m), 147.43, 138.45, 126.03, 121.29, 86.76 (p, *J* = 50.9 Hz), 79.95-79.80 (m), 33.36, 29.89, 27.71, 25.58. HRMS (ESI+) Calculated for $C_{14}H_{17}F_4NSNa$ $[M + Na]^+$ 330.0916 found 330.0914. ATR-FTIR (KBr): v = 2928, 2868, 2228, 1574, 1455, 1431, 1056, 770 cm^{-1}.

2-((cyclooctylethynyl)tetrafluoro-*λ*^{6}-sulfaneyl)pyridine (**3ac**)

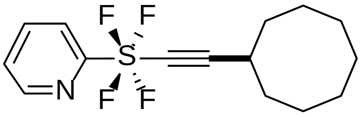

Prepared according to general procedure with **1a** (0.2 mmol) and **2c** (0.4 mmol, 2.0 equiv) at 50 °C for 5 mints to give **3ac** (58.0 mg, 90%), as a yellow colored thick liquid. (5% EtOAc/hexane). ^{1}H NMR (500 MHz, $CDCl_3$) δ 8.53 (dd, *J* = 4.8, 1.9 Hz, 1H), 7.86 (t, *J* = 8.2 Hz, 1H), 7.72 (d, *J* = 8.3 Hz, 1H), 7.42 (dd, *J* = 7.4, 4.7 Hz, 1H), 2.76-2.71 (m, 1H), 1.96-1.90 (m, 2H), 1.80-1.73 (m, 4H), 1.61-1.48 (m, 8H). ^{19}F NMR (282 MHz, $CDCl_3$) *δ* 76.17 (d, *J* = 3.7 Hz, 4F). ^{13}C NMR (126 MHz, $CDCl_3$) 169.80 (p, *J* = 29.7 Hz), 147.44, 138.46, 126.03, 121.30, 86.36 (p, *J* = 50.9 Hz), 80.27-80.12 (m), 30.56, 29.05, 27.11, 25.40, 24.45. HRMS(ESI+) Calculated for $C_{15}H_{19}F_4NSNa$ $[M + Na]^+$ 344.1072 found 344.1071 ATR-FTIR (KBr): v = 2933, 2854, 2232, 1566, 1463, 1435, 993, 774 cm^{-1}.

2-((cyclopentylethynyl)tetrafluoro-*λ*^{6}-sulfaneyl)pyridine (**3ad**)

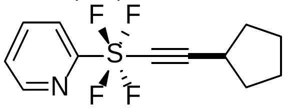

Prepared according to general procedure with **1a** (0.2 mmol) and **2d** (0.4 mmol, 2.0 equiv) at 50 °C for 5 mints to give **3ad** (50.2 mg, 90%) as a yellow-colored thick liquid. (5% EtOAc/hexane). ^{1}H NMR (300 MHz, $CDCl_3$) δ 8.53 (dd, *J* = 4.8, 1.9 Hz, 1H), 7.85 (t, *J* = 8.2 Hz, 1H), 7.71 (d, *J* = 8.2 Hz, 1H), 7.41 (dd, *J* = 7.4, 4.7 Hz, 1H), 2.80-2.73 (m, 1H), 2.05-1.95 (m, 2H), 1.82-1.72 (m, 4H), 1.65-1.58 (m, 2H). ^{19}F NMR (282 MHz, $CDCl_3$) *δ* 76.05 (d, *J* = 3.7 Hz, 4F). ^{13}C NMR (126 MHz, $CDCl_3$) 169.78 (p, *J* = 29.9 Hz), 147.43, 138.45, 126.03, 121.30, 85.79 (p, *J* = 51.1 Hz), 79.72-79.57 (m), 32.93, 28.77, 25.17.HRMS(ESI+) Calculated for $C_{12}H_{13}F_4NSNa$ for $[M + Na]^+$ 302.0603 found 302.0608. ATR-FTIR (KBr): v = 3064, 2960, 2884, 2232, 1574, 1458, 1431, 1340, 1303, 1088, 778 cm^{-1}.

2-(tetrafluoro(oxetan-3-ylethynyl)-λ^6-sulfaneyl)pyridine (**3ae**)

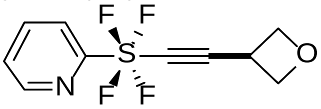

Prepared according to general procedure with **1a** (0.2 mmol) and **2e** (0.4 mmol, 2.0 equiv) at 50 °C for 5 mints to give **3ae** (37.4 mg, 70%) as a pale-yellow solid. mp: 69.8 – 70.7 °C (10% EtOAc/hexane). ^{1}H NMR (300 MHz, $CDCl_3$) δ 8.55 - 8.53 (m, 1H), 7.87 (t, *J* = 8.2 Hz, 1H), 7.72 (d, *J* = 8.2 Hz, 1H), 7.46 – 7.41 (m, 1H), 4.88 – 4.80 (m, 4H), 4.06 - 3.95 (m, 1H). ^{19}F NMR (282 MHz, $CDCl_3$) *δ* 75.45 (d, *J* = 3.3 Hz, 4F). ^{13}C NMR (126 MHz, $CDCl_3$) 169.20 (p, *J* = 28.7 Hz), 147.57, 138.58, 126.28, 121.29, 89.27 (p, *J* = 52.8 Hz), 75.48, 73.20 (q, *J* = 9.8 Hz), 24.63. HRMS(ESI+) Calculated for $C_{15}H_{19}F_4NSNa$ $[M + Na]^+$ $C_{10}H_9NF_4NaOS$ 290.0239; found 290.0246. ATR-FTIR (KBr): v = 2968, 2892, 2236, 2219, 1582, 1463, 1431, 1303, 1160, 1140 cm^{-1}.

2-(tetrafluoro((tetrahydrofuran-3-yl)ethynyl)-*λ*^{6}-sulfaneyl)pyridine (**3af**)

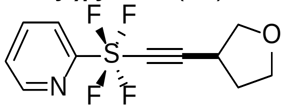

Prepared according to general procedure with **1a** (0.2 mmol) and **2f** (0.4 mmol, 2.0 equiv) at 50 °C for 5 mints to give **3af** (49.4 mg, 88%) as an off white solid. mp: 51.5 – 52.4 °C (10% EtOAc/hexane). ^{1}H NMR (500 MHz, $CDCl_3$) δ 8.52 (dd, *J* = 4.7, 1.1 Hz, 1H), 7.85 (t, *J* = 8.2 Hz, 1H), 7.70 (d, *J* = 8.2 Hz, 1H), 7.41 (dd, *J* = 7.4, 4.7 Hz, 1H), 4.08-4.05 (m, 1H), 3.96 – 3.84 (m, 2H), 3.77 - 3.74 (m, 1H), 3.17 – 3.11 (m, 1H), 2.32 – 2.25 (m, 1H), 2.13 – 2.07 (m 1H). ^{19}F NMR (282 MHz, $CDCl_3$) *δ* 75.66 (d, *J* = 3.4 Hz, 4F). ^{13}C NMR (126 MHz, $CDCl_3$) 169.40 (p, *J* = 29.4 Hz), 147.50, 138.50, 126.16, 121.27 (t, *J* = 4.6 Hz), 86.94 (p, *J* = 52.3 Hz), 75.46 (p, *J* = 9.7 Hz), 72.30, 68.04, 32.70, 28.96. HRMS(ESI+) Calculated for $C_{15}H_{19}F_4NSNa$ $[M + Na]^+$ $C_{11}H_{11}NF_4NaOS$ 304.0395; found 304.0396. ATR-FTIR (KBr): v = 3059, 2985, 2944, 2868, 2231, 1580, 1457, 1429, 1065 cm^{-1}.

2-(tetrafluoro((tetrahydro-2H-pyran-4-yl)ethynyl)-*λ*^{6}-sulfaneyl)pyridine (**3ag**)

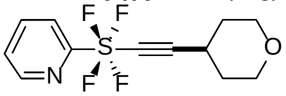

Prepared according to general procedure with **1a** (0.2 mmol) and **2g** (0.4 mmol, 2.0 equiv) at 50 °C for 5 mints to give **3ag** (49.0 mg, 83%) as an off white solid. mp: 94.7 – 95.3 °C (10% EtOAc/hexane). ^{1}H NMR (500 MHz, $CDCl_3$) δ 8.53 (dd, *J* = 4.8, 1.8 Hz, 1H), 7.86 (t, *J* = 7.9 Hz, 1H), 7.71 (d, *J* = 8.2 Hz, 1H), 7.42 (dd, *J* = 7.4, 4.7 Hz, 1H), 3.94 -3.89 (m, 2H), 3.58 – 3.53 (m, 2H), 2.83 – 2.79 (m, 1H), 1.92 – 1.88 (m, 2H), 1.80 – 1.72 (m, 2H). ^{19}F NMR (282 MHz, $CDCl_3$) *δ* 75.92 (d, *J* = 3.8 Hz, 4F). ^{13}C NMR (126 MHz, $CDCl_3$) 169.49 (p, *J* = 29.3 Hz), 147.51, 138.52, 126.16, 121.28, 87.17 (p, *J* = 51.8 Hz), 66.04, 30.89, 25.17. HRMS(ESI+) Calculated for $C_{12}H_{13}F_4NOSNa$ for $[M + Na]^+$ 318.0552 found 318.0556. ATR-FTIR (KBr): v = 3079, 2960, 2849, 2239, 1570, 1467, 1423, 1243, 1129, 1092, 993, 781 cm^{-1}.

2-(tetrafluoro((tetrahydro-2H-thiopyran-4-yl)ethynyl)-λ^6-sulfaneyl)pyridine (**3ah**)

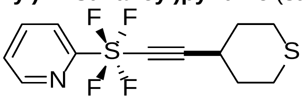

Prepared according to general procedure with **1a** (0.2 mmol) and **2h** (0.4 mmol, 2.0 equiv) at 50 °C for 5 mints to give **3ah** (50.3 mg, 81%) as an off white solid. mp: 110 – 111.2 °C (10% EtOAc/hexane). ^{1}H NMR (500 MHz, $CDCl_3$) δ 8.54 (ddd, *J* = 4.6, 1.9, 0.8 Hz, 1H), 7.92 – 7.84 (m, 1H), 7.72 (d, *J* = 8.3 Hz, 1H), 7.43 (ddd, *J* = 7.5, 4.7, 0.9 Hz, 1H), 2.92 – 2.87 (m, 2H), 2.80 – 2.76 (m, 1H), 2.58 – 2.53 (m, 2H), 2.17 – 2.10 (m, 2H) 2.04 – 1.97 (m, 2H). ^{19}F NMR (282 MHz, $CDCl_3$) *δ* 76.01 (d, *J* = 3.4 Hz, 4F). ^{13}C NMR (126 MHz, $CDCl_3$) 169.49 (p, *J* = 29.7 Hz), 147.54, 138.54, 126.18, 121.30, 88.52 – 88. 69 (m), 31.73, 27.29, 26.04. HRMS(ESI+) Calculated for $C_{12}H_{13}F_4NS_2Na$$[M + Na]^+$ 334.0323 found 334.0309. ATR-FTIR (KBr): v = 3060, 2948, 2920, 2247, 1570, 1463, 1431, 1280, 1084 cm^{-1}.

***t*ert-butyl 3-((tetrafluoro(pyridin-2-yl)-λ^6-sulfaneyl)ethynyl)pyrrolidine-1-carboxylate** (**3ai**)

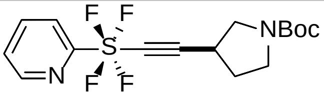

Prepared according to general procedure with **1a** (0.2 mmol) and **2i** (0.4 mmol, 2.0 equiv) at 50 °C for 5 mints to give 3ai (66.12 mg, 87%) as a brick red colored thick liquid. (10% EtOAc/hexane). ^{1}H NMR (300 MHz, $CDCl_3$) δ 8.54 (dd, *J* = 4.8, 1.9 Hz, 1H), 7.87 (t, *J* = 7.9 Hz, 1H), 7.71 (d, *J* = 8.3 Hz, 1H), 7.44 (dd, *J* = 7.4, 4.7 Hz, 1H), 3.73 – 3.35 (m, 4H), 3.13 – 3.09 (m, 1H), 2.27 – 2.22 (m, 1H), 2.11 – 2.04 (m, 1H), 1.47 (s, 9H). ^{19}F NMR (282 MHz, $CDCl_3$) *δ* 75.60 (s, 4F). ^{13}C NMR (126 MHz, $CDCl_3$) 169.26 (p, *J* = 29.0 Hz), 154.17,147.49, 138.52, 126.19, 121.24, 87.04 (p, *J* = 52.6 Hz), 79.68 (d, *J* = 12.6 Hz), 74.56, 50.43 (d, *J* = 34.8 Hz), 44.92 (d, *J* = 28.8 Hz), 31.45 (d, *J* = 99.2 Hz), 28.4 (d, *J* = 91.98 Hz), 28.44. HRMS(ESI+) Calculated for $C_{16}H_{20}F_4N_2NaO_2S$ $[M + Na]^+$ 403.1079 found 403.1070. ATR-FTIR (KBr): v = 2976, 2900, 2243, 1690, 1574, 1411, 1160, 886, 781 cm^{-1}.

***t*ert-butyl 3-((tetrafluoro(pyridin-2-yl)-λ^6-sulfaneyl)ethynyl)azetidine-1-carboxylate** (**3aj**)

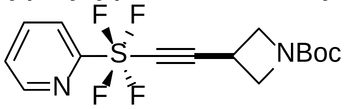

Prepared according to general procedure with **1a** (0.2 mmol) and **2j** (0.4 mmol, 2.0 equiv) at 50 °C for 5 mints to give **3aj** (64.41 mg, 88%) as a pale-yellow solid. mp: 115.5 – 116.5 °C (10% EtOAc/hexane). ^{1}H NMR (500 MHz, $CDCl_3$) δ 8.52 (dd, *J* = 4.7, 1.1 Hz, 1H), 7.86 (t, *J* = 8.3 Hz, 1H), 7.70 (d, *J* = 8.2 Hz, 1H), 7.42 (dd, *J* = 7.4, 4.7 Hz, 1H), 4.19 (t, *J* = 8.6 Hz, 2H), 4.06 – 4.03 (m, 2H), 3.49 – 3.43 (m, 1H), 1.43 (s, 9H). ^{19}F NMR (282 MHz, $CDCl_3$) *δ* 75.47 (d, *J* = 3.6 Hz, 4F). ^{13}C NMR (126 MHz, $CDCl_3$) 169.15 (p, *J* = 28.9 Hz), 155.78,147.53, 138.56, 126.26, 121.26, 88.51 (p, *J* = 53.0 Hz), 80.10, 74.37 – 73.85 (m), 54.26, 28.31, 18.05. HRMS (ESI+) Calculated for $C_{15}H_{18}F_4N_2NaO_2S$ $[M + Na]^+$ 389.0923 found 389.0923. ATR-FTIR (KBr): v = 3056, 2976, 2892, 2232, 1694, 1582, 1471, 1407, 1152, 781 cm^{-1}.

2-(((1,4-dioxaspiro[4.5]decan-8-yl)ethynyl)tetrafluoro-λ^6-sulfaneyl)pyridine (**3ak**)

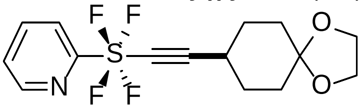

Prepared according to general procedure with **1a** (0.2 mmol) and **2k** (0.4 mmol, 2.0 equiv) at 50 °C for 5 mints to give **3ak** (64.6 mg, 92%) as an off white solid. mp: 111.3 – 112.0 °C (10% EtOAc/hexane). ^{1}H NMR (500 MHz, $CDCl_3$) δ 8.50 (dd, *J* = 4.9, 1.8 Hz, 1H), 7.83 (t, *J* = 7.5 Hz, 1H), 7.68 (d, *J* = 8.2 Hz, 1H), 7.39 (dd, *J* = 7.6, 4.7 Hz, 1H), 3.93 – 3.92 (m, 4H), 2.63 – 2.58 (m, 1H), 1.95 – 1.89 (m, 2H), 1.86 – 1.78 (m, 4H), 1.60 – 1.54 (m, 2H). ^{19}F NMR (282 MHz, $CDCl_3$) *δ* 75.96 (d, *J* = 3.2 Hz, 4F). ^{13}C NMR (126 MHz, $CDCl_3$) 169.55 (p, *J* = 29.5 Hz), 147.42, 138.48, 126.08, 121.25, 107. 78, 86.58 (p, *J* = 51.4 Hz), 77.96 – 77.51 (m), 64.30, 32.74, 28.54, 26.32. HRMS (ESI+) Calculated for $C_{15}H_{17}F_4NO_2SNa$ $[M + Na]^+$ 374.0814 found 374.0811. ATR-FTIR (KBr): v = 3088, 2960, 2888, 2223, 1718, 1578, 1458, 1431, 1359, 1140, 1088, 785 cm^{-1}.

2-(4-((tetrafluoro(pyridin-2-yl)-λ^6-sulfaneyl)ethynyl)piperidin-1-yl)pyrimidine (**3al**)

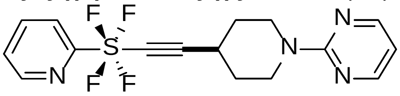

Prepared according to general procedure with **1a** (0.2 mmol) and **2l** (0.4 mmol, 2.0 equiv) at 50 °C for 5 mints to give **3al** (55.80 mg, 75%) as a pale-yellow solid. mp: 103.8 – 104.6 °C (10% EtOAc/hexane). ^{1}H NMR (500 MHz, $CDCl_3$) δ 8.51 (dd, *J* = 4.7, 1.4 Hz, 1H), 8.29 (d, *J* = 4.9, 2H), 7.84 (t, *J* = 7.5 Hz, 1H), 7.69 (d, *J* = 8.2 Hz, 1H), 7.40 (dd, *J* = 7.5, 4.7 Hz, 1H), 6.46 (t, *J* = 4.9 1H), 4.19 – 4.15 (m, 2H), 3.63 – 3.58 (m, 2H), 2.87 – 2.82 (m, 1H), 1.96 – 1.91 (m, 2H), 1.80 – 1.74 (m, 2H). ^{19}F NMR (282 MHz, $CDCl_3$) *δ* 75.97 (d, *J* = 3.6 Hz, 4F). ^{13}C NMR (126 MHz, $CDCl_3$) 169.48 (p, *J* = 29.1 Hz), 161.50, 157.77, 147.48, 138.49, 126.13, 121.26, 109. 76, 87.39 (p, *J* = 51.8 Hz), 42.14, 30.14, 26.41. HRMS (ESI+) Calculated for $C_{16}H_{16}F_4N_4SNa$ $[M + Na]^+$ 395.0929 found 395.0923. ATR-FTIR (KBr): v = 3079, 3036, 2937, 2853, 2236, 1598, 1542, 1507, 1439, 1224, 1084, 977, 774 cm^{-1}.

2-(((2,3-dihydro-1*H*-inden-2-yl)ethynyl)tetrafluoro-λ^6-sulfaneyl)pyridine (**3am**)

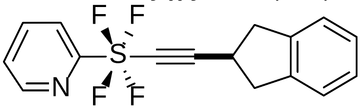

Prepared according to general procedure with **1a** (0.2 mmol) and **2m** (0.4 mmol, 2.0 equiv) at 50 °C for 5 mints to give **3am** (58.20 mg, 89%) as a brick red colored solid. mp: 40 - 41 °C (5% EtOAc/hexane). ^{1}H NMR (500 MHz, $CDCl_3$) δ 8.54 (dd, *J* = 4.8, 1.9 Hz, 1H), 7.86 (t, *J* = 7.9 Hz, 1H), 7.72 (d, *J* = 8.3 Hz, 1H), 7.42 (dd, *J* = 7.4, 4.7 Hz, 1H), 7.24 – 7.18 (m, 4H), 3.39 – 3.31 (m, 3H), 3.32 – 3.16 (m, 2H). ^{19}F NMR (282 MHz, $CDCl_3$) *δ* 75.87 (s, 4F). ^{13}C NMR (126 MHz, $CDCl_3$) 169.57 (p, *J* = 29.4 Hz), 147.49, 141.14, 138.50, 126.84, 126.13, 124.39, 121.30, 86.11 (p, *J* = 51.7 Hz), 78.0 – 77.76 (m), 39.26, 28.59. HRMS (ESI+) Calculated for $C_{16}H_{14}F_4NS$ $[M + H]^+$ 328.0783 found 328.0770. ATR-FTIR (KBr): v = 2964, 2875, 2234, 1581, 1461, 1425, 1370, 1055, 670, cm^{-1}.

2-((((2R)-2-ethoxycyclohexyl)ethynyl)tetrafluoro-λ^6-sulfaneyl)pyridine (dr ratio = 1 : 3.2) (**3an**)

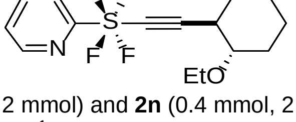

Prepared according to general procedure with **1a** (0.2 mmol) and **2n** (0.4 mmol, 2.0 equiv) at 50 °C for 5 mints to give **3an** (49.87 mg, 74%) as a pale yellow thick liquid. (5% EtOAc/hexane). ^{1}H NMR (500 MHz, $CDCl_3$) δ 8.54 (m, 1H), 7.86 (t, *J* = 7.3 Hz, 1H), 7.72 (d, *J* = 8.2 Hz, 1H), 7.43 – 7.40 (m,1H), 3.68 (q, *J* = 7.0 Hz, 2H), 3.45 – 3.42 (m, 1H, minor) 3.33 – 3.29 (m, 1H, major) 3.03 – 3.00 (m, 1H, minor) 3.25 – 2.49 (m, 1H, major), 2.05 – 1.93 (m, 2H), 1.78 – 1.42 (m, 4H), 1.35 – 1.30 (m, 2H), 1.24 (t, *J* = 7.0 Hz, 3H). ^{19}F NMR (282 MHz, $CDCl_3$) *δ* 75.98 (d, *J* = 4.3, 4F, minor) 75.72 (d, J = 3.3, 4F, major). ^{13}C NMR (126 MHz, $CDCl_3$) 169.66 (p, *J* = 29.4 Hz), 147.43, 138.45, 126.05, 121.28, 86.76 – 86.10 (m, major and minor), 79.02, 65.44 (major), 63.89 (minor), 35.71, 32.25, 31.05, 29.55, 29.08,

28.21, 24.03, 23.37, 22.89, 22.48, 15.58. HRMS (ESI+) Calculated for $C_{15}H_{19}F_4NOSNa$ [M + Na]$^+$ 360.1021 found 360.1021. ATR-FTIR (KBr): ν = 2972, 2937, 2864, 2236, 1582, 1451, 1423, 1108, 1092, 798 cm^{-1}.

2-(tetrafluoro(3-methylbut-1-yn-1-yl)-λ^6-sulfaneyl)pyridine (3ao)

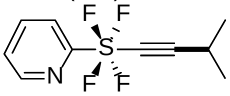

Prepared according to general procedure with **1a** (0.2 mmol) and **2o** (0.4 mmol, 2.0 equiv.) at 50 °C for 5 mints to give **3ao** (41.0 mg, 81%) as an off white solid. mp: 64 - 65 °C (5% EtOAc/hexane). ^{1}H NMR (500 MHz, $CDCl_3$) δ 8.52 (dd, *J* = 4.7, 1.2 Hz, 1H), 7.85 (t, *J* = 7.9 Hz, 1H), 7.70 (d, *J* = 8.2 Hz, 1H), 7.41 (dd, *J* = 7.4, 4.7 Hz, 1H), 2.74 – 2.66 (m, 1H), 1.26 (d, *J* = 6.9 Hz, 6H). ^{19}F NMR (282 MHz, $CDCl_3$) *δ* 75.91 (d, *J* = 3.8, 4F). ^{13}C NMR (126 MHz, $CDCl_3$) 169.71 (p, *J* = 29.5 Hz), 147.46, 138.48, 126.07, 121.32, 85.49 (p, *J* = 51.4 Hz), 81.75 – 78.62 (m), 21.75, 19.68. HRMS (ESI+) Calculated for $C_{10}H_{11}F_4NSNa$ [M + Na]$^+$ 276.0446 found 276.0452. ATR-FTIR (KBr): ν = 3079, 2984, 2940, 2877, 2236, 1574, 1463, 1427,1323, 1172, 1105, 981, 862 cm^{-1}.

2-((3-cyclohexylprop-1-yn-1-yl)tetrafluoro-λ^6-sulfaneyl)pyridine (3ap)

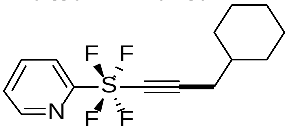

Prepared according to general procedure with **1a** (0.2 mmol) and **2p** (0.4 mmol, 2.0 equiv.) at 50 °C for 5 mints to give **3ap** (50.34 mg, 82%) as a pale-yellow solid. mp: 64.6 – 65.8 °C (5% EtOAc/hexane). ^{1}H NMR (300 MHz, $CDCl_3$) δ 8.53 (dd, *J* = 4.4, 1.7 Hz, 1H), 7.85 (t, *J* = 7.9 Hz, 1H), 7.71 (d, *J* = 8.3 Hz, 1H), 7.41 (dd, *J* = 7.4, 4.7 Hz, 1H), 2.26 – 2.21 (m, 2H), 1.87 – 1.59 (m, 6H), 1.35 – 0.99 (m, 5H). ^{19}F NMR (282 MHz, $CDCl_3$) *δ* 75.94 (d, *J* = 4.1, 4F). ^{13}C NMR (126 MHz, $CDCl_3$) 169.82 (p, *J* = 29.5 Hz), 147.44, 138.48, 126.07, 121.28, 87.02 (p, *J* = 51.4 Hz), 75.05 (t, *J* = 9.7 Hz), 36.49, 32.59, 26.07, 26.02, 25.34. HRMS (ESI+) Calculated for $C_{14}H_{17}F_4NSNa$ [M + Na]$^+$ 330.0916 found 330.0917. ATR-FTIR (KBr): ν = 3092, 3050, 2933, 2849, 2236, 1574, 1458, 1427, 1045, 781 cm^{-1}.

2-((4,4-dimethylpent-1-yn-1-yl)tetrafluoro-λ^6-sulfaneyl)pyridine (3aq)

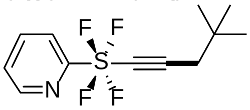

Prepared according to general procedure with **1a** (0.2 mmol) and **2q** (0.4 mmol, 2.0 equiv.) at 50 °C for 5 mints to give **3aq** (46.08 mg, 82%) as a thick liquid. (5% EtOAc/hexane). ^{1}H NMR (500 MHz, $CDCl_3$) δ 8.52 (dd, *J* = 4.9, 1.9 Hz, 1H), 7.85 (t, *J* = 7.9 Hz, 1H), 7.71 (d, *J* = 8.3 Hz, 1H), 7.41 (dd, *J* = 7.4, 4.3 Hz, 1H), 2.22 – 2.19 (m, 2H), 1.05 (s, 9H). ^{19}F NMR (282 MHz, $CDCl_3$) *δ* 76.0 (d, *J* = 4.5, 4F). ^{13}C NMR (126 MHz, $CDCl_3$) 169.69 (p, *J* = 29.5 Hz), 147.45, 138.47, 126.07, 121.28, 87.68 (p, *J* = 51.1 Hz), 74.68 (t, *J* = 10.0 Hz), 32.45, 31.38, 29.01. HRMS (ESI+) Calculated for $C_{12}H_{15}F_4NSNa$ [M + Na]$^+$ 304.0759 found 304.0764. ATR-FTIR (KBr): ν = 2964, 2875, 2234, 1581, 1461, 1425, 1054, 778, 671 cm^{-1}.

2-((5-(4-bromophenyl)pent-1-yn-1-yl)tetrafluoro-λ^6-sulfaneyl)pyridine (3ar)

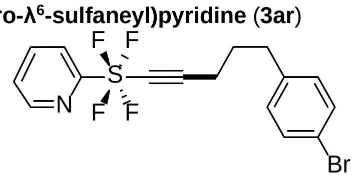

Prepared according to general procedure with **1a** (0.2 mmol) and **2r** (0.4 mmol, 2.0 equiv.) at 50 °C for 5 mints to give **3ar** (65.28 mg, 80%) as a brick red colored liquid. (5% EtOAc/hexane). ^{1}H NMR (500 MHz, $CDCl_3$) δ 8.53 (dd, *J* = 4.7, 1.2 Hz, 1H), 7.86 (t, *J* = 8.3 Hz, 1H), 7.72 (d, *J* = 8.2 Hz, 1H), 7.43 – 7.39 (m, 3H), 7.10 – 7.07 (m, 2H), 2.73 (t, *J* = 7.5 Hz, 2H), 2.31 (tt, *J* = 7.0, 3.6 Hz, 2H), 1.89 (p, *J* = 7.0 2H). ^{19}F NMR (282 MHz, $CDCl_3$) *δ* 75.78 (d, *J* = 4.0, 4F). ^{13}C NMR (126 MHz, $CDCl_3$) 169.55 (p, *J* = 29.3 Hz), 147.49, 139.84, 138.54,131.54, 130.37, 126.18, 121.29, 119.89, 86.89 (p, *J* = 51.7 Hz), 76.30 – 73.79 (m), 33.84, 28.70, 16.83. HRMS (ESI+) Calculated for $C_{16}H_{14}F_4NSBrNa$ [M + Na]$^+$ 429.9864 found 429.9868. ATR-FTIR (KBr): ν = 3064, 2944, 2868, 2239, 1563, 1451, 1327, 1056, 785 cm^{-1}.

2-(5-(tetrafluoro(pyridin-2-yl)-λ^6-sulfaneyl)pent-4-yn-1-yl)isoindoline-1,3-dione (3as)

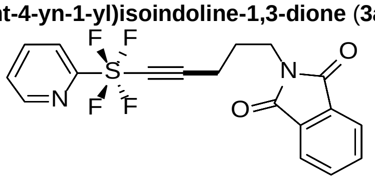

Prepared according to general procedure with **1a** (0.2 mmol) and **2s** (0.4 mmol, 2.0 equiv.) at 50 °C for 5 mints to give **3as** (47.76 mg, 60%) as a brick red colored solid. mp: 112.3 – 113.8 °C (10% EtOAc/hexane). ^{1}H NMR (300 MHz, $CDCl_3$) δ 8.51(dd, *J* = 4.7, 1.1 Hz, 1H), 7.87 – 7.81 (m, 3H), 7.74 – 7.65 (m, 3H), 7.40 (dd, *J* = 7.4, 4.7 Hz, 1H), 3.81 (t, *J* = 6.9 Hz, 2H), 2.48 – 2.39 (m, 2H), 2.03 (p, *J* = 7.3 2H). ^{19}F NMR (282 MHz, $CDCl_3$) *δ* 75.54 (d, *J* = 3.9, 4F). ^{13}C NMR (176 MHz, $CDCl_3$) 169.57 (t, *J* = 30.0 Hz), 168.41, 147.53, 138.49, 134.24, 132.15, 126.13, 123.44, 121.34, 86.58 (t, *J* = 51.7 Hz), 74.22 – 74.16 (m), 37.16, 26.70, 15.75. HRMS (ESI+) Calculated for $C_{18}H_{14}F_4N_2O_2SNa$ for [M + Na]$^+$ 421.0610 found 421.0610. ATR-FTIR (KBr): ν = 3056, 2952, 2928, 2849, 2243, 1774, 1698, 1574, 1399, 1112, 977, 770 cm^{-1}.

2-(tetrafluoro(4-(2-(2-(2-(4-nitrophenoxy)ethoxy)ethoxy)ethoxy)but-1-yn-1-yl)-λ^6-sulfaneyl)pyridine (3at)

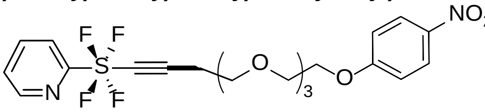

Prepared according to general procedure with **1a** (0.2 mmol) and **2t** (0.4 mmol, 2.0 equiv.) at 50 °C for 5 mints to give **3at** (77.21 mg, 76%) as a brick red colored gummy compound. (10% EtOAc/hexane). ^{1}H NMR (700 MHz, $CDCl_3$) δ 8.49 (dd, *J* = 4.8, 1.9 Hz, 1H), 8.15 (d, *J* = 9.2 Hz, 2H), 7.83 (t, *J* = 7.9 Hz, 1H), 7.67 (d, *J* = 8.3 Hz, 1H), 7.40 (dd, *J* = 7.4, 4.7 Hz, 1H), 6.95 (d, *J* = 9.2 Hz, 2H), 4.20 (t, *J* = 7.0 Hz, 2H), 3.88 (t, *J* = 7.0 Hz, 2H), 3.73 – 3.71 (m, 2H), 3.69 – 3.65 (m, 8H), 2.61 (dt, *J* = 7.0, 3.5 Hz, 2H). ^{19}F NMR (282 MHz, $CDCl_3$) *δ* 75.45 (t, *J* = 3.9, 4F). ^{13}C NMR (176 MHz, $CDCl_3$) 169.57 (p, *J* = 29.92 Hz), 163.99, 147.55, 141.63, 138.59, 126.24, 125.93, 121.30 (t, *J* = 3.5 Hz), 114.68, 87.15, 86.71 (p, *J* = 52.0 Hz) 72.78 (p, *J* = 9.6 Hz), 70.99, 70.79, 70.71, 70.69, 69.43, 68.31, 68.23, 19.29. HRMS (ESI+) Calculated for $C_{21}H_{24}F_4N_2O_6SNa$ [M + Na]$^+$ 531.1189 found 531.1191. ATR-FTIR (KBr): v = 3107, 3072, 2873, 2236, 1590, 1514, 1331, 1271, 1120, 1060 cm^{-1}.

2-((adamantan-1-ylethynyl)tetrafluoro-λ^6-sulfaneyl)pyridine (3au)

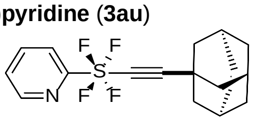

Prepared according to general procedure with **1a** (0.2 mmol) and **2u** (0.4 mmol, 2.0 equiv.) at 50 °C for 5 mints to give **3au** (51.06 mg, 74%) as a brick red colored solid. mp: 126 - 127 °C (5% EtOAc/hexane). ^{1}H NMR (500 MHz, $CDCl_3$) δ 8.52 (dd, *J* = 4.8, 1.8 Hz, 1H), 7.84 (t, *J* = 8.0 Hz, 1H), 7.70 (d, *J* = 8.3 Hz, 1H), 7.40 (dd, *J* = 7.4, 4.7 Hz, 1H), 1.99 – 1.95 (m, 9H), 1.71 (s, 6H). ^{19}F NMR (282 MHz, $CDCl_3$) *δ* 75.45 (s, 4F). ^{13}C NMR (126 MHz, $CDCl_3$) 169.82 (p, *J* = 29.7 Hz), 147.42, 138.42, 125.98, 121.30, 85.49 (p, *J* = 50.9 Hz) 82.70 – 81.55 (m), 41.48, 36.11, 28.92, 27.60. HRMS (ESI+) Calculated for $C_{17}H_{19}F_4NSNa$ [M + Na]$^+$ 368.1072 found 368.1076. ATR-FTIR (KBr): v = 3064, 2912, 2860, 2845, 2219, 1578, 1458, 1427, 1160, 1101 cm^{-1}.

2-(tetrafluoro((4-methyltetrahydro-2*H*-pyran-4-yl)ethynyl)-λ^6-sulfaneyl)pyridine (3av)

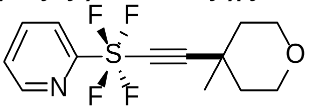

Prepared according to general procedure with **1a** (0.2 mmol) and **2v** (0.4 mmol, 2.0 equiv.) at 50 °C for 5 mints to give **3av** (21.63 mg, 35%) as an off white solid. mp: 127.7 – 128.9 °C (5% EtOAc/hexane). ^{1}H NMR (300 MHz, $CDCl_3$) δ 8.54 (dd, *J* = 4.7, 1.3 Hz, 1H), 7.87 (t, *J* = 8.0 Hz, 1H), 7.72 (d, *J* = 8.3 Hz, 1H), 7.43 (dd, *J* = 6.9, 4.2 Hz, 1H), 3.92 – 3.86 (m, 2H), 3.80 – 3.72 (m, 2H), 1.79 – 1.73 (m, 2H) 1.65 – 1.64 (m, 1H), 1.60 – 1.55 (m, 1H), 1.34 (s, 3H). ^{19}F NMR (282 MHz, $CDCl_3$) *δ* 76.29 (s, 4F). ^{13}C NMR (126 MHz, $CDCl_3$) 169.55 (t, *J* = 28.16 Hz), 147.51, 138.49, 126.12, 121.25, 88.64 (t, *J* = 50.6 Hz), 79.48, 64.97, 38.35, 30.10, 28.75. HRMS (ESI+) Calculated for $C_{13}H_{15}F_4NOSNa$ [M + Na]$^+$ 332.0708 found 332.0716. ATR-FTIR (KBr): v = 2968, 2924, 2860, 2243, 2215, 1570, 1467, 1423, 1112, 770 cm^{-1}.

2-((cyclohexylethynyl)tetrafluoro-λ^6-sulfaneyl)-5-(trifluoromethyl)pyridine (3ba)

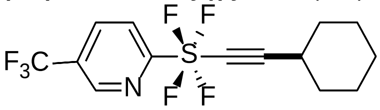

Prepared according to general procedure with **1b** (0.1 mmol) and **2a** (0.2 mmol, 2.0 equiv.) at 50 °C for 5 mints to give **3ba** (28.0 mg, 77.0%) as an off white solid. mp: 124.3 – 125.6 °C (10% EtOAc/hexane). ^{1}H NMR (500 MHz, $CDCl_3$) δ 8.81 (d, *J* = 2.3 Hz, 2H), 8.11 (d, *J* = 8.5, 1H), 7.86 (d, *J* = 8.5 Hz, 1H), 2.60 - 2.55 (m, 1H), 1.89 – 1.84 (m, 2H), 1.78 – 1.72 (m, 2H), 1.62 – 1.53 (m, 3H), 1.41 – 1.35 (m, 3H). ^{19}F NMR (282 MHz, $CDCl_3$) *δ* 77.02 (s, 4F), -63.03 (s, 3F). ^{13}C NMR (126 MHz, $CDCl_3$) 171.56 (p, *J* = 32.2 Hz), 144.83, 135.89 (d, *J* = 3.6 Hz), 128.80 (q, *J* = 33.8 Hz), 127.92, 122.57 (d, *J* = 272.9 Hz), 121.56 (p, *J* = 4.5 Hz), 85.79 (p, *J* = 50.1 Hz), 80.08 (t, *J* = 9.5 Hz), 31.26, 27.89, 25.63, 24.49. (ESI+) $C_{14}H_{15}F_7NS$ [M + H]$^+$ 362.05. ATR-FTIR (KBr): v = 3068, 2943, 2859, 2236, 1596, 1578, 1175, 1082, 584 cm^{-1}.

2-((((3r,5r,7r)-adamantan-1-yl)ethynyl)tetrafluoro-λ^6-sulfaneyl)-5-(trifluoromethyl)pyridine (3bu)

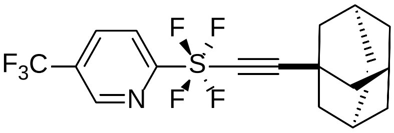

Prepared according to general procedure with **1b** (0.1 mmol) and **2a** (0.2 mmol, 2.0 equiv.) at 50 °C for 5 mints to give **3bu** (28.1 mg, 68%) as an off white solid. mp: 202.6 – 203.9 °C (10% EtOAc/hexane). ^{1}H NMR (500 MHz, $CDCl_3$) δ 8.80 (d, *J* = 2.3 Hz, 2H), 8.10 (d, *J* = 8.5, 1H), 7.85 (d, *J* = 8.5 Hz, 1H), 2.02 – 1.96 (m, 9H), 1.73 – 1.72 (m, 6H). ^{19}F NMR (282 MHz, $CDCl_3$) *δ* 77.30 (s, 4F), -63.03 (s, 3F). ^{13}C NMR (126 MHz, $CDCl_3$) 171.62 (p, *J* = 32.5, 31.7 Hz), 144.82, 135.87 (d, *J* = 3.6 Hz), 128.76 (q, *J* = 33.8 Hz), 127.92, 122.58 (d, *J* = 272.9 Hz), 121.56 (p, *J* = 3.9, 3.3 Hz), 84.90 (p, *J* = 49.8 Hz), 83.33 – 82.61 (m),41.43, 36.10, 28.98, 27.58. ATR-FTIR (KBr): v = 2857, 2222, 1595, 1576, 1454, 1077, 937, 578 cm^{-1}.

3-((cyclohexylethynyl)tetrafluoro-λ^6-sulfaneyl)-2-fluoropyridine (3ca)

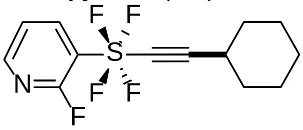

Prepared according to general procedure with **1c** (0.2 mmol) and **2a** (0.4 mmol, 2.0 equiv.) at 50 °C for 5 mints to give **3ca** (52.47 mg, 84%) as a red color solid. mp: 66.2 – 67.2 °C (5% EtOAc/hexane). ^{1}H NMR (500 MHz, $CDCl_3$) δ 8.30 (d, *J* = 4.7 Hz, 1H), 8.17 (td, *J* = 8.3, 1.8 Hz, 1H), 7.28 (t, *J* = 7.5 Hz, 1H), 2.57 – 2.52 (m, 1H), 1.87 – 1.82 (m, 2H), 1.77 – 1.71 (m, 2H), 1.60 – 1.52 (m, 3H), 1.40 – 1.34 (m, 3H).^{19}F NMR (282 MHz, $CDCl_3$) *δ* 91.44 (dd *J* = 22.5, 3.6 Hz, 4F), -60.81 (pd, *J* = 21.8, 8.1 Hz, 1F). ^{13}C NMR (126 MHz, $CDCl_3$) 155.23 (d, *J* = 246.0 Hz),149.58 (d, *J* = 15.0 Hz), 140.75 (q, *J* = 27.8 Hz), 139.60, 121.44, 86.60 (p, *J* = 50.9, 50.4 Hz), 79.85 – 79.39 (m), 31.28, 27.84, 25.64, 24.51. HRMS (ESI+) Calculated for $C_{13}H_{15}F_5NS$ [M + H]$^+$ 312.0845 found 312.0846. ATR-FTIR (KBr): v = 3071, 2940, 2859, 2240, 1588, 1444, 1272, 862, 793, 752 cm^{-1}.

3-((4,4-dimethylpent-1-yn-1-yl)tetrafluoro-λ^6-sulfaneyl)-2-fluoropyridine (3cq)

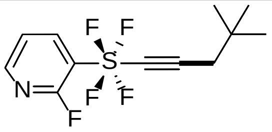

Prepared according to general procedure with **1c** (0.2 mmol) and **2q** (0.4 mmol, 2.0 equiv.) at 50 °C for 5 mints to give **3cq** (43.84 mg, 73%) as a red color thick liquid. (5% EtOAc/hexane). ^{1}H NMR (500 MHz, $CDCl_3$) δ 8.31 (d, *J* = 4.7 Hz, 1H), 8.17 (td, *J* = 8.3, 1.8 Hz 1H), 7.29 – 7.27 (m, 1H), 2.22 – 2.19 (m, 2H), 1.06 (s, 9H). ^{19}F NMR (282 MHz, $CDCl_3$) *δ* 91.26 (dt, *J* = 22.0, 3.9 Hz, 4F), -60.78 (tt, *J* = 22.3, 13.4 Hz, 1F). ^{13}C NMR (126 MHz, $CDCl_3$) 155.24 (d, *J* = 246.1 Hz),149.62 (d, *J* = 15.2 Hz), 140.68 (q, *J* = 27.8 Hz), 139.60, 121.44, 87.89 (p, *J* = 50.6 Hz), 75.03 (t, *J* = 9.9 Hz), 32.42, 31.37, 29.04. HRMS (ESI+) Calculated for $C_{12}H_{15}F_5NS$ [M + H]$^+$ 300.0845 found 300.0846. ATR-FTIR (KBr): ν = 2963, 2873, 2237, 1587, 1442, 1065, 862, 796, 757 cm^{-1}.

3-((adamantan-1-ylethynyl)tetrafluoro-λ^6-sulfaneyl)-2-fluoropyridine (**3cu**)

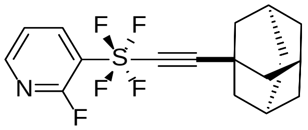

Prepared according to general procedure with **1c** (0.2 mmol) and **2u** (0.4 mmol, 2.0 equiv.) at 50 °C for 5 mints to give **3cu** (58.33 mg, 80%) as a red color solid. mp: 186 – 187.3 °C (5% EtOAc/hexane). ^{1}H NMR (500 MHz, $CDCl_3$) δ 8.30 (d, *J* = 4.7 Hz, 1H), 8.15 (td, *J* = 8.2, 1.7 Hz, 1H), 7.28 – 7.26 (m, 1H), 2.01 – 1.95 (m, 9H), 1.72 – 1.71 (m, 6H). ^{19}F NMR (282 MHz, $CDCl_3$) *δ* 91.71 (dt, *J* = 22.0 Hz, 4F), -60.80 (m, 1F). ^{13}C NMR (126 MHz, $CDCl_3$) 155.20 (d, *J* = 246.1 Hz),149.52 (d, *J* = 15.1 Hz), 140.78 (q, *J* = 28.1 Hz), 139.57, 121.42, 85.72 (p, *J* = 50.3 Hz), 82.38 – 82.16 (m), 41.43, 36.08, 28.90, 27.56. HRMS (ESI+) Calculated for $C_{17}H_{19}F_5NS$ [M + H]$^+$ 364.1158 found 364.1158. ATR-FTIR (KBr): ν = 2919, 2854, 2214, 1587, 1450, 1279, 862, 788, 761 cm-^{1}.

2-(4-((tetrafluoro(2-fluoropyridin-3-yl)-λ^6-sulfaneyl)ethynyl)piperidin-1-yl)pyrimidine (**3cl**)

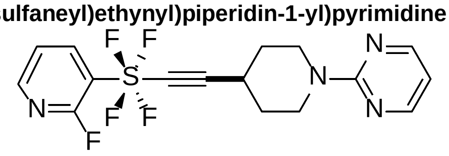

Prepared according to general procedure with **1c** (0.2 mmol) and **2l** (0.4 mmol, 2.0 equiv.) at 50 °C for 5 mints to give **3cl** (60.01 mg, 80%) as a pale-yellow solid. mp: 131.5 – 132.8 °C (10% EtOAc/hexane). ^{1}H NMR (300 MHz, $CDCl_3$) δ 8.31 (d, *J* = 4.7 Hz 3H), 8.16 (ddd, *J* = 8.5, 8.0, 1.8 Hz, 1H), 7.30 – 7.26 (m, 1H), 6.49 (t, *J* = 4.7 Hz, 1H), 4.23 – 4.15 (m, 2H), 3.66 – 3.58 (m, 2H), 2.90 – 2.82 (m, 1H), 2.00 – 1.91 (m, 2H), 1.83 – 1.72 (m, 2H). ^{19}F NMR (282 MHz, $CDCl_3$) *δ* 91.20 (dd, *J* = 21.9, 3.5 Hz, 4F), -60.81 (pd, J = 22.0, 8.1 Hz, 1F). ^{13}C NMR (126 MHz, $CDCl_3$) 160.53, 157.78, 155.17 (d, *J* = 246.0 Hz),149.70 (d, *J* = 15.2 Hz), 140.43 (q, *J* = 27.7 Hz), 139.55, 121.48, 109.84, 87.55 (p, *J* = 51.0 Hz), 42.15, 30.09, 26.38. HRMS (ESI+) Calculated for $C_{16}H_{16}F_5N_4S$ [M + H]$^+$ 391.1016 found 391.013. ATR-FTIR (KBr): ν = 3065, 2946, 2838, 2237, 1590, 1548, 1504, 1444, 1360, 1237, 759, 633 cm-^{1}.

4-((cyclohexylethynyl)tetrafluoro-l6-sulfaneyl)-2-fluoropyridine (**3da**)

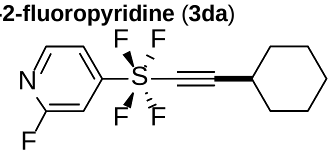

Prepared according to general procedure with **1d** (0.1 mmol) and **2a** (0.2 mmol, 2.0 equiv.) at 50 °C for 5 mints to give **3da** (20.2 mg, 65%) as a oil. (5% EtOAc/hexane). ^{1}H NMR (500 MHz, $CDCl_3$) δ 8.33 (d, *J* = 5.6 Hz, 1H), 7.50 (d, *J* = 5.7, 1H), 7.25 (t, *J* = 2.0 Hz, 1H), 2.58 – 2.53 (m, 1H), 1.87 – 1.83 (m, 2H), 1.77 – 1.71 (m, 2H), 1.58 – 1.53 (m, 3H), 1.40 – 1.34 (m, 3H).^{19}F NMR (282 MHz, $CDCl_3$) *δ* 85.82 (d *J* = 3.5 Hz, 4F), -65.48 (s, 1F). ^{13}C NMR (126 MHz, $CDCl_3$) 169.60 – 168.45 (m),163.52 (d, *J* = 241.1 Hz), 148.22 (d, *J* = 15.0 Hz), 118.20 (q, *J* = 4.9 Hz), 107.54 – 107.31 (m), 86.03 (p, *J* = 50.5 Hz), 79.99 (p, *J* = 9.5 Hz), 31.22, 27.84, 25.60, 24.49. MS (ESI+) Calculated for $C_{13}H_{14}F_5NSNa$ [M + Na]$^+$ 334.06 found 314.10. ATR-FTIR (KBr): ν = 2936, 2858, 2232, 1594, 1579, 1398, 1232, 793, 761, 641 cm^{-1}.

4-((((3r,5r,7r)-adamantan-1-yl)ethynyl)tetrafluoro-l6-sulfaneyl)-2-fluoropyridine (**3du**)

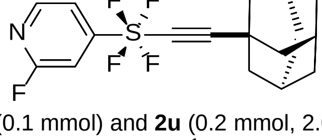

Prepared according to general procedure with **1d** (0.1 mmol) and **2u** (0.2 mmol, 2.0 equiv.) at 50 °C for 5 mints to give **3du** (22.2 mg, 61%) as an off white solid. mp: 138.2 – 139.0°C (5% EtOAc/hexane). ^{1}H NMR (500 MHz, $CDCl_3$) δ 8.32 (d, *J* = 5.7 Hz, 1H), 7.49 (dt, *J* = 5.6, 1.4 Hz, 1H), 7.24 (t, *J* = 1.9 Hz, 1H), 2.02 – 1.95 (m, 3H), 1.95 (s, 6H), 1.73 – 1.71 (m, 6H). ^{19}F NMR (282 MHz, $CDCl_3$) *δ* 86.11(s, 4F), -65.52 (m, 1F). ^{13}C NMR (126 MHz, $CDCl_3$) 169.01(qd, *J* = 28.0, 8.0 Hz),163.51 (d, *J* = 241.1 Hz), 148.20 (q, *J* = 15.0 Hz), 118.21 (q, *J* = 5.0 Hz), 107.59 (dt, *J* = 42.2, 5.4 Hz), 85.17 (p, *J* = 50.4 Hz), 82.84 (p, *J* = 9.6 Hz), 41.39, 36.06, 28.94, 27.54. MS (ESI+) Calculated for $C_{17}H_{19}F_5NS$ [M + H]$^+$ 364.11 found 364.10. ATR-FTIR (KBr): ν = 2909, 2855, 2223, 1591, 1577, 1472, 1452, 1397, 1098, 939, 794, 664, 579 cm-^{1}.

2-((cyclohexylethynyl)tetrafluoro-λ^6-sulfaneyl)pyrimidine (**3ea**)

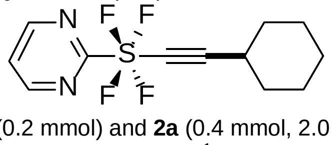

Prepared according to general procedure with **1e** (0.2 mmol) and **2a** (0.4 mmol, 2.0 equiv.) at 50 °C for 5 mints to give **3ea** (45.27 mg, 77%) as an off white solid. mp: 120.1 – 121.8 °C (5% EtOAc/hexane). ^{1}H NMR (300 MHz, $CDCl_3$) δ 8.88 (d, *J* = 4.9 Hz, 2H), 7.46 (t, *J* = 4.9 Hz, 1H), 2.62 – 2.55 (m, 1H), 1.90 – 1.84 (m, 2H), 1.78 – 1.72 (m, 2H) 1.62 – 1.52 (m, 3H), 1.43 – 1.33 (m, 3H). ^{19}F NMR (282

MHz, $CDCl_3$) *δ* 71.39 (d, *J* = 3.7 Hz, 4F). ^{13}C NMR (126 MHz, $CDCl_3$) 173.54 (p, *J* = 35.8 Hz), 158.42, 122.89, 85.64 (p, *J* = 50.3 Hz), 80.09 – 79.87 (m), 31.26, 27.91, 25.65, 24.49. HRMS (ESI+) Calculated for $C_{12}H_{14}F_4N_2SNa$ $[M + Na]^+$ 317.0712 found 317.0707. ATR-FTIR (KBr): v = 2937, 2860, 2236, 1563,1447, 1387, 1080, 969 cm^{-1}.

2-((4,4-dimethylpent-1-yn-1-yl)tetrafluoro-λ^6-sulfaneyl)pyrimidine (**3eq**)

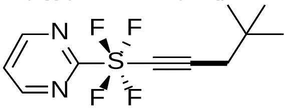

Prepared according to general procedure with **1e** (0.2 mmol) and **2q** (0.4 mmol, 2.0 equiv.) at 50 °C for 5 mints to give **3eq** (45.12 mg, 80%) as an off white solid. mp: 87.1 – 88.6 °C (5% EtOAc/hexane). ^1H NMR (500 MHz, $CDCl_3$) δ 8.87 (d, *J* = 4.8 Hz, 2H), 7.47 (t, *J* = 4.8 Hz, 1H), 2.22 – 2.19 (m, 2H) 1.05 (s, 9H). ^{19}F NMR (282 MHz, $CDCl_3$) *δ* 71.25 (t, *J* = 3.8 Hz, 4F). ^{13}C NMR (126 MHz, $CDCl_3$) 173.47 (p, *J* = 35.6 Hz), 158.43, 122.94, 86.90 (p, *J* = 50.1 Hz), 75.65 (t, *J* = 9.7 Hz), 32.47, 31.41, 29.01. HRMS (ESI+) Calculated for $C_{11}H_{14}N_2SF_4Na$ $[M + Na]^+$ 305.0712 found 305.0716. ATR-FTIR (KBr): v = 2956, 2912, 2868, 2236, 1566, 1467, 1383, 1232, 1060, 781 cm^{-1}.

2-((adamantan-1-ylethynyl)tetrafluoro-λ^6-sulfaneyl)pyrimidine (**3eu**)

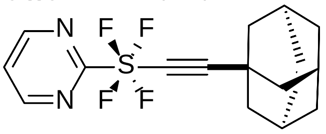

Prepared according to general procedure with **1e** (0.2 mmol) and **2u** (0.4 mmol, 2.0 equiv.) at 50 °C for 5 mints to give **3eu** (44.28 mg, 64%) as an off white solid. mp: 151.2 – 125.7 °C (5% EtOAc/hexane). ^1H NMR (500 MHz, $CDCl_3$) δ 8.86 (d, *J* = 4.8 Hz, 2H), 7.45 (t, *J* = 4.8 Hz, 1H), 1.99 – 1.95 (m, 9H) 1.71 – 1.70 (m, 6H). ^{19}F NMR (282 MHz, $CDCl_3$) *δ* 71.64 (s, 4F). ^{13}C NMR (126 MHz, $CDCl_3$) 173.60 (p, *J* = 36.0 Hz), 158.41, 122.86, 84.73 (p, *J* = 49.6 Hz), 82.94 – 82.80 (m), 41.41, 36.10, 28.96, 27.57. HRMS (ESI+) Calculated for $C_{16}H_{18}F_4N_2SNa$ $[M + Na]^+$ 369.1025 found 369.1027. ATR-FTIR (KBr): v = 2912, 2857, 2219, 1705, 1563, 1451, 1383, 1172, 1097 cm^{-1}.

2-(tetrafluoro((1-(pyrimidin-2-yl)piperidin-4-yl)ethynyl)-λ^6-sulfaneyl)pyrimidine (**3el**)

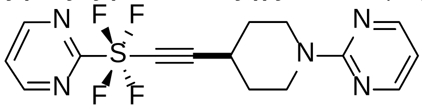

Prepared according to general procedure with **1e** (0.2 mmol) and **2l** (0.4 mmol, 2.0 equiv.) at 50 °C for 5 mints to give **3el** (59.68 mg, 80%) as an off white solid. mp: 150.5 – 151.7 °C (10% EtOAc/hexane). ^1H NMR (500 MHz, $CDCl_3$) δ 8.86 (d, *J* = 4.7 Hz, 2H), 8.29 (d, *J* = 4.7, 2H), 7.46 (t, *J* = 4.7 Hz, 1H), 6.46 (t, *J* = 4.7 Hz, 1H), 4.20 – 4.16 (m, 2H), 3.64 – 3.58 (m, 2H), 2.89 – 2.84 (m, 1H), 1.98 – 1.92 (m, 2H), 1.82 – 1.75 (m, 2H). ^{19}F NMR (282 MHz, $CDCl_3$) *δ* 71.21 (d *J* = 3.8 Hz, 4F). ^{13}C NMR (126 MHz, $CDCl_3$) 173.33 (p, *J* = 35.3 Hz), 161.50, 158.46, 157.78,122.98, 109.78, 86.63 (p, *J* = 50.3 Hz), 77.95 – 77.85 (m), 42.15, 30.11, 26.47. HRMS (ESI+) Calculated for $C_{15}H_{15}F_4N_5SNa$ $[M + Na]^+$ 396.10882 found 396.0887. ATR-FTIR (KBr): v = 3406, 2952, 2860, 2232, 1957, 1598, 1503, 1435, 1372, 1295, 1073, 785 cm^{-1}.

2-(tetrafluoro(6,6,6-trifluorohex-1-yn-1-yl)-λ^6-sulfaneyl)pyridine (**3aw**)

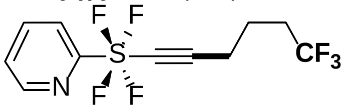

Prepared according to general procedure with **1a** (0.2 mmol) and **2w** (0.4 mmol, 2.0 equiv.) at 50 °C for 5 mints to give **3aw** (53.92 mg, 84%) as a red color thick liquid. (5% EtOAc/hexane). ^1H NMR (300 MHz, $CDCl_3$) δ 8.54 (dd, *J* = 4.7, 1.1 Hz, 1H), 7.87 (t, *J* = 7.4, 1H), 7.72 (d, J = 8.3 Hz, 1H), 7.43 (dd, *J* = 7.4, 4.7 Hz, 1H), 2.52 – 2.43 (m, 2H), 2.35 – 2.19 (m, 2H), 1.95 – 1.85 (m, 2H). ^{19}F NMR (282 MHz, $CDCl_3$) *δ* 75.63 (d, *J* = 3.9 Hz, 4F), - 67.18 (t, *J* = 10.6 Hz, 3F). ^{13}C NMR (126 MHz, $CDCl_3$) 169.35 (p, *J* = 29.1 Hz), 147.53, 138.56, 126.23, 125.97 (q, *J* = 276.6 Hz),121.27, 87.17 (p, *J* = 52.5 Hz), 73.52 (q, *J* = 10.3, 9.9 Hz), 32.60 (q, *J* = 29.1 Hz), 20.16, 16.89. HRMS (ESI+) Calculated for $C_{11}H_{10}F_7NSNa$ $[M + Na]^+$ 344.03 found 344.0321. ATR-FTIR (KBr): v = 3056, 2956, 2239, 1718, 1574, 1455, 1431, 1395, 1256, 1140, 1021 cm^{-1}.

2-(tetrafluoro(5,5,6,6,6-pentafluorohex-1-yn-1-yl)-λ^6-sulfaneyl)pyridine (**3ax**)

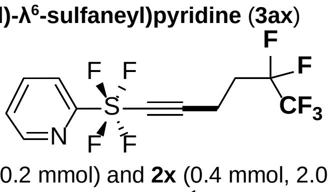

Prepared according to general procedure with **1a** (0.2 mmol) and **2x** (0.4 mmol, 2.0 equiv.) at 50 °C for 5 mints to give **3ax** (62.11 mg, 87%) as a pale-yellow solid. mp: 40.4 – 41.4 °C (5% EtOAc/hexane). ^1H NMR (300 MHz, $CDCl_3$) δ 8.55 (dd, *J* = 4.7, 1.9 Hz, 1H), 7.87 (t, *J* = 7.4, 1H), 7.72 (d, *J* = 8.3 Hz, 1H), 7.44 (dd, *J* = 7.5, 4.7 Hz, 1H), 2.73 – 2.65 (m, 2H), 2.51 – 2.34 (m, 2H). ^{19}F NMR (282 MHz, $CDCl_3$) *δ* 75.16 (t, *J* = 4.0 Hz, 4F), - 86.43 (s, 3F), - 120.23 (t, *J* = 17.3 Hz, 2F). ^{13}C NMR (126 MHz, $CDCl_3$) 169.17 (p, *J* = 28.6 Hz), 147.55, 138.57, 126.27, 122.49 – 112.10 (m),121.2, 86.80 (p, *J* = 53.0 Hz), 71.68 (p, *J* = 9.7 Hz), 29.15 (t, *J* = 22.0 Hz), 10.11. HRMS (ESI+) Calculated for $C_{11}H_8F_9NSNa$ $[M + Na]^+$ 380.0131 found 380.0130. ATR-FTIR (KBr): v = 3442, 2243, 1714, 1587, 1455, 1439, 1355, 1208, 1120, 1050, 981, 785 cm^{-1}.

2-fluoro-3-(tetrafluoro(5,5,6,6,6-pentafluorohex-1-yn-1-yl)-l6-sulfaneyl)pyridine (**3cx**)

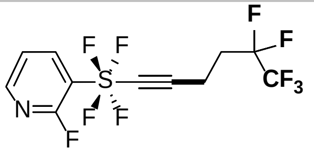

Prepared according to general procedure with **1c** (0.2 mmol) and **2x** (0.4 mmol, 2.0 equiv.) at 50 °C for 5 mints to give **3cx** (52.72 mg, 70%) as a red color gummy compound. (5% EtOAc/hexane). ^{1}H NMR (500 MHz, $CDCl_3$) δ 8.32 (d, *J* = 4.7 Hz, 1H), 8.16 (td, *J* = 8.2, 1.8 Hz, 1H), 7.29 (dd, *J* = 8.2, 4.3 Hz, 1H), 2.70 – 2.66 (m, 2H), 2.46 – 2.36 (m, 2H). ^{19}F NMR (282 MHz, $CDCl_3$) *δ* 90.34 (dt, *J* = 21.9, 3.6 Hz, 4F), -60.73 (td, *J* = 20.0, 8.4 Hz, 1F) - 86.42 (s, 3F), - 120.21 (t, *J* = 17.3 Hz, 2F). ^{13}C NMR (126 MHz, $CDCl_3$) 155.20 (t, *J* = 246.1 Hz), 149. 89 (d, *J* = 15.1 Hz), 140.08 (dd, *J* = 55.9, 28.7 Hz), 139.56, 121.50, 122.51 – 112.11 (m), 87.01 (p, *J* = 52.2 Hz), 72.12 (m), 29.14 (t, *J* = 22.0 Hz), 10.08. HRMS (ESI+) Calculated for $C_{11}H_8F_{10}NS$ [M + H]$^+$ 376.0218 found 376.0214. ATR-FTIR (KBr): ν = 3083, 2250, 1589, 1450, 1201, 973, 800, 754 cm^{-1}.

2-(tetrafluoro(5,5,6,6,6-pentafluorohex-1-yn-1-yl)-λ^6-sulfaneyl)pyrimidine (**3ex**)

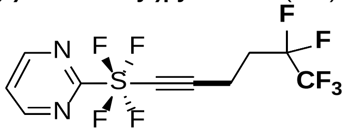

Prepared according to general procedure with **1e** (0.2 mmol) and **2x** (0.4 mmol, 2.0 equiv.) at 50 °C for 5 mints to give **3ex** (45.10 mg, 63%) as a red color solid. mp: 47.3 – 48.8 °C (5% EtOAc/hexane). ^{1}H NMR (300 MHz, $CDCl_3$) δ 8.89 (d, *J* = 4.7Hz, 2H), 7.49 (t, *J* = 4.7 Hz, 1H), 2.74 – 2.66 (m, 2H), 2.52 – 2.35 (m, 2H). ^{19}F NMR (282 MHz, $CDCl_3$) *δ* 70.35 (t, *J* = 3.6 Hz, 4F), - 86.40 (s, 3F), - 120.21 (t, *J* = 17.3 Hz, 2F). ^{13}C NMR (176 MHz, $CDCl_3$) 173.12 (t, *J* = 34.6 Hz), 158.50, 123.07, 119.85 – 113.04 (m), 86.12 (t, *J* = 51.7 Hz), 72.57 (t, *J* = 9.3 Hz), 29.24 (t, *J* = 22.1 Hz), 10.19. HRMS (ESI+) Calculated for $C_{10}H_7F_9N_2NaS$ [M + Na]$^+$ 381.0084 found 381.0083. ATR-FTIR (KBr): ν = 3092, 3054, 2965, 2251, 1563, 1443, 1387, 1192, 1116, 985 cm^{-1}.

2-(tetrafluoro(5,5,6,6,7,7,8,8,8-nonafluorooct-1-yn-1-yl)-λ^6-sulfaneyl)pyridine (**3ay**)

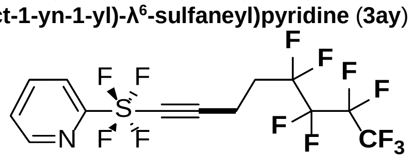

Prepared according to general procedure with **1a** (0.2 mmol) and **2y** (0.4 mmol, 2.0 equiv.) at 50 °C for 5 mints to give **3ay** (71.29 mg, 78%) as a pale-yellow solid. mp: 32.1 – 33.2 °C (5% EtOAc/hexane). ^{1}H NMR (300 MHz, $CDCl_3$) δ 8.54 (dd, *J* = 4.7, 2.0 Hz, 1H), 7.87 (t, *J* = 8.2 Hz, 1H), 7.72 (d, *J* = 8.2 Hz, 1H), 7.44 (dd, *J* = 7.4, 4.7 Hz, 1H) 2.75 – 2.65 (m, 2H), 2.55 – 2.38 (m, 2H). ^{19}F NMR (282 MHz, $CDCl_3$) *δ* 75.22 (t, *J* = 4.0 Hz, 4F), - 82.02 (t, *J* = 9.8 Hz, 3F), – 116.53 (p, *J* = 13.6 Hz, 2F), - 125.44 (td, *J* = 9.4, 5.1 Hz, 2F) -127.01 (p, *J* = 9.1, 8.3 Hz, 2F). ^{13}C NMR (126 MHz, $CDCl_3$) 169.19 (p, *J* = 28.9 Hz), 147.56, 138.58, 126.28, 121.28, 120.77 – 106.10 (m), 86.85 (p, *J* = 52.9 Hz), 72.27 – 71.15 (m), 29.35 (t, *J* = 22.1 Hz), 10.02. HRMS (ESI+) Calculated for $C_{13}H_8F_{13}NSNa$ [M + Na]$^+$ 480.0068 found 480.0068. ATR-FTIR (KBr): ν = 3064, 2251, 1587, 1455, 1439, 1228, 1132, 774 cm^{-1}.

2-(tetrafluoro(5,5,6,6,7,7,8,8,9,9,10,10,10-tridecafluorodec-1-yn-1-yl)-λ^6-sulfaneyl)pyridine (**3az**)

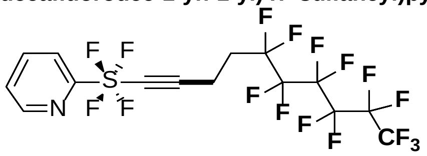

Prepared according to general procedure with **1a** (0.2 mmol) and **2z** (0.4 mmol, 2.0 equiv.) at 50 °C for 5 mints to give **3az** (80.20 mg, 72%) as a red color solid. mp: 55.1 – 56.1 °C (5% EtOAc/hexane). ^{1}H NMR (500 MHz, $CDCl_3$) δ 8.47 (d, *J* = 4.6 Hz, 1H), 7.80 (t, *J* = 7.9 Hz, 1H), 7.65 (d, *J* = 8.3 Hz, 1H), 7.36 (dd, *J* = 7.4, 4.8 Hz, 1H) 2.65 – 2.60 (m, 2H), 2.45 – 2.34 (m, 2H). ^{19}F NMR (282 MHz, $CDCl_3$) *δ* 75.22 (t, *J* = 3.9 Hz, 4F), - 81.77 (t, *J* = 9.8 Hz, 3F), – 116.48 (m, 2F), - 122.81 - - 122.90 (m, 2F) -123.84 (s, 2F), - 124.43 – - 124.53 (m, 2F), -127.13 (td, *J* = 14.8, 6.7 Hz, 2F). ^{13}C NMR (126 MHz, $CDCl_3$) 169.19 (p, *J* = 28.9 Hz), 147.57, 138.56, 126.27, 121.28, 120.63 – 105.84 (m), 86.85 (p, *J* = 53.1 Hz), 72.81 – 70.58 (m), 29.45 (t, *J* = 22.2 Hz), 10.06. HRMS (ESI+) Calculated for $C_{15}H_9F_{17}NS$ [M + H]$^+$ 558.0184 found 558.0187. ATR-FTIR (KBr): ν = 3056, 2255, 1587, 1458, 1439, 1240, 1196, 1144, 778 cm^{-1}.

2-(tetrafluoro(5,5,6,6,7,7,8,8,9,9,10,10,11,11,12,12,13,13,14,14,14-henicosafluorotetradec-1-yn-1-yl)-λ^6-sulfaneyl)pyridine (3aaa)

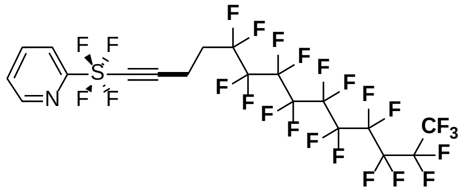

Prepared according to general procedure with **1a** (0.2 mmol) and **2aa** (0.4 mmol, 2.0 equiv.) at 50 °C for 5 mints to give **3aaa** (48.44 mg, 32%) as a off white solid. mp: 105.3 – 106.8 °C (5% EtOAc/hexane). ^{1}H NMR (500 MHz, $CDCl_3$) δ 8.55 (d, *J* = 5.0, 1.7 Hz, 1H), 7.88 (t, *J* = 7.9 Hz, 1H), 7.72 (d, *J* = 8.2 Hz, 1H), 7.44 (dd, *J* = 7.4, 4.7 Hz, 1H), 2.72 – 2.67 (m, 2H), 2.52 – 2.42 (m, 2H). ^{19}F NMR (282 MHz, $CDCl_3$) *δ* 75.08 (t, *J* = 3.8 Hz, 4F), - 81.84 (t, *J* = 9.9 Hz, 3F), – 116.34 (s, 2F), - 122.85 (s, 10F), -123.80 (s, 2F), - 124.55 (s, 2F), -127.20 (s, 2F). ^{13}C NMR (126 MHz, $CDCl_3$) 169.20 (p, *J* = 28.8 Hz), 147.58, 138.58, 126.28, 121.30, 120.81 – 105.77 (m), 86.86 (p, *J* = 53.1 Hz), 71.71 (m), 29.47 (t, *J* = 22.0 Hz), 10.07. HRMS (ESI+) Calculated for $C_{19}H_9F_{25}NS$ [M + H]$^+$ 758.0057 found 758.0052. ATR-FTIR (KBr): ν = 3064, 2916, 2260, 1742, 1587, 1463, 1344, 1224, 1080 cm^{-1}.

(3S,8R,9S,10S,13S,14S)-10,13-dimethyl-3-((tetrafluoro(pyridin-2-yl)-λ^6-sulfaneyl)ethynyl)hexadecahydro-17*H*-cyclopenta[a]phenanthren-17-one (dr ratio = 1:2) (3aab)

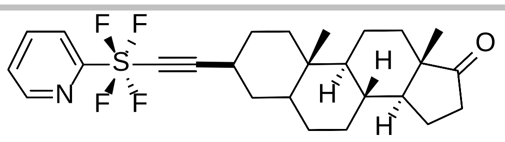

Prepared according to general procedure with **1a** (0.1 mmol) and **2ab** (0.2 mmol, 2.0 equiv.) at 50 °C for 5 mints to give **3aab** (42.01 mg, 87%) as a pale-yellow solidmp: 158.1 – 159.1 °C (5% EtOAc/hexane). ^{1}H NMR (500 MHz, $CDCl_3$) δ 8.55 – 8.53 (m, 1H), 7.89 - 8.53 (m, 1H), 7.72 – 7.71 (m, 1H), 7.44 – 7.41 (m, 1H), 2.95 – 2.92 (m, 1H), 2.47 – 2.41 (m, 1H), 2.12 – 2.03 (m, 1H), 1.97 – 1.91 (m, 1H), 1.82 – 1.23 (m, 18H), 1.15 – 0.94 (m, 2H), 0.86 (s, 6H, Major), 0.82 (s, 6H, Minor). ^{19}F NMR (282 MHz, $CDCl_3$) *δ* 76.21 (d, *J* = 3.7 Hz, 4F, major), 76.06 (d, *J* = 3.4 Hz, 4F, minor). ^{13}C NMR (126 MHz, $CDCl_3$) 169.68 (p, *J* = 30.3, 29.7 Hz), 147.43, 138.46, 126.05, 121.23, 87.90 – 85.44 (m), 79.23 (s, major), 78.77 (s, minor), 54.28, 54.14, 51.37, 51.29, 47.82, 46.11, 41.77, 37.67, 36.22, 35.90, 35.84, 35.71, 34.95, 34.10, 33.75, 32.04, 31.44, 30.74, 30.59, 28.73, 28.10, 28.05, 27.34, 26.38, 25.77, 21.74, 20.14, 20.05, 13.82, 12.23, 11.85. HRMS (ESI+) Calculated for $C_{26}H_{34}F_4NOS$ $[M + H]^+$ 484.2297 found 484.2303. ATR-FTIR (KBr): v = 3056, 2933, 2864, 2232, 1729, 1582, 1455, 1005, 778 cm^{-1}.

2-(tetrafluoro(((4S,5'R,6aR,6bS,8aS,8bR,9S,10R,11aS,12aS,12bS)-5',6a,8a,9-tetramethyl-1,3,3',4,4',5,5',6,6a,6b,6',7,8, 8a,8b,9,11a,12,12a,12b-icosahydrospiro[naphtho[2',1':4,5]indeno[2,1-b]furan-10,2'-pyran]-4-yl)ethynyl)-λ^6-sulfaneyl)pyridine (dr ratio = 1: 1.82) (**3aac**)

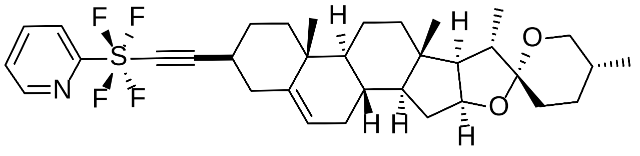

Prepared according to general procedure with **1a** (0.1 mmol) and **2ac** (0.2 mmol, 2.0 equiv.) at 50 °C for 5 mints to give **3aac** (40.06 mg, 66%) as an off white solid. mp: 202.7 – 203.5 °C (5% EtOAc/hexane). ^{1}H NMR (500 MHz, $CDCl_3$) δ 8.54 (dd, *J* = 4.7, 1.4 Hz, 1H), 7.86 (t, *J* = 7.9 Hz, 1H), 7.72 (d, *J* = 8.2 Hz, 1H), 7.42 (dd, *J* = 6.9, 5.3 Hz, 1H), 5.44 (d, *J* = 5.3 Hz, 1H, major), 5.37 (d, *J* = 5.3 Hz, 1H, minor), 4.44 – 4.38 (m, 1H), 3.49 – 3.46 (m, 1H), 3.38 (t, *J* = 10.9 Hz, 1H), 2.94 – 2.92 (m, 1H), 2.56 – 2.32 (m, 2H) 2.18 – 2.15 (m, 3H), 2.04 – 1.85 (m, 4H), 1.80 – 1.41 (m, 15H), 1.32 – 1.08 (m, 4H), 1.03 (d, *J* = 11.9 Hz, 3H), 0.98 (d, *J* = 7.0 Hz, 3H), 0.80 (s, 3H, minor), 0.79 (s, 3H, major). ^{19}F NMR (282 MHz, $CDCl_3$) *δ* 76.22 (d, *J* = 3.3 Hz, 4F, major), 76.03 (s, 4F, minor). ^{13}C NMR (126 MHz, $CDCl_3$) 169.76 (t, *J* = 29.7 Hz), 147.44, 140.39, 138.45, 138.20, 126.03, 122.84, 121.32, 109.33, 88.10 (p, *J* = 51.0 Hz), 86.76 – 85.75 (m), 80.90, 78.43, 78.09, 66.86, 62.02, 56.49, 56.26, 50.10, 50.04, 41.61, 40.23, 39.71, 38.61, 37.50, 37.33, 36.89, 36.58, 35.39, 31.99, 31.84, 31.38, 31.32, 31.26, 31.02, 30.31, 29.93, 28.80, 28.03, 27.93, 26.24, 20.59, 19.36, 19.25, 17.19, 16.33, 14.59. HRMS (ESI+) Calculated for $C_{34}H_{45}F_4NO_2SNa$ for $[M + Na]^+$ 630.3005 found 630.3003. ATR-FTIR (KBr): v = 2937, 2905, 2868, 2343, 2228, 1574, 1451, 1419, 1367, 1050, 778 cm^{-1}.

2-((((3S,8S,9S,10R,13R,14S,17R)-17-((2R,5S,E)-5-ethyl-6-methylhept-3-en-2-yl)-10,13-dimethyl-2,3,4,7, 8,9,10,11,12,13,14,15,16,17-tetradecahydro-1*H*-cyclopenta[a]phenanthren-3-yl)ethynyl)tetrafluoro-λ^6-sulfaneyl)pyridine (3aad) (minor)

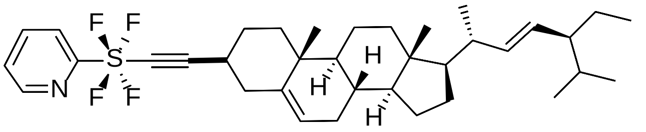

Prepared according to general procedure with 1a (0.1 mmol) and 2ad (0.2 mmol, 2.0 equiv.) at 50 °C for 5 mints to give 3aad (12.0 mg, 20%) as an off white solid. 5% EtOAc/hexane). ^{1}H NMR (500 MHz, $CDCl_3$) δ 8.54 (dd, *J* = 5.1, 2.4 Hz, 1H), 7.86 (t, *J* = 7.9 Hz, 1H), 7.72 (d, *J* = 8.2 Hz, 1H), 7.42 (dd, *J* = 7.0, 5.2 Hz, 1H), 5.38 – 5.35 (m, 1H), 5.15 (dd, *J* = 15.1, 8.7 Hz, 1H), 5.01 (dd, *J* = 15.1, 8.7 Hz, 1H), 2.43 – 2.31 (m, 3H), 2.06 – 1.87 (m, 5H), 1.75 – 1.67 (m, 2H), 1.56 – 1.40 (m, 8H), 1.28 – 1.06 (m, 7H) 1.03 – 1.02 (m, 6H), 1.00 – 0.90 (m, 1H), 0.85 – 0.79 (m, 9H), 0.70 (s, 3H). ^{19}F NMR (282 MHz, $CDCl_3$) *δ* 76.04 (s, 4F). ^{13}C NMR (126 MHz, $CDCl_3$) 169.71 (t, *J* = 29.0 Hz), 147.46, 140.37, 138.46, 138.38, 129.25, 126.05, 121.62, 121.33, 86.18 (t, *J* = 51.66 Hz), 78.59, 56.86, 55.91, 51.26, 50.17, 42.19, 40.59, 39.63, 38.65, 37.54, 36.78, 31.93, 31.83, 31.73, 29.96, 28.99, 27.97, 25.47, 24.37, 21.26, 21.17, 20.82, 19.36, 19.01, 12.33, 12.07. HRMS (ESI+) Calculated for $C_{36}H_{51}F_4SNa$ $[M + Na]^+$628.3576 found 628.3574. ATR-FTIR (KBr): v = 2952, 2858, 2237, 1577, 1460, 1427, 782, 677 cm^{-1}.

2-((((3S,8S,9S,10R,13R,14S,17R)-10,13-dimethyl-17-((R)-6-methylheptan-2-yl)-2,3,4,7,8,9,10,11,12,13,14,15,16,17-tetradecahydro-1H-cyclopenta[a]phenanthren-3-yl)ethynyl)tetrafluoro-λ^6-sulfaneyl)pyridine major isomer (**3aae**)

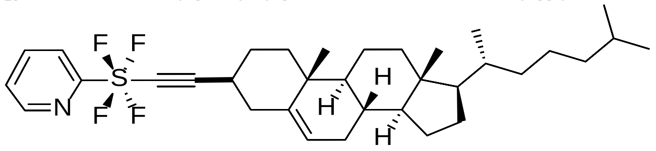

Prepared according to general procedure with **1a** (0.1 mmol) and **2ae** (0.2 mmol, 2.0 equiv.) at 50 °C for 5 mints to give **3aae** (18.0 mg, 31%) as a off white solid. mp: 190.3 – 191.7 °C (5% EtOAc/hexane). ^{1}H NMR (500 MHz, $CDCl_3$) δ 8.53 (ddd, *J* = 4.7, 1.9, 0.8 Hz, 1H), 7.85 (t, *J* = 7.9 Hz, 1H), 7.72 (d, *J* = 8.3 Hz, 1H), 7.41 (ddd, *J* = 7.4, 4.7, 1.0 Hz, 1H), 5.45 – 5.44 (m,1H), 2.92 (s, 1H), 2.16 – 2.14 (m, 1H), 2.02 – 1.94 (m, 2H), 1.83 – 1.78 (m, 3H), 1.74 – 1.72 (m, 1H), 1.65 – 1.61 (m, 1H) 1.58 – 1.50 (m, 4H), 1.47 – 1.40 (m, 2H), 1.37 – 1.32 (m, 3H), 1.25 – 1.05 (m, 10H), 1.00 (s, 3H), 0.92 (d, *J* = 6.6 Hz, 3H), 0.86 (dd, *J* = 6.6, 3.1 Hz, 6H), 0.68 (s, 3H). ^{19}F NMR (282 MHz, $CDCl_3$) *δ* 76.24 (s, 4F). ^{13}C NMR (126 MHz, $CDCl_3$) 169.76 (t, *J* = 29.92 Hz), 147.50, 138.44, 138.23, 126.01, 123.21, 121.39, 88.18 (t, *J* = 49.28 Hz), 78.33 (t, *J* = 8.8 Hz), 56.65, 56.24, 50.31, 42.36, 39.79, 39.60, 37.26, 36.68, 36.28, 35.92, 35.49, 31.93, 31.88, 28.34, 28.15, 28.09, 26.34, 24.36, 23.95, 22.90, 22.65, 20.88, 19.29, 18.79, 11.93. HRMS (ESI+) Calculated for $[M + H]^+$ $C_{34}H_{50}F_4NS$ 580.3600 found 580.3601. ATR-FTIR (KBr): v = 3477, 2944, 2873, 2232, 1582, 1455, 1439, 1375, 1037, 785, 770, 654 cm^{-1}.

2-(tetrafluoro(((1S,2R,4R)-2-isopropyl-4-methylcyclohexyl)ethynyl)-λ^6-sulfaneyl)pyridine (dr ratio = 1: 4.34) (**3aaf**)

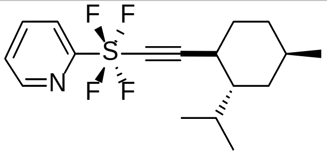

Prepared according to general procedure with **1a** (0.1 mmol) and **2af** (0.2 mmol, 2.0 equiv.) at 50 ºC for 5 mints to give **3aaf** (24.43 mg, 70%) as a red color solid. mp: 57.0 – 57.8 °C (5% EtOAc/hexane). ^{1}H NMR (300 MHz, $CDCl_3$) δ 8.53 (dd, *J* = 4.4, 1.7 Hz, 1H), 7.85 (t, *J* = 7.8 Hz, 1H), 7.71 (d, *J* = 8.2 Hz, 1H), 7.41 (dd, *J* = 7.4, 4.7 Hz, 1H), 2.38 – 2.17 (m, 2H), 2.08 – 1.90 (m, 1H), 1.78 – 1.64 (m, 2H), 1.38 – 1.14 (m, 3H) 0.97 – 0.89 (m, 8H), 0.83 (d, *J* = 7.0 Hz, 3H), ^{19}F NMR (282 MHz, $CDCl_3$) *δ* 76.27 (d, *J* = 3.7 Hz, 4F, minor), 76.13 (d, J = 3.5 Hz, 4F, major). ^{13}C NMR (126 MHz, $CDCl_3$) 169.79 (p, *J* = 29.8 Hz), 147.44, 138.44, 126.02, 121.27, 89.27 – 88.25 (m, minor), 87.22 (p, *J* = 50.7 Hz), 78.58 (t, *J* = 9.8 Hz), 46.95, 46.53, 40.79, 39.15, 35.07, 34.54, 32.36, 30.91, 29.88, 28.93, 27.93, 26.56, 25.60, 24.35, 22.19, 21.15, 20.99, 20.72, 15.91. HRMS (ESI+) Calculated for [M + Na]$^+$ $C_{17}H_{23}F_4NSNa$ for [M + Na]$^+$ 372.1385 found 372.1392. ATR-FTIR (KBr): v = 3056, 2956, 2928, 2888, 2232, 1574, 1447, 1423, 1379, 1101, 790 cm^{-1}.

***t*ert-butyl 3-((tetrafluoro(pyridin-2-yl)-λ^6-sulfaneyl)ethynyl)-8-azabicyclo[3.2.1]octane-8-carboxylate** (**3aag**)

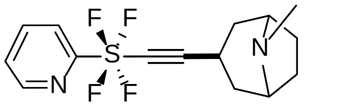

Prepared according to general procedure with **1a** (0.1 mmol) and **2ag** (0.2 mmol, 2.0 equiv.) at 50 ºC for 5 mints to give **3aag** (36.54 mg, 87%) as an off white solid. mp: 130.1 – 131.0 °C (10% EtOAc/hexane). ^{1}H NMR (300 MHz, $CDCl_3$) δ 8.52 (dd, *J* = 4.4, 1.8 Hz, 1H), 7.85 (t, *J* = 7.9 Hz, 1H), 7.70 (d, *J* = 8.4 Hz, 1H), 7.41 (dd, *J* = 7.4, 3.8 Hz, 1H), 4.27 – 4.21 (m, 2H), 2.98 – 2.97 (m, 1H), 2.04 – 1.83 (m, 7H), 1.64 (s, 1H), 1.48 (s, 9H), ^{19}F NMR (282 MHz, $CDCl_3$) *δ* 75.83 (d, *J* = 3.3 Hz, 4F), ^{13}C NMR (126 MHz, $CDCl_3$) 169.45 (p, *J* = 29.4 Hz), 153.10, 147.43, 138.48, 126.10, 121.23, 86.15 (p, *J* = 51.8 Hz), 79.55, 53.10, 52.32, 36.23, 35.49, 28.47, 28.08, 27.40, 20.45. HRMS (ESI+) Calculated for $C_{19}H_{24}F_4N_2O_2SNa$ [M + Na]$^+$ 443.1392 found 443.1395. ATR-FTIR (KBr): v = 3051, 2972, 2896, 2232, 1686, 1574, 1407, 1168, 1112 cm^{-1}.

1,3-dimethyl-7-(2-methyl-4-(tetrafluoro(pyridin-2-yl)-λ^6-sulfaneyl)but-3-yn-1-yl)-3,7-dihydro-1*H*-purine-2,6-dione (**3aah**)

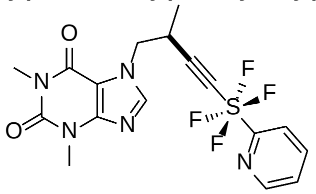

Prepared according to general procedure with **1a** (0.1 mmol) and **2ah** (0.2 mmol, 2.0 equiv.) at 50 ºC for 5 mints to give **3aah** (34.91 mg, 81%) as an off white solid. mp: 183.3 – 184.9 °C (10% EtOAc/hexane). ^{1}H NMR (700 MHz, $CDCl_3$) δ 8.53 (dd, *J* = 4.3, 1.6 Hz, 1H), 7.87 (t, *J* = 7.8 Hz, 1H), 7.74 (s, 1H), 7.69 (d, *J* = 8.3 Hz, 1H), 7.44 (dd, *J* = 7.5, 4.7 Hz, 1H), 4.56 (dd, *J* = 13.5, 5.0 Hz, 1H), 4.23 (dd, *J* = 13.5, 9.4 Hz, 1H), 3.61 (s, 3H), 3.42 (s, 3H), 3.32 – 3.28 (m, 1H), 1.38 (d, *J* = 7.0 Hz, 3H), ^{19}F NMR (282 MHz, $CDCl_3$) *δ* 75.61 (d, *J* = 3.3 Hz, 4F), ^{13}C NMR (176 MHz, $CDCl_3$) 169.10 (t, *J* = 28.9 Hz), 151.68, 149.12, 147.55, 141.80, 138.54, 126.27, 121.22, 106.69, 88.64 (t, *J* = 52.7 Hz), 74.94 (t, *J* = 9.2 Hz), 50.97, 29.88, 27.99, 27.57, 16.84. HRMS (ESI+) Calculated for $C_{17}H_{17}F_4N_5O_2SNa$ [M +Na]$^+$ 454.0937 found 454.0937. ATR-FTIR (KBr): v = 3127, 3079, 2948, 2223, 1701, 1646, 1559, 1463, 1236, 1116, 1024, 965 cm^{-1}.

2-(tetrafluoro(5-(2-iodophenyl)pent-1-yn-1-yl)-λ^6-sulfaneyl)pyridine (**3aai**)

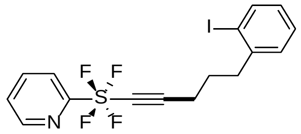

Prepared according to general procedure with **1a** (0.1 mmol) and **2ai** (0.2 mmol, 2.0 equiv.) at 50 ºC for 5 mints to give **3a** (28.14 mg, 62%) as a brick red colored liqued. (10% EtOAc/hexane). ^{1}H NMR (500 MHz, $CDCl_3$) δ 8.54 (dd, *J* = 4.7, 1.1 Hz, 1H), 7.86 (t, *J* = 7.9 Hz, 1H), 7.82 (dd, *J* = 7.9, 1.3 Hz, 1H), 7.73 (d, *J* = 8.2 Hz, 1H), 7.42 (dd, *J* = 7.5, 4.7 Hz, 1H), 7.30 – 7.25 (m, 2H), 6.90 (ddd, *J* = 7.8, 6.9, 2.1 Hz, 1H), 2.92 – 2.86 (m, 2H), 2.40 (tt, *J* = 7.0, 3.6 Hz, 2H), 1.96 – 1.87 (m, 2H).^{19}F NMR (282 MHz, $CDCl_3$) *δ* 75.84 (s, 4F). ^{13}C NMR (126 MHz, $CDCl_3$) 169.60 (p, *J* = 29.5 Hz), 147.46, 143.54, 139.62, 138.45, 129.73,128.43, 128.05, 126.09, 121.28 (*t*, J = 4.5 Hz), 86.82 (p, *J* = 51.5 Hz), 75.59 – 74.29 (m), 39.50, 27.65, 17.12. HRMS (ESI+) Calculated for $C_{16}H_{14}F_4NINaS$ [M +Na]+ 477.9725 found 477.9746. ATR-FTIR (KBr): v = 3057, 2934, 2863, 2237, 1580, 1460, 1427, 1009, 664 cm^{-1}.

(1R,3S,5s,7s)-5-((tetrafluoro(pyridin-2-yl)-λ^6-sulfaneyl)ethynyl)adamantan-2-one (**3aaj**)

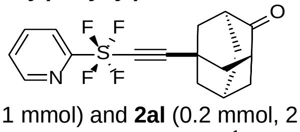

Prepared according to general procedure with **1a** (0.1 mmol) and **2al** (0.2 mmol, 2.0 equiv.) at 50 ºC for 5 mints to give **3aal** (29.0 mg, 80%) as a brick red colored solid. mp: 153.2 – 154.0 °C (% EtOAc/hexane). ^{1}H NMR (500 MHz, $CDCl_3$) δ 8.53 (dd, *J* = 4.7, 1.8 Hz, 1H), 7.86 (t, *J* = 7.5 Hz, 1H), 7.70 (d, *J* = 8.3 Hz, 1H), 7.43 (dd, *J* = 7.4, 4.7 Hz, 1H), 2.61 (s, 2H), 2.27 (s, 4H), 2.20 (s, 3H), 2.07 (d, *J* = 10.7 Hz, 2H), 1.99 (d, *J* = 12.1 Hz, 2H). ^{19}F NMR (282 MHz, $CDCl_3$) *δ* 75.98 (s, 4F). ^{13}C NMR (126 MHz, $CDCl_3$) 215.66, 169.45 (p, *J* = 29.2 Hz), 147.51, 138.48, 126.13, 121.25, 86.46 (p, *J* = 51.9 Hz), 79.08 – 78.58 (m),45.73, 42.62, 40.50, 38.0, 28.82, 27.0. LCMS (ESI+) Calculated for $C_{17}H_{18}F_4NOS$ [M + H]$^+$. ATR-FTIR (KBr): v = 3052, 2930, 2859, 2223, 1720, 1577, 1456, 1424, 1070, 952, 660 cm^{-1}.

phenyl(4-((tetrafluoro(pyridin-2-yl)-λ^6-sulfaneyl)ethynyl)piperidin-1-yl)methanone (**3aak**)

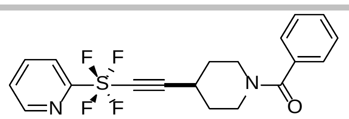

Prepared according to general procedure with **1a** (0.1 mmol) and **2** (0.2 mmol, 2.0 equiv.) at 50 °C for 5 mints to give **3aak** (34.6 mg, 87%) as a brick red colored liqued. (30% EtOAc/hexane). ^{1}H NMR (500 MHz, $CDCl_3$) δ 8.54 (dd, *J* = 4.7, 1.2 Hz, 1H), 7.87 (t, *J* = 7.9 Hz, 1H), 7.72 (d, *J* = 8.2 Hz, 1H), 7.49 – 7.39 (m, 6H), 4.02 (s, 1H), 3.66 (s, 2H), 3.34 (s, 1H), 2.92 – 2.88 (m, 1H), 2.0 (s, 1H), 1.89 – 1.83 (m, 2H), 1.73 (s, 1H). ^{19}F NMR (282 MHz, $CDCl_3$) *δ* 75.93 (d, J = 3.4 Hz, 4F). ^{13}C NMR (126 MHz, $CDCl_3$) 169.93 – 169.29 (m), 147.45, 138.44, 137.21, 131.86, 128.54, 127.40, 127.29, 126.18, 126.07, 121.31 – 121.25 (m), 86.99 – 86.40 (m), 74.92 – 74.81 (m), 30.86, 18.06. HRMS (ESI+) Calculated for $C_{19}H_{18}F_4N_2ONaS$ $[M +Na]^+$ 421.0974 found 421.0979. ATR-FTIR (KBr): v = 3477, 3059, 2928, 2861, 2235, 1742, 1578, 1495, 1276, 649 cm^{-1}.

5-(tetrafluoro(pyridin-2-yl)-λ^6-sulfaneyl)pent-4-yn-1-ol (**3aal**)

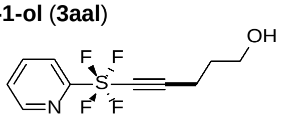

Prepared according to general procedure with **1a** (0.1 mmol) and **2al** (0.2 mmol, 2.0 equiv.) at 50 °C for 5 mints to give **3aal** (17.5 mg, 65%) as a brick red colored liqued. (20% EtOAc/hexane). ^{1}H NMR (500 MHz, $CDCl_3$) δ 8.54 (dd, *J* = 4.7, 1.1 Hz, 1H), 7.86 (t, *J* = 7.9 Hz, 1H), 7.72 (d, *J* = 8.2 Hz, 1H), 7.43 (dd, *J* = 6.9, 5.1 Hz, 1H), 3.81 (t, *J* = 6.1 Hz, 2H), 2.50 (tt, *J* = 7.1, 3.6 Hz, 2H), 1.88 (tt, *J* = 7.0, 6.1 Hz, 2H). ^{19}F NMR (282 MHz, $CDCl_3$) *δ* 75.76 (s, 4F). ^{13}C NMR (126 MHz, $CDCl_3$) 169.55 (p, *J* = 29.5 Hz), 147.47, 138.47, 126.09, 121.28, 86.42 (p, *J* = 51.9 Hz), 75.69 – 73.79 (m), 61.13, 30.10, 14.22. MS (ESI+) $C_{10}H_{12}F_4N0S$ $[M + H]^+$ 270.05. ATR-FTIR (KBr): v = 3398, 2931, 2883, 2237, 1582, 1461, 1428, 1072, 994, 665 cm^{-1}.

(E)-2-(tetrafluoro(6-phenylhex-5-en-1-yn-1-yl)-λ^6-sulfaneyl)pyridine (**3aam**)

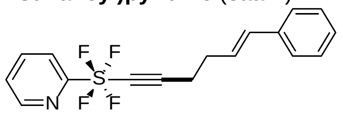

Prepared according to general procedure with **1a** (0.1 mmol) and **2am** (0.2 mmol, 2.0 equiv.) at 50 °C for 5 mints to give **3aam** (27.28 mg, 80%) as a off white solid. mp: 67.6 – 68.8 °C (10% EtOAc/hexane). ^{1}H NMR (700 MHz, $CDCl_3$) δ 8.53 (dd, *J* = 4.6, 2.2 Hz, 1H), 7.84 (t, *J* = 7.8 Hz, 1H), 7.71 (d, *J* = 8.4 Hz, 1H), 7.41 (dd, *J* = 7.4, 4.6 Hz, 1H), 7.39 – 7.35 (m, 2H), 7.30 (t, *J* = 7.8 Hz, 2H), 7.24 – 7.19 (m, 1H), 6.50 (d, *J* = 15.8 Hz, 1H), 6.26 (dt, *J* = 15.6, 6.6 Hz, 1H), 2.52 (ddd, *J* = 9.9, 5.3, 2.5 Hz, 4H).^{19}F NMR (282 MHz, $CDCl_3$) *δ* 75.73 (s, 4F). ^{13}C NMR (126 MHz, $CDCl_3$) 169.64 (p, *J* = 29.5 Hz), 147.47, 140.89,138.45, 128.58, 128.48, 126.12, 126.07, 121.29 (*t*, *J* = 4.6 Hz), 86.69 (p, *J* = 51.5 Hz), 75.31 (p, *J* = 9.7 Hz), 34.51, 28.95, 16.95. HRMS (ESI+) Calculated for $C_{17}H_{15}F_4NNaS$ $[M +Na]^+$ 364.0759 found 364.0744. ATR-FTIR (KBr): v = 3061, 3028, 2921, 2238, 1596, 1578, 1495, 1459, 1443, 1424, 1247, 1057, 991, 963, 741, 671, 621 cm^{-1}.

2-(tetrafluoro(5-phenylpent-1-yn-1-yl)-λ^6-sulfaneyl)pyridine (**3aan**)

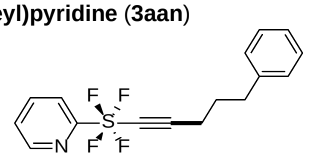

Prepared according to general procedure with **1a** (0.1 mmol) and **2** (0.2 mmol, 2.0 equiv.) at 50 °C for 5 mints to give **3an** (25.6 mg, 78%) as a brick red colored liqued. (10% EtOAc/hexane). ^{1}H NMR (500 MHz, $CDCl_3$) δ 8.54 (dd, *J* = 4.7, 1.2 Hz, 1H), 7.89 – 7.84 (m, 1H), 7.73 (d, *J* = 8.2 Hz, 1H), 7.42 (dd, *J* = 7.4, 4.7 Hz, 1H), 7.33 – 7.28 (m, 2H), 7.24 – 7.17 (m, 3H), 2.78 (t, *J* = 7.5 Hz, 2H), 2.34 (tt, *J* = 7.1, 3.6 Hz, 2H), 1.94 (p, *J* = 7.1 Hz, 2H).^{19}F NMR (282 MHz, $CDCl_3$) *δ* 75.84 (s, 4F). ^{13}C NMR (126 MHz, $CDCl_3$) 169.64 (p, *J* = 29.5 Hz), 147.47, 140.89,138.45, 128.58, 128.48, 126.12, 126.07, 121.29 (*t*, *J* = 4.6 Hz), 86.69 (p, *J* = 51.5 Hz), 75.31 (p, *J* = 9.7 Hz), 34.51, 28.95, 16.95. HRMS (ESI+) Calculated for $C_{16}H_{15}F_4NNaS$ $[M +Na]^+$ 352.0759 found 352.0758. ATR-FTIR (KBr): v = 3061, 3027, 2943, 2862, 2237, 1580, 1495, 1458, 1427, 664 cm^{-1}.

2-(tetrafluoro((3,4,5-trimethoxyphenyl)ethynyl)λl^6-sulfaneyl)pyridine

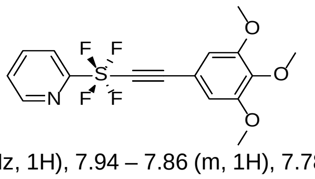

^{1}H NMR (300 MHz, $CDCl_3$) δ 8.58 (dd, *J* = 4.3, 1.6 Hz, 1H), 7.94 – 7.86 (m, 1H), 7.78 (d, *J* = 8.4 Hz, 1H), 7.46 (dd, *J* = 7.4, 4.8 Hz, 1H), 6.84 (s, 2H), 3.89 (s, 9H).^{19}F NMR (282 MHz, $CDCl_3$) *δ* 76.06 (s, 4F). MS (ESI+) Calculated for $C_{16}H_{16}F_4NO_3S$ $[M +H]^+$ 378.07 found 378.05. ATR-FTIR (KBr): v = 2940, 2845, 2216, 1577, 1503, 1462, 1410, 1236, 993, 810, 765, 607 cm^{-1}.

2-((5-cyclohexyl-1-phenyl-1H-1,2,3-triazol-4-yl)tetrafluoro-λ^6-sulfaneyl)pyridine (4)

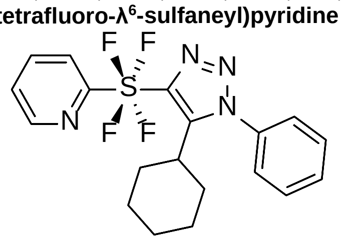

An oven dried test tube was charged with **3a** (0.1 mmol), phenyl azide (0.15 mmol) and toluene (0.5 mL) and allowed to stir at 110 °C for 48 h. [3] The reaction was allowed to cool to room temperature and the solvent was evaporated in vacuo to give the crude products.

The products were isolated using column chromatography on silica-gel, eluting with 10% n-Hexane/AcOEt mixture, to get pure **4** (9.47 mg, 23%) as an off white solidmp: 160.1 – 160.8 °C (5% EtOAc/hexane). ^{1}H NMR (500 MHz, $CDCl_3$) δ 8.62 (dt, *J* = 4.7, 1.4 Hz, 1H), 7.95 (d, *J* = 3.4 Hz, 2H), 7.64 – 7.59 (m, 1H), 7.59 – 7.54 (m, 2H), 7.50 – 7.47 (m, 1H), 7.46 – 7.43 (m, 2H), 3.57 – 3.45 (m, 1H), 1.85 – 1.73 (m, 2H), 1.66 (dt, *J* = 9.4, 3.3 Hz, 2H), 1.60 – 1.54 (m, 1H), 1.33 – 1.20 (m, 5H).^{19}F NMR (282 MHz, $CDCl_3$) *δ* 59.15 (s, 4F), ^{13}C NMR (126 MHz, $CDCl_3$) δ 170.30 (t, *J* = 30.0 Hz), 158.44 (t, *J* = 32.8 Hz),147.45, 139.00, 138.58, 137.52, 132.04, 130.80, 129.14, 127.99, 126.02, 121.67, 35.24, 30.93, 26.50, 25.51. HRMS (ESI+) Calculated for $C_{19}H_{20}F_4N_4SNa$ [M +Na]$^+$ 435.1242 found 435.1239. ATR-FTIR (KBr): ν = 2933, 2849, 1738, 1574, 1503, 1467, 1267, 1116 cm^{-1}.

5-cyclohexyl-2-methyl-3-phenyl-4-(tetrafluoro(pyridin-2-yl)-λ^6-sulfaneyl)-2,3-dihydroisoxazole (5)

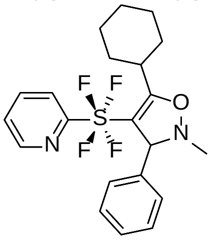

A test tube was charged with alkyne 3a (0.1 mmol), nitrone (0.11 mmol, 1.1 equiv), Et_3N (0.11 mmol, 1.1 equiv) and p-xylene (0.2 mL). The mixture was stirred at 80 °C for 16 h. [4] The reaction mixture was evaporated under reduced pressure to give the crude product, which was purified by neutral alumina column chromatography (10% n-Hexane/AcOEt) to provide $PyrSF_4$-isoxazoline **5** (31.7 mg, 74%) as a gummy compound. ^{1}H NMR (500 MHz, $CDCl_3$) δ 8.48 (dd, *J* = 4.7, 1.9 Hz, 1H), 7.78 (t, *J* = 7.9 Hz, 1H), 7.66 (d, *J* = 8.4 Hz, 1H), 7.36 – 7.27 (m, 6H), 4.99 (s, 1H), 3.25 (tt, *J* = 12.1, 3.5 Hz, 1H), 2.90 (s, 3H), 1.97 – 1.90 (m, 2H), 1.85 – 1.80 (m, 2H), 1.71 (d, *J* = 12.7 Hz, 1H), 1.64 – 1.52 (m, 2H), 1.40 – 1.31 (m, 2H), 1.28 – 1.22 (m, 1H).^{19}F NMR (282 MHz, $CDCl_3$) *δ* 62.44 (s, 4F). ^{13}C NMR (126 MHz, $CDCl_3$) δ 171.41 (p, *J* = 32.2, 31.8 Hz), 160.52, 147.14, 141.08, 138.29, 130.99 (t, *J* = 27.3 Hz), 128.59, 127.92, 127.09, 125.66, 121.33, 46.73, 36.62, 29.63, 29.35, 26.06, 25.81, 25.74. HRMS (ESI+) Calculated for $C_{21}H_{25}F_4N_2SO$ [M +H]$^+$ 429.1624 found 429.1627. ATR-FTIR (KBr): ν = 3051, 2937, 2857, 2351, 1650, 1570, 1463, 1431, 1164 cm^{-1}.

V. Experiments for determining the reactivity of C-Br bonds

a.

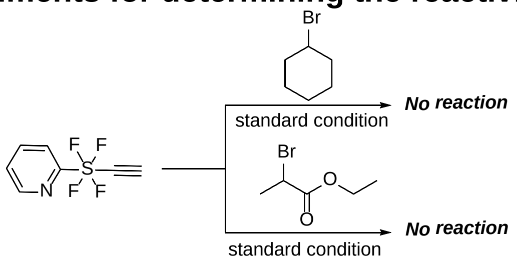

b.

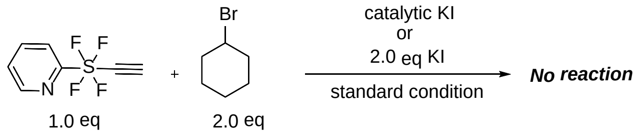

c.

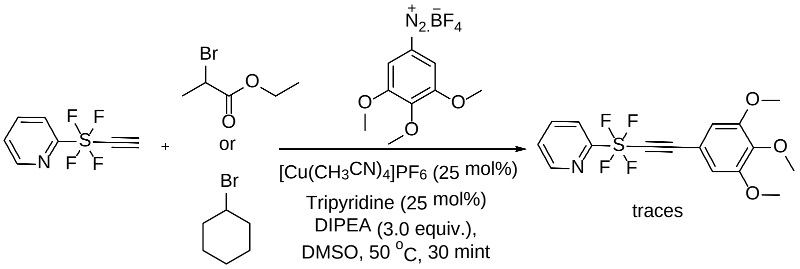

d.

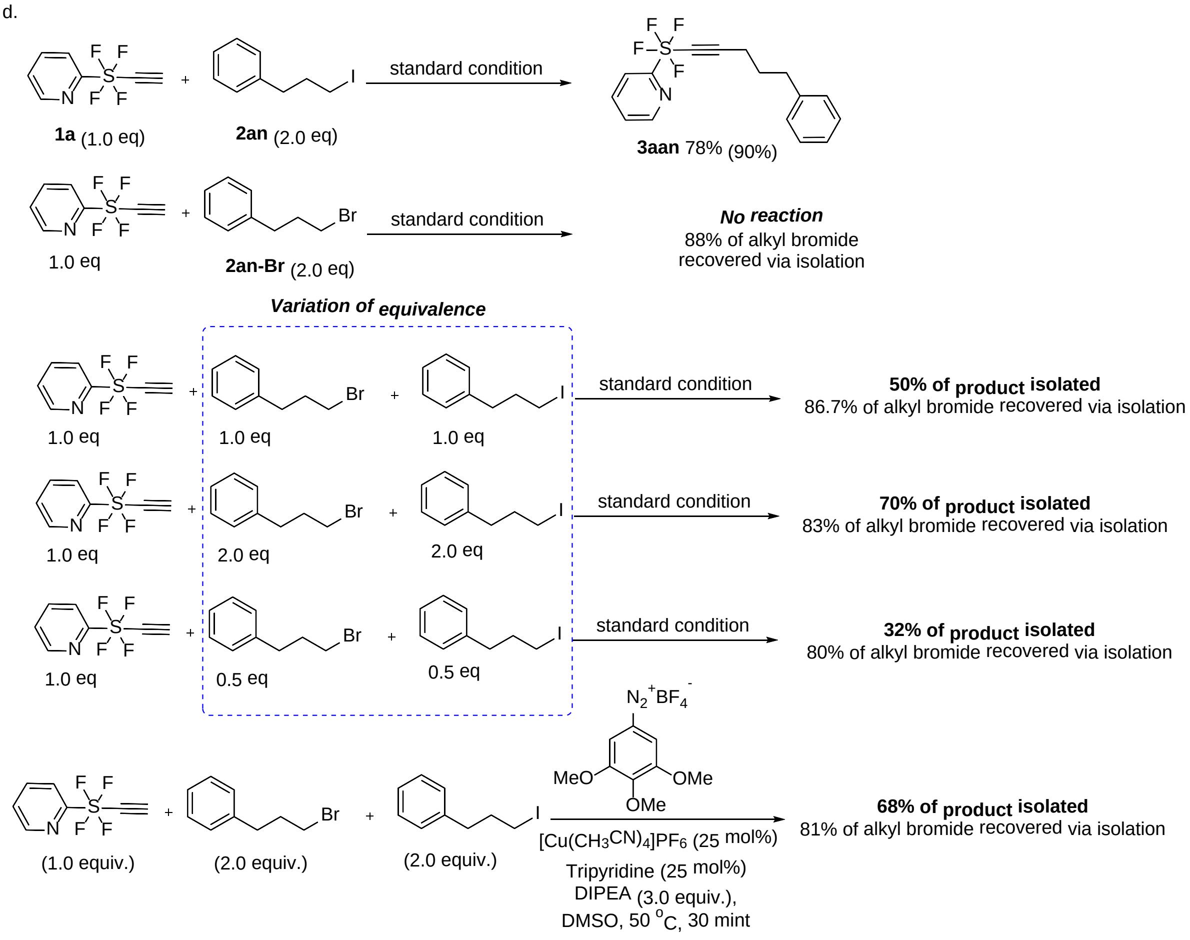

VI. Experiment with TEMPO

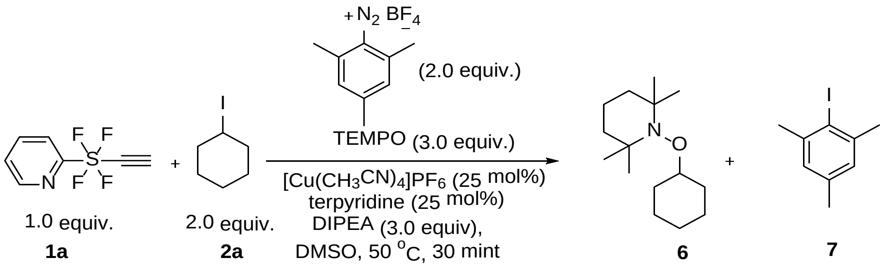

An oven-dried 4 mL vial equipped with a magnetic stir bar was charged with the alkyl iodide **2a** (84.0 mg, 0.4 mmol, 2.0 equiv.), $[Cu(CH_3CN)_4]PF_6$ (18.6 mg, 0.05 mmol, 0.25 equiv.), terpyridine (11.7 mg, 0.05 mmol, 0.25 equiv.), DIPEA (77.6 mg, 0.6 mmol, 3.0 equiv.) TEMPO (93.6 mg, 0.6 mmol, 3.0 equiv.) and Pyridine SF_4 alkyne **1a** (42.2 mg, 0.2 mmol, 1.0 equiv.) dissolved in 0.5 mL of dry DMSO, the dark brown solution was heated to 50 ºC and was stirred for 5 min. Aryldiazonium salt (93.6 mg, 0.4 mmol, 2.0 equiv.) taken in dry DMSO (0.5 mL) slowly added to the vial within 5 minutes via syringe. The resultant mixture was stirred at 50 ºC for an additional 10 minutes. The reaction mixture was then cooled down to room temperature, diluted with water (10 mL) and extracted with ethyl acetate (10 mL × 3). The organic layers were combined, washed with brine (10 mL) and concentrated under vacuo. The crude obtained was subjected to GC-MS and the obtained results were demonstrated as follows.[5]

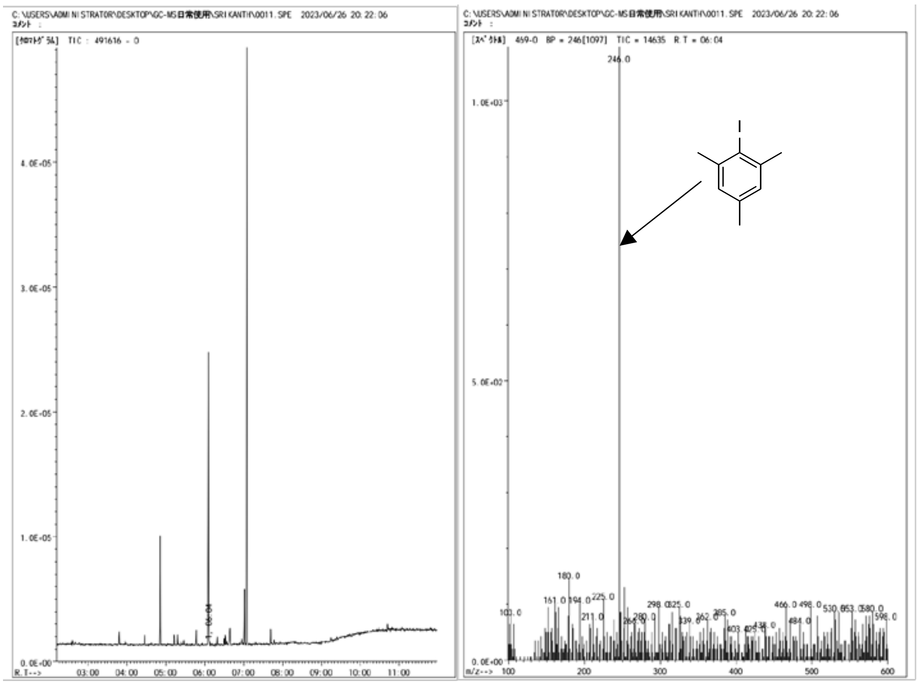

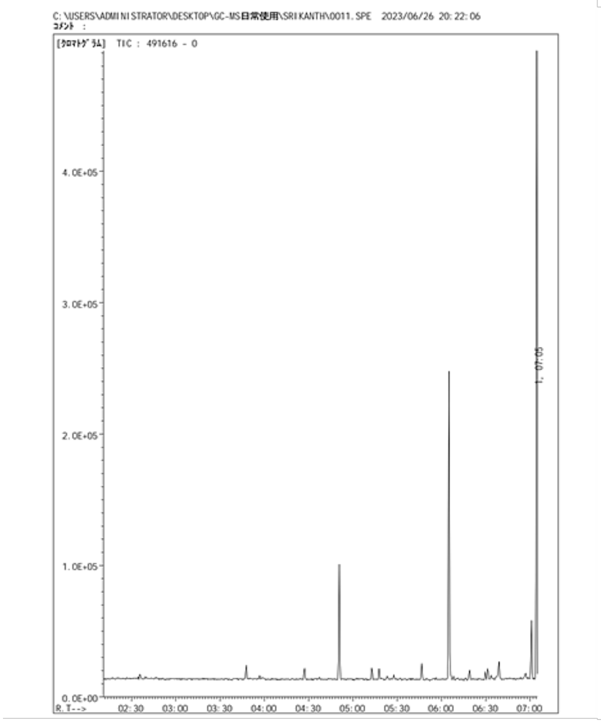

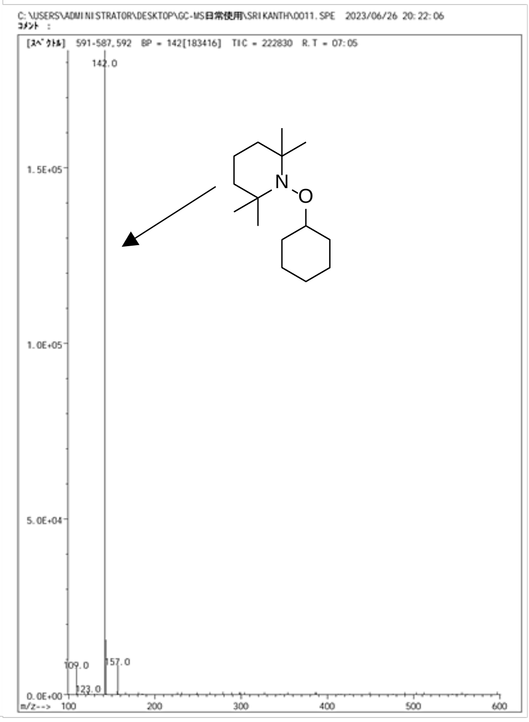

VII. References:

[1.0] K. Iwaki, K. Maruno, O. Nagata, N. Shibata *J. Org. Chem.* **2022,** *87***,** 6302−6311.

[2.0] A. R. Tripathy, A. Kumar, R. Rahmathulla, A. K. Jha, V. R. Yatham, *Org. Lett*. **2022**, *24,* 5186-5191.

[3.0] P. Das, K. Niina, T. Hiromura, E. Tokunaga, N. Saito, N. Shibata, *Chem. Sci.* **2018**, *9*, 4931-4936.

[4.0] K. Maruno, K. Hada, Y. Sumii, O. Nagata, N. Shibata, *Org. Lett*. **2022**, *24*, 3755-3759.

[5.0] X. Lv, R. Martin*, Org. Lett.* **2023**, *20*, 3750–3754.

VIII. NMR spectra

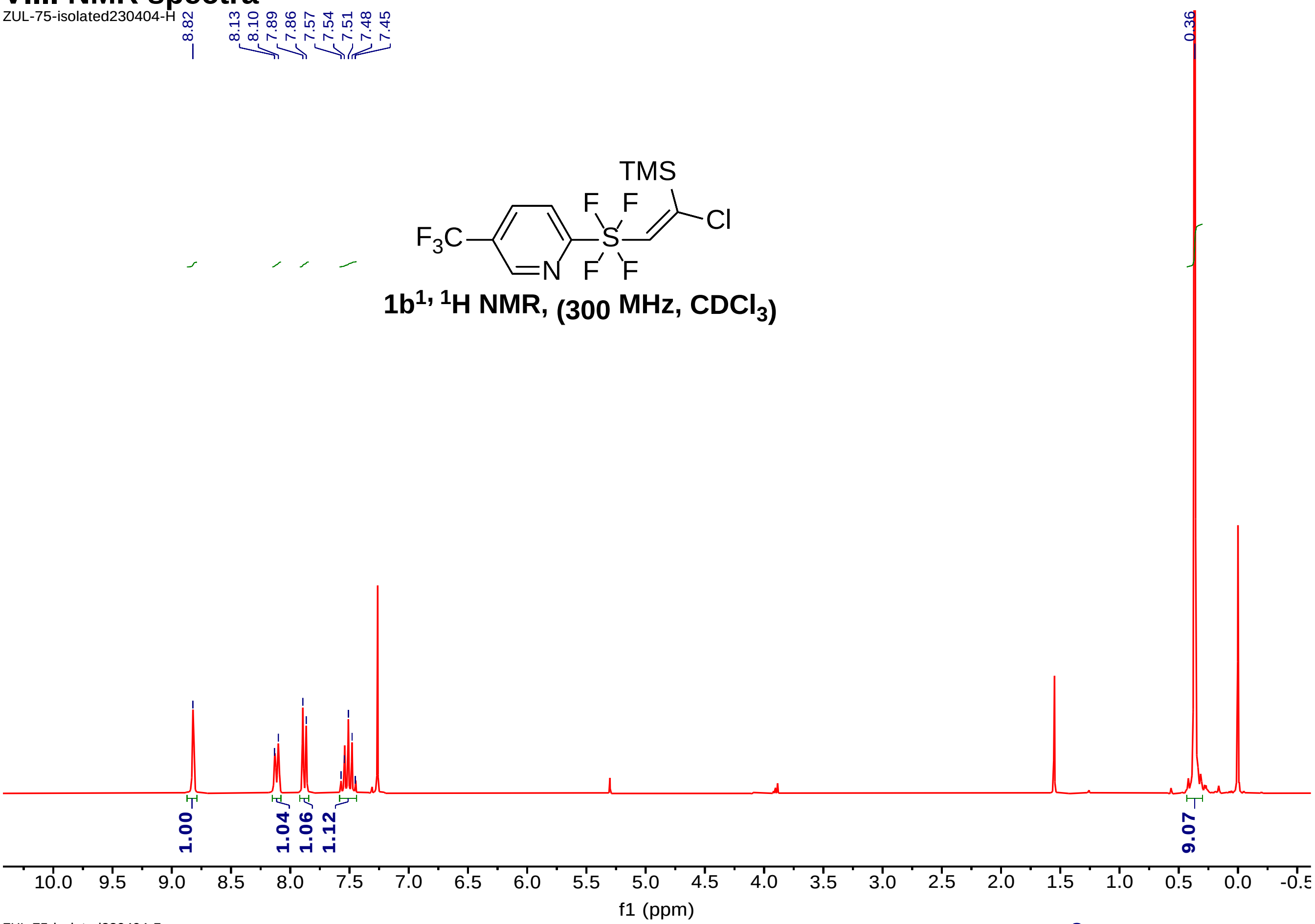

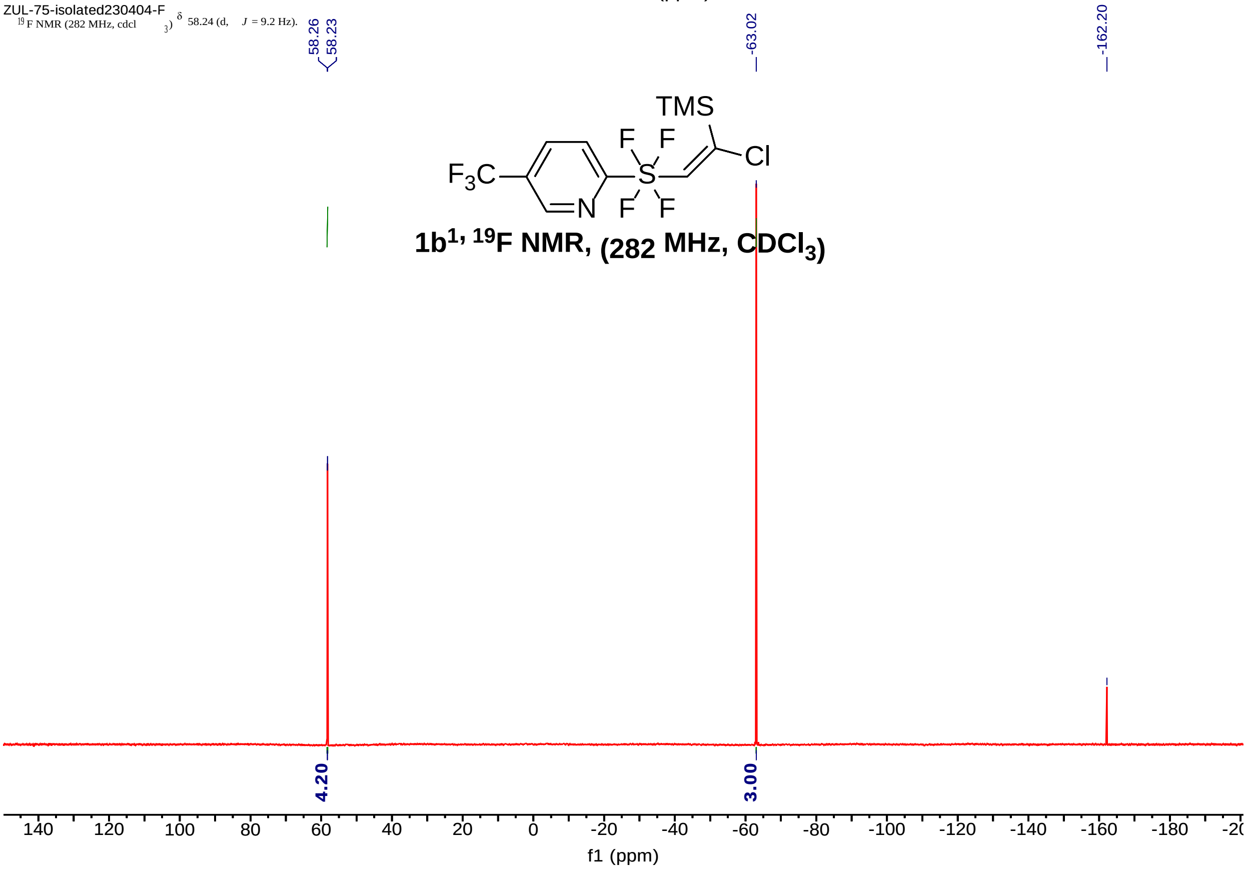

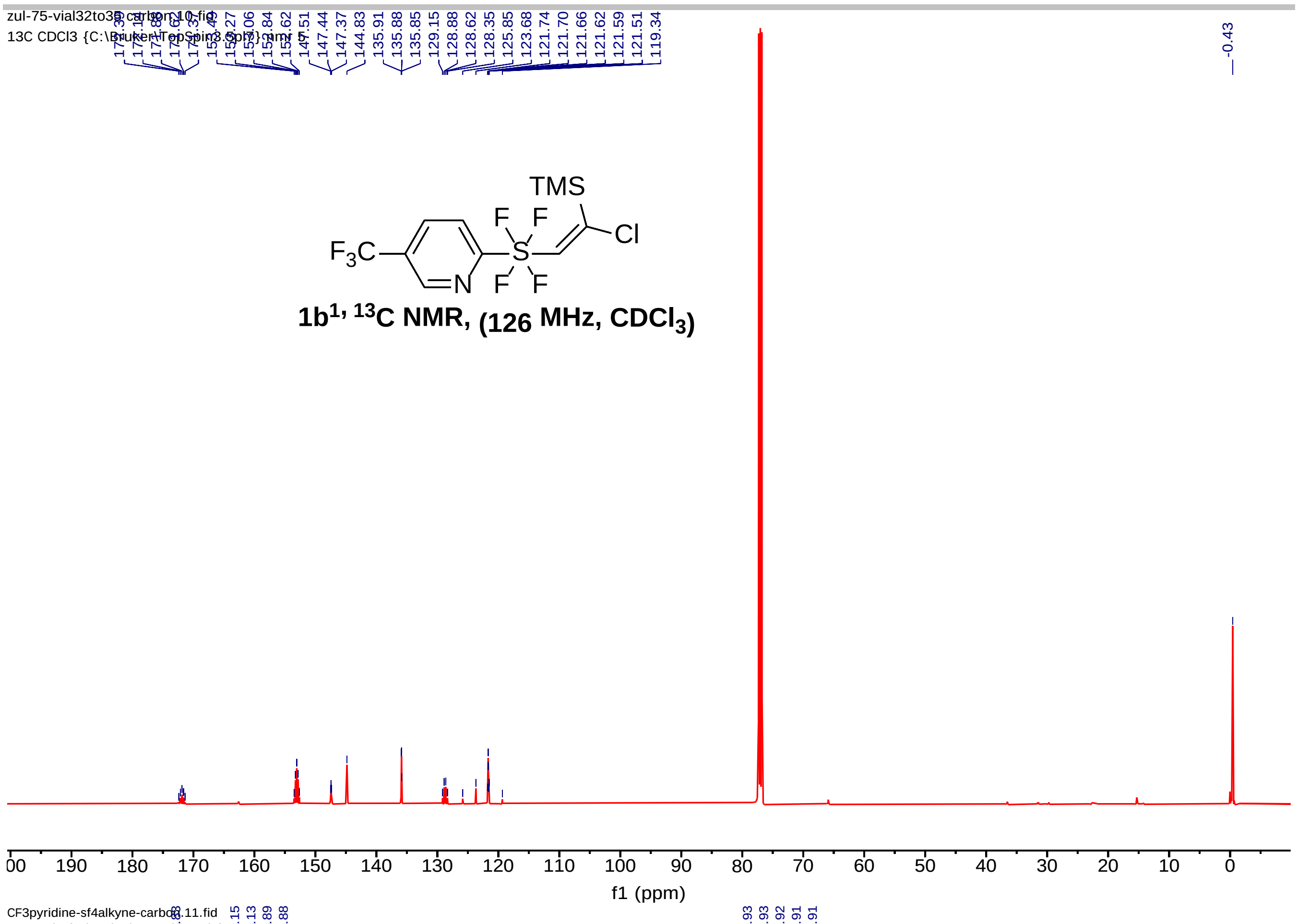
1b[1], ^{13}C NMR, (126 MHz, $CDCl_3$)
f1 (ppm)

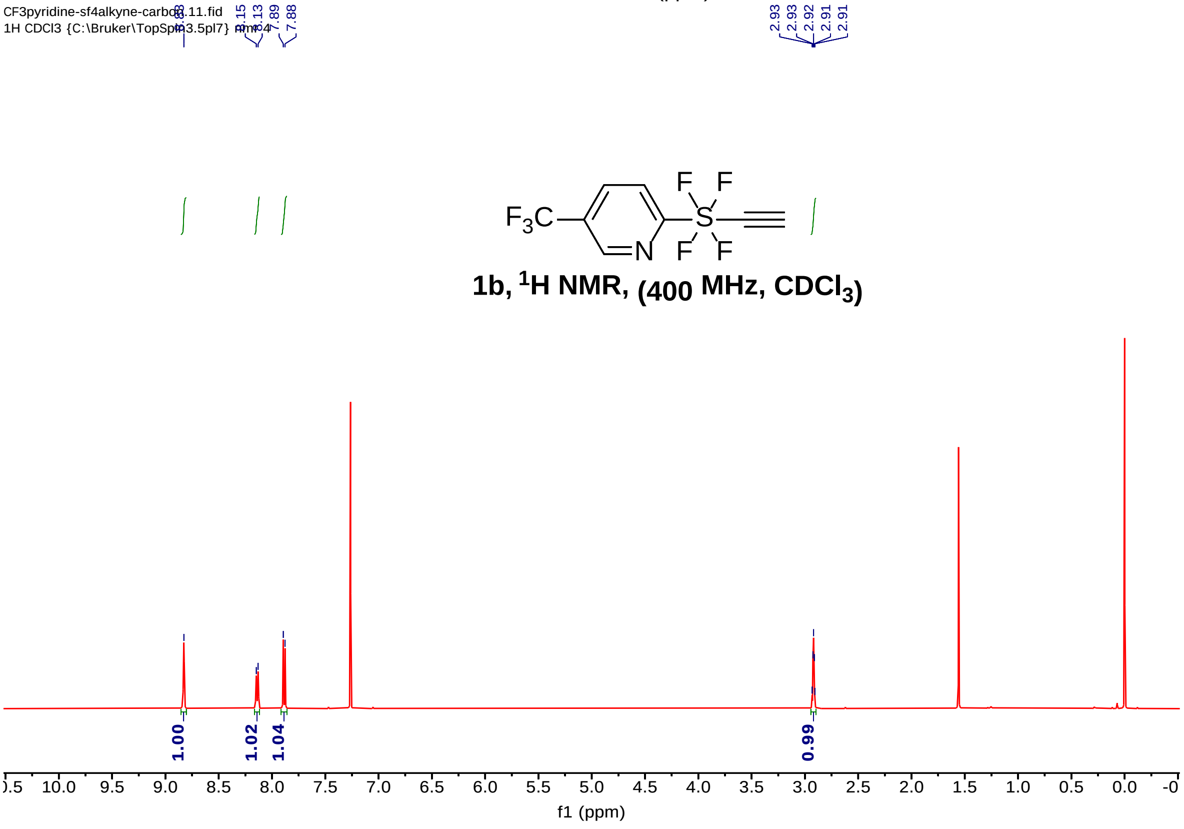
1b, ^{1}H NMR, (400 MHz, $CDCl_3$)
f1 (ppm)

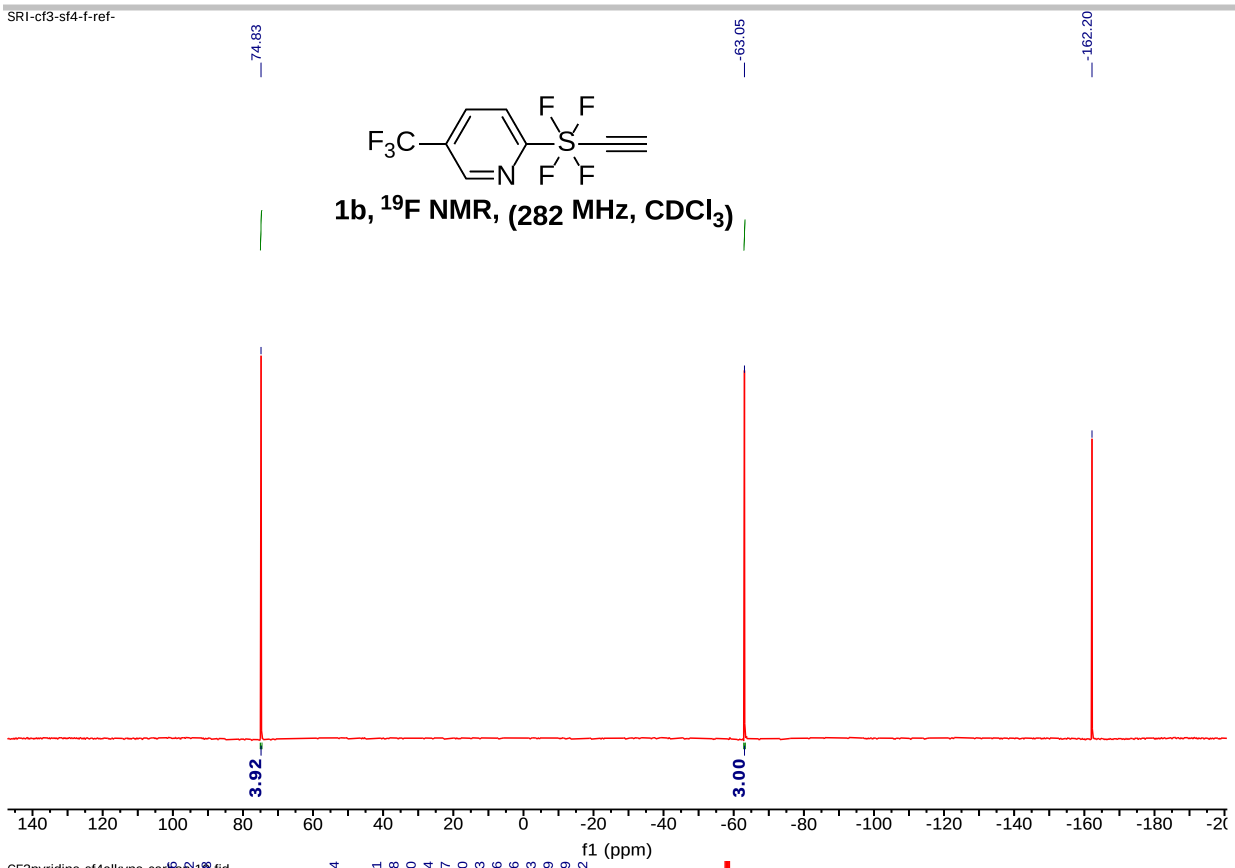
SRI-cf3-sf4-f-ref-
-74.83
-63.05
-162.20
1b, ^{19}F NMR, (282 MHz, CDCl$_3$)
3.92
3.00
140 120 100 80 60 40 20 0 -20 -40 -60 -80 -100 -120 -140 -160 -180
f1 (ppm)

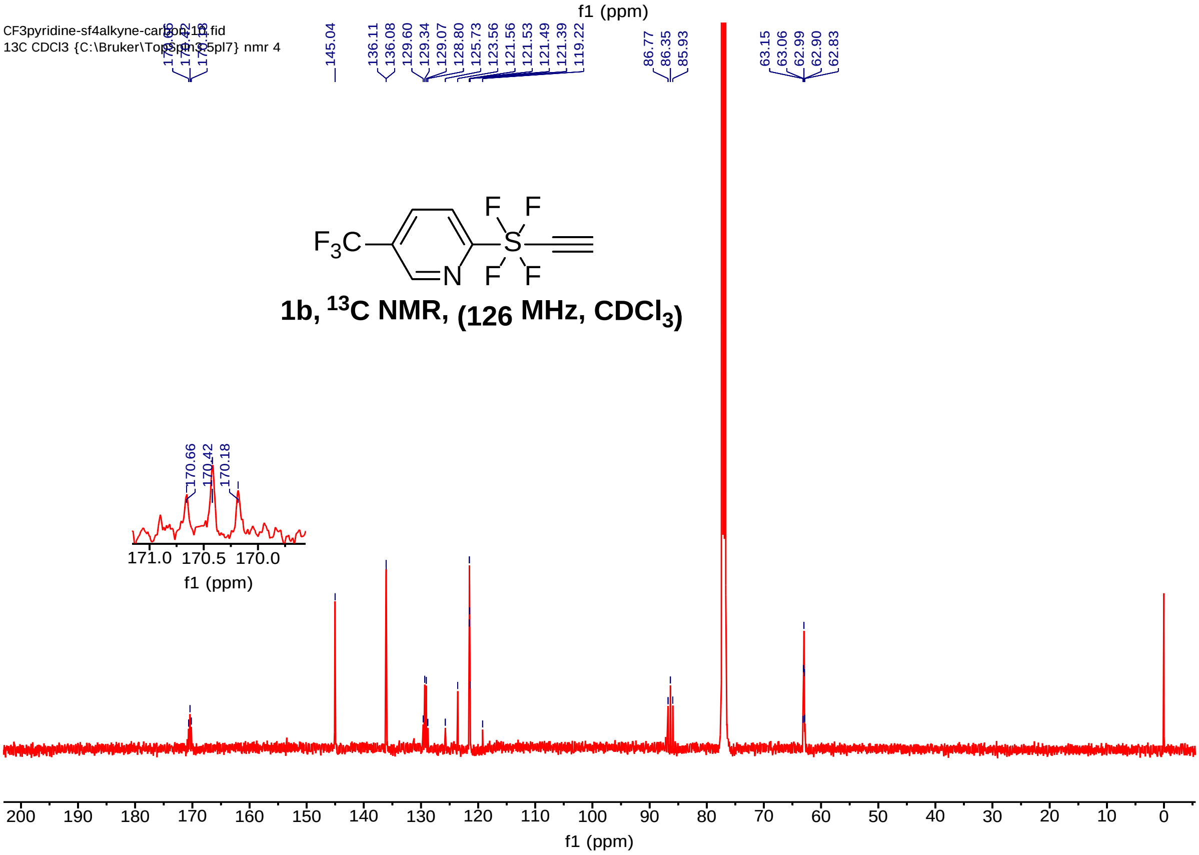
CF3pyridine-sf4alkyne-carbon.1.fid
13C CDCl3 {C:\Bruker\TopSpin3.5pl7} nmr 4
145.04
136.11
136.08
129.60
129.34
129.07
128.80
125.73
123.56
121.56
121.53
121.49
121.39
119.22
86.77
86.35
85.93
63.15
63.06
62.99
62.90
62.83
1b, ^{13}C NMR, (126 MHz, CDCl$_3$)
170.66
170.42
170.18
171.0 170.5 170.0
f1 (ppm)
200 190 180 170 160 150 140 130 120 110 100 90 80 70 60 50 40 30 20 10 0
f1 (ppm)

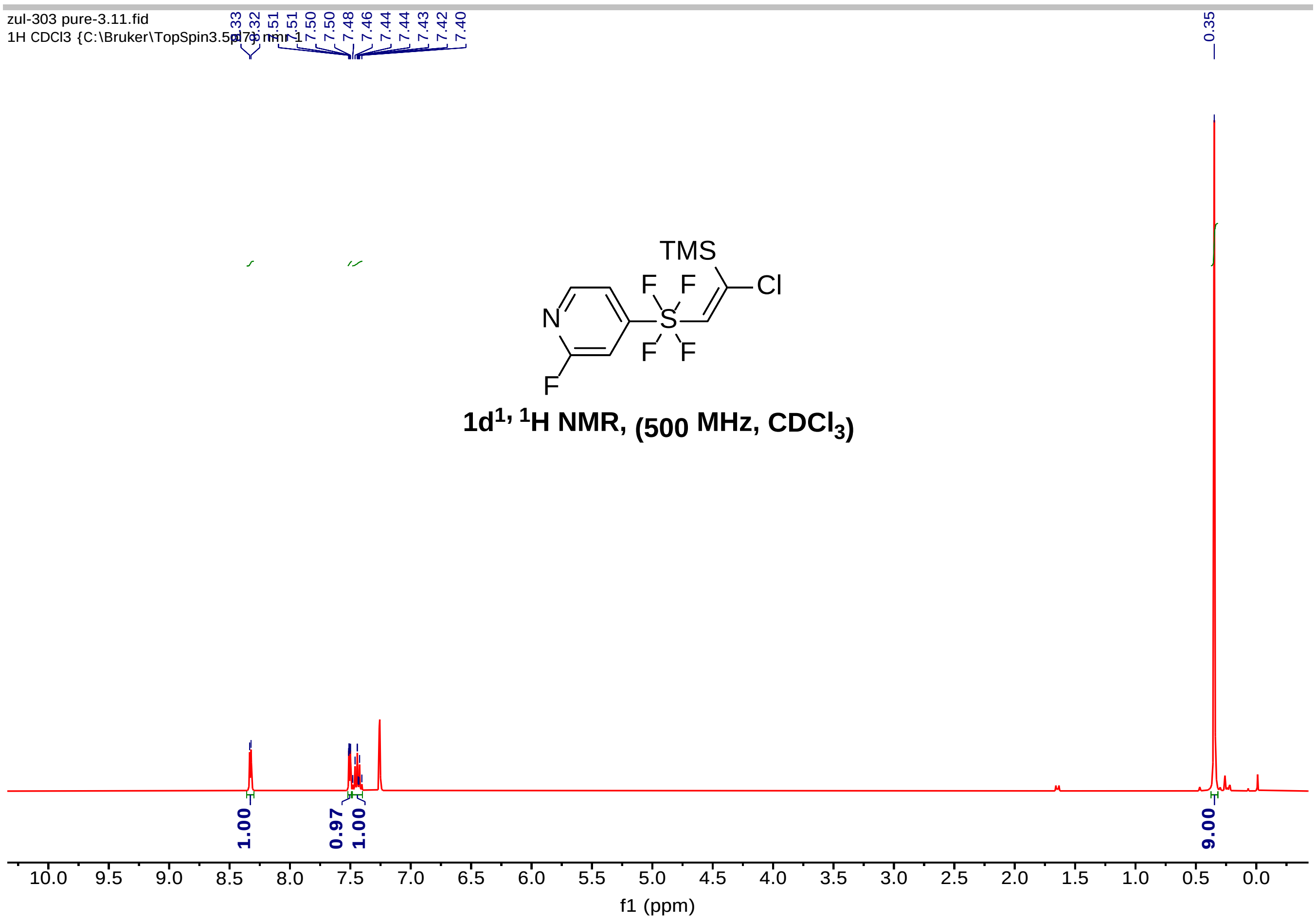
zul-303 pure-3.11.fid
1H CDCl3 {C:\Bruker\TopSpin3.5pl7} nmr 1
TMS
Cl
N
F
S
1d1, 1H NMR, (500 MHz, CDCl3)
f1 (ppm)

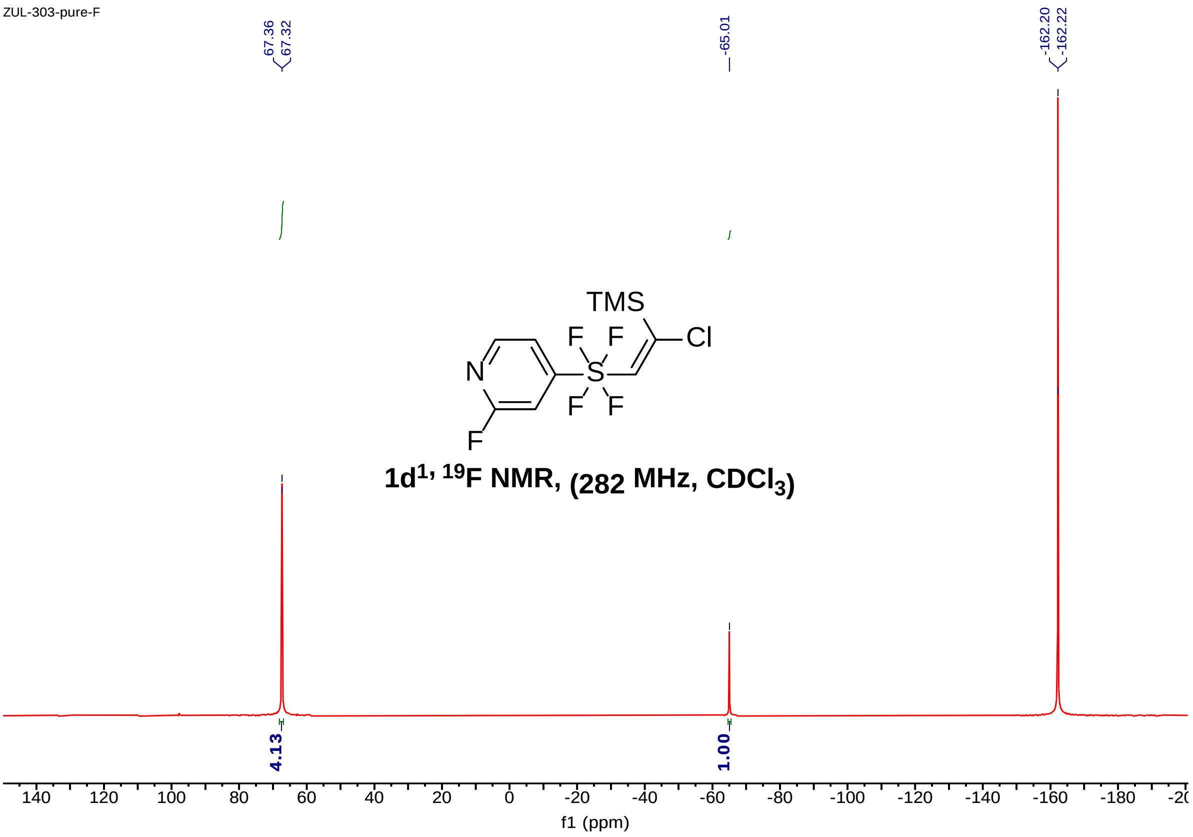
ZUL-303-pure-F
TMS
Cl
N
F
S
1d1, 19F NMR, (282 MHz, CDCl3)
f1 (ppm)

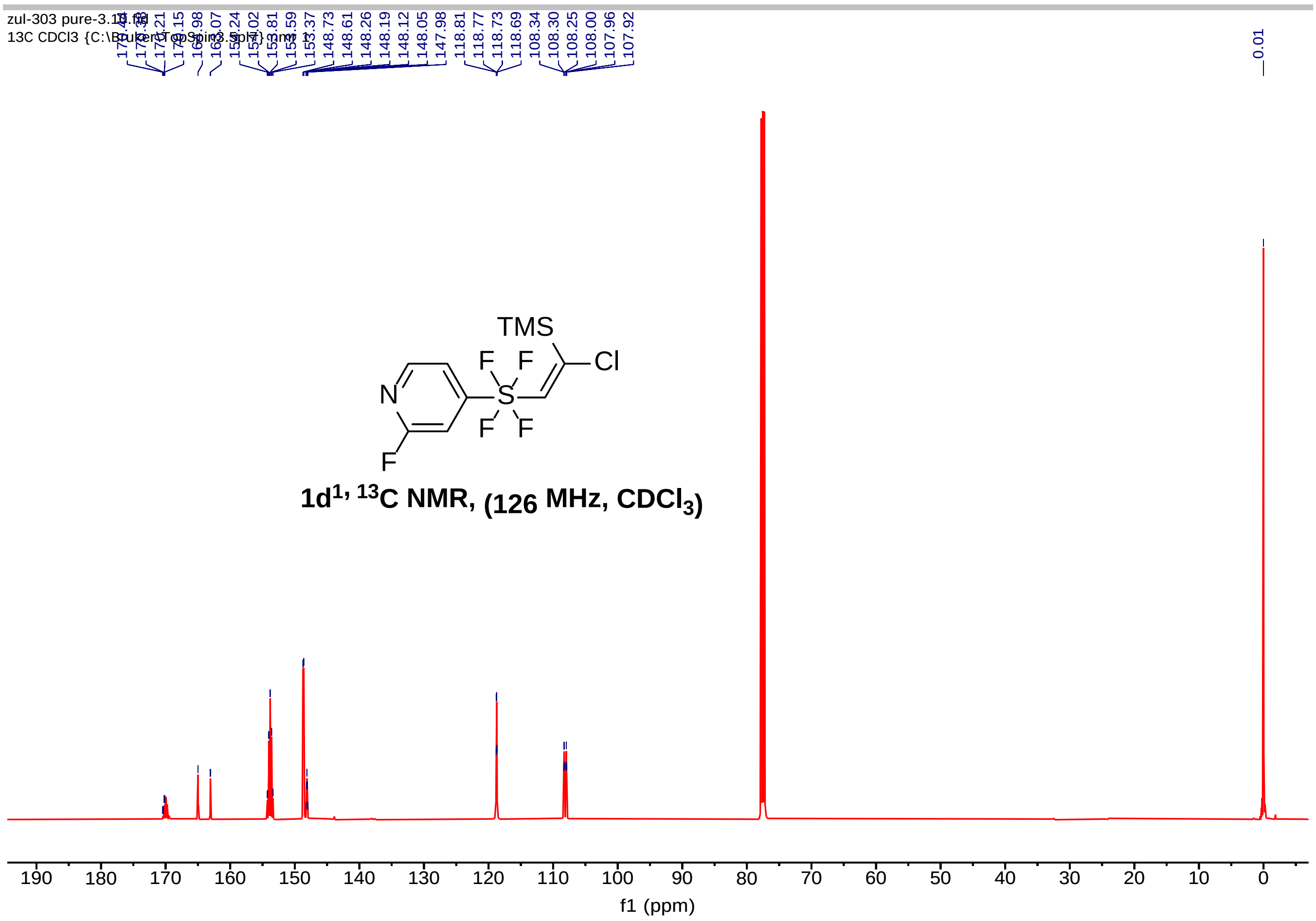
zul-303 pure-3.10.fid
13C CDCl3 {C:\Bruker\TopSpin3.5pl7} nmr 1
TMS
Cl
F F
N
S
F F
F
1d1, 13C NMR, (126 MHz, CDCl3)
f1 (ppm)

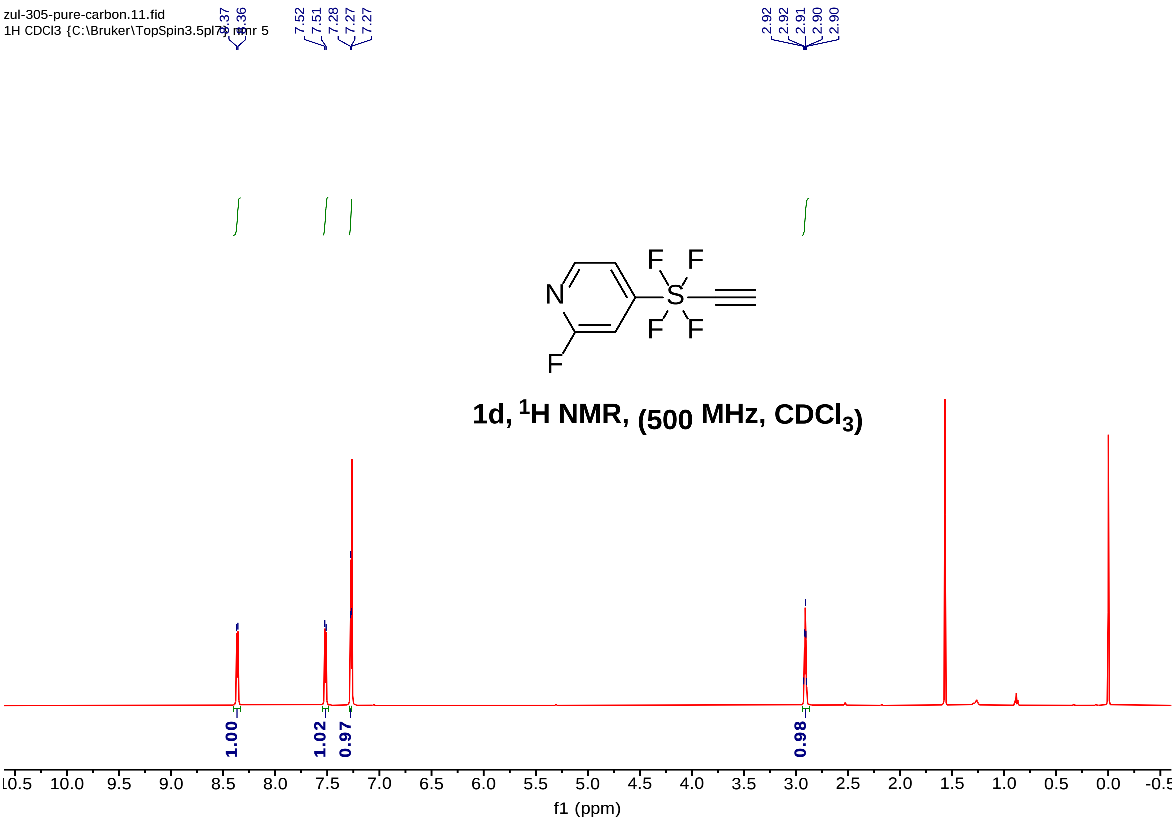
zul-305-pure-carbon.11.fid
1H CDCl3 {C:\Bruker\TopSpin3.5pl7} nmr 5
F F
N
S
F F
F
1d, 1H NMR, (500 MHz, CDCl3)
1.00
1.02
0.97
0.98
f1 (ppm)

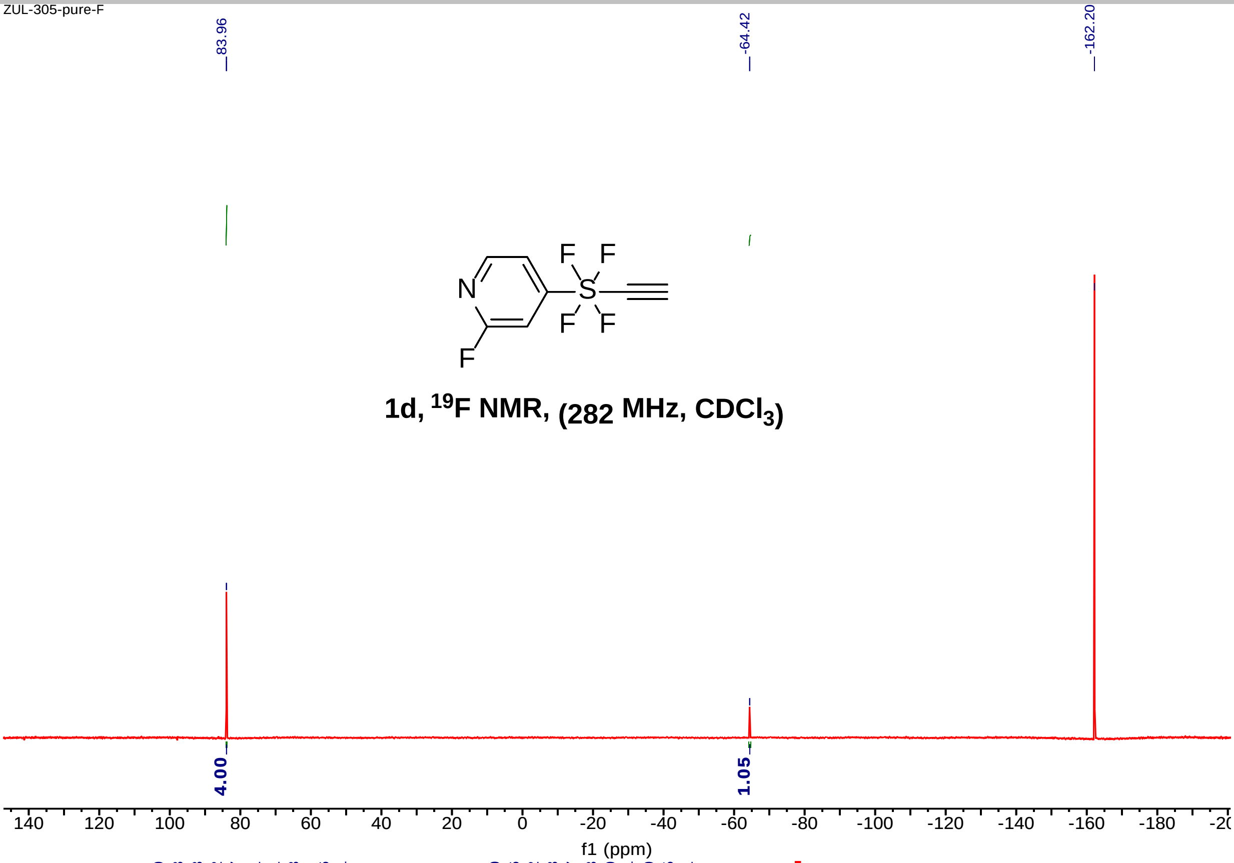
ZUL-305-pure-F
83.96
-64.42
-162.20
1d, ^{19}F NMR, (282 MHz, $CDCl_3$)
4.00
1.05
140 120 100 80 60 40 20 0 -20 -40 -60 -80 -100 -120 -140 -160 -180
f1 (ppm)

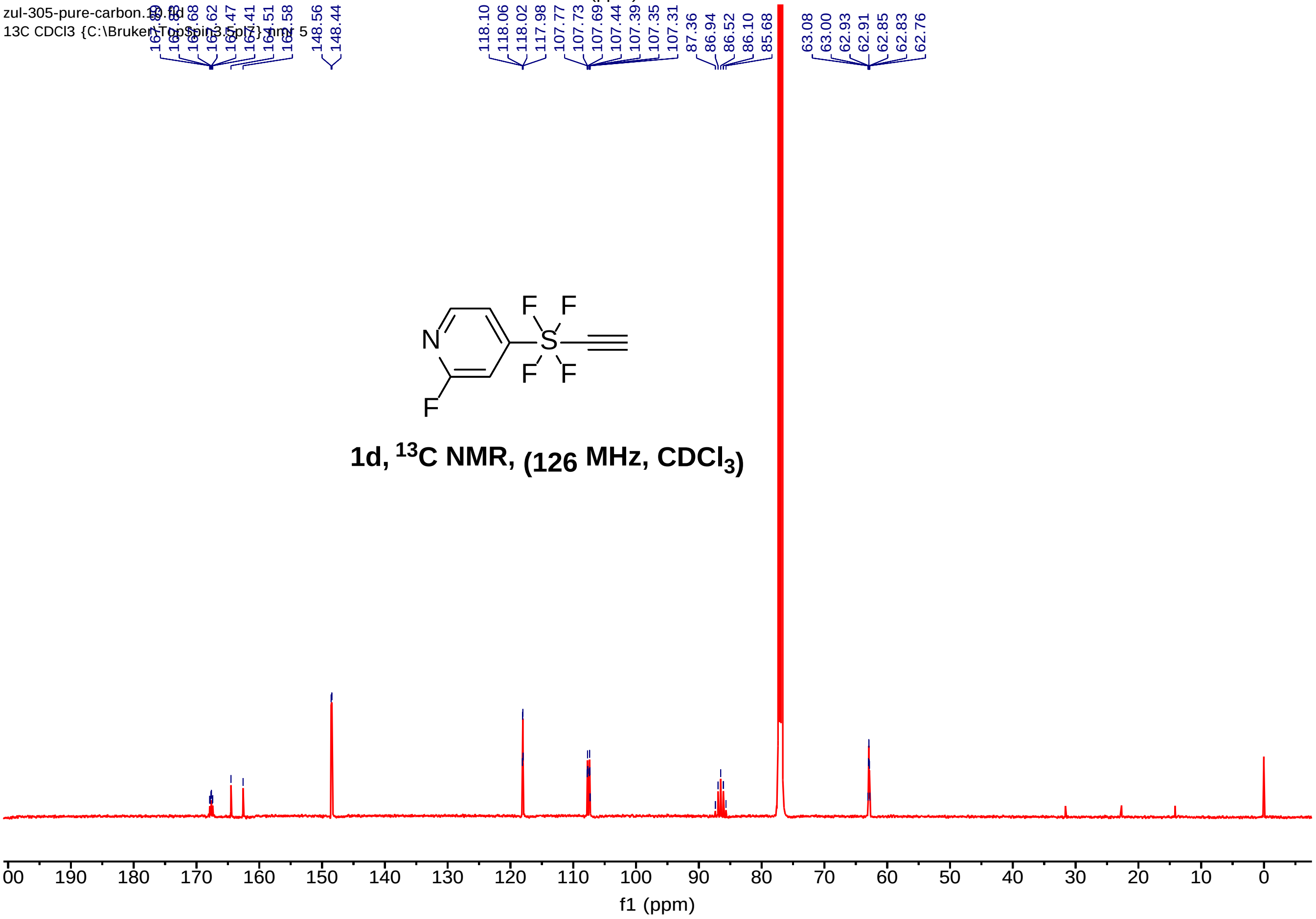
zul-305-pure-carbon.10.fid
13C CDCl3 {C:\Bruker\TopSpin3.5pl7} nmr 5
148.56
148.44
118.10
118.06
118.02
117.98
107.77
107.73
107.69
107.44
107.39
107.35
107.31
87.36
86.94
86.52
86.10
85.68
63.08
63.00
62.93
62.91
62.85
62.83
62.76
1d, ^{13}C NMR, (126 MHz, $CDCl_3$)
190 180 170 160 150 140 130 120 110 100 90 80 70 60 50 40 30 20 10 0
f1 (ppm)

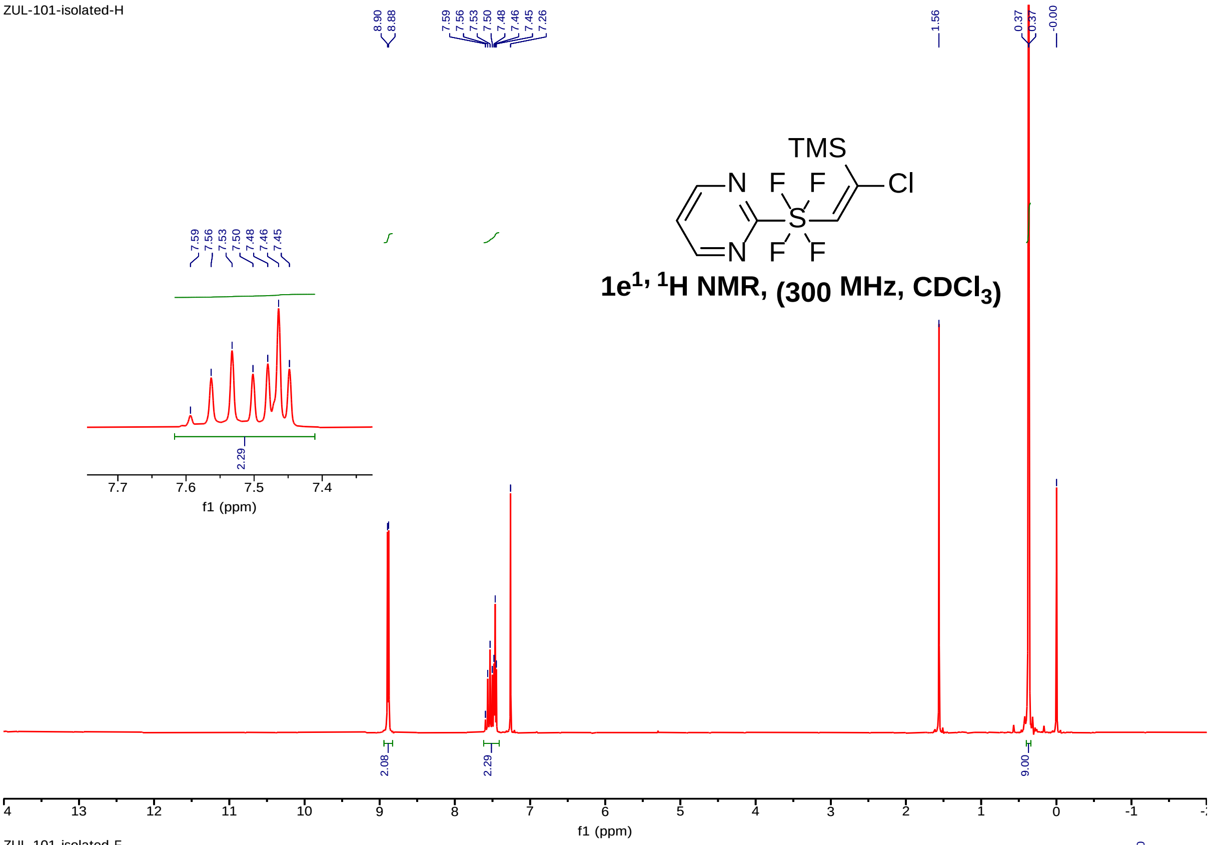
ZUL-101-isolated-H
TMS
Cl
N
F F
S
F F
N
1e1, 1H NMR, (300 MHz, CDCl3)
f1 (ppm)

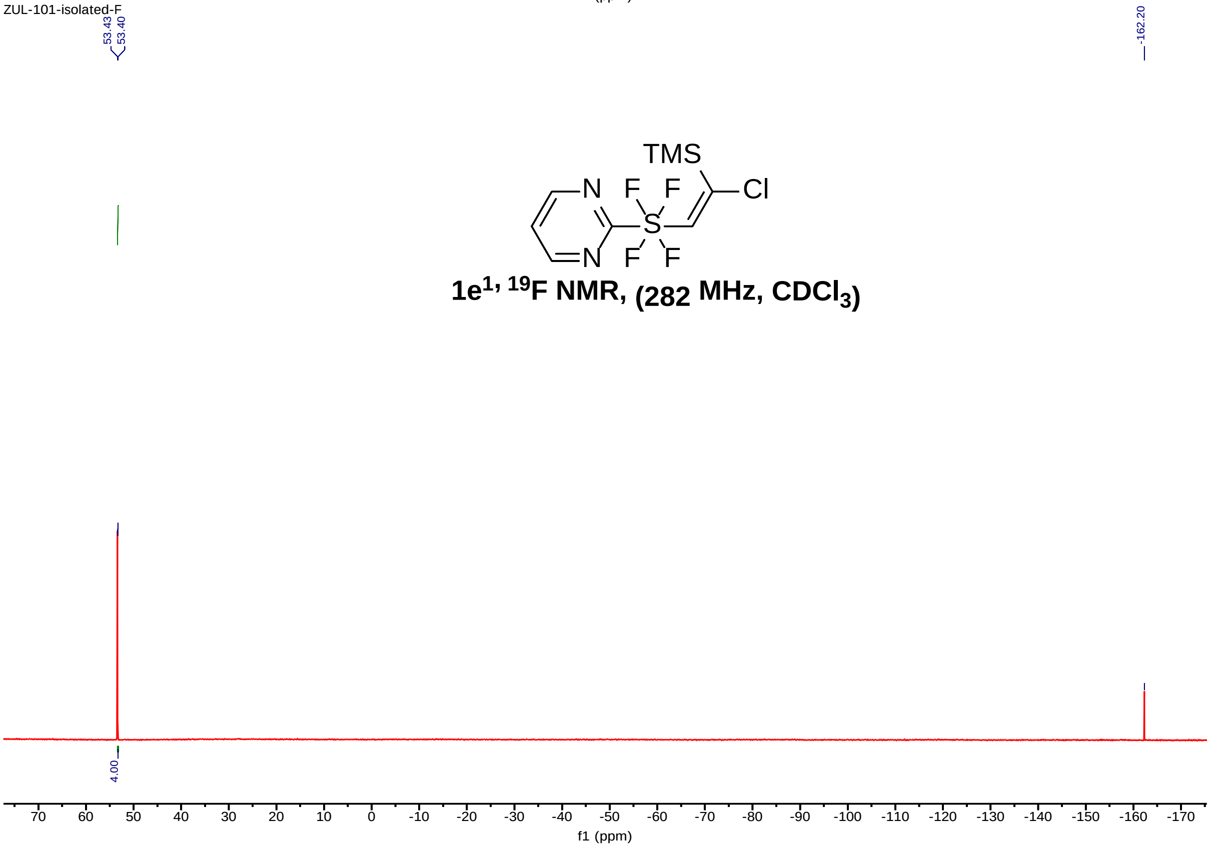
ZUL-101-isolated-F
TMS
Cl
N
F F
S
F F
N
1e1, 19F NMR, (282 MHz, CDCl3)
f1 (ppm)

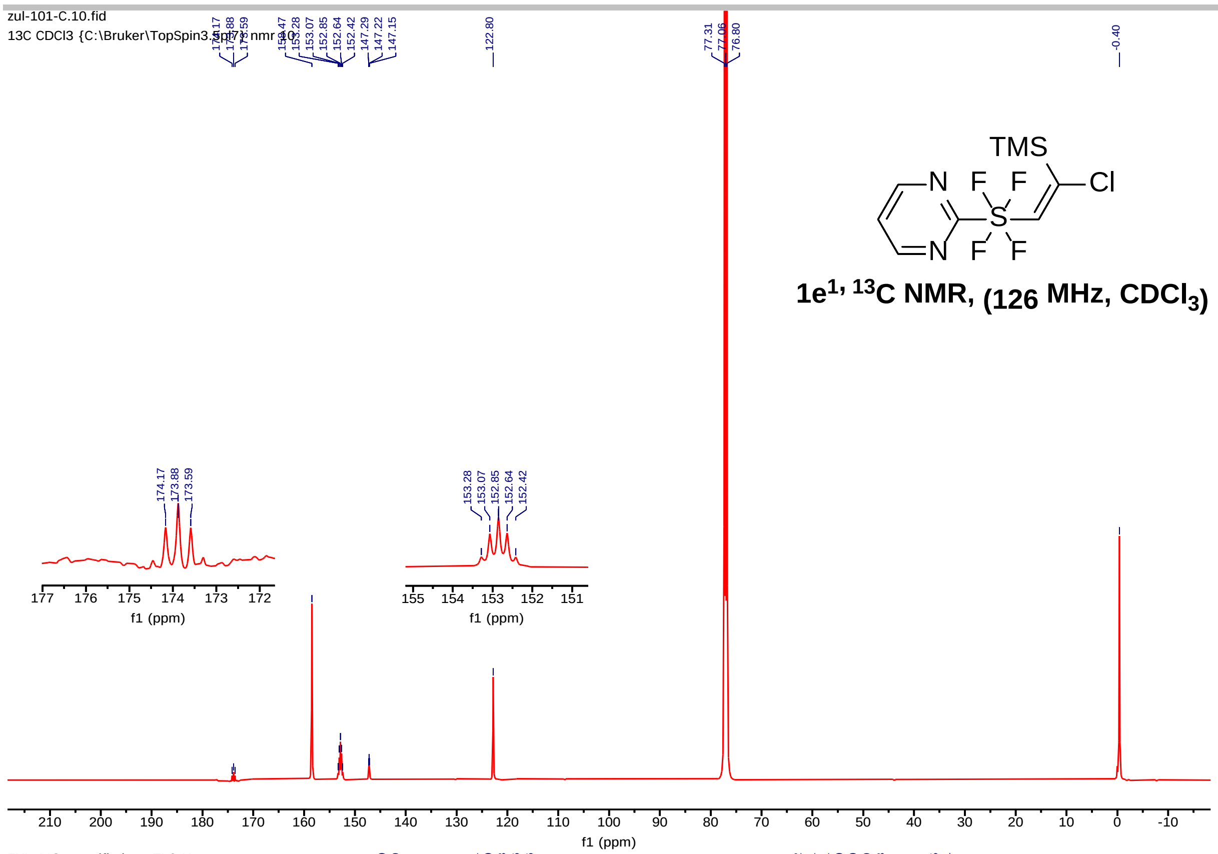
zul-101-C.10.fid
13C CDCl3 {C:\Bruker\TopSpin3.5pl7} nmr 10
TMS
Cl
N
F
S
1e1, 13C NMR, (126 MHz, CDCl3)
f1 (ppm)

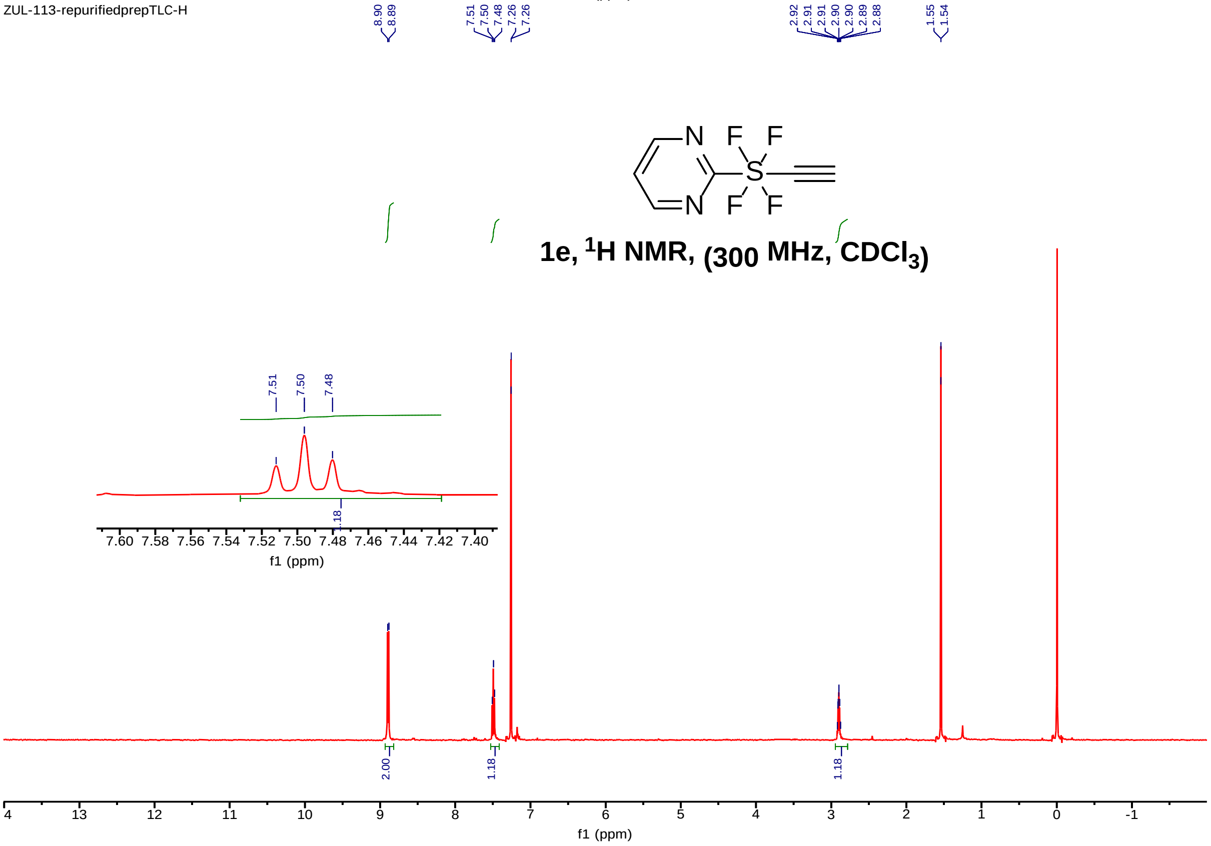
ZUL-113-repurifiedprepTLC-H
N
F
S
1e, 1H NMR, (300 MHz, CDCl3)
f1 (ppm)

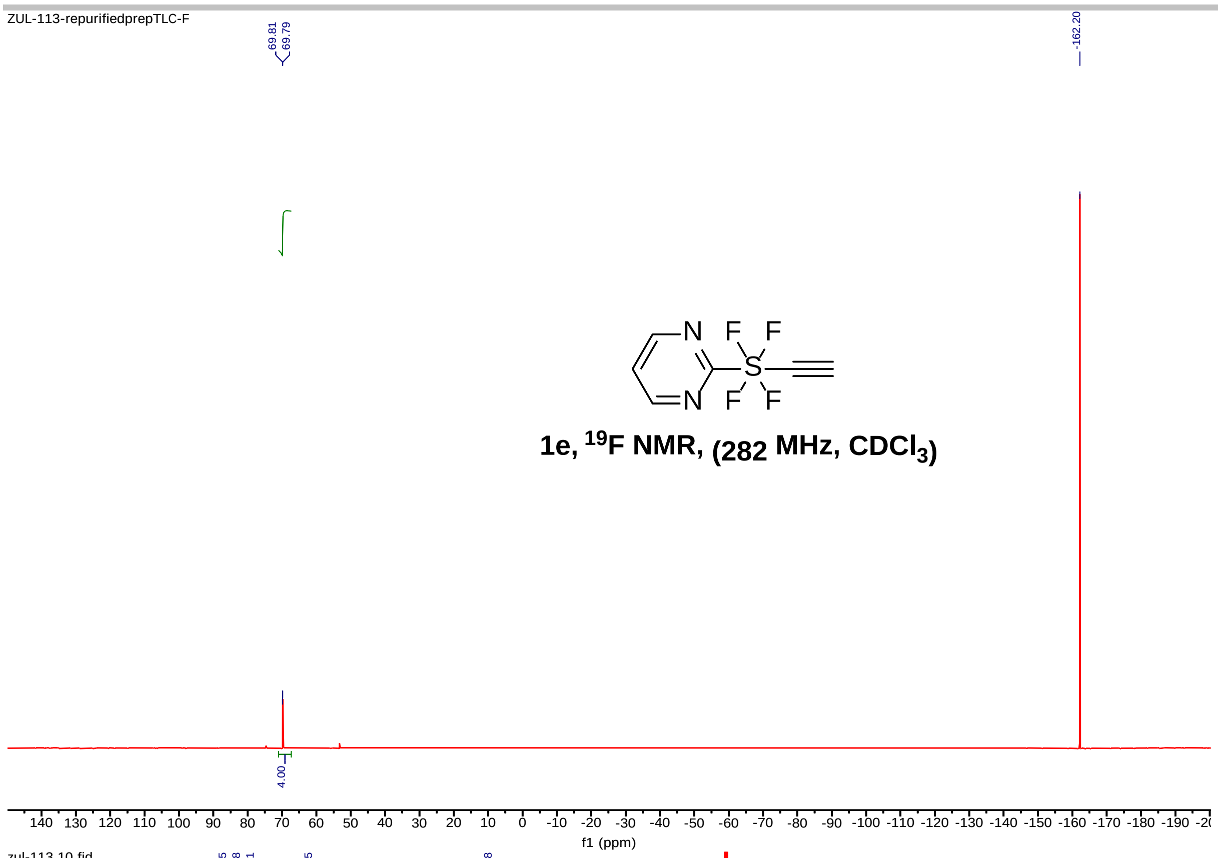
ZUL-113-repurifiedprepTLC-F
69.81
69.79
-162.20
1e, 19F NMR, (282 MHz, CDCl3)
4.00
f1 (ppm)

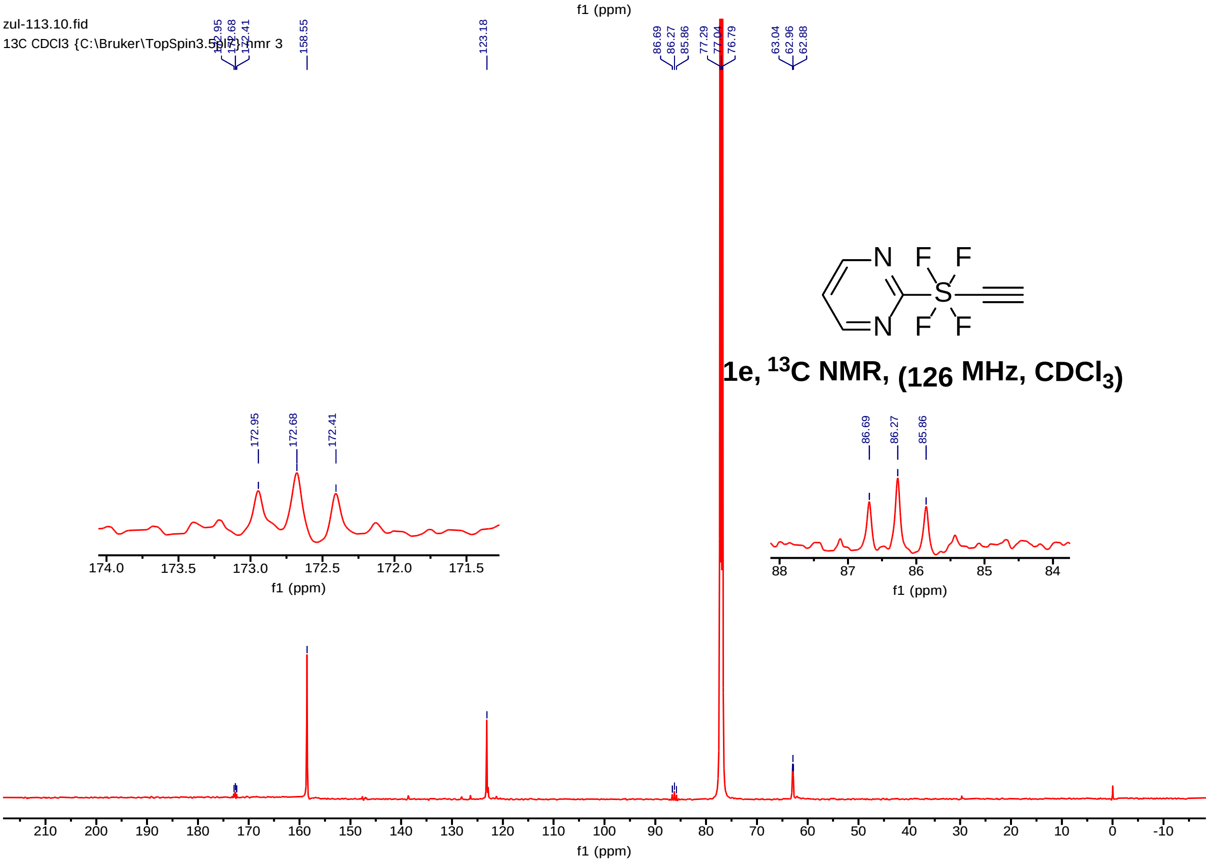
zul-113.10.fid
13C CDCl3 {C:\Bruker\TopSpin3.5pl7} nmr 3
172.95
172.68
172.41
158.55
123.18
86.69
86.27
85.86
77.29
77.04
76.79
63.04
62.96
62.88
1e, 13C NMR, (126 MHz, CDCl3)
f1 (ppm)

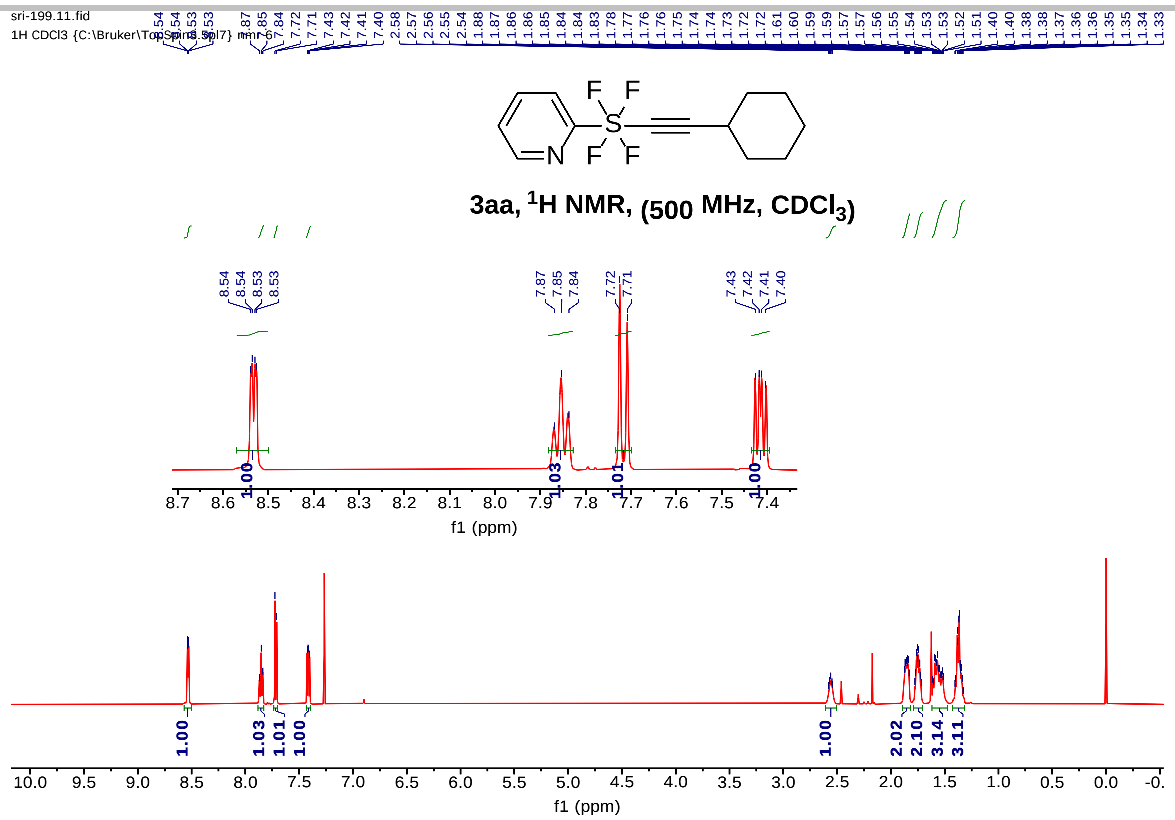
sri-199.11.fid
1H CDCl3 {C:\Bruker\TopSpin3.5pl7} nmr 6
3aa, 1H NMR, (500 MHz, CDCl3)
f1 (ppm)
f1 (ppm)

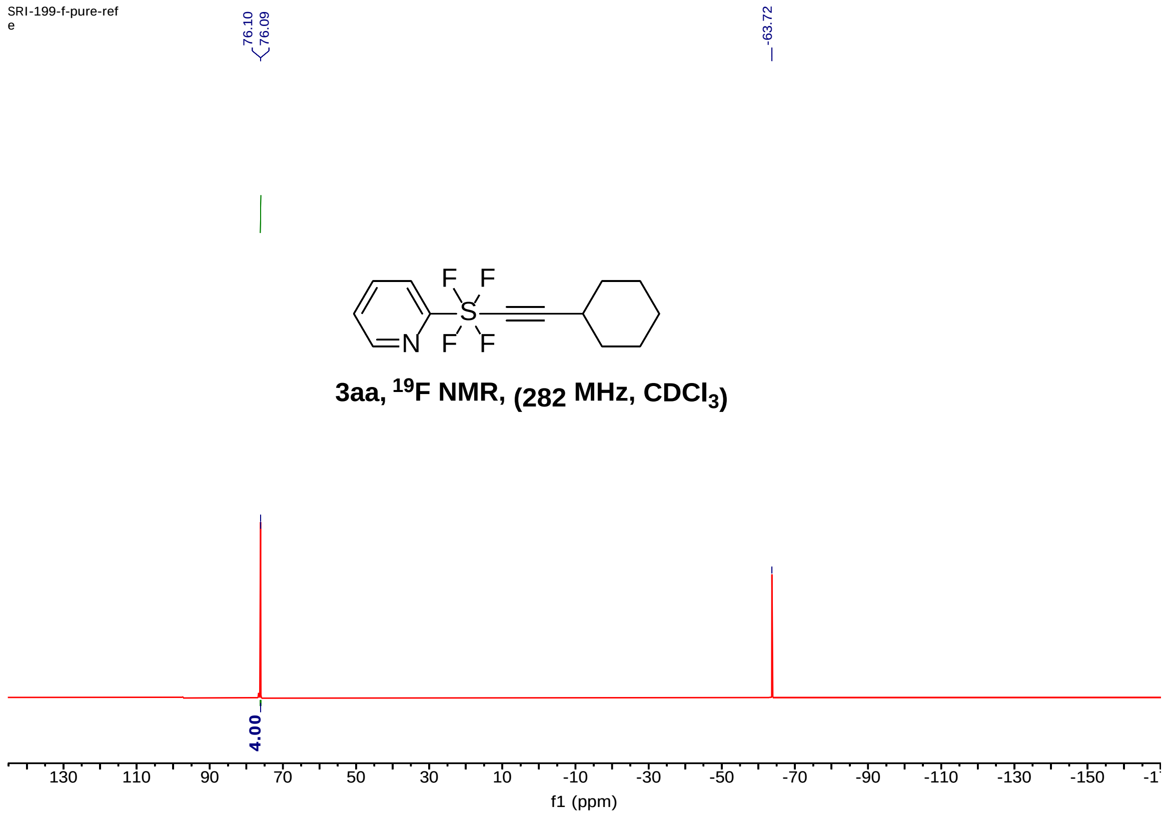
SRI-199-f-pure-ref
e
76.10
76.09
-63.72
3aa, 19F NMR, (282 MHz, CDCl3)
4.00
f1 (ppm)

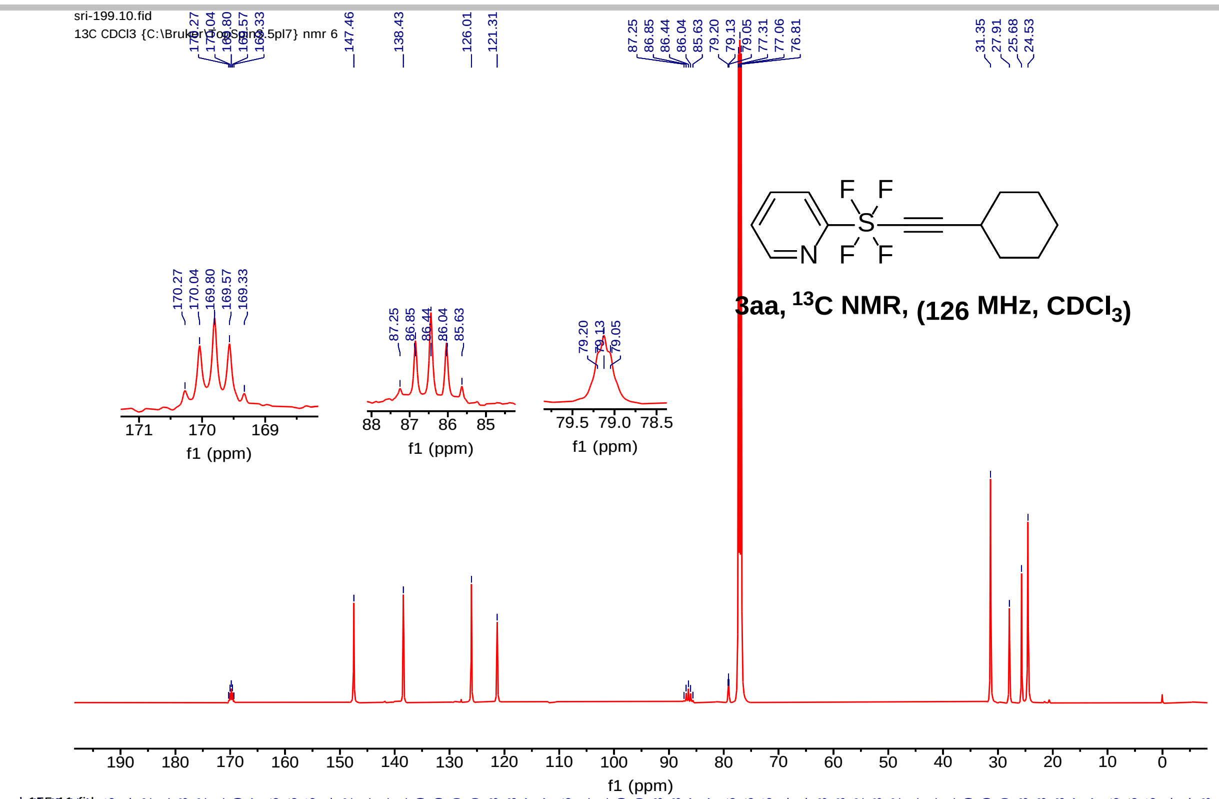
sri-199.10.fid
13C CDCl3 {C:\Bruker\TopSpin3.5pl7} nmr 6
170.27
170.04
169.80
169.57
169.33
147.46
138.43
126.01
121.31
87.25
86.85
86.44
86.04
85.63
79.20
79.13
79.05
77.31
77.06
76.81
31.35
27.91
25.68
24.53
3aa, 13C NMR, (126 MHz, CDCl3)
f1 (ppm)

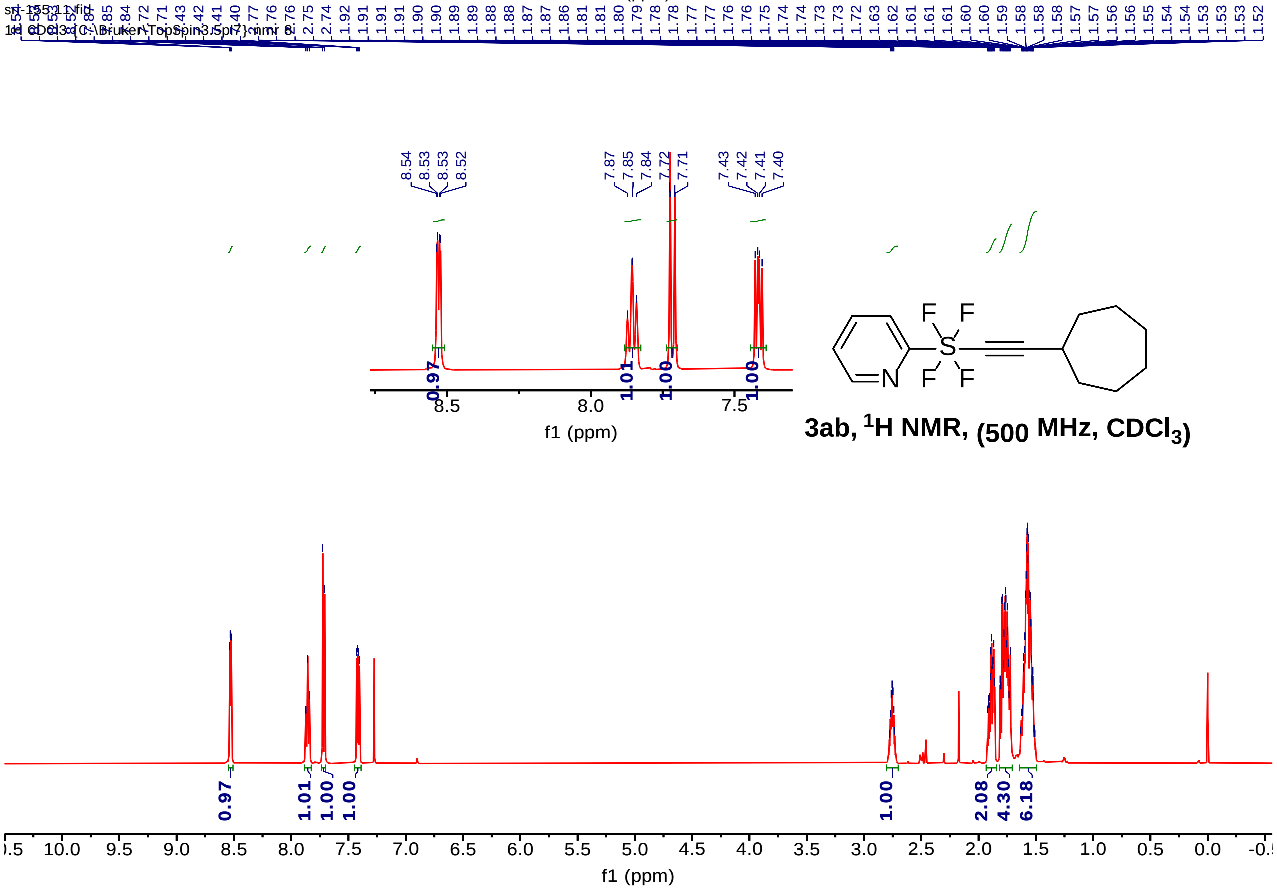
sri-155.11.fid
1H CDCl3 {C:\Bruker\TopSpin3.5pl7} nmr 8
8.54
8.53
8.53
8.52
7.87
7.85
7.84
7.72
7.71
7.43
7.42
7.41
7.40
3ab, 1H NMR, (500 MHz, CDCl3)
0.97
1.01
1.00
1.00
2.08
4.30
6.18
f1 (ppm)

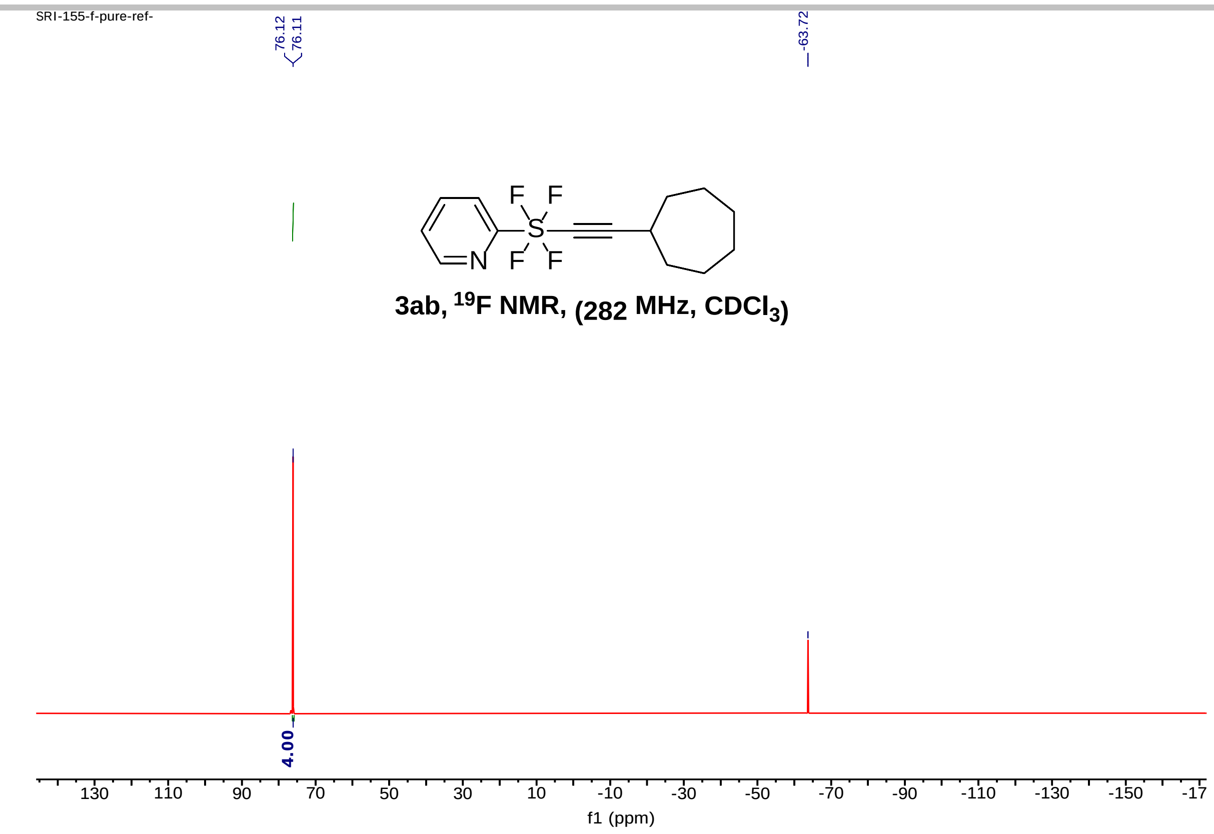

3ab, ^{19}F NMR, (282 MHz, $CDCl_3$)

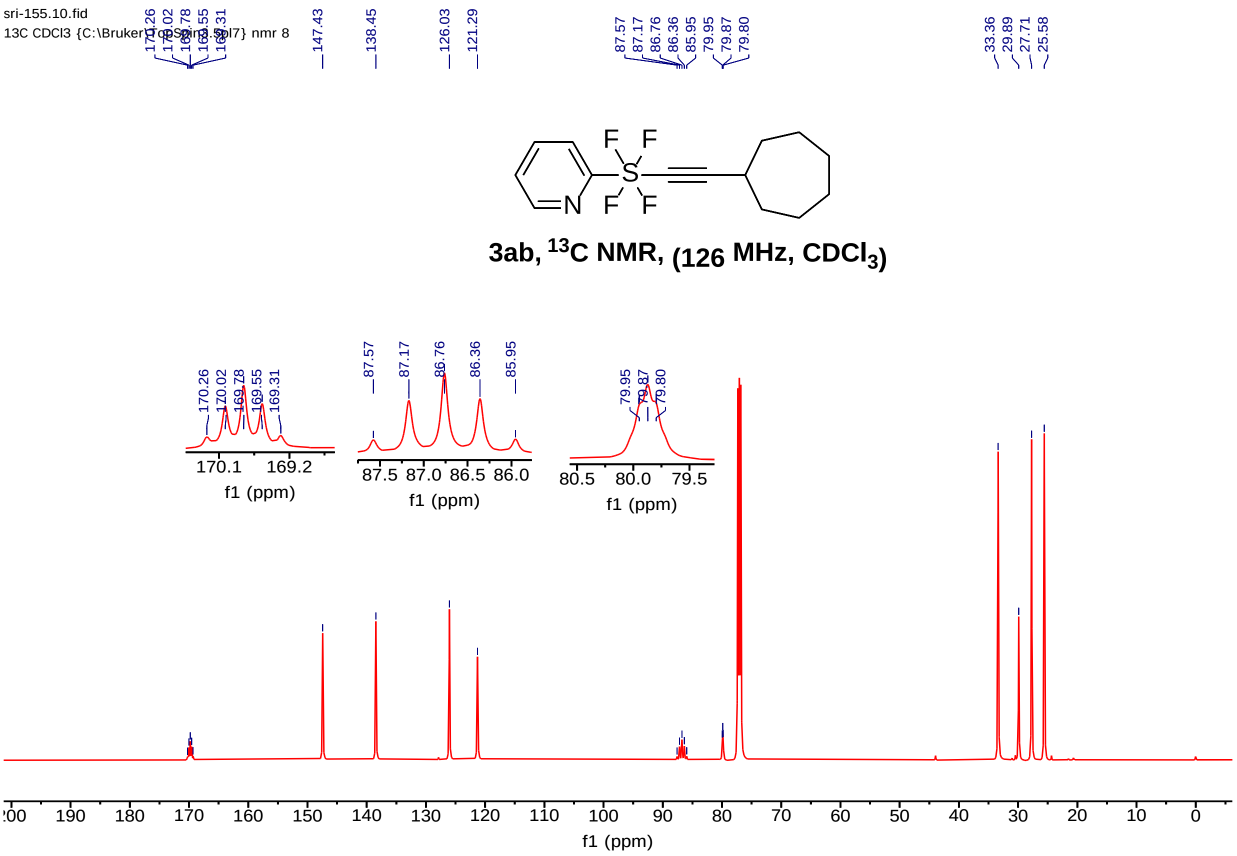

3ab, ^{13}C NMR, (126 MHz, $CDCl_3$)

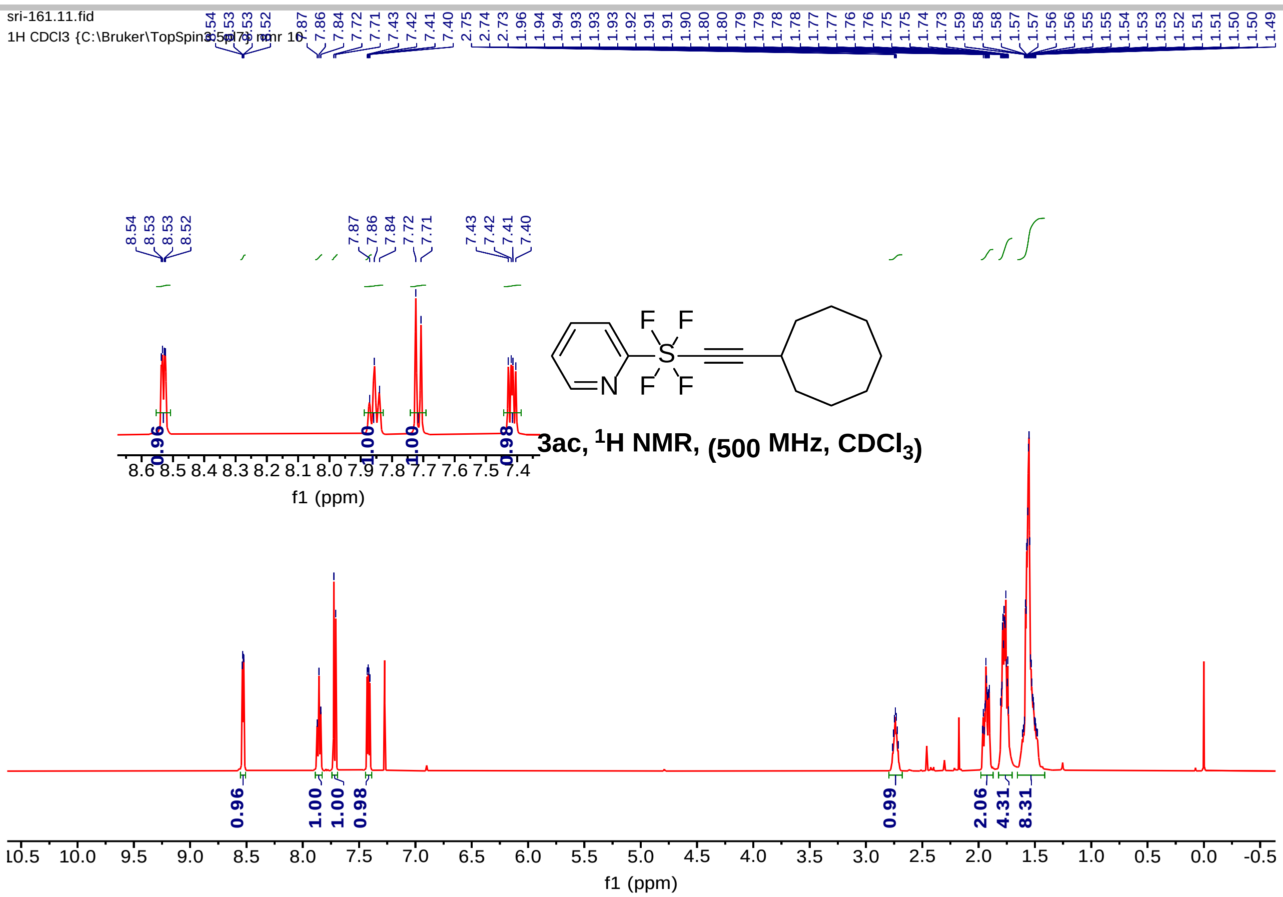

3ac, ^{1}H NMR, (500 MHz, $CDCl_3$)

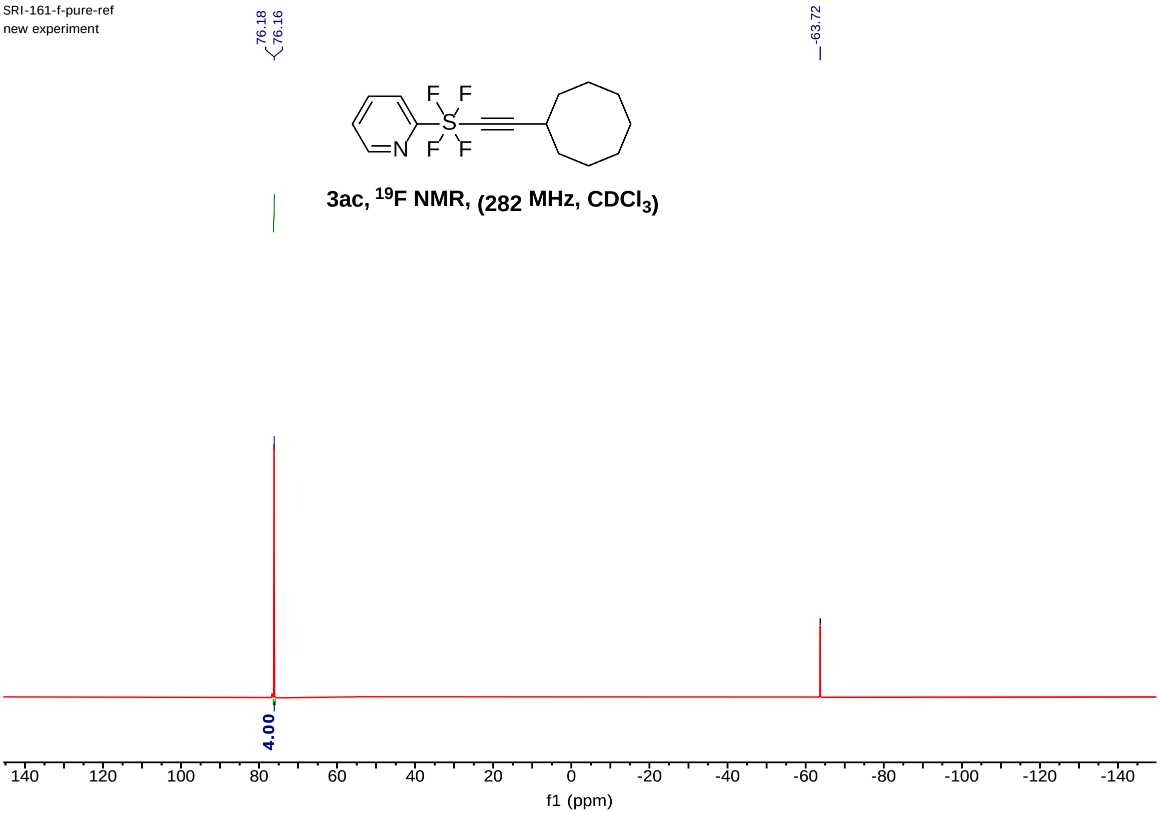

3ac, ^{19}F NMR, (282 MHz, $CDCl_3$)

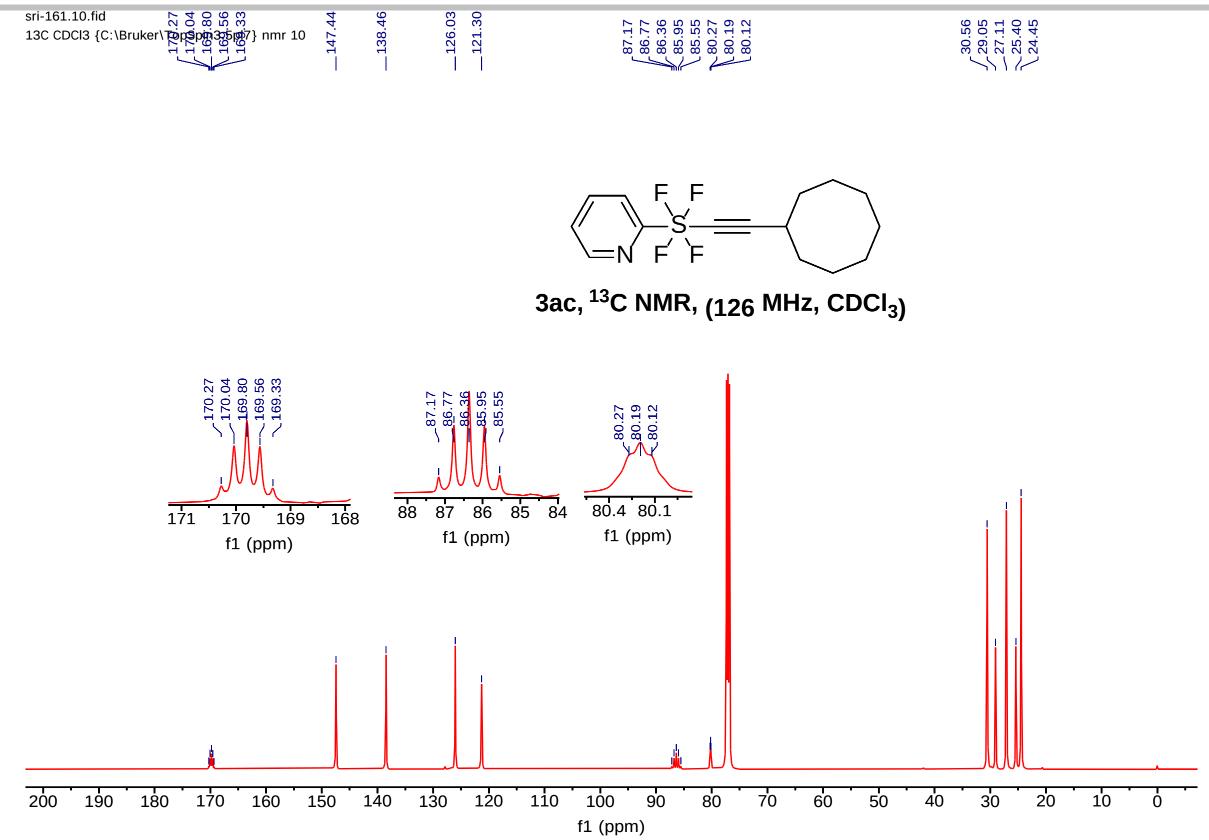

3ac, ^{13}C NMR, (126 MHz, $CDCl_3$)

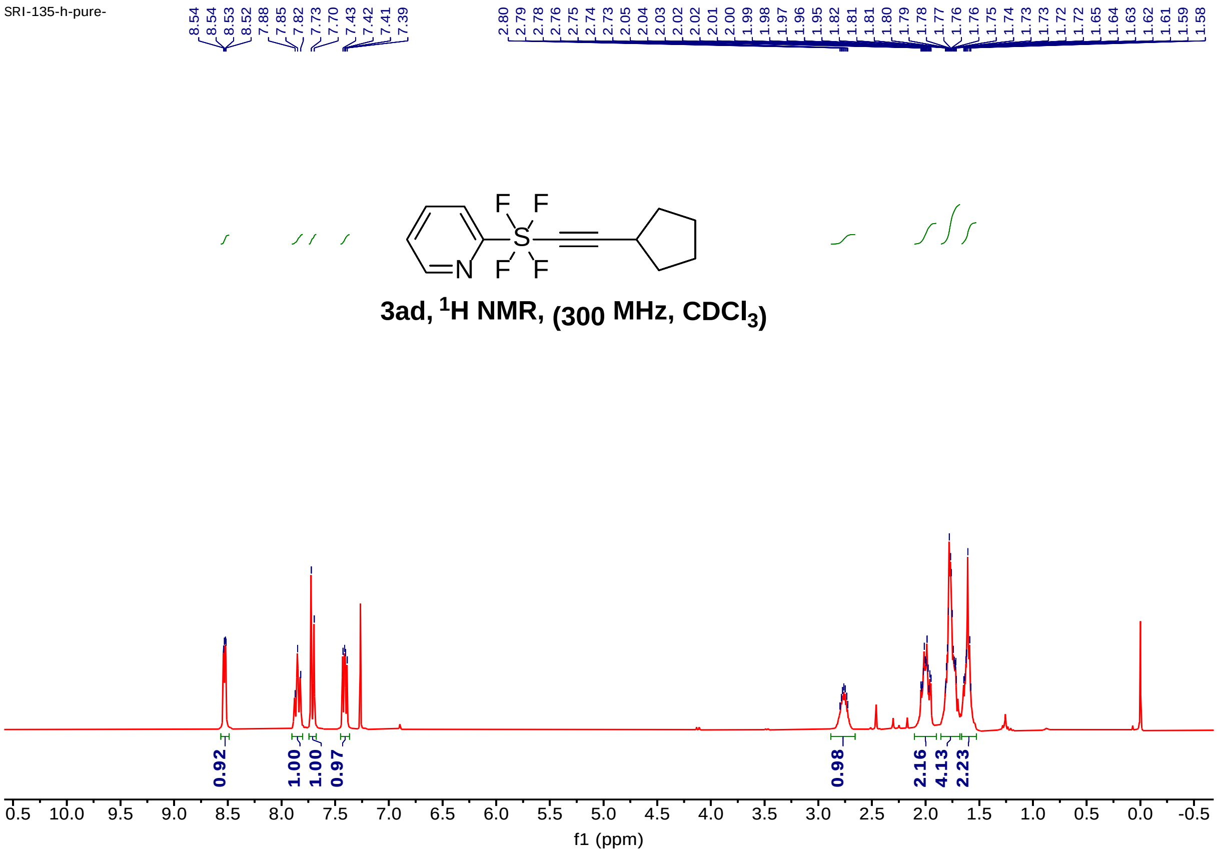

3ad, ^{1}H NMR, (300 MHz, $CDCl_3$)

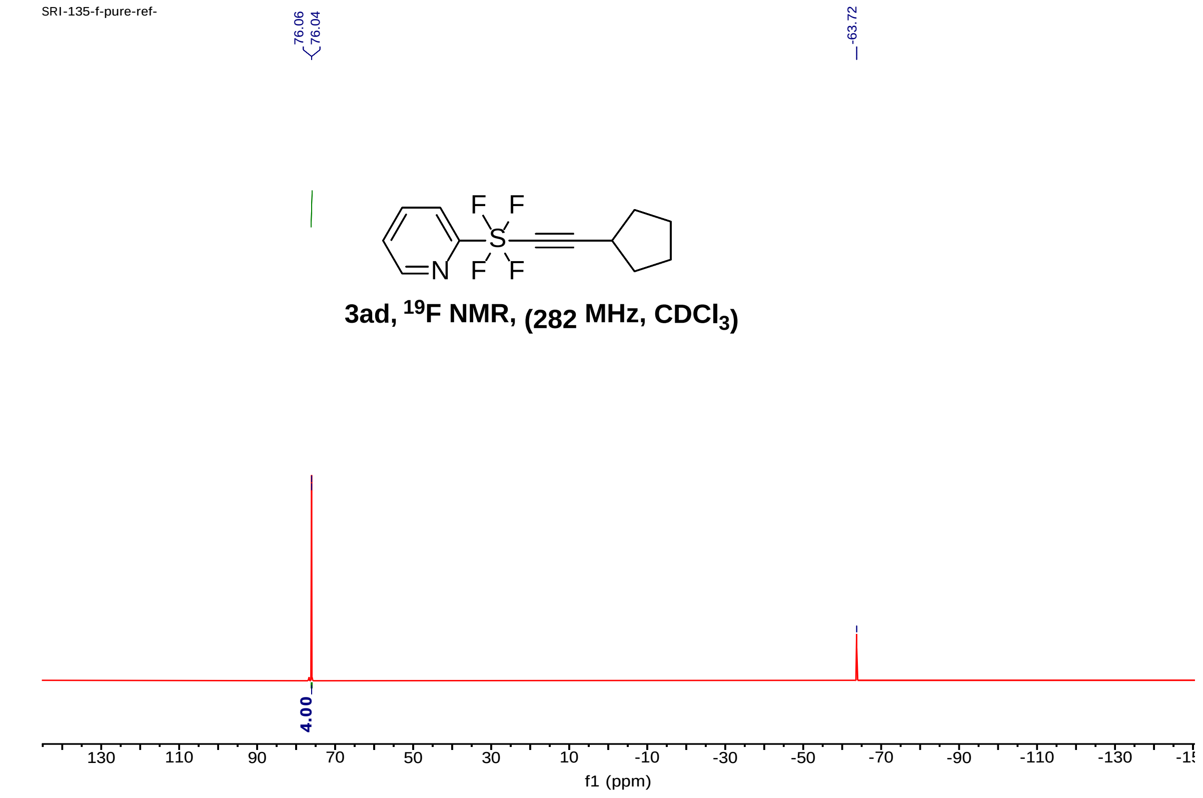

3ad, ^{19}F NMR, (282 MHz, $CDCl_3$)

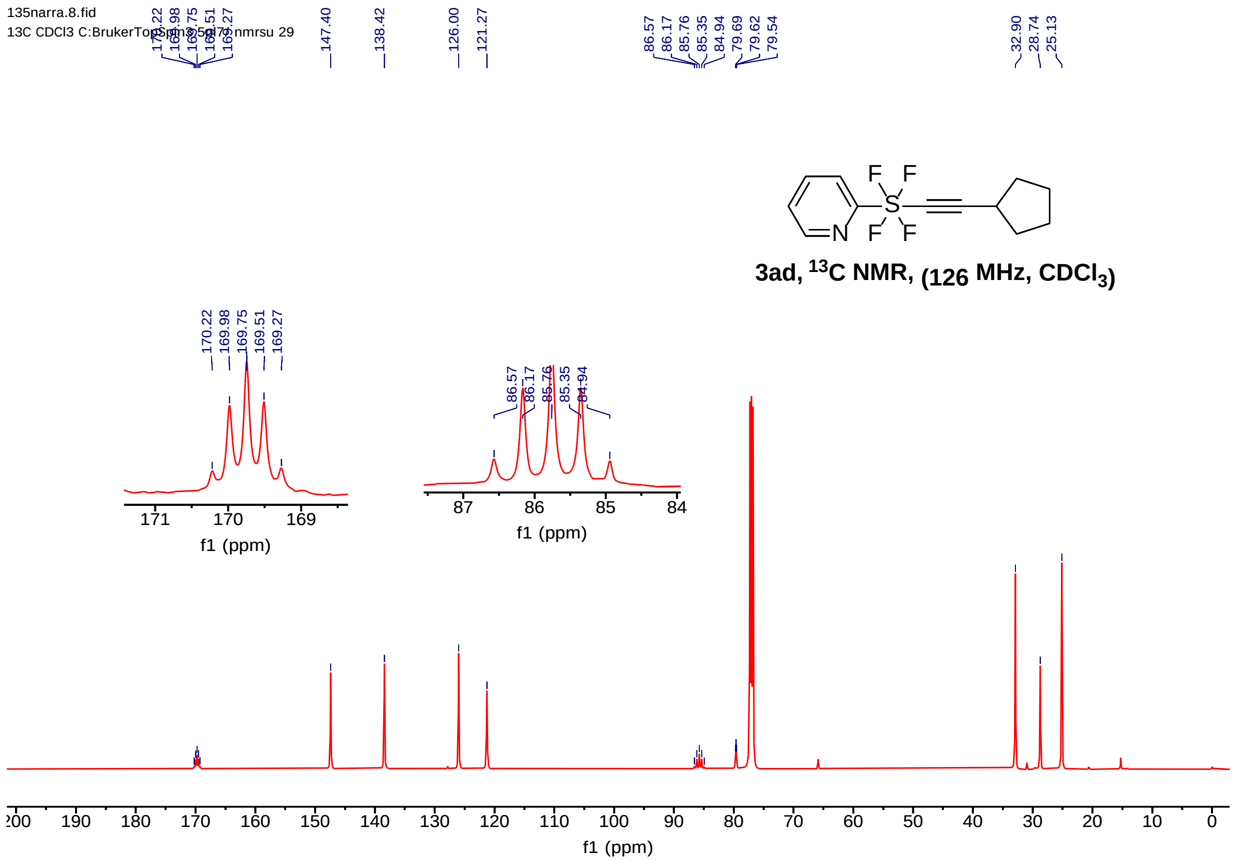

3ad, ^{13}C NMR, (126 MHz, $CDCl_3$)

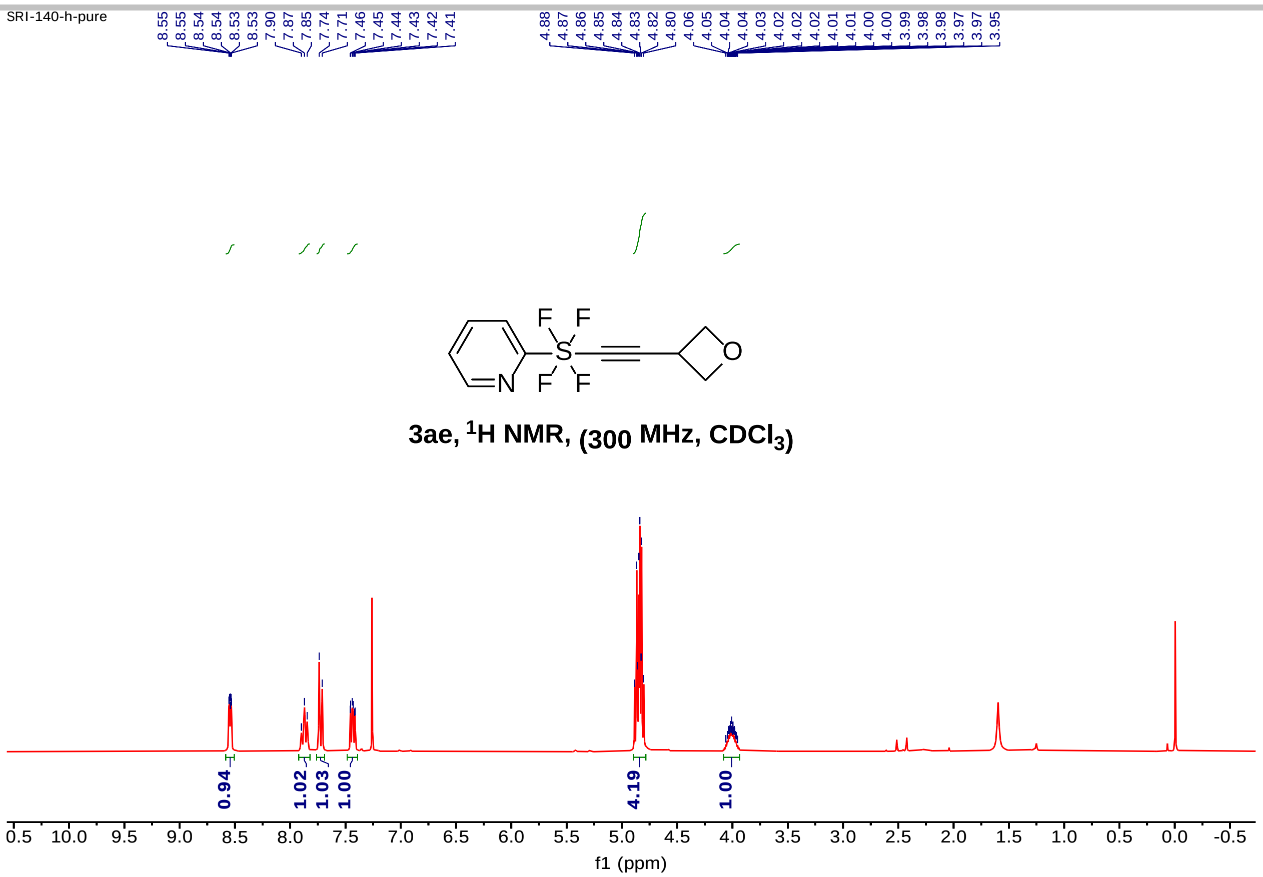

3ae, ^{1}H NMR, (300 MHz, $CDCl_3$)

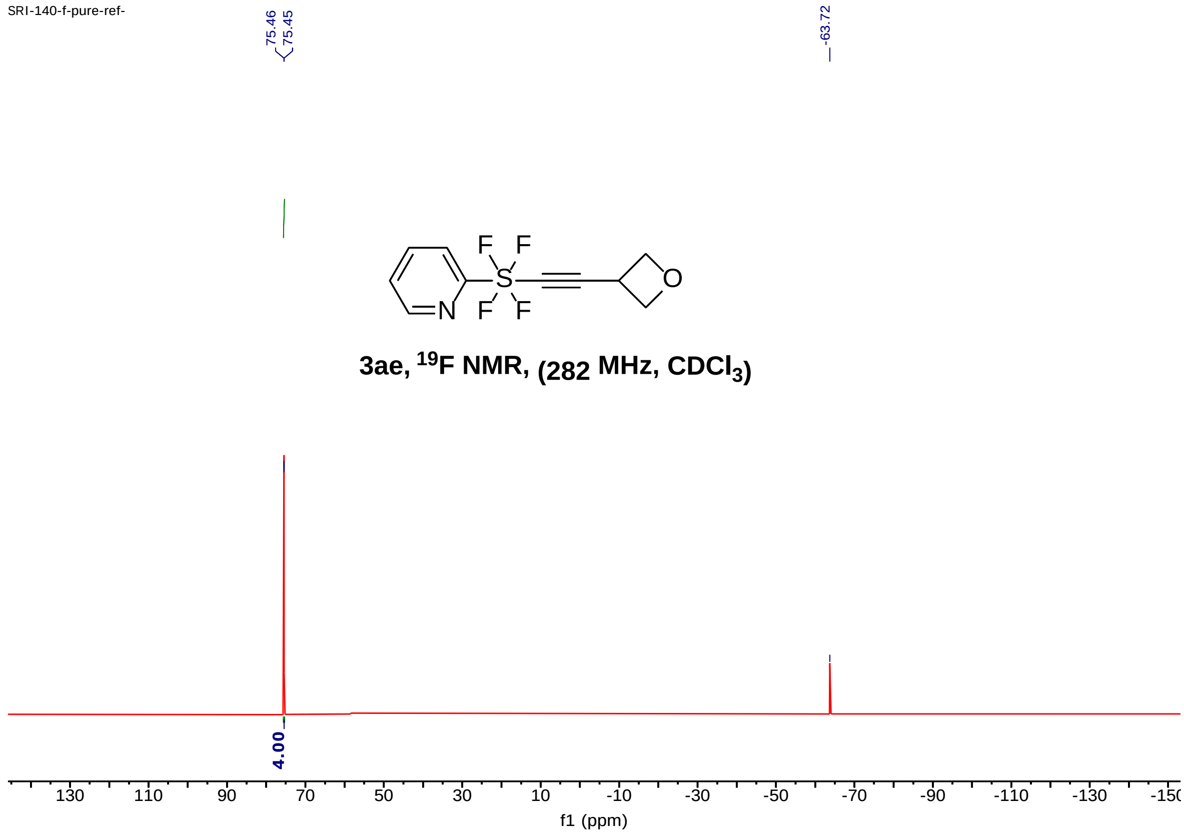

3ae, ^{19}F NMR, (282 MHz, $CDCl_3$)

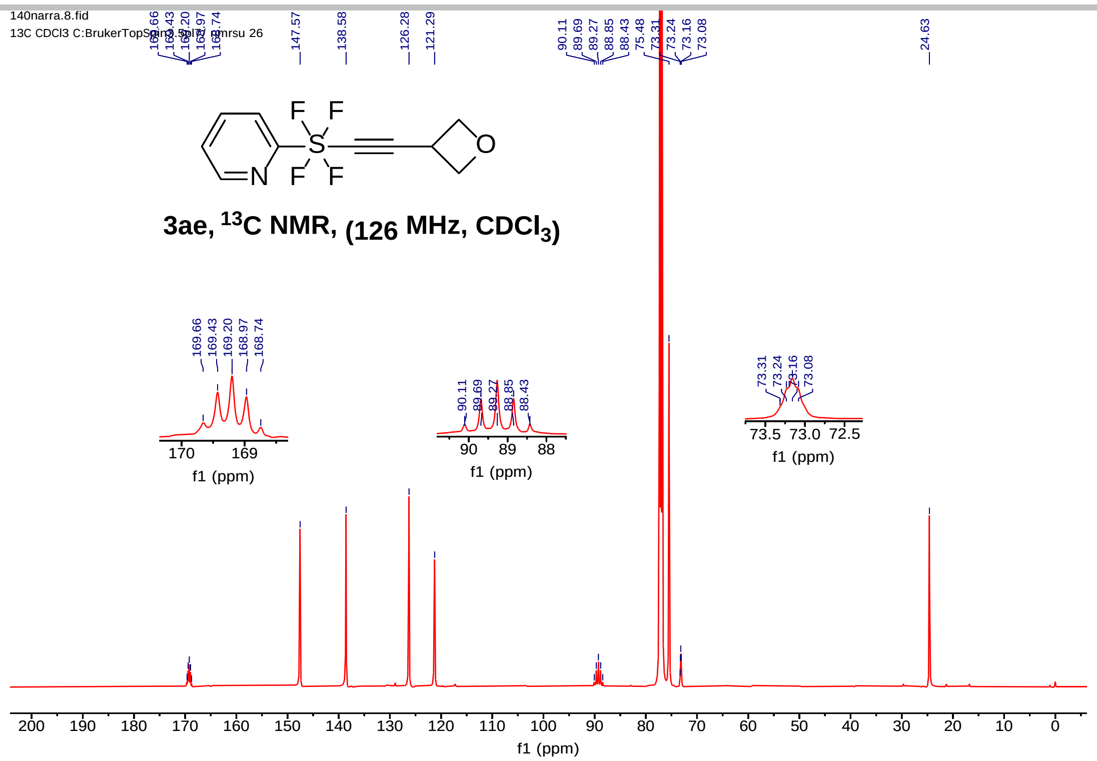
140narra.8.fid
13C CDCl3 C:BrukerTopSpin3.5pl7 nmrsu 26
3ae, ^{13}C NMR, (126 MHz, CDCl$_3$)
f1 (ppm)

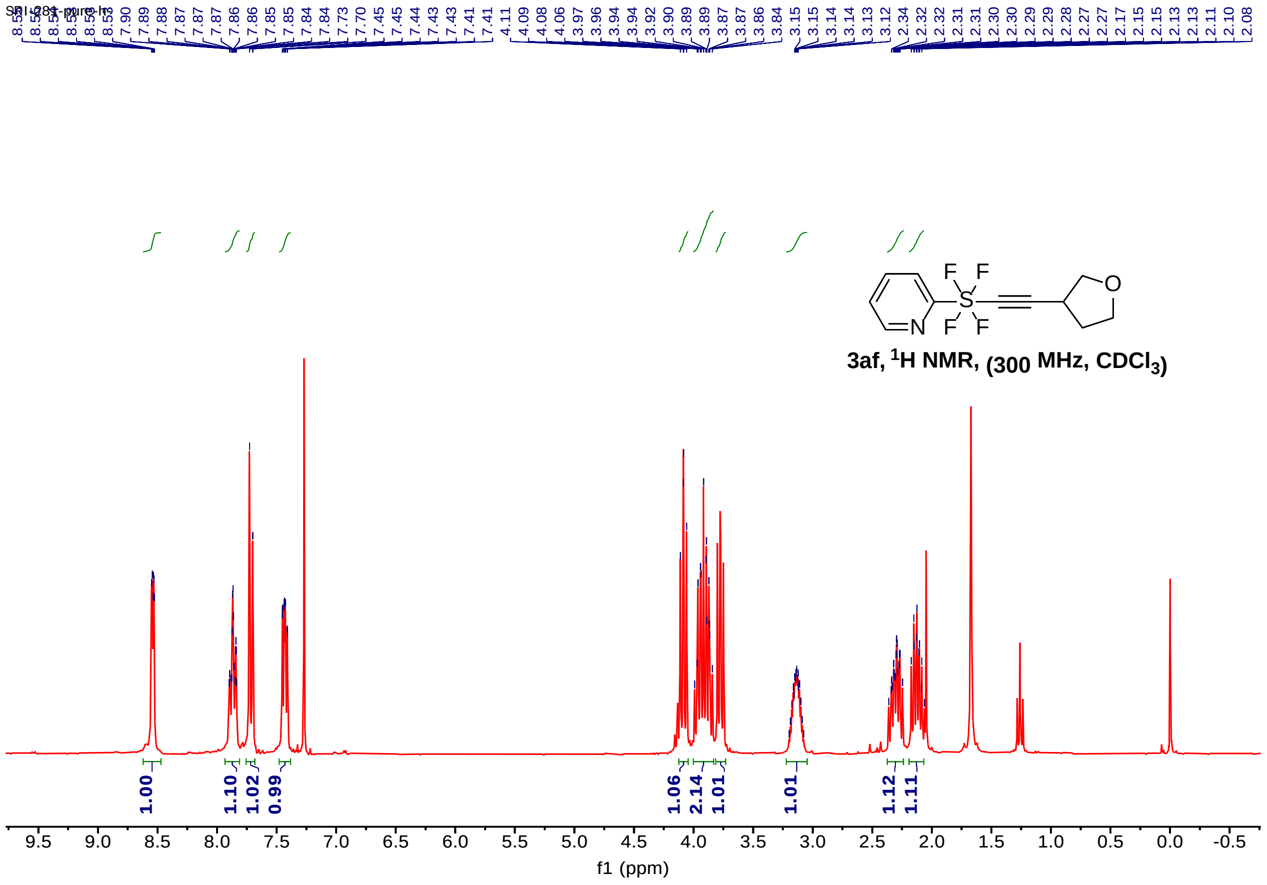
3af, ^{1}H NMR, (300 MHz, CDCl$_3$)
1.00
1.10
1.02
0.99
1.06
2.14
1.01
1.01
1.12
1.11
f1 (ppm)

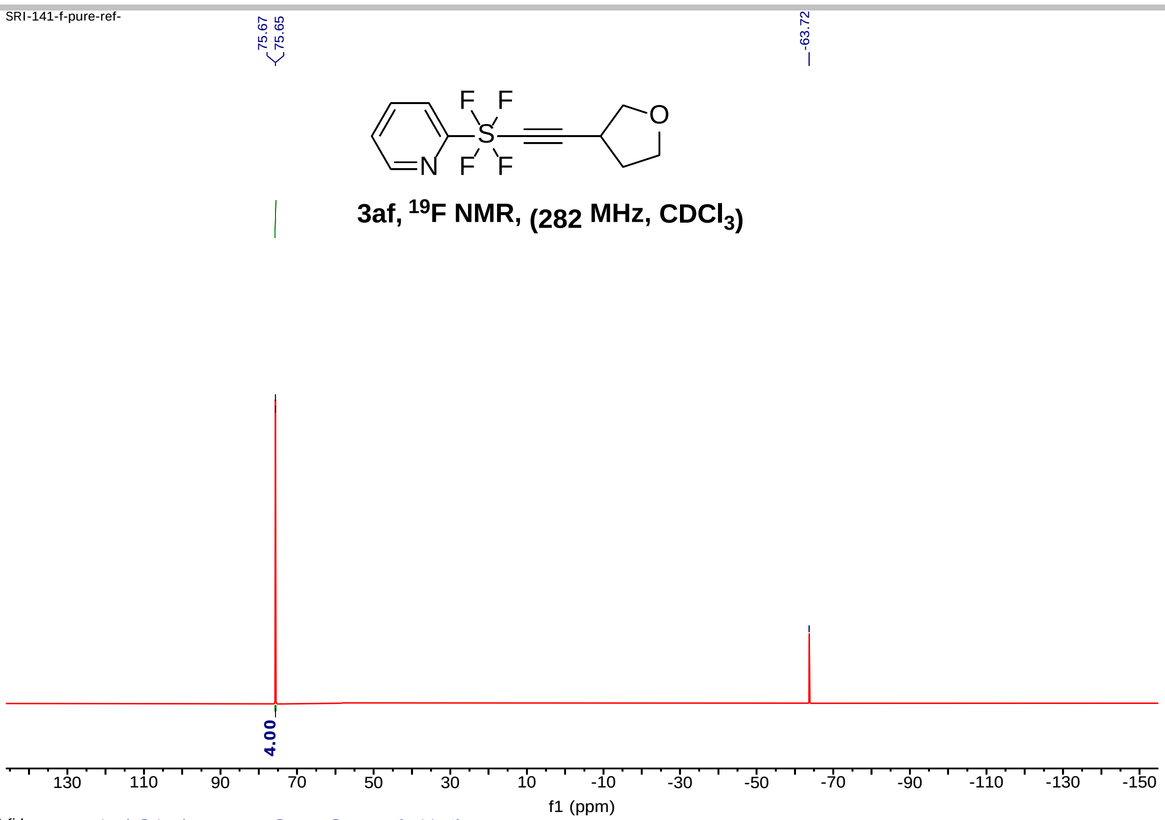
SRI-141-f-pure-ref-
-75.67
-75.65
-63.72
3af, ¹⁹F NMR, (282 MHz, CDCl3)
4.00
f1 (ppm)

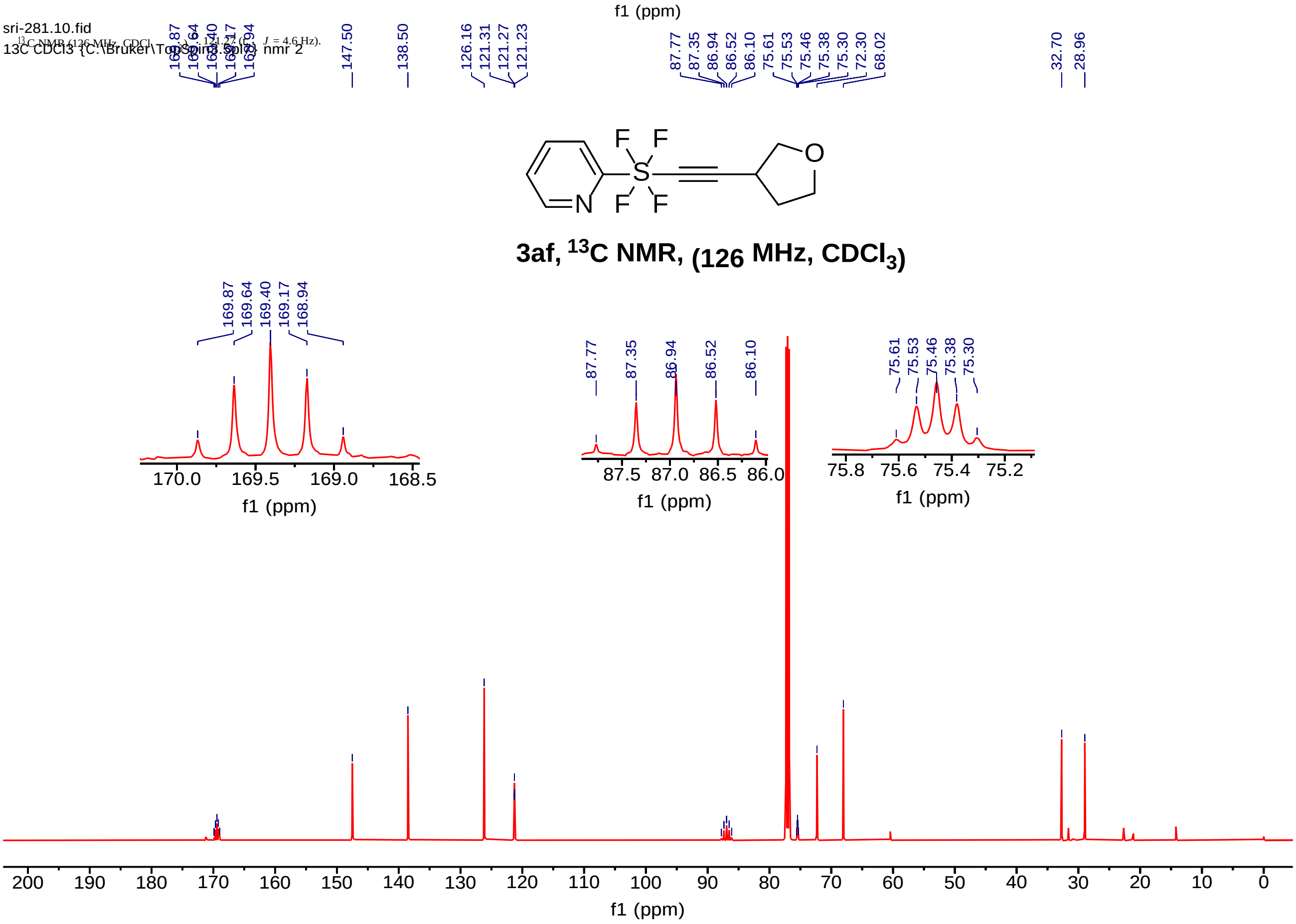
sri-281.10.fid
13C CDCl3 {C:\Bruker\TopSpin3.5pl2} nmr 2
169.87
169.64
169.40
169.17
168.94
147.50
138.50
126.16
121.31
121.27
121.23
87.77
87.35
86.94
86.52
86.10
75.61
75.53
75.46
75.38
75.30
72.30
68.02
32.70
28.96
3af, ¹³C NMR, (126 MHz, CDCl3)
f1 (ppm)

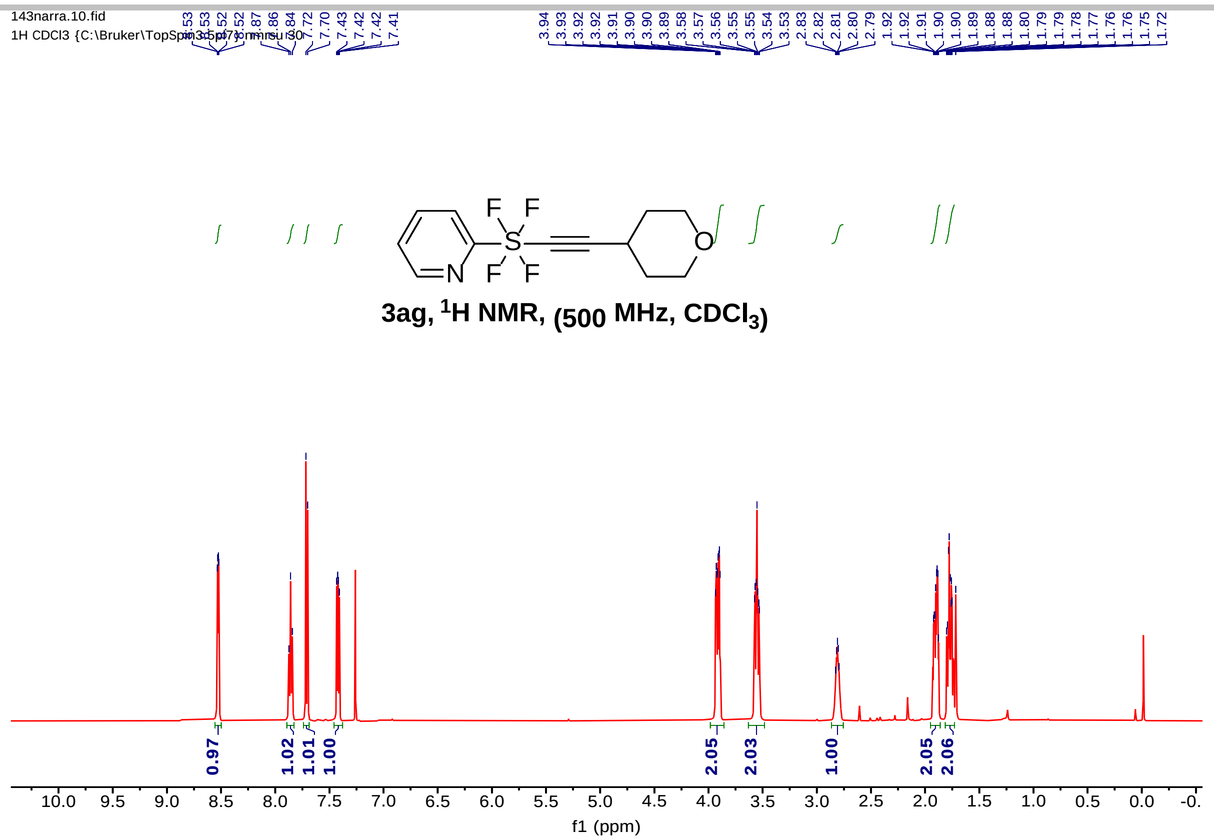

3ag, ^{1}H NMR, (500 MHz, $CDCl_3$)

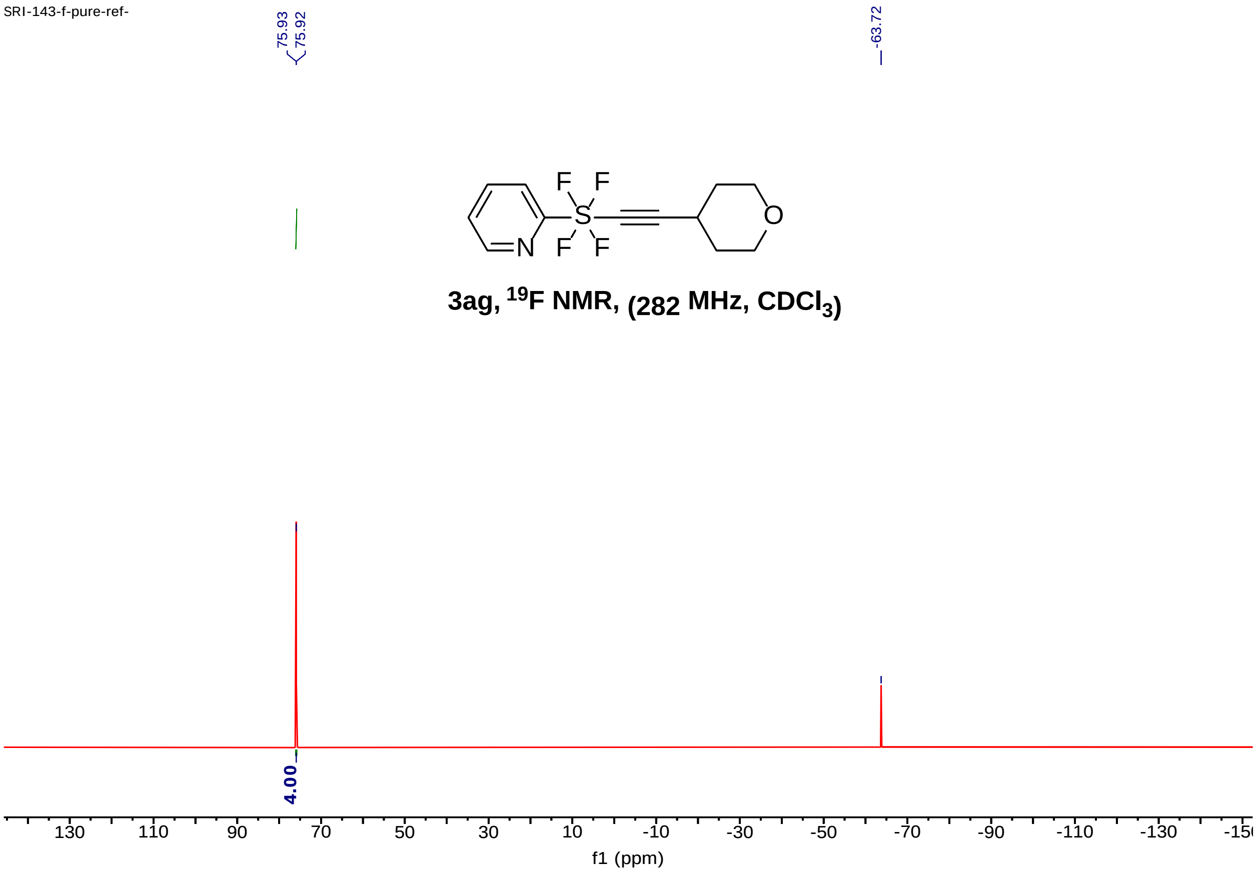

3ag, ^{19}F NMR, (282 MHz, $CDCl_3$)

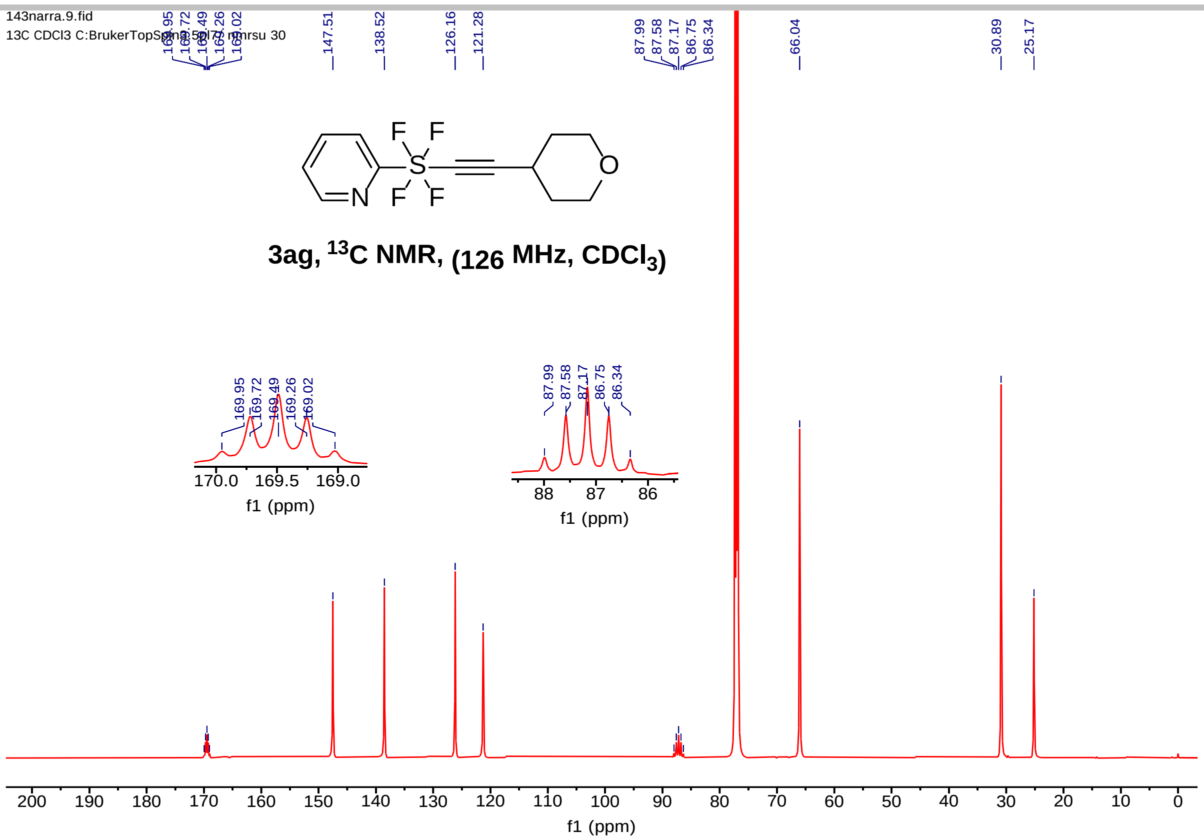
143narra.9.fid
13C CDCl3 C:BrukerTopSpin3.5pl7 nmrsu 30
3ag, 13C NMR, (126 MHz, CDCl3)
f1 (ppm)

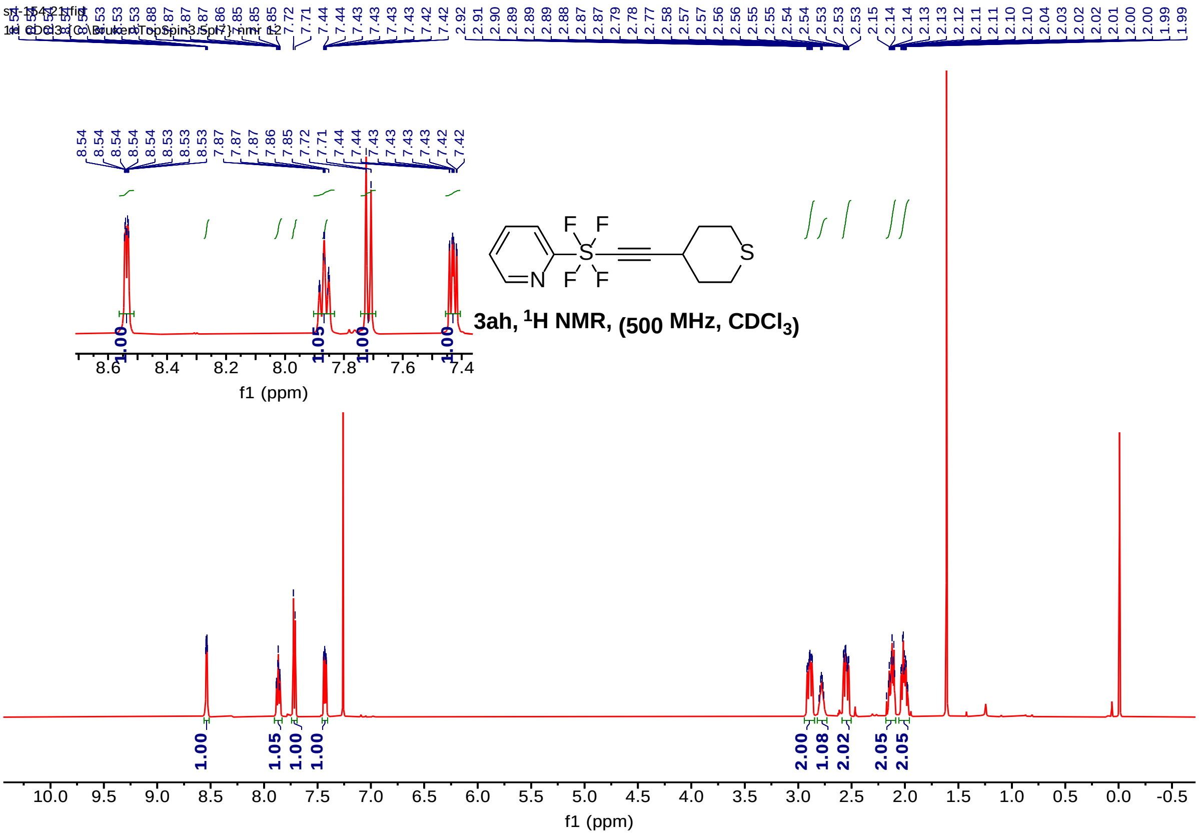
3ah, 1H NMR, (500 MHz, CDCl3)
f1 (ppm)

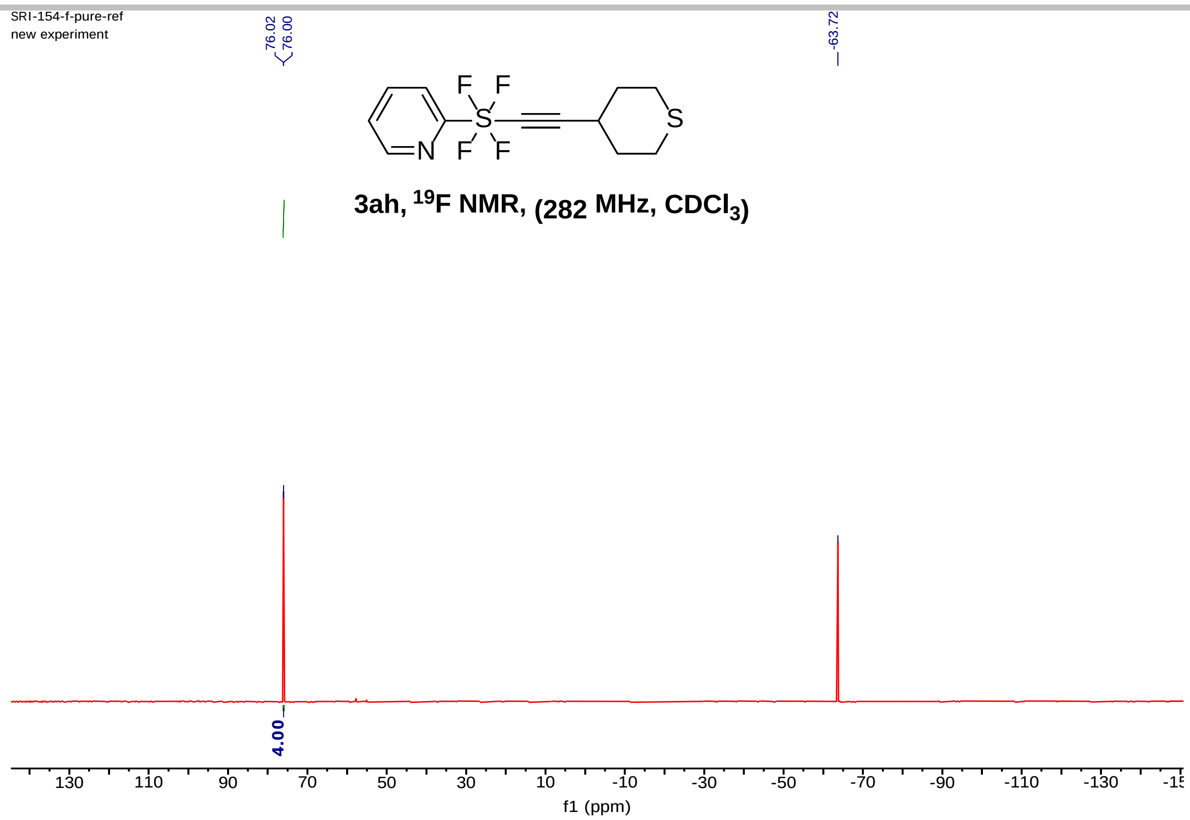
SRI-154-f-pure-ref
new experiment
76.02
76.00
-63.72
3ah, 19F NMR, (282 MHz, CDCl3)
4.00
130 110 90 70 50 30 10 -10 -30 -50 -70 -90 -110 -130
f1 (ppm)

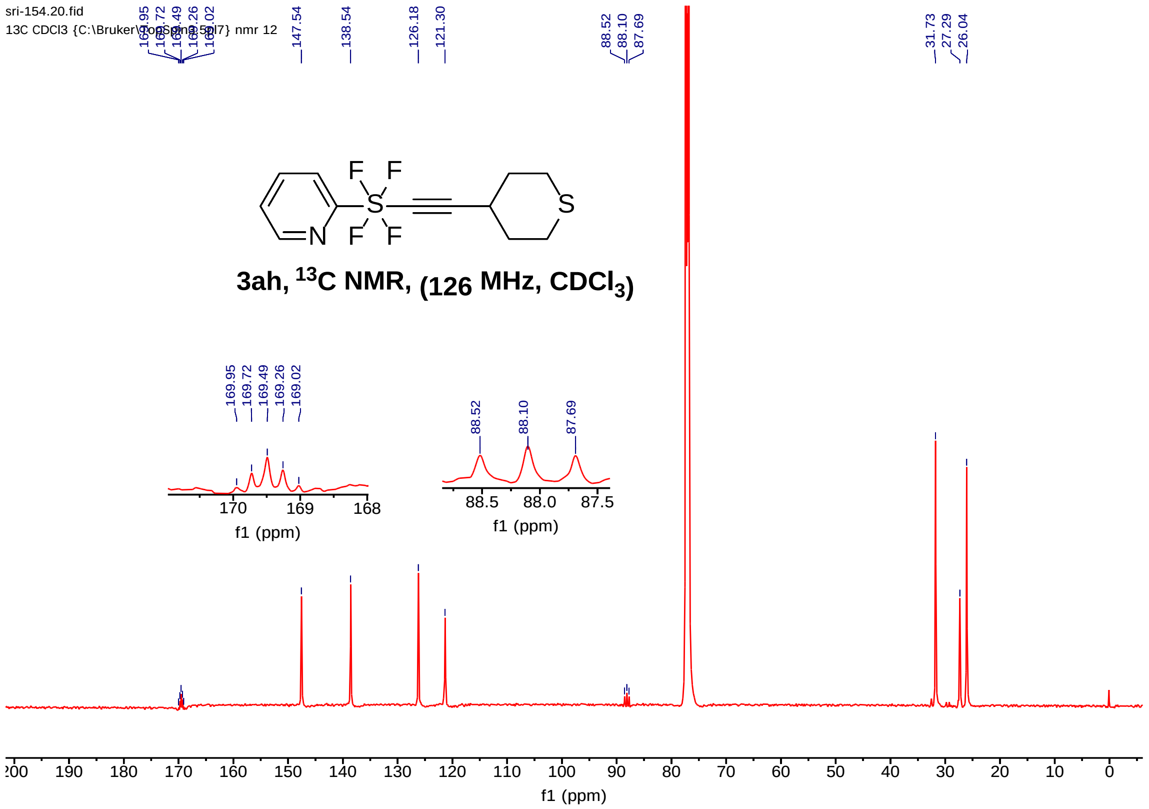
sri-154.20.fid
13C CDCl3 {C:\Bruker\TopSpin3.5pl7} nmr 12
169.95
169.72
169.49
169.26
169.02
147.54
138.54
126.18
121.30
88.52
88.10
87.69
31.73
27.29
26.04
3ah, 13C NMR, (126 MHz, CDCl3)
170 169 168
f1 (ppm)
88.5 88.0 87.5
f1 (ppm)
190 180 170 160 150 140 130 120 110 100 90 80 70 60 50 40 30 20 10 0
f1 (ppm)

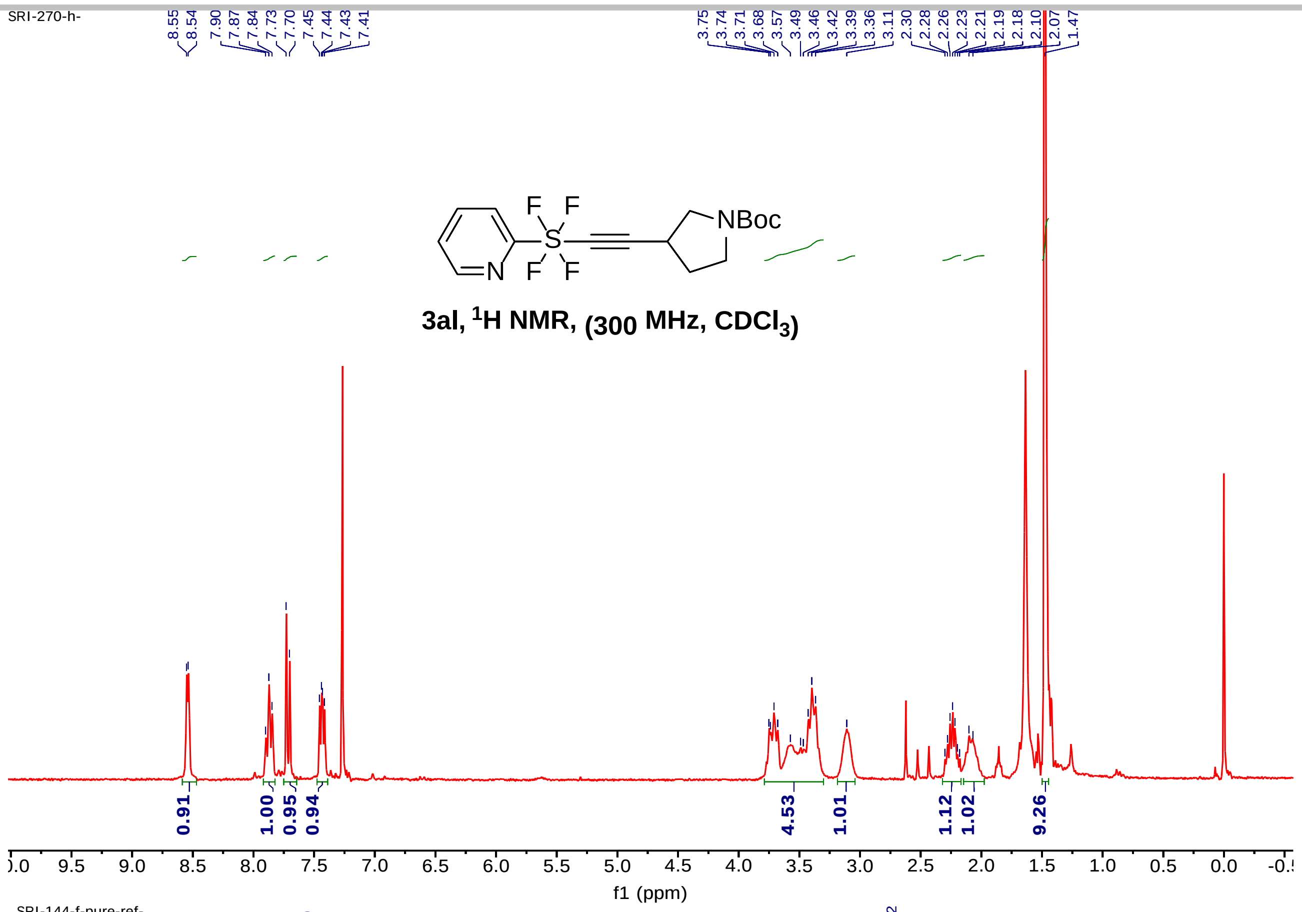

3al, ^{1}H NMR, (300 MHz, $CDCl_3$)

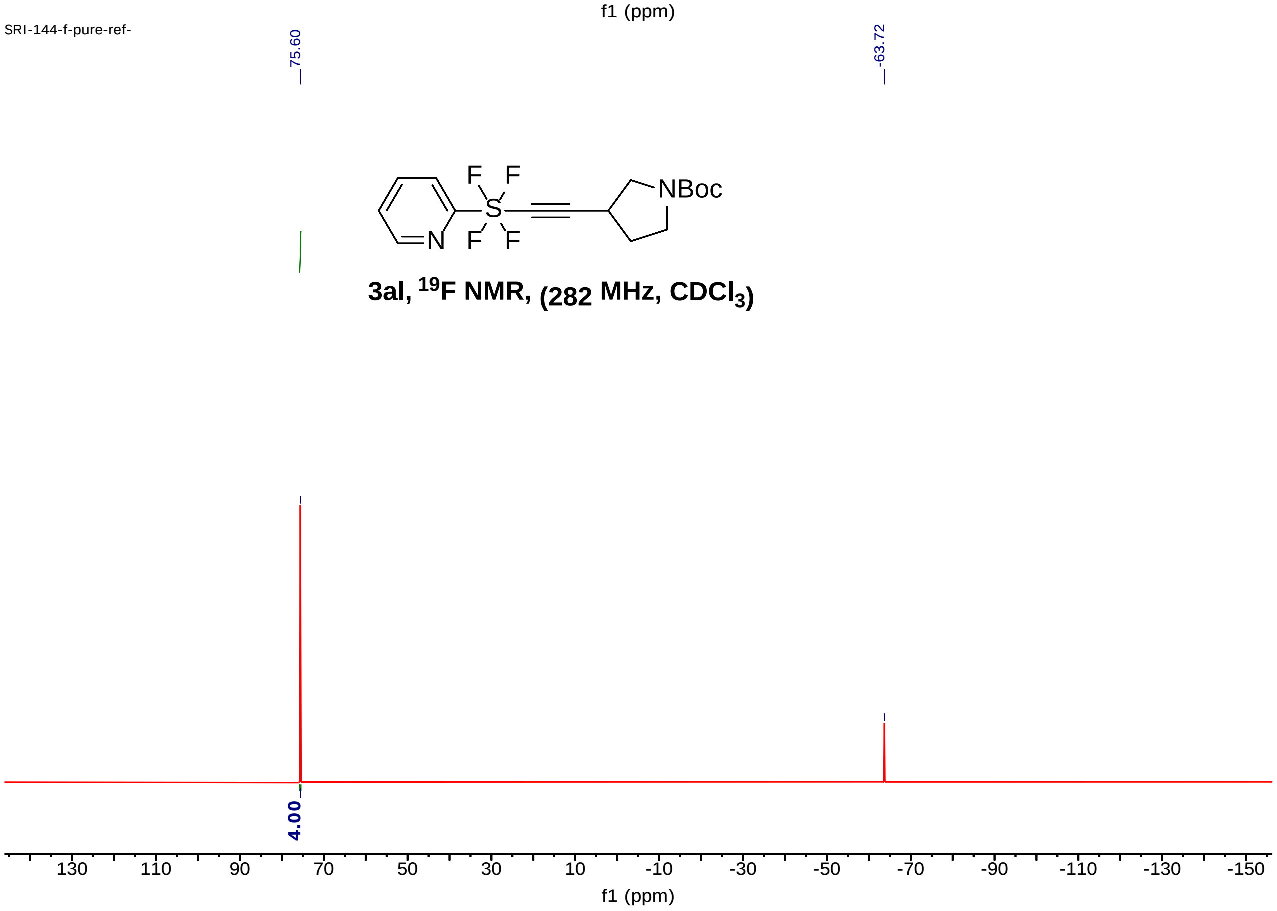

3al, ^{19}F NMR, (282 MHz, $CDCl_3$)

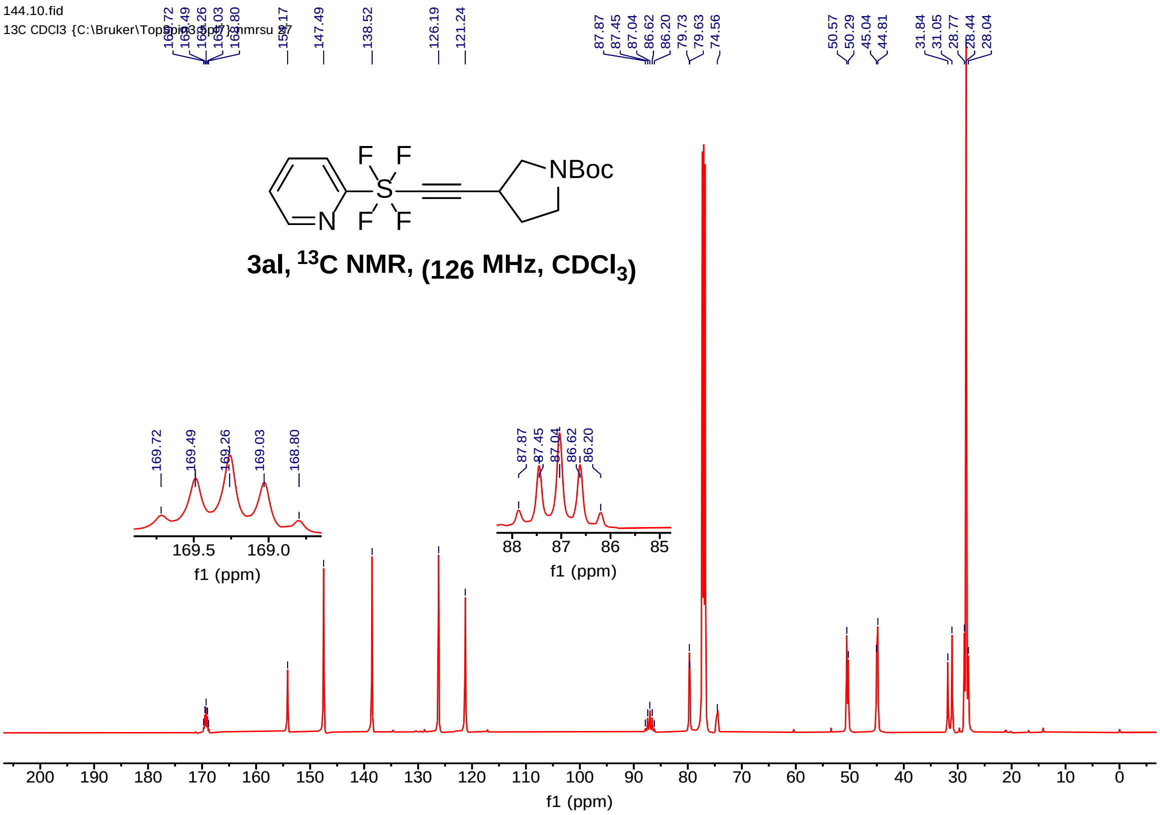

3al, ^{13}C NMR, (126 MHz, $CDCl_3$)

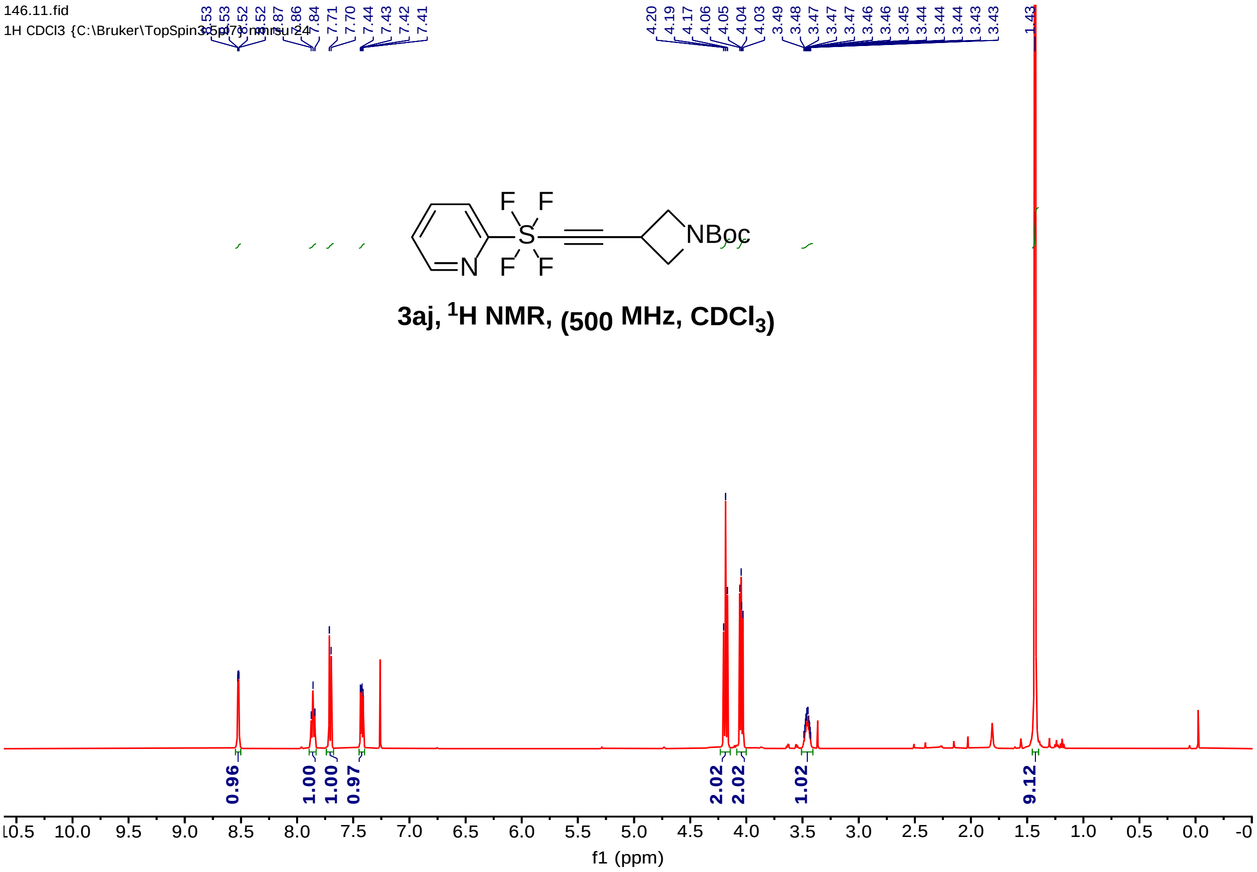

3aj, ^{1}H NMR, (500 MHz, $CDCl_3$)

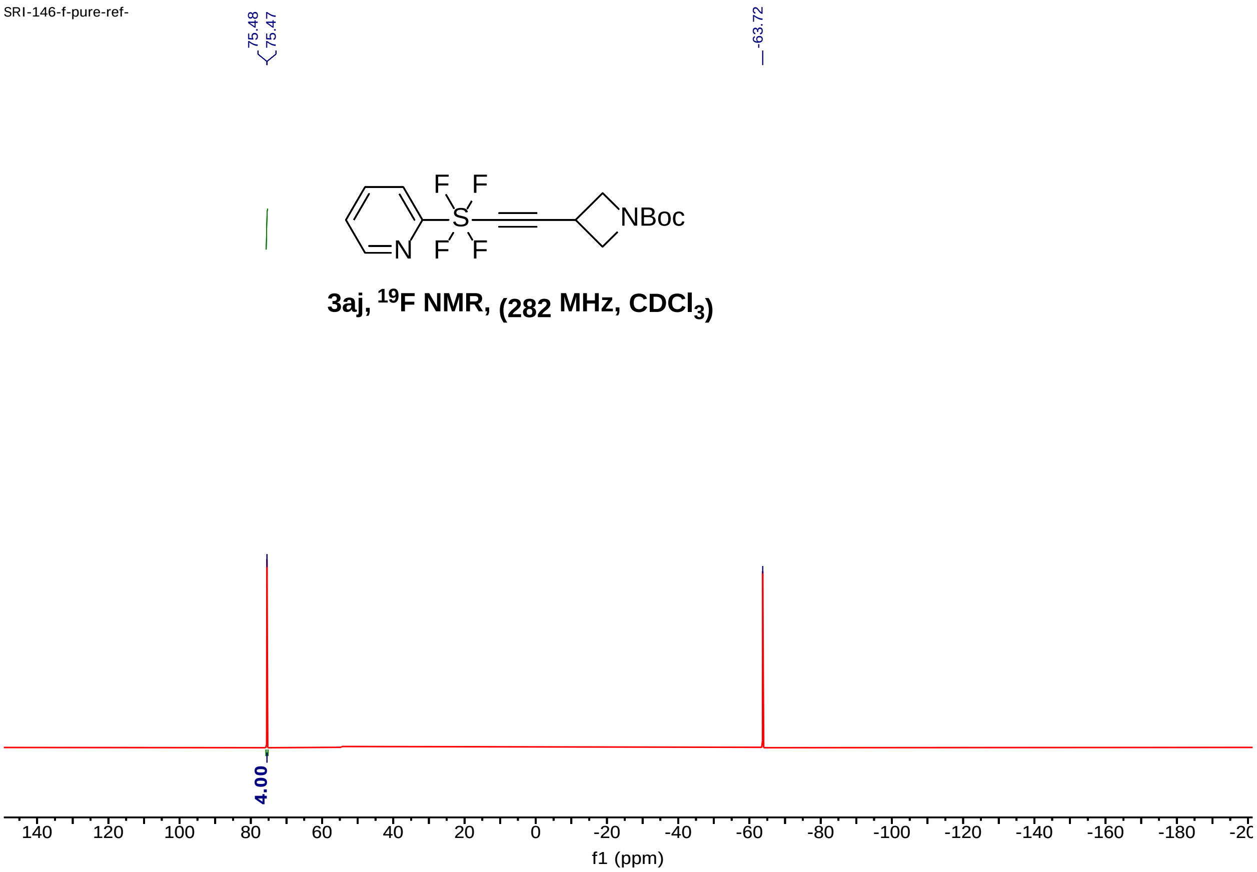

3aj, ^{19}F NMR, (282 MHz, $CDCl_3$)

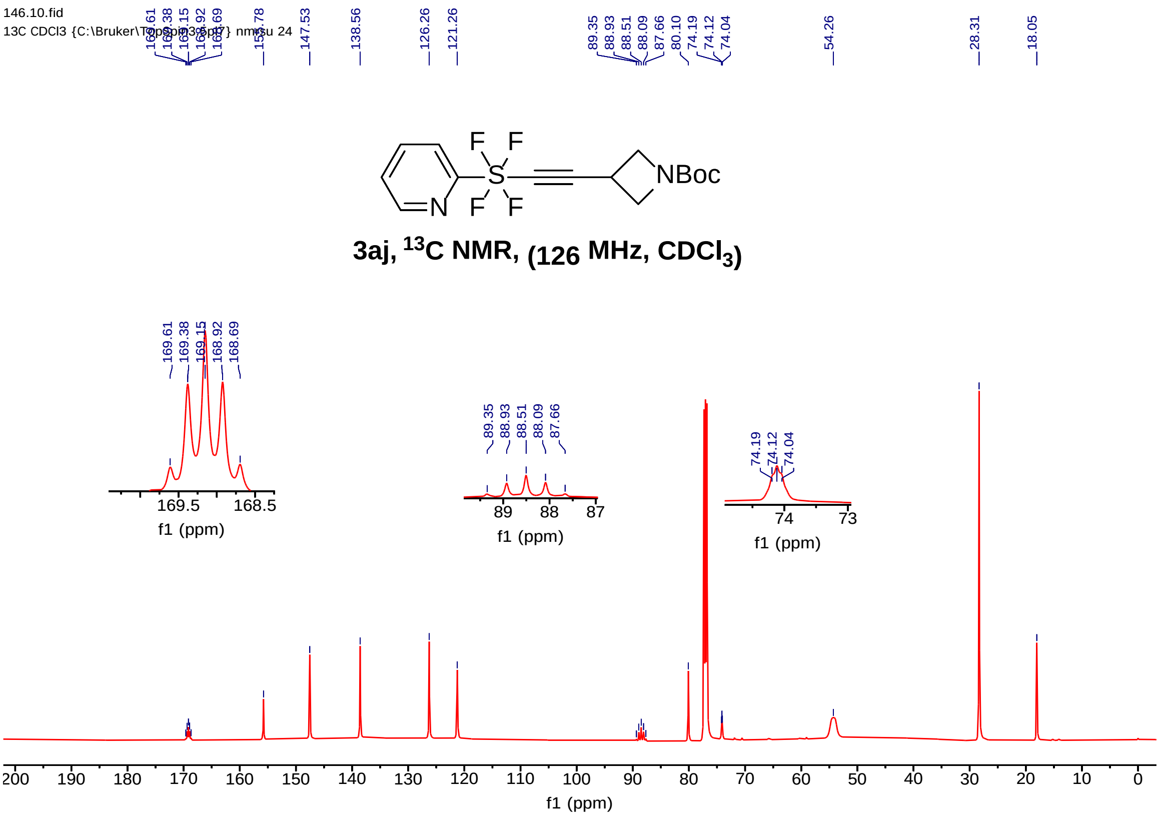

3aj, ^{13}C NMR, (126 MHz, $CDCl_3$)

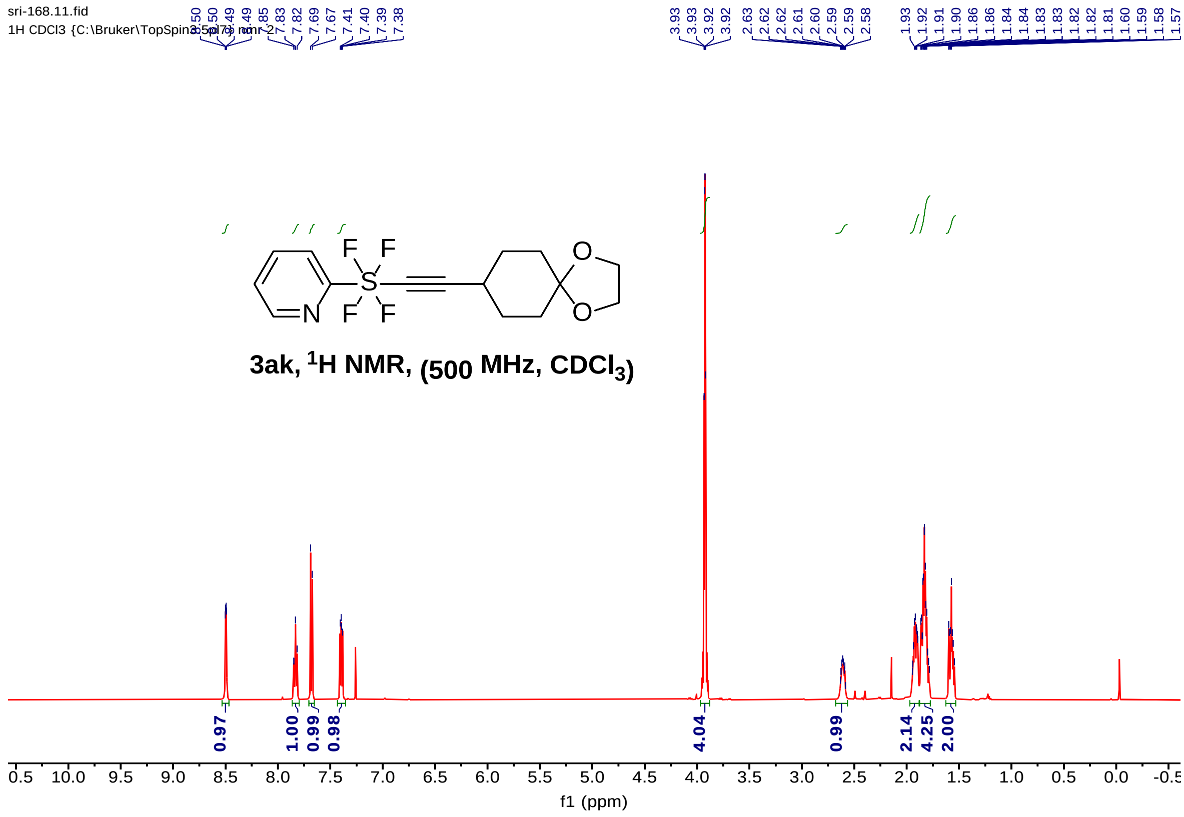
sri-168.11.fid
1H CDCl3 {C:\Bruker\TopSpin3.5pl7} nmr 2
3ak, 1H NMR, (500 MHz, CDCl3)
0.97
1.00
0.99
0.98
4.04
0.99
2.14
4.25
2.00
f1 (ppm)

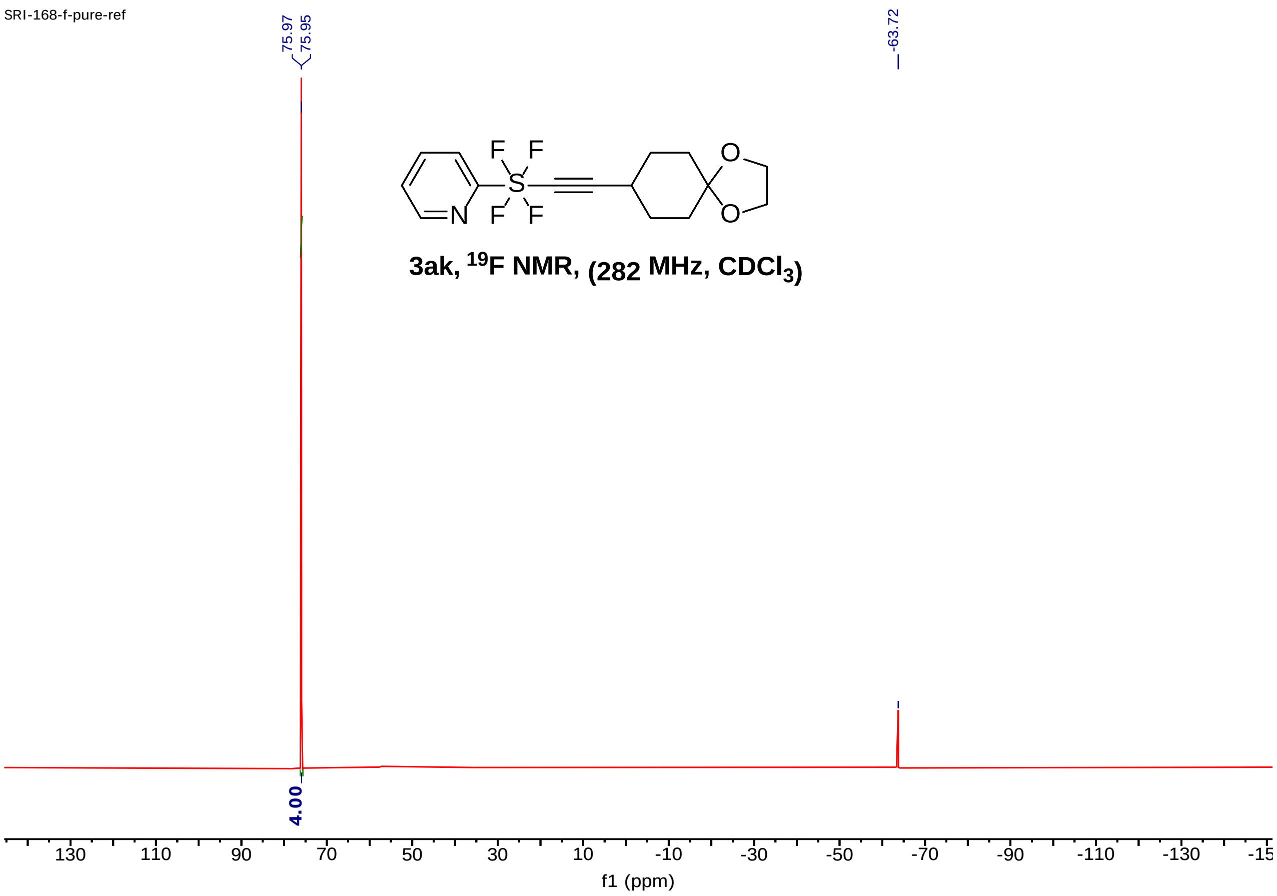
SRI-168-f-pure-ref
75.97
75.95
-63.72
3ak, 19F NMR, (282 MHz, CDCl3)
4.00
f1 (ppm)

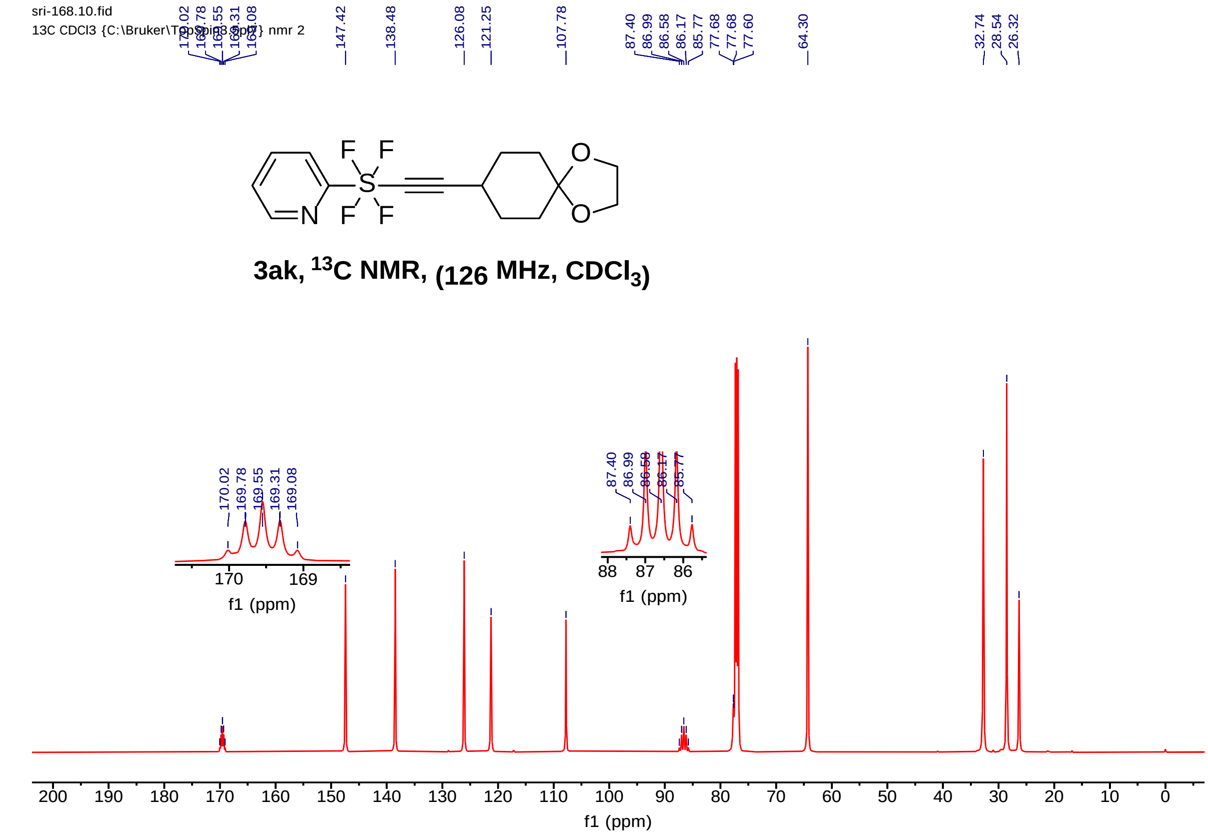

3ak, ^{13}C NMR, (126 MHz, $CDCl_3$)

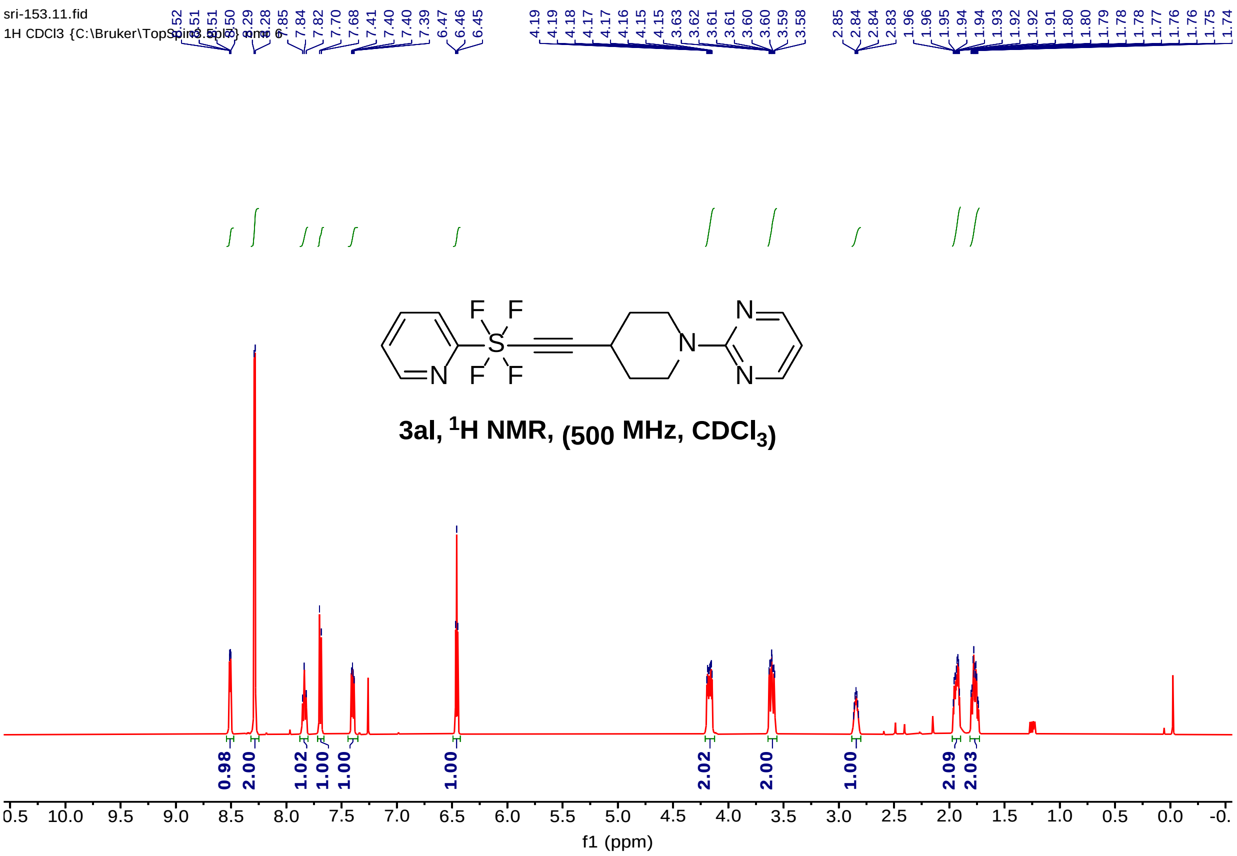

3al, ^{1}H NMR, (500 MHz, $CDCl_3$)

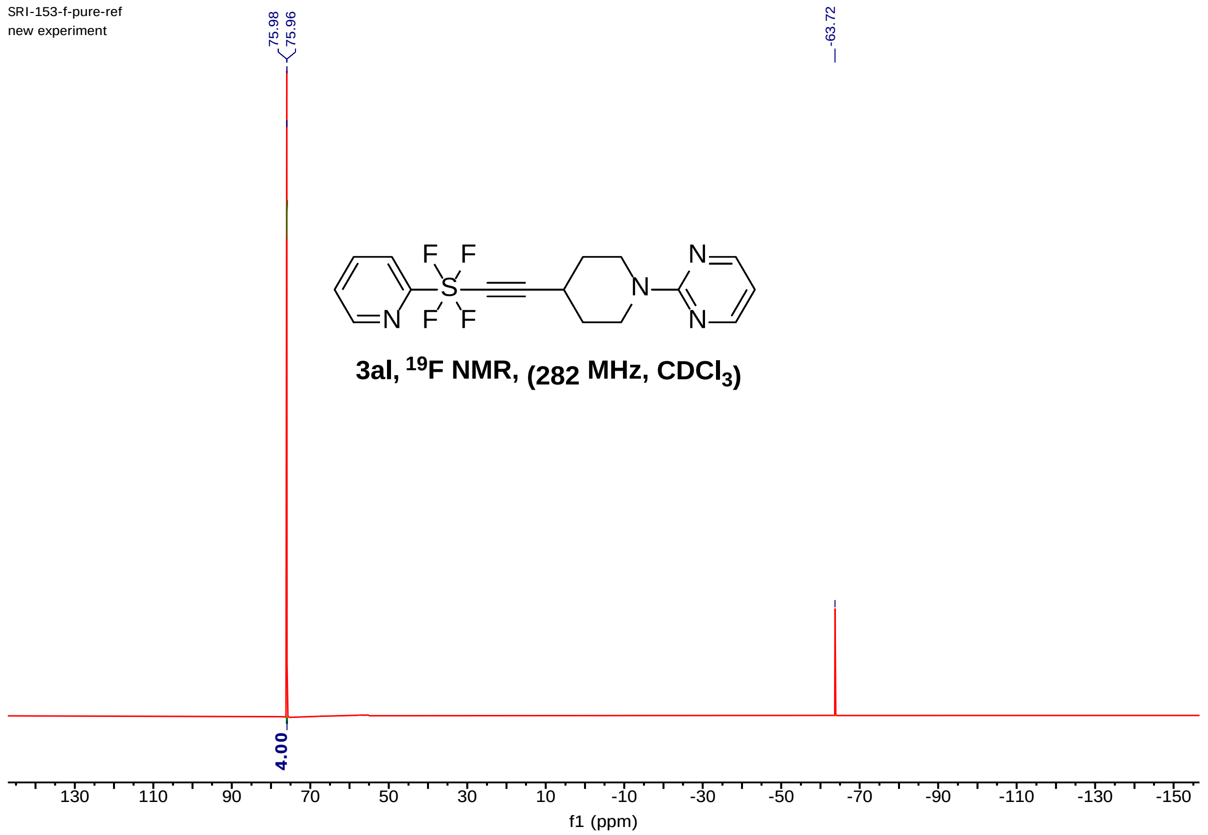
SRI-153-f-pure-ref
new experiment
75.98
75.96
-63.72
3al, ^{19}F NMR, (282 MHz, CDCl$_3$)
4.00
f1 (ppm)

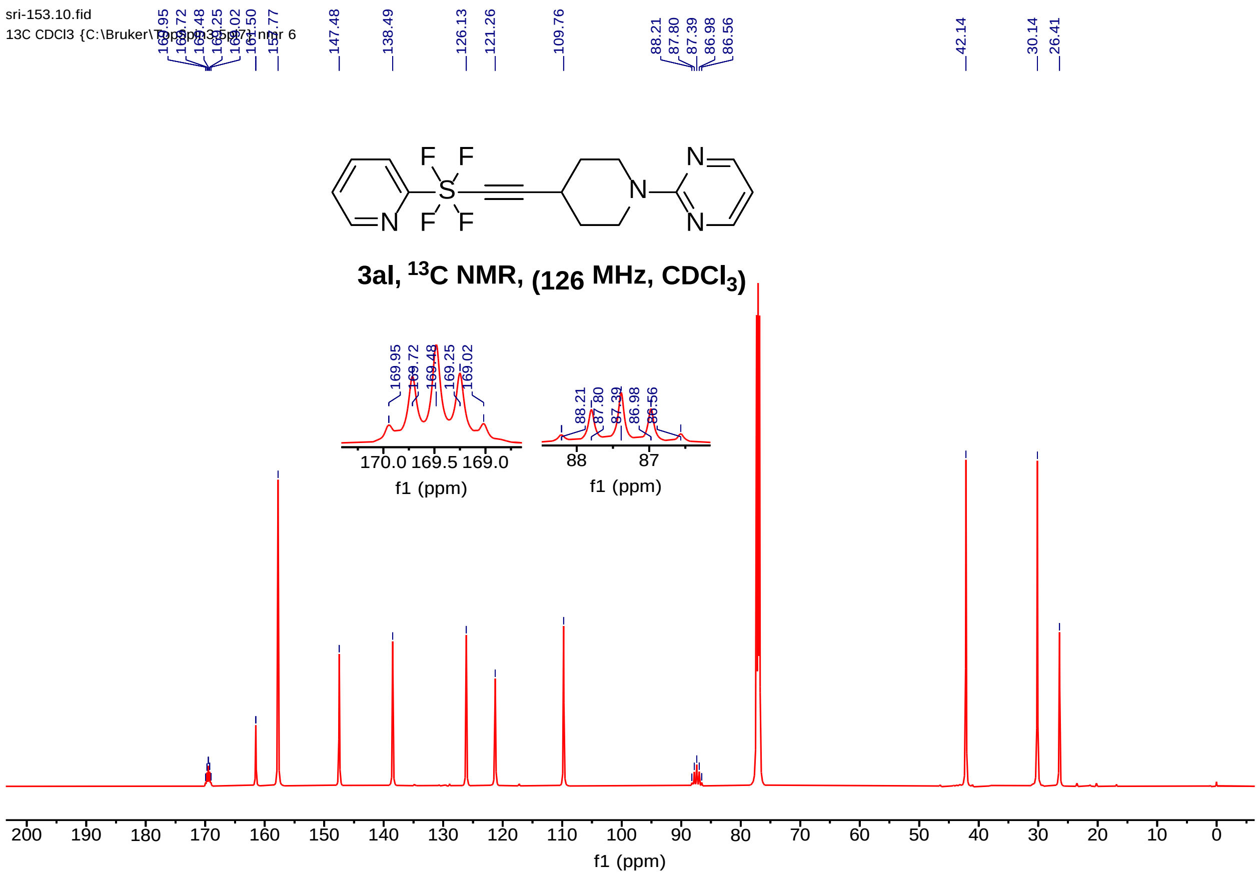
sri-153.10.fid
13C CDCl3 {C:\Bruker\TopSpin3.5pl7} nmr 6
169.95
169.72
169.48
169.25
169.02
161.50
157.77
147.48
138.49
126.13
121.26
109.76
88.21
87.80
87.39
86.98
86.56
42.14
30.14
26.41
3al, ^{13}C NMR, (126 MHz, CDCl$_3$)
f1 (ppm)

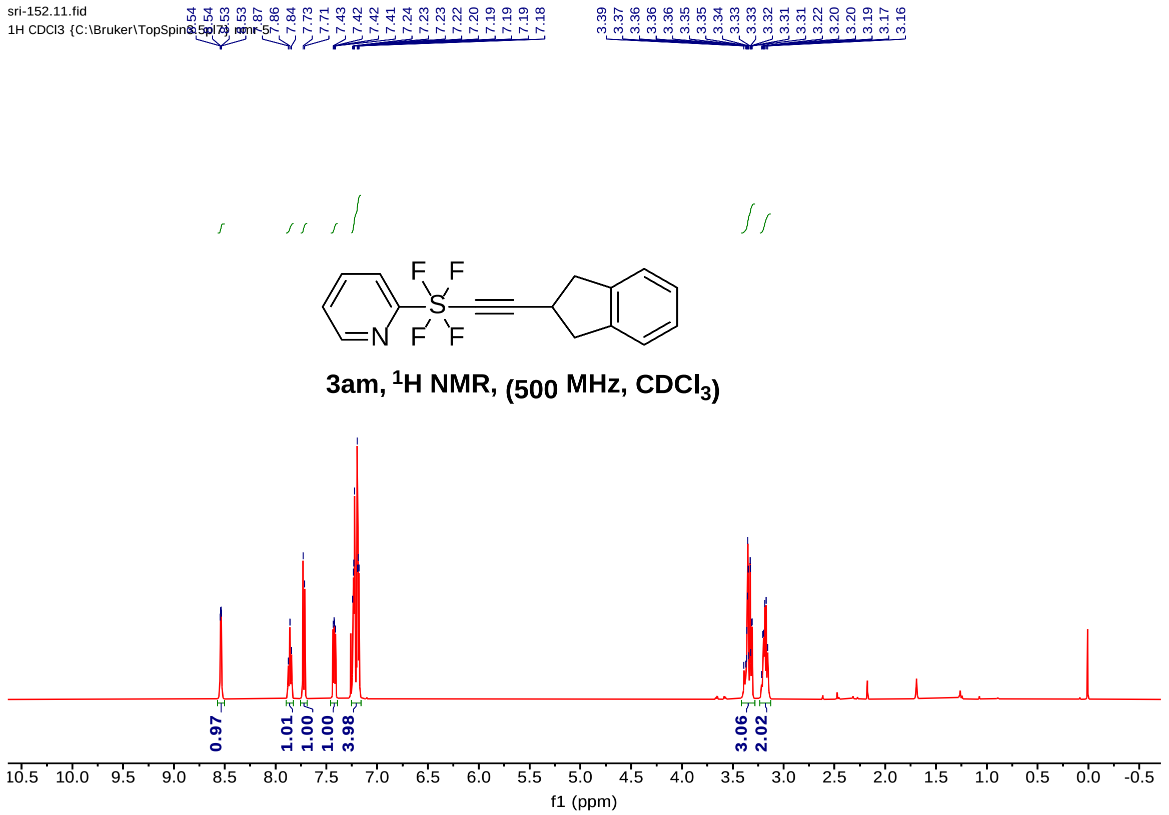
sri-152.11.fid
1H CDCl3 {C:\Bruker\TopSpin3.5pl7} nmr 5
8.54
8.54
8.53
8.53
7.87
7.86
7.84
7.73
7.71
7.43
7.42
7.42
7.41
7.24
7.23
7.23
7.22
7.20
7.19
7.19
7.19
7.18
3.39
3.37
3.36
3.36
3.36
3.35
3.35
3.34
3.33
3.33
3.32
3.31
3.31
3.22
3.20
3.20
3.19
3.17
3.16
F
S
N
3am, 1H NMR, (500 MHz, CDCl3)
0.97
1.01
1.00
1.00
3.98
3.06
2.02
f1 (ppm)

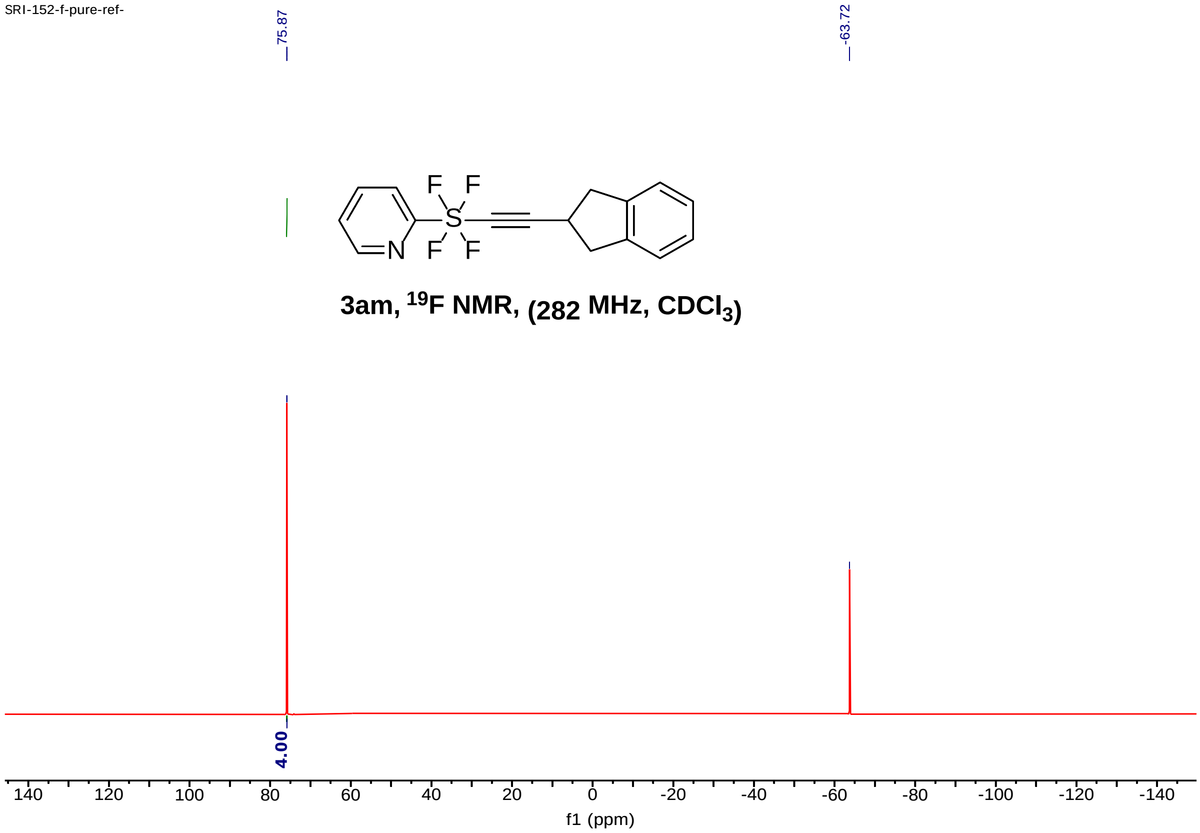
SRI-152-f-pure-ref-
75.87
-63.72
F
S
N
3am, 19F NMR, (282 MHz, CDCl3)
4.00
f1 (ppm)

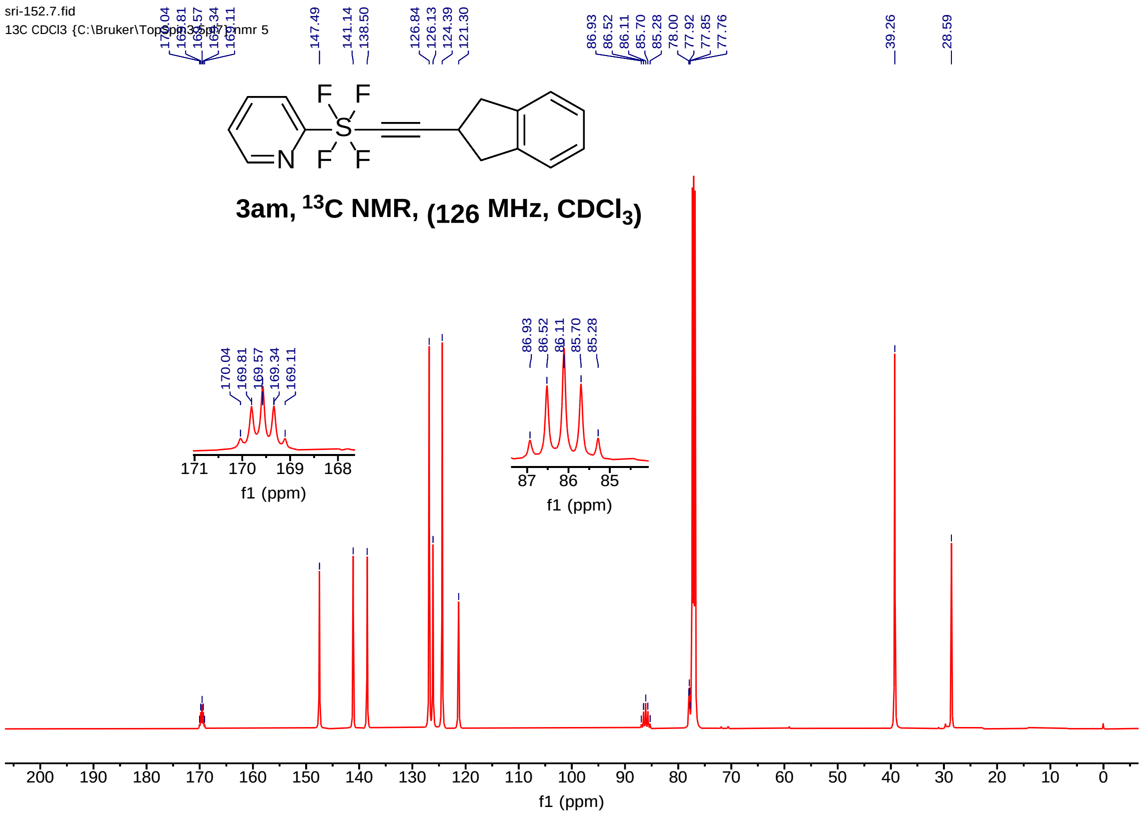
sri-152.7.fid
13C CDCl3 {C:\Bruker\TopSpin3.5pl7} nmr 5
3am, 13C NMR, (126 MHz, CDCl3)
f1 (ppm)

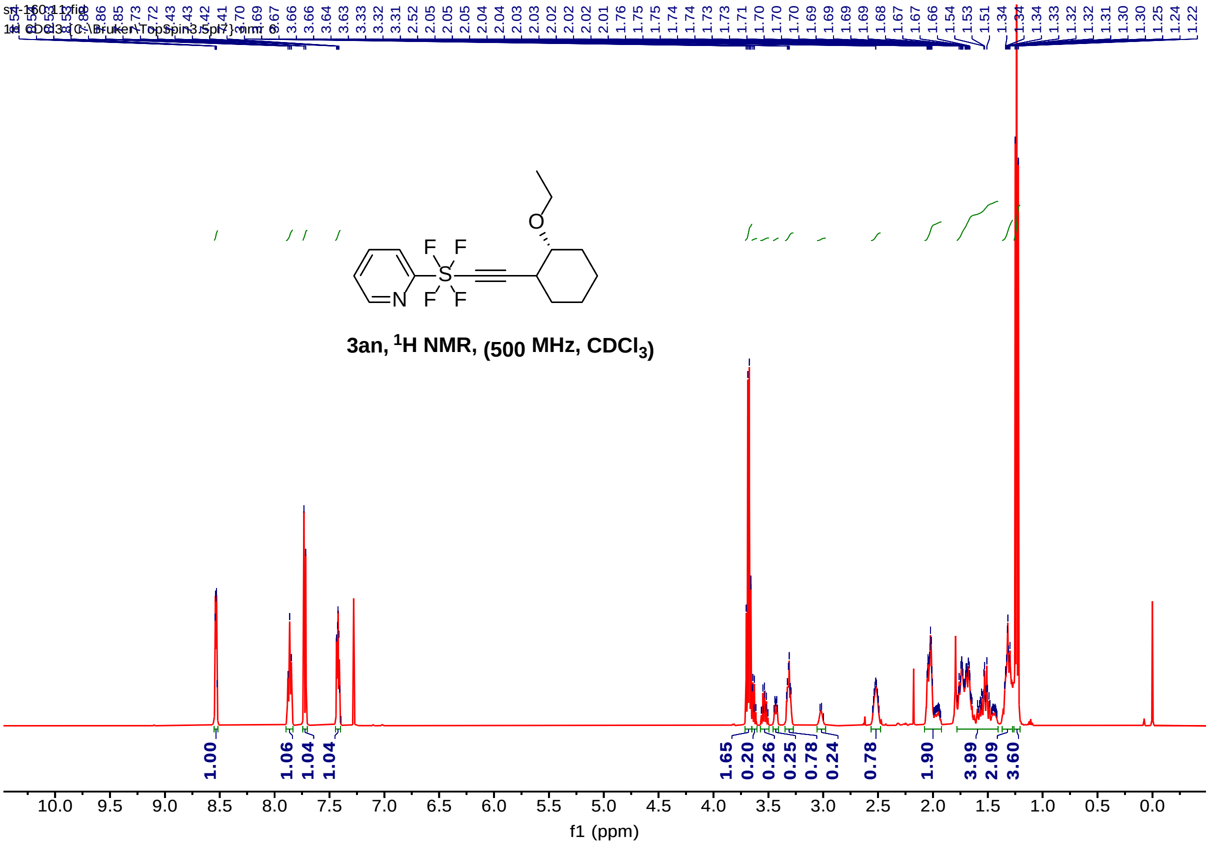
3an, 1H NMR, (500 MHz, CDCl3)
f1 (ppm)

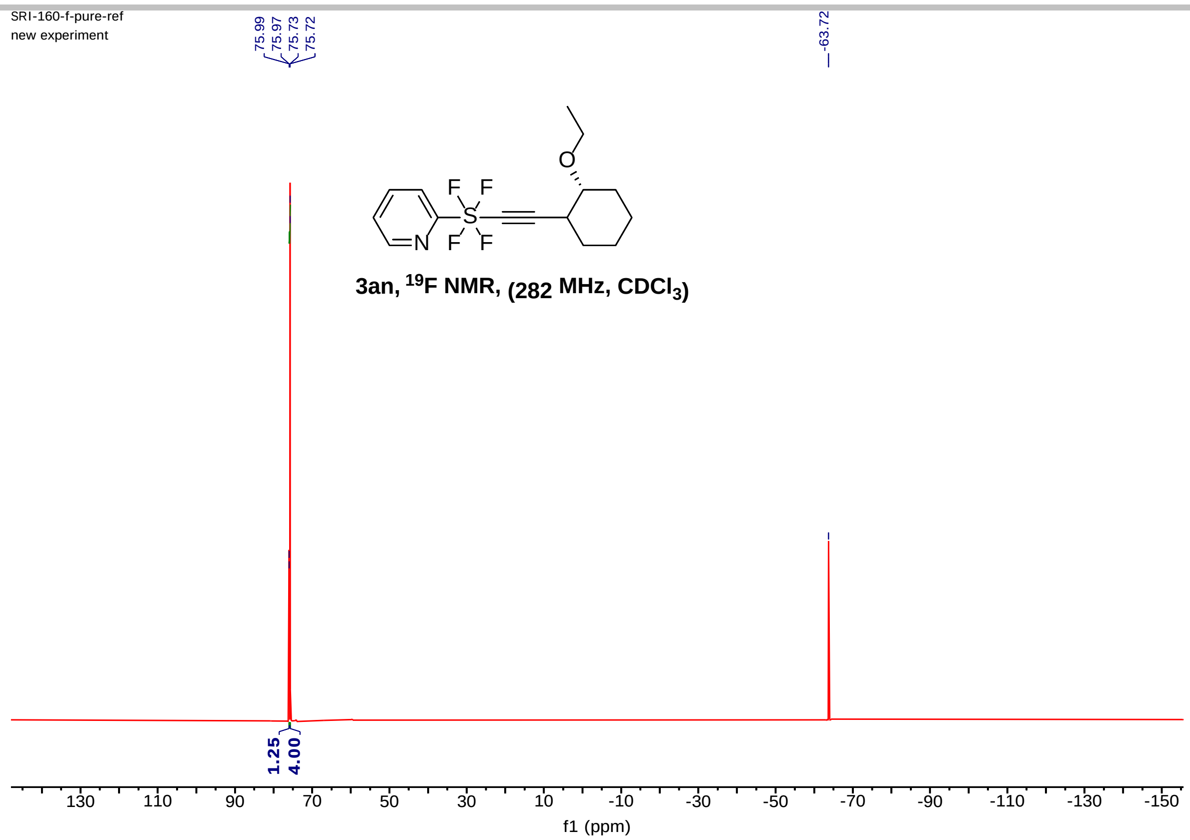
SRI-160-f-pure-ref
new experiment
75.99
75.97
75.73
75.72
-63.72
3an, 19F NMR, (282 MHz, CDCl3)
1.25
4.00
f1 (ppm)

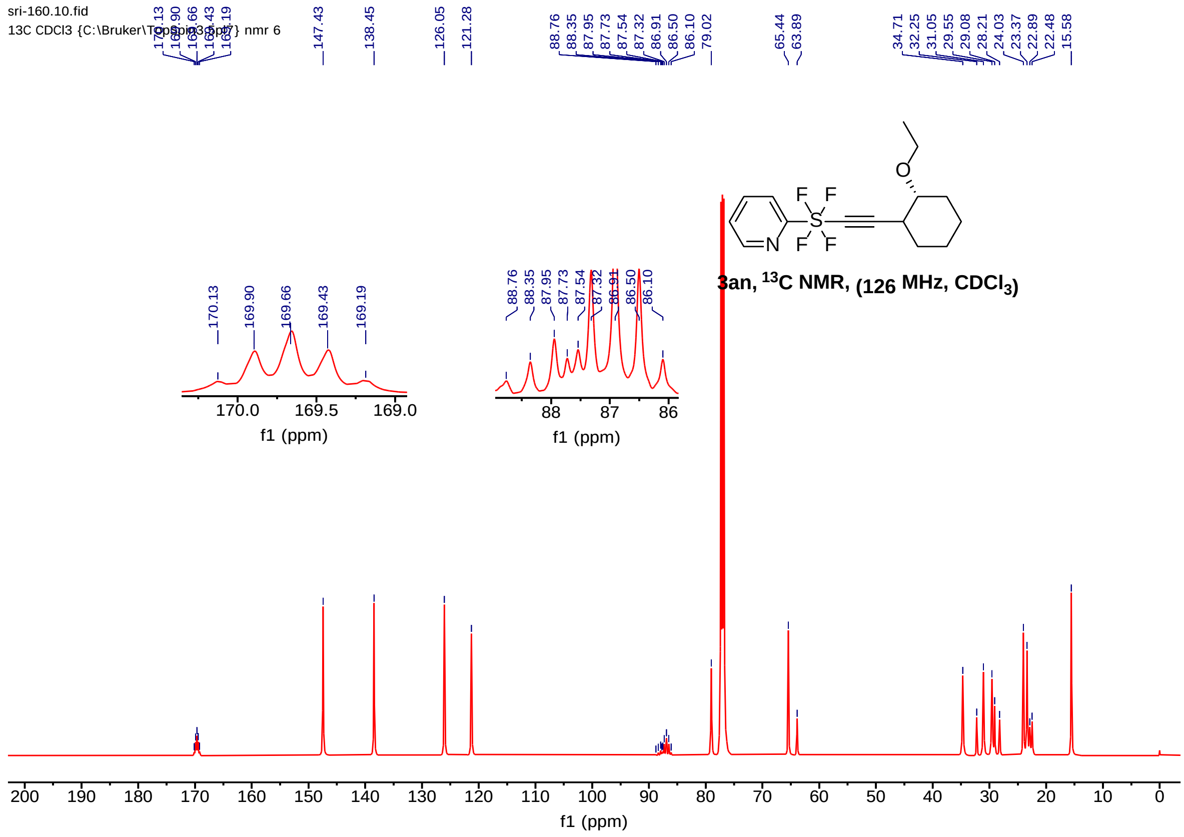
sri-160.10.fid
13C CDCl3 {C:\Bruker\TopSpin3.6pl7} nmr 6
170.13
169.90
169.66
169.43
169.19
147.43
138.45
126.05
121.28
88.76
88.35
87.95
87.73
87.54
87.32
86.91
86.50
86.10
79.02
65.44
63.89
34.71
32.25
31.05
29.55
29.08
28.21
24.03
23.37
22.89
22.48
15.58
3an, 13C NMR, (126 MHz, CDCl3)
f1 (ppm)

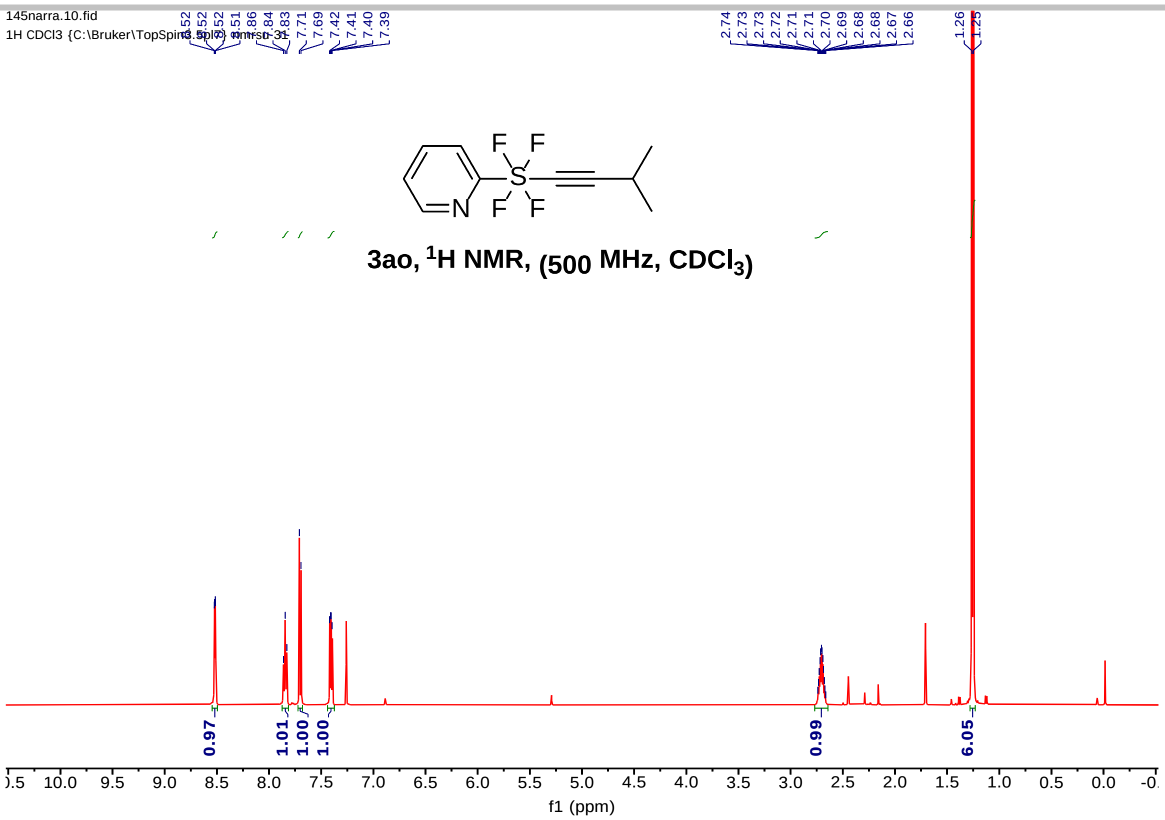

3ao, ^{1}H NMR, (500 MHz, $CDCl_3$)

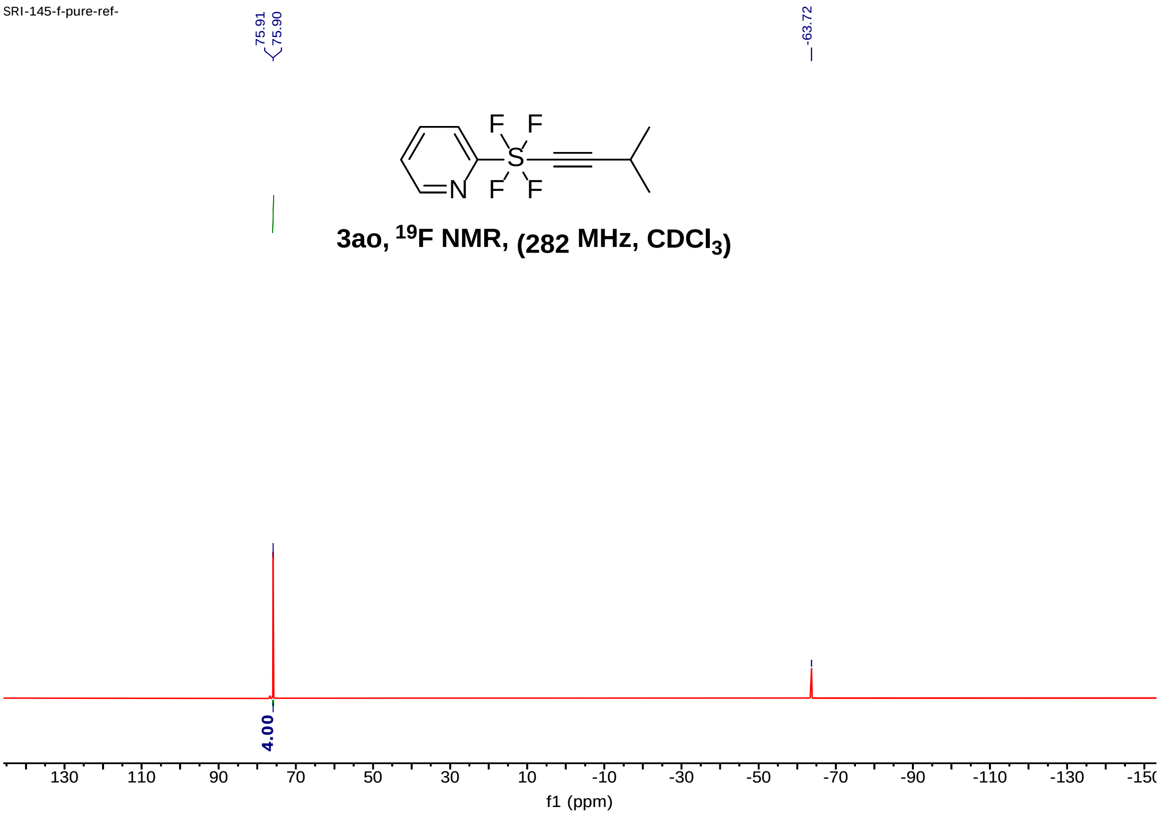

3ao, ^{19}F NMR, (282 MHz, $CDCl_3$)

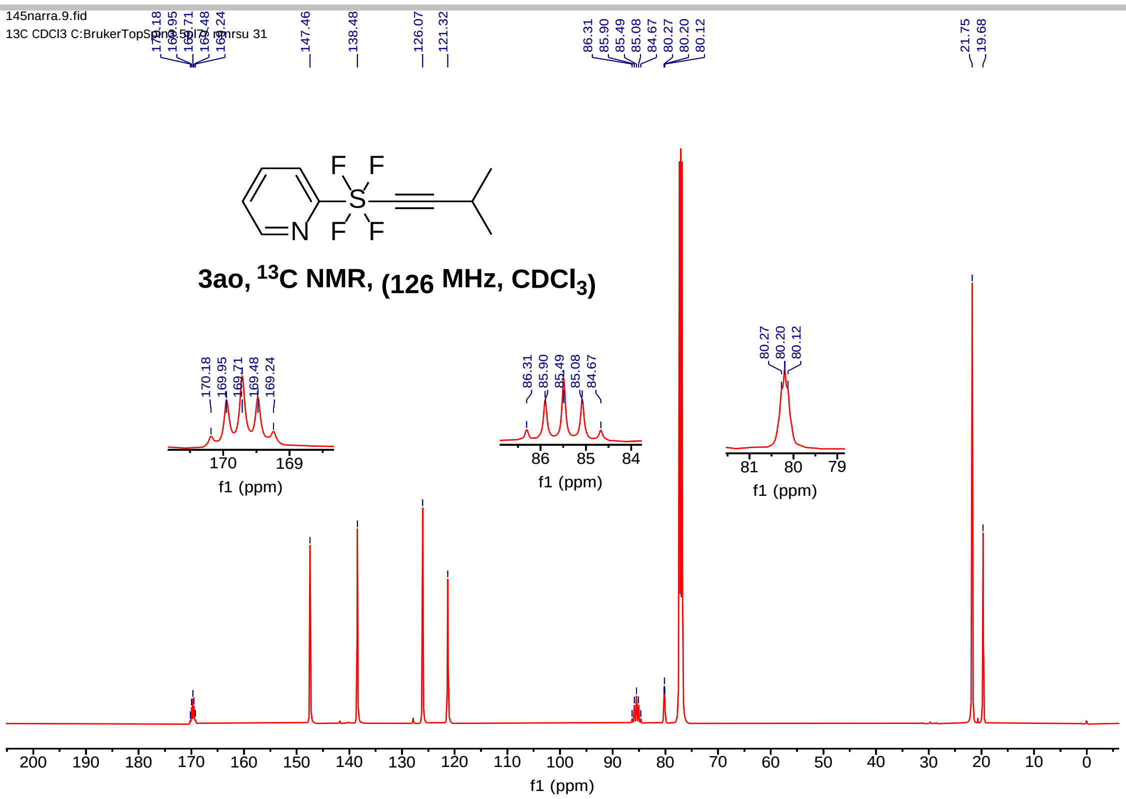
145narra.9.fid
13C CDCl3 C:BrukerTopSpin3.5pl7 nmrsu 31
3ao, 13C NMR, (126 MHz, CDCl3)
f1 (ppm)

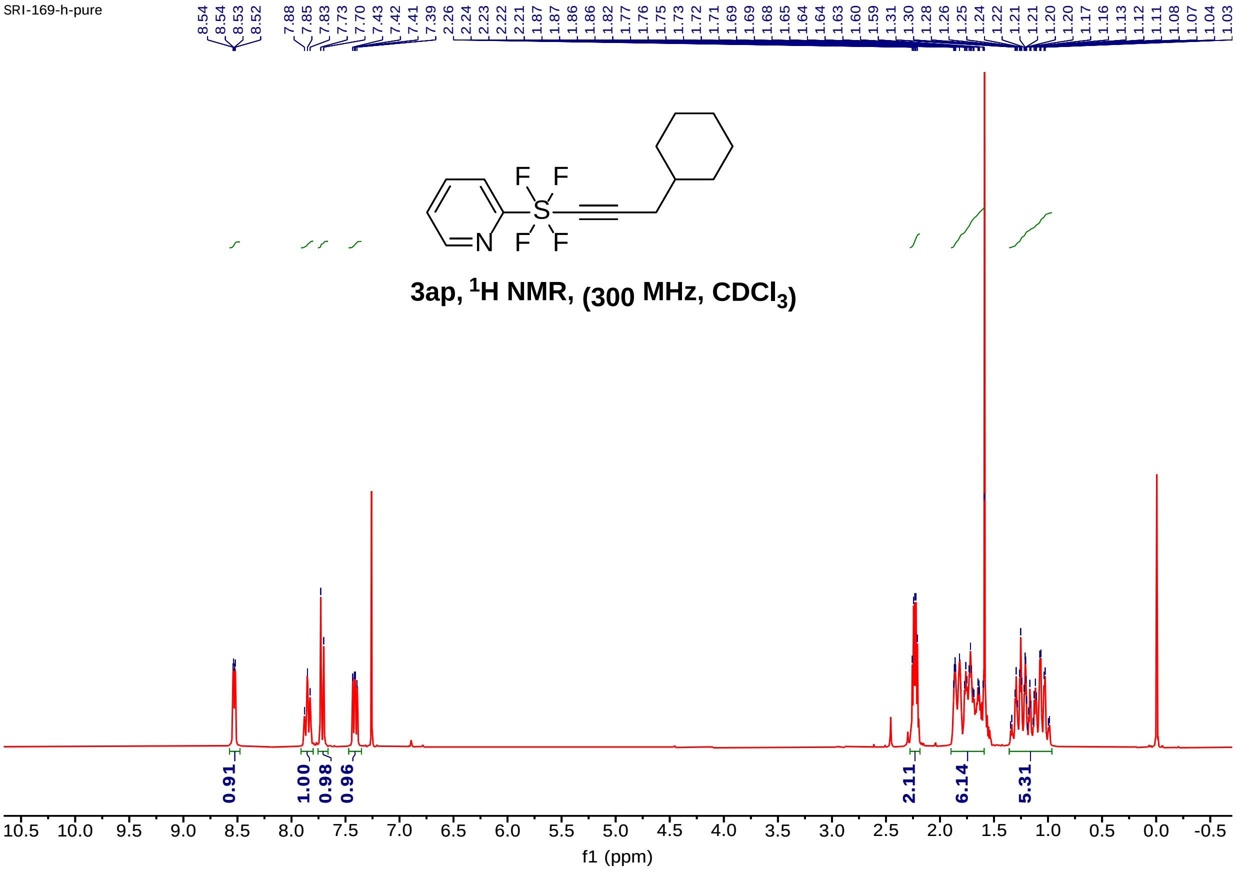
SRI-169-h-pure
3ap, 1H NMR, (300 MHz, CDCl3)
0.91
1.00
0.98
0.96
2.11
6.14
5.31
f1 (ppm)

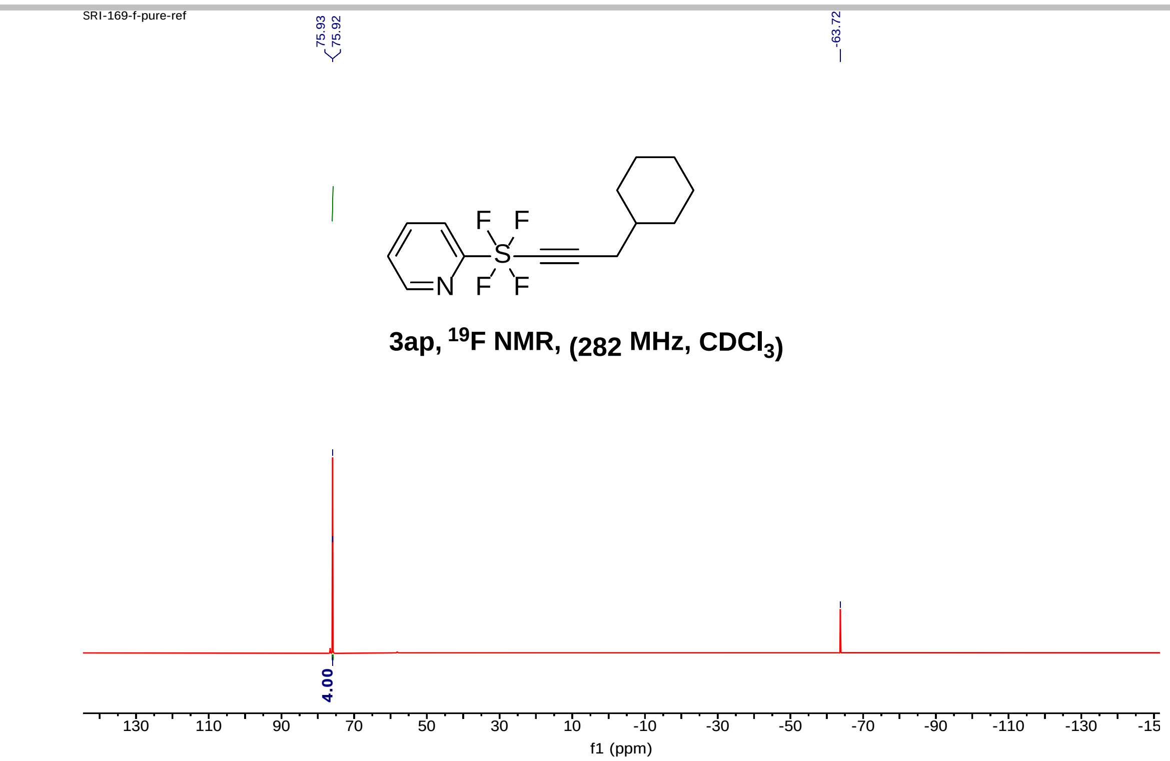

3ap, ^{19}F NMR, (282 MHz, $CDCl_3$)

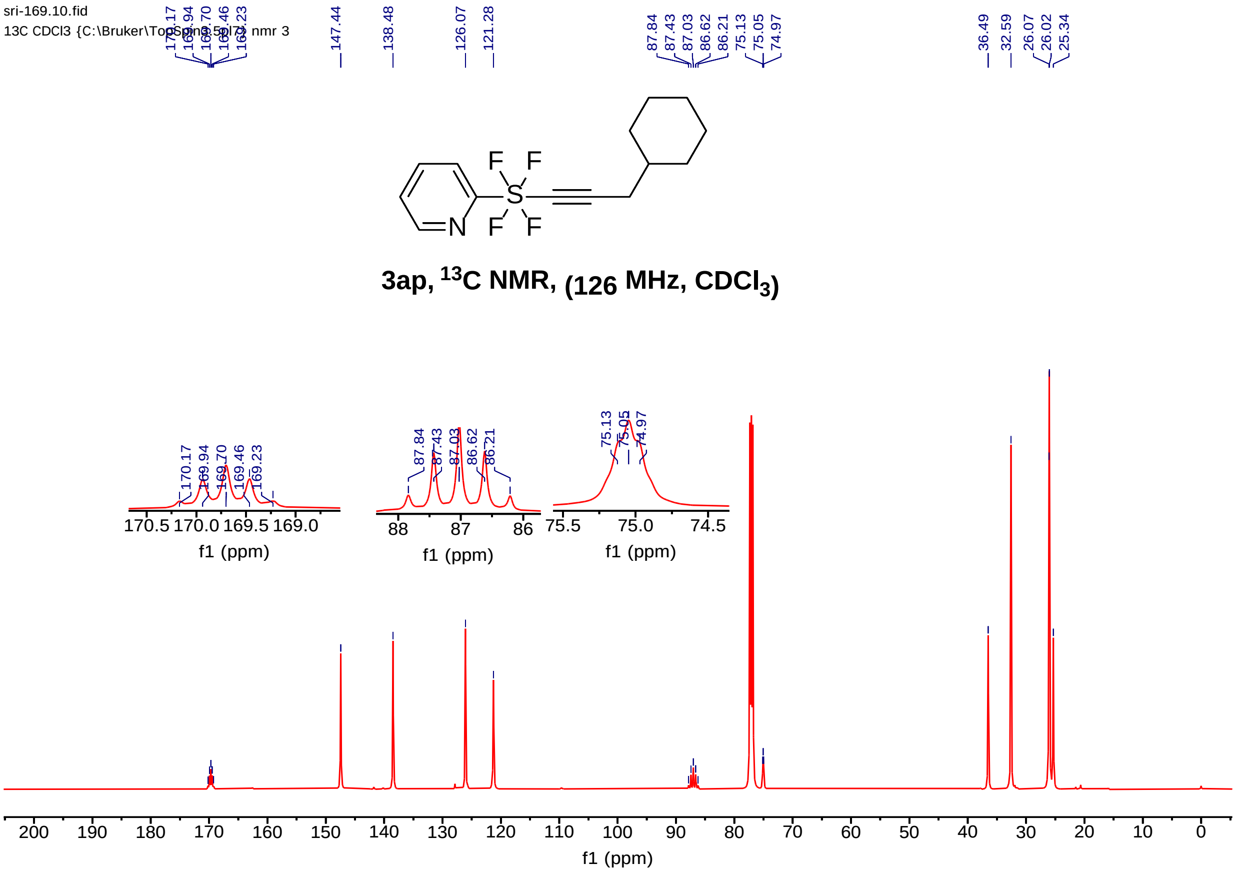

3ap, ^{13}C NMR, (126 MHz, $CDCl_3$)

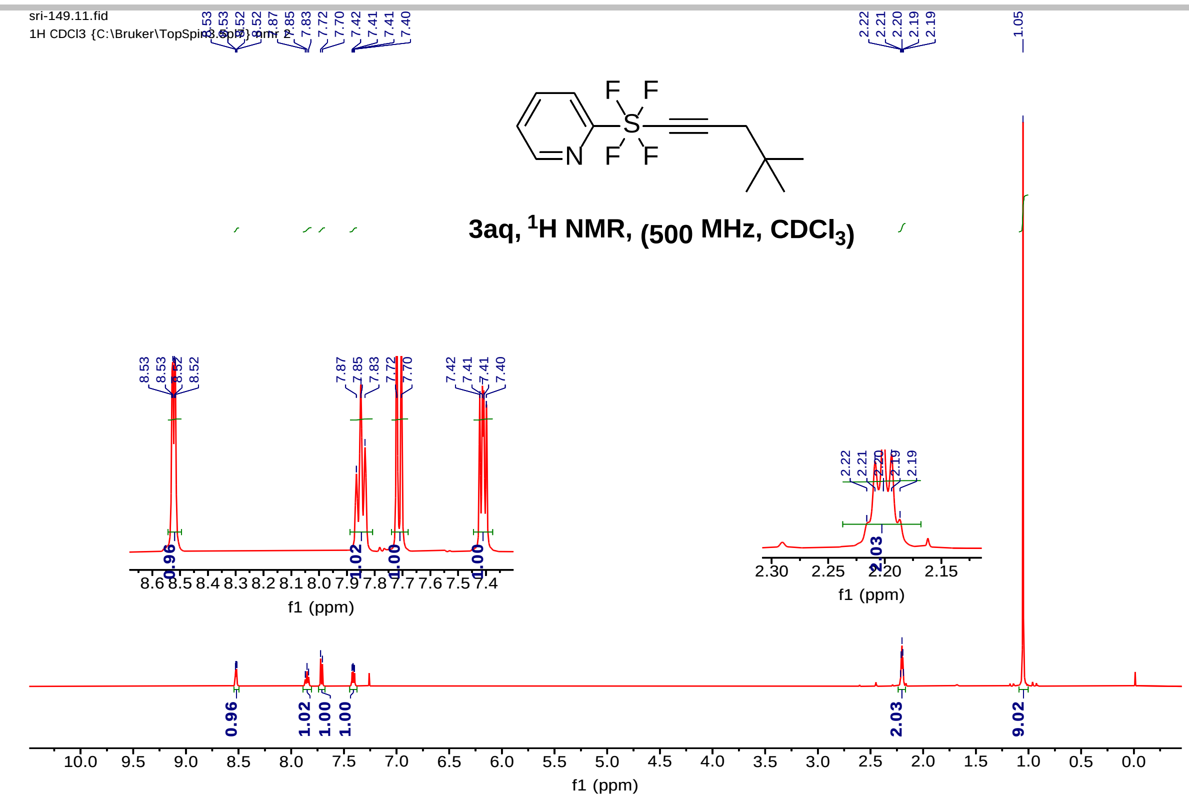
sri-149.11.fid
1H CDCl3 {C:\Bruker\TopSpin3.5pl7} nmr 2
3aq, ^{1}H NMR, (500 MHz, CDCl$_3$)
f1 (ppm)

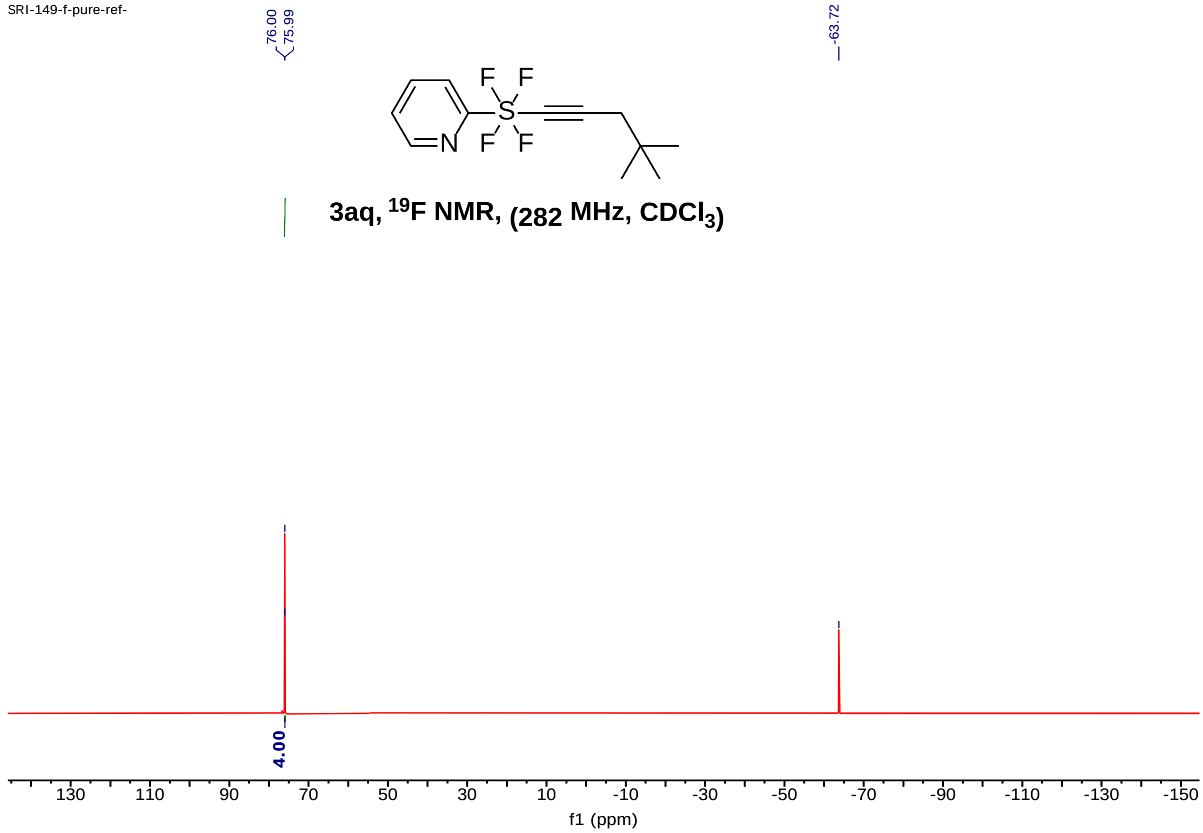
SRI-149-f-pure-ref-
76.00
75.99
-63.72
3aq, ^{19}F NMR, (282 MHz, CDCl$_3$)
4.00
f1 (ppm)

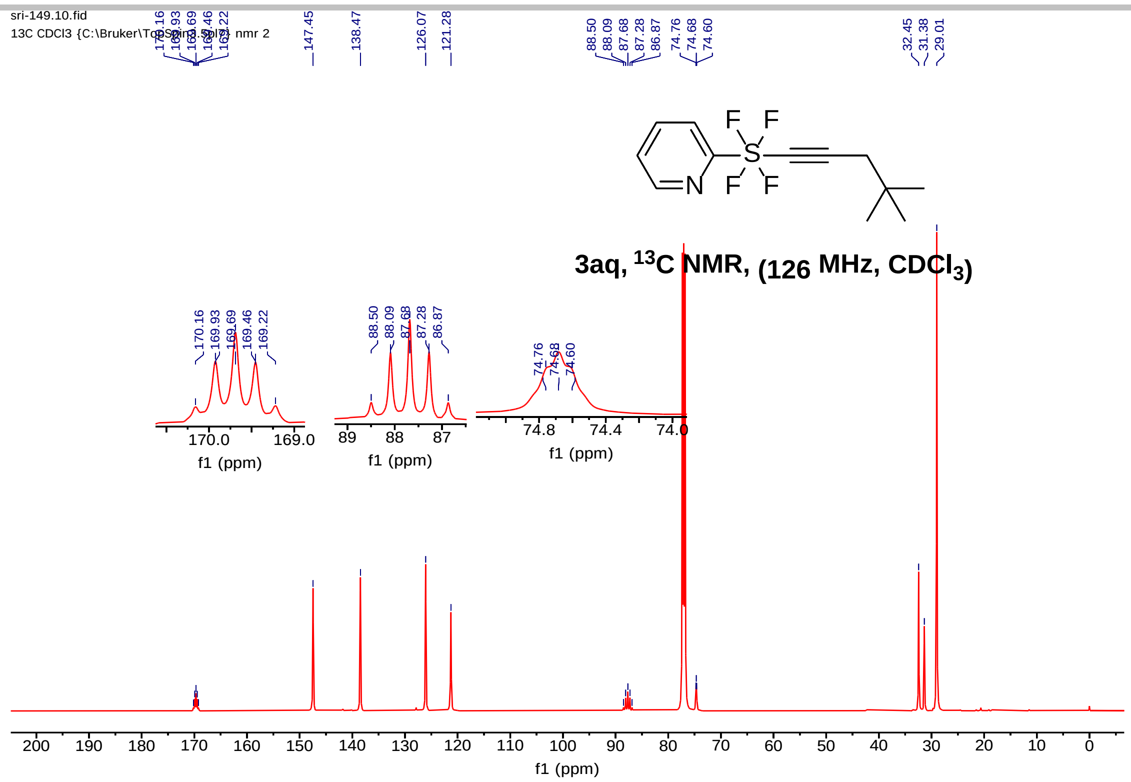
sri-149.10.fid
13C CDCl3 {C:\Bruker\TopSpin3.5pl7} nmr 2
3aq, 13C NMR, (126 MHz, CDCl3)
f1 (ppm)

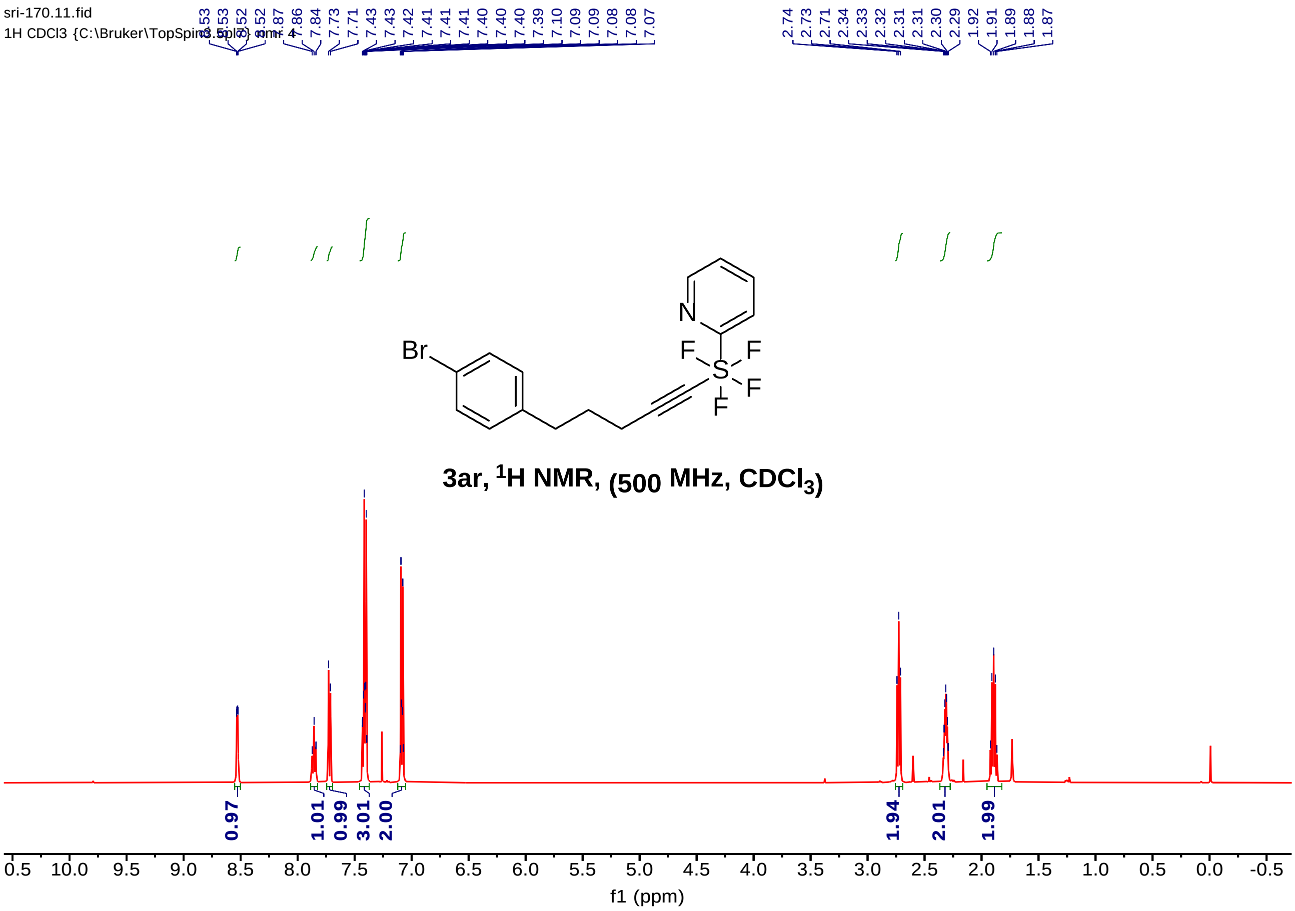
sri-170.11.fid
1H CDCl3 {C:\Bruker\TopSpin3.5pl7} nmr 4
3ar, 1H NMR, (500 MHz, CDCl3)
f1 (ppm)

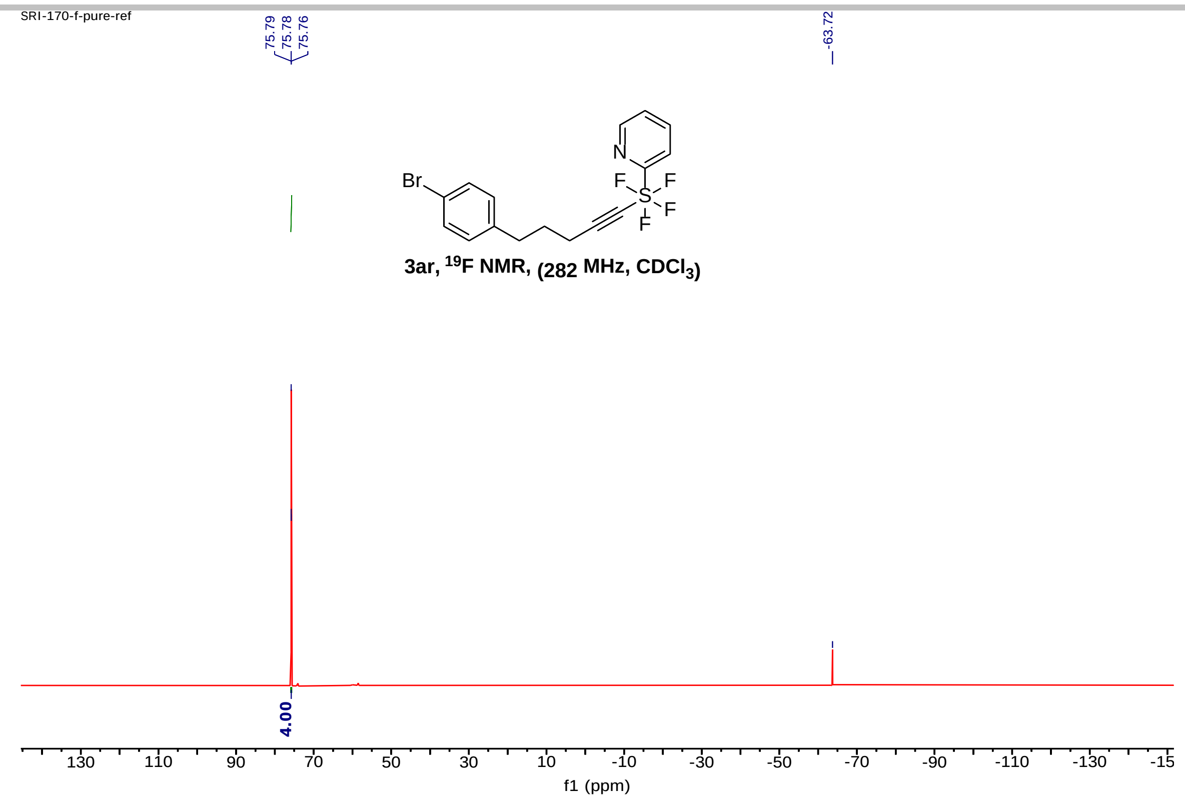
SRI-170-f-pure-ref
75.79
75.78
75.76
-63.72
Br
N
F
S
3ar, 19F NMR, (282 MHz, CDCl3)
4.00
f1 (ppm)

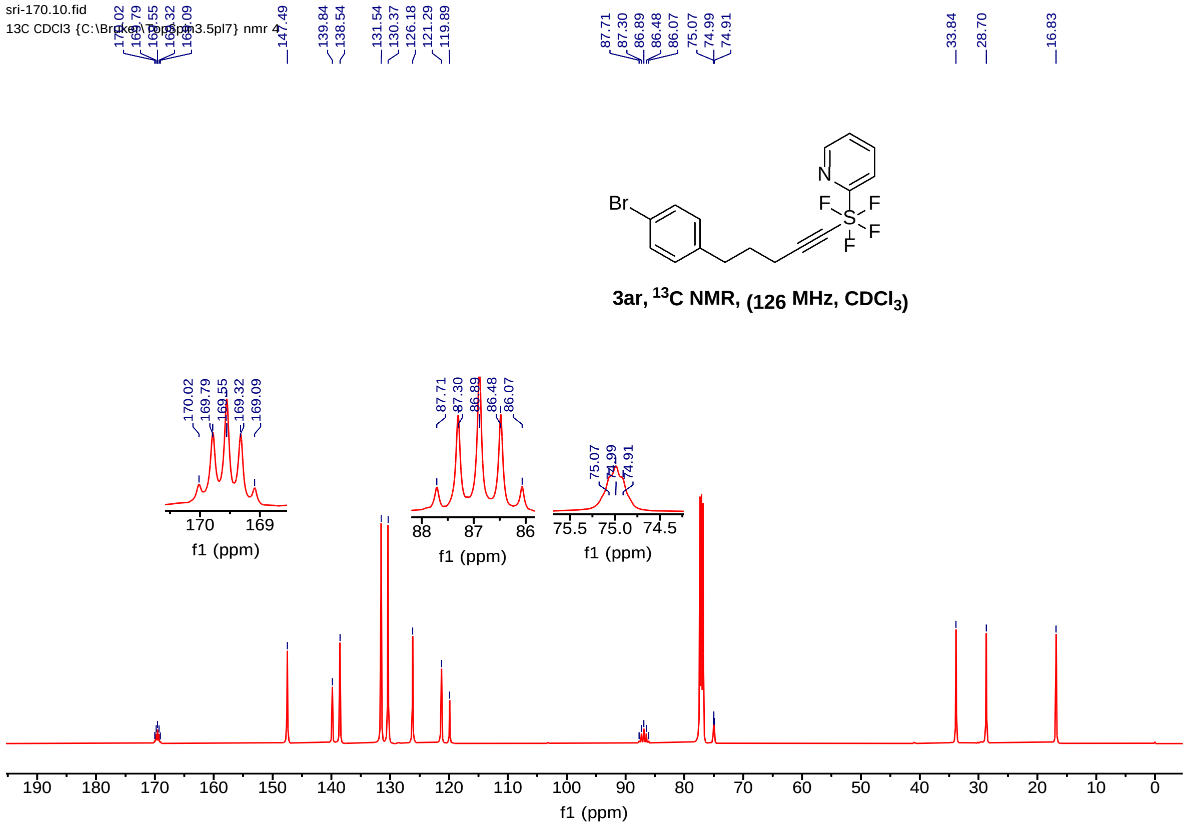
sri-170.10.fid
13C CDCl3 {C:\Bruker\TopSpin3.5pl7} nmr 4
170.02
169.79
169.55
169.32
169.09
147.49
139.84
138.54
131.54
130.37
126.18
121.29
119.89
87.71
87.30
86.89
86.48
86.07
75.07
74.99
74.91
33.84
28.70
16.83
Br
N
F
S
3ar, 13C NMR, (126 MHz, CDCl3)
f1 (ppm)

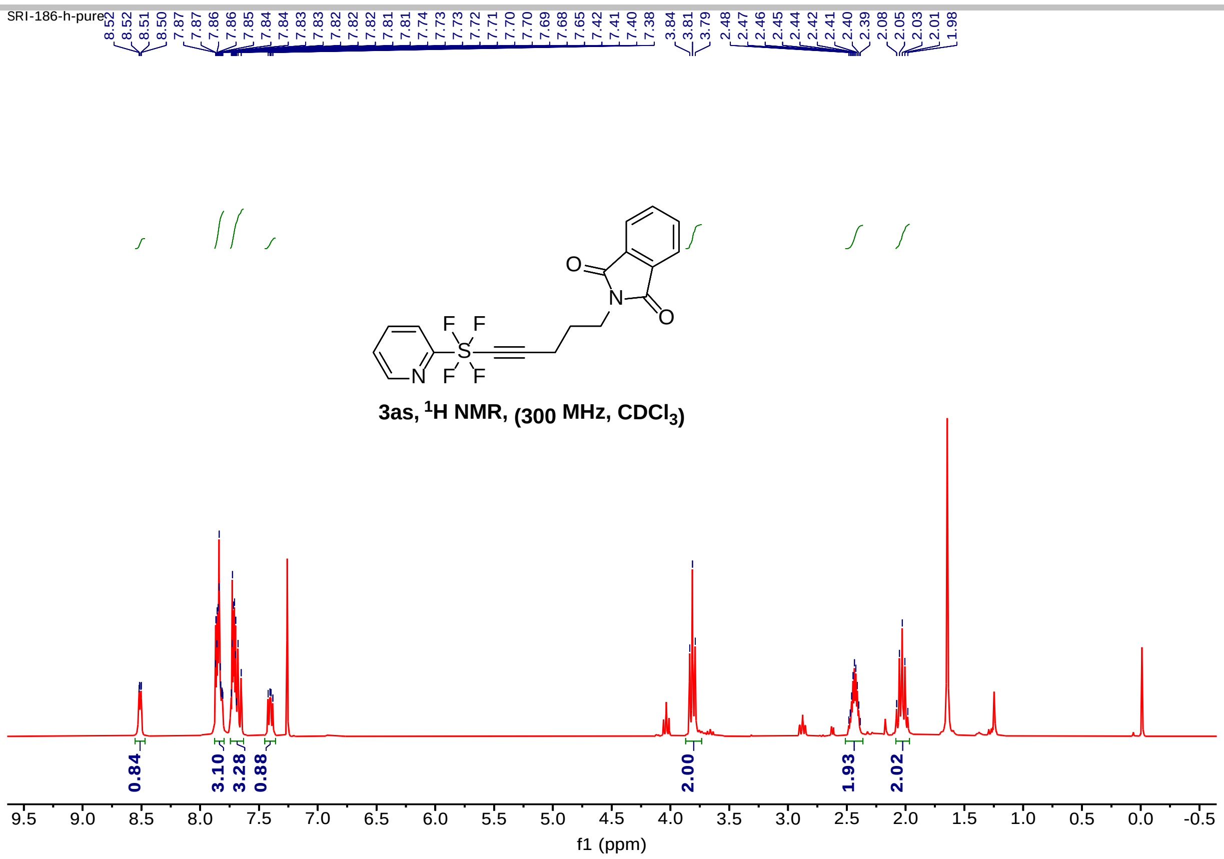

3as, ^{1}H NMR, (300 MHz, $CDCl_3$)

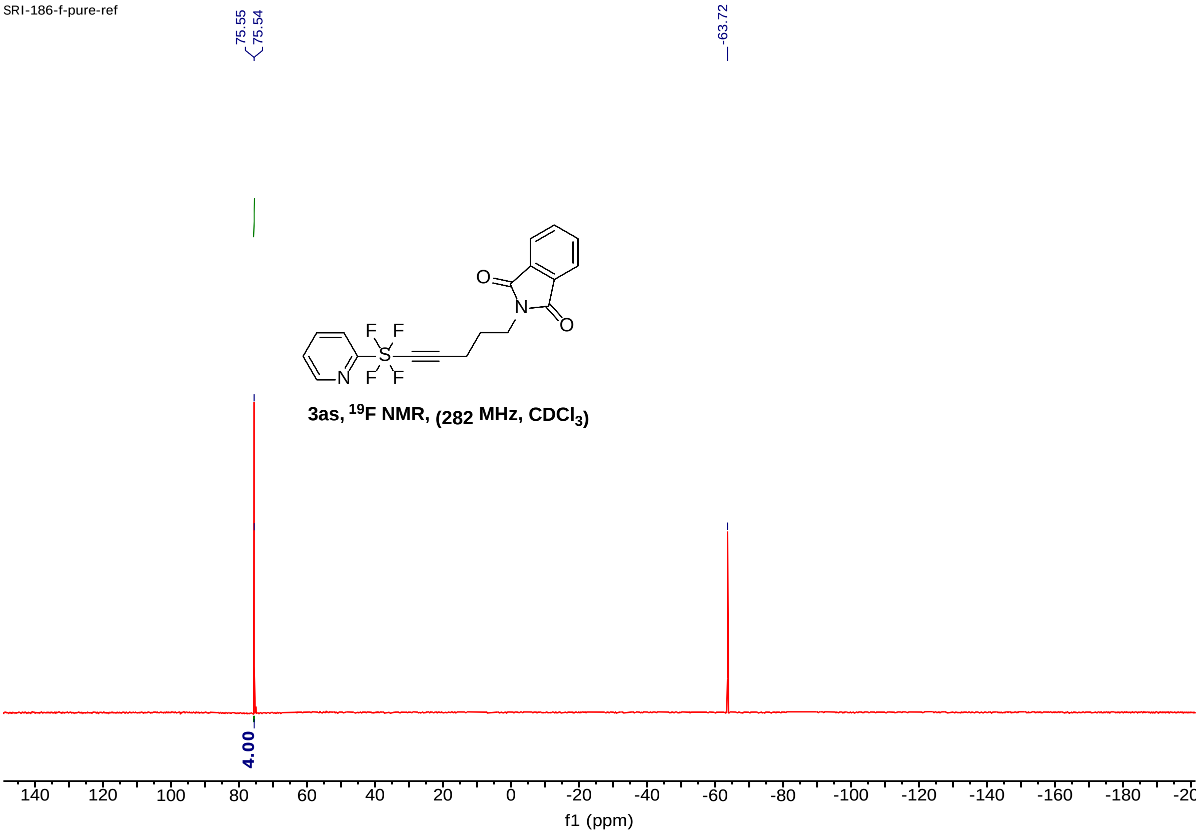

3as, ^{19}F NMR, (282 MHz, $CDCl_3$)

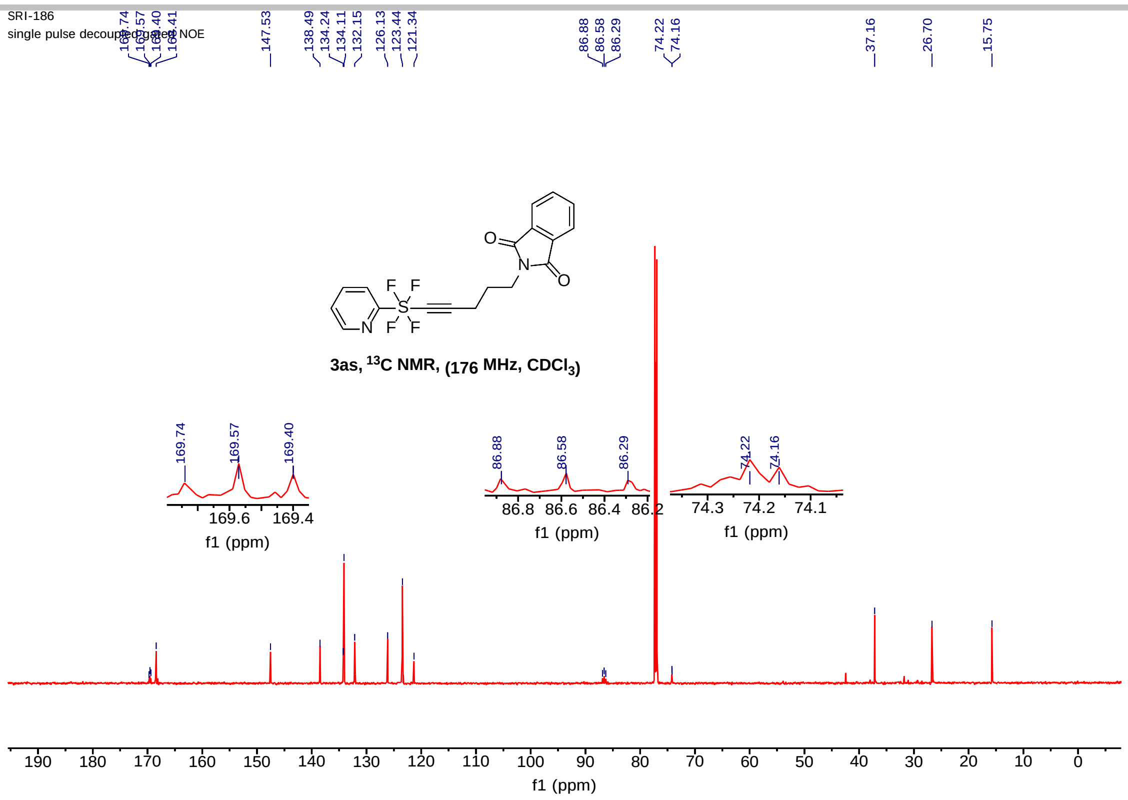
SRI-186
single pulse decoupled gated NOE
3as, ^{13}C NMR, (176 MHz, CDCl$_3$)
f1 (ppm)

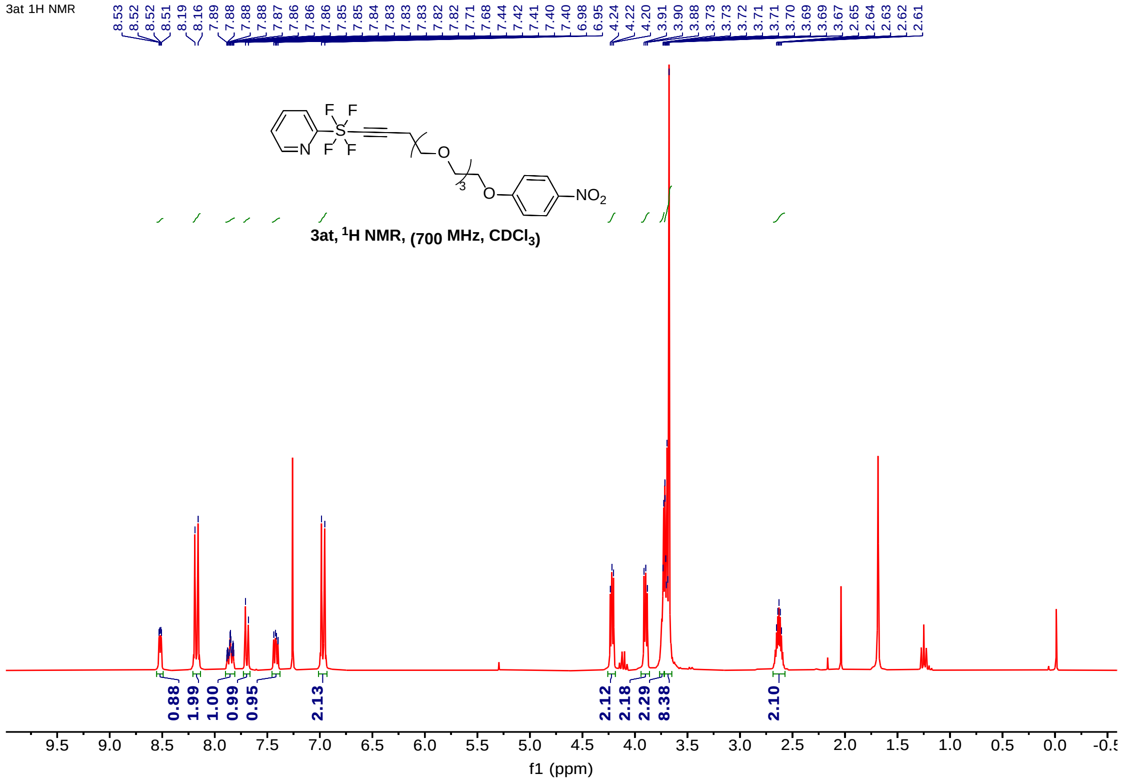
3at 1H NMR
3at, ^{1}H NMR, (700 MHz, CDCl$_3$)
f1 (ppm)

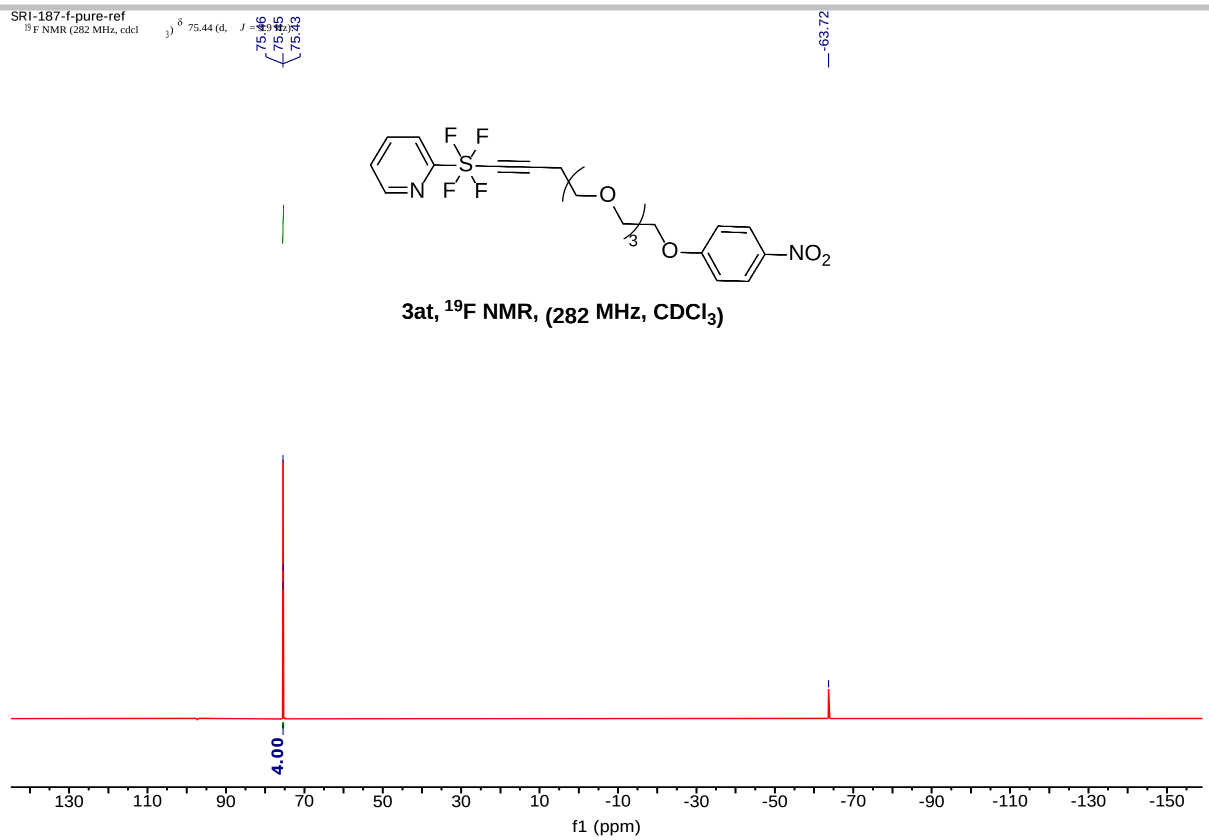

3at, ^{19}F NMR, (282 MHz, $CDCl_3$)

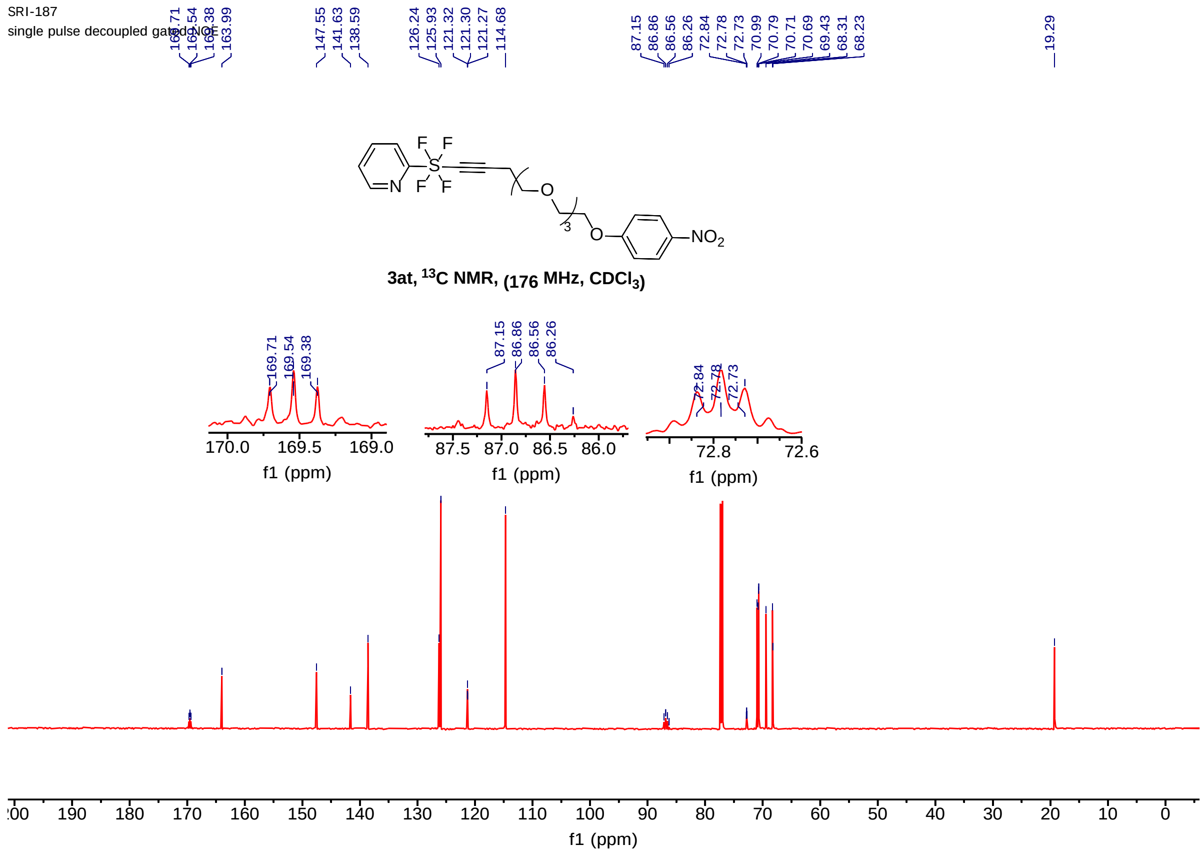

3at, ^{13}C NMR, (176 MHz, $CDCl_3$)

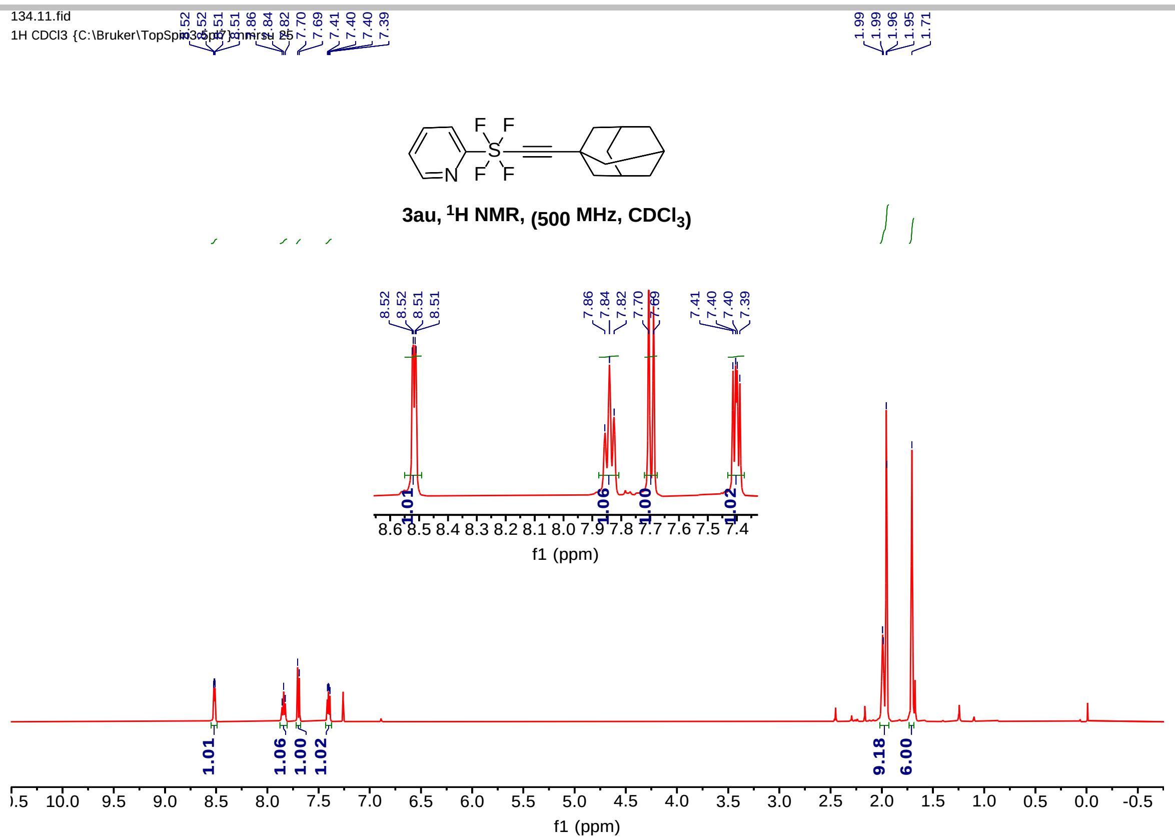
134.11.fid
1H CDCl3 {C:\Bruker\TopSpin3.5pl7} nmrsu 25
3au, ^{1}H NMR, (500 MHz, $CDCl_3$)
1.01
1.06
1.00
1.02
9.18
6.00
f1 (ppm)

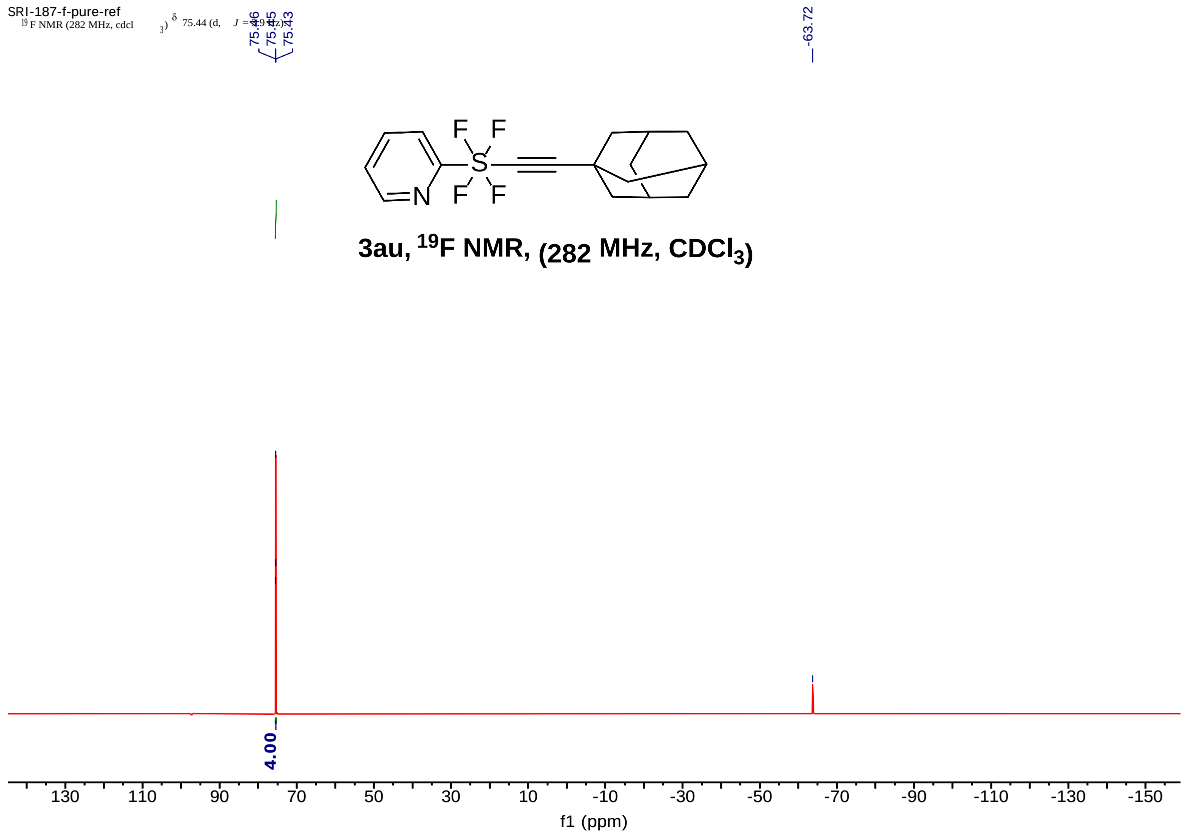
SRI-187-f-pure-ref
-63.72
3au, ^{19}F NMR, (282 MHz, $CDCl_3$)
4.00
f1 (ppm)

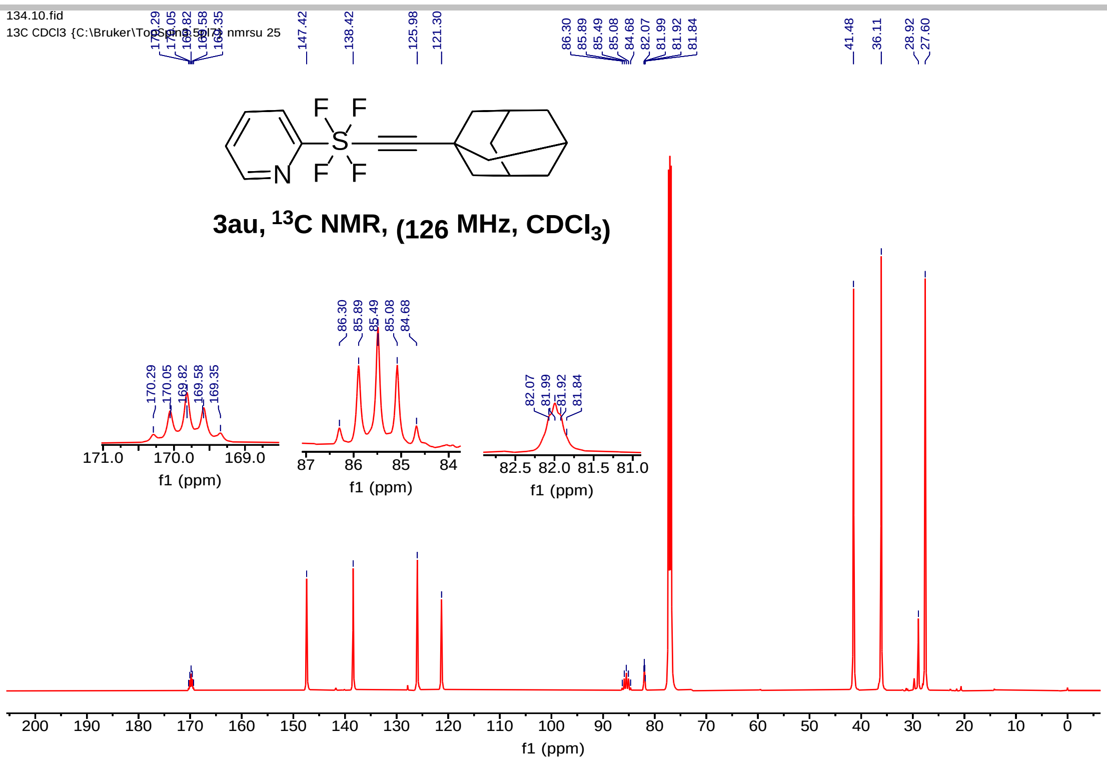
134.10.fid
13C CDCl3 {C:\Bruker\TopSpin3.5pl7} nmrsu 25
3au, 13C NMR, (126 MHz, CDCl3)
f1 (ppm)

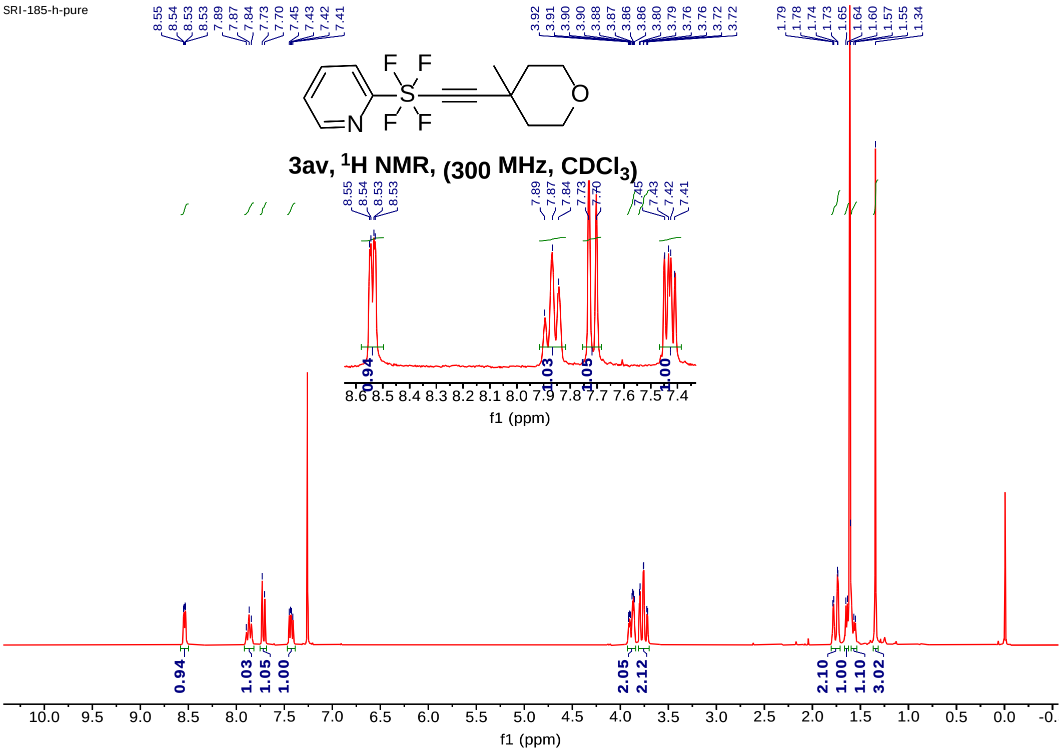
SRI-185-h-pure
3av, 1H NMR, (300 MHz, CDCl3)
f1 (ppm)

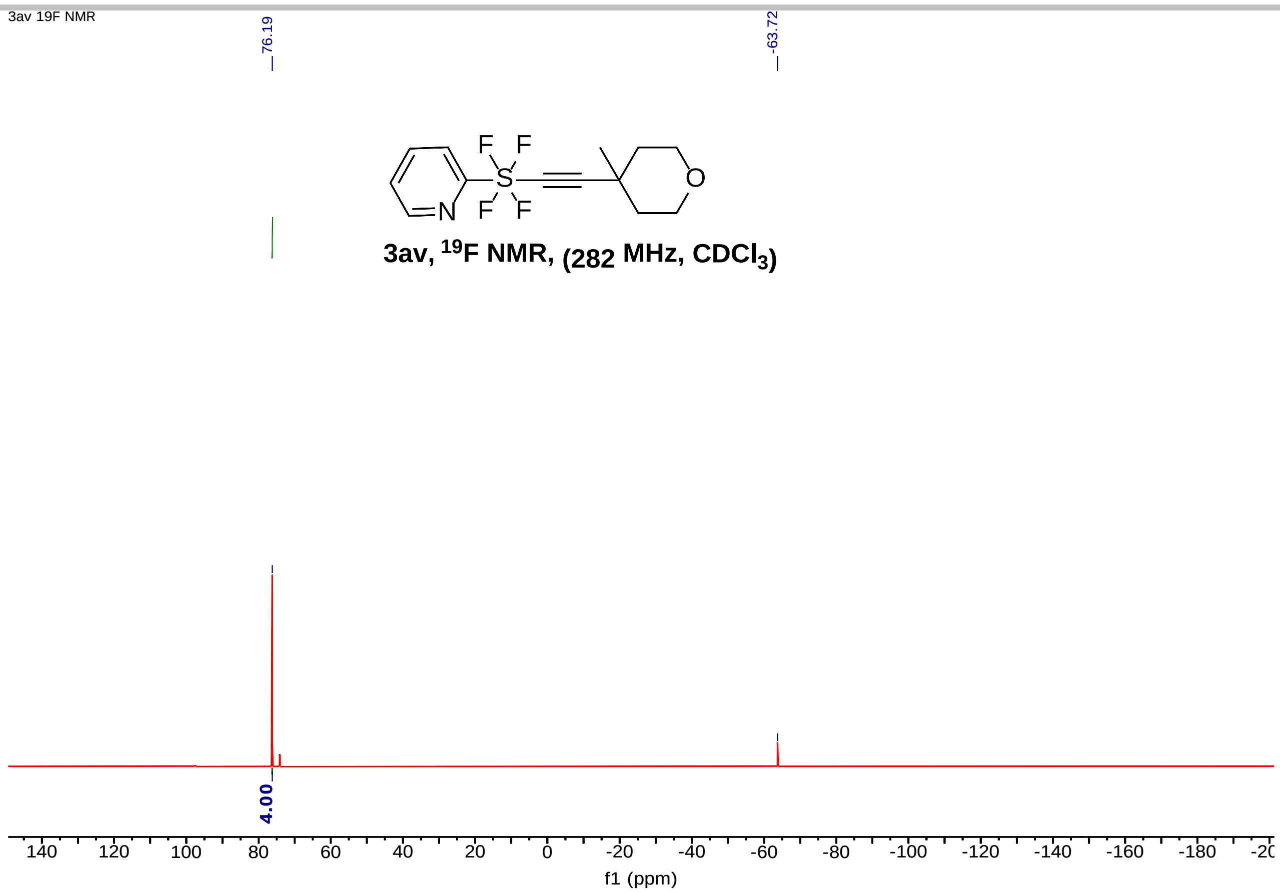
3av 19F NMR
-76.19
-63.72
F F
S
N F F
O
3av, 19F NMR, (282 MHz, CDCl3)
4.00
140 120 100 80 60 40 20 0 -20 -40 -60 -80 -100 -120 -140 -160 -180
f1 (ppm)

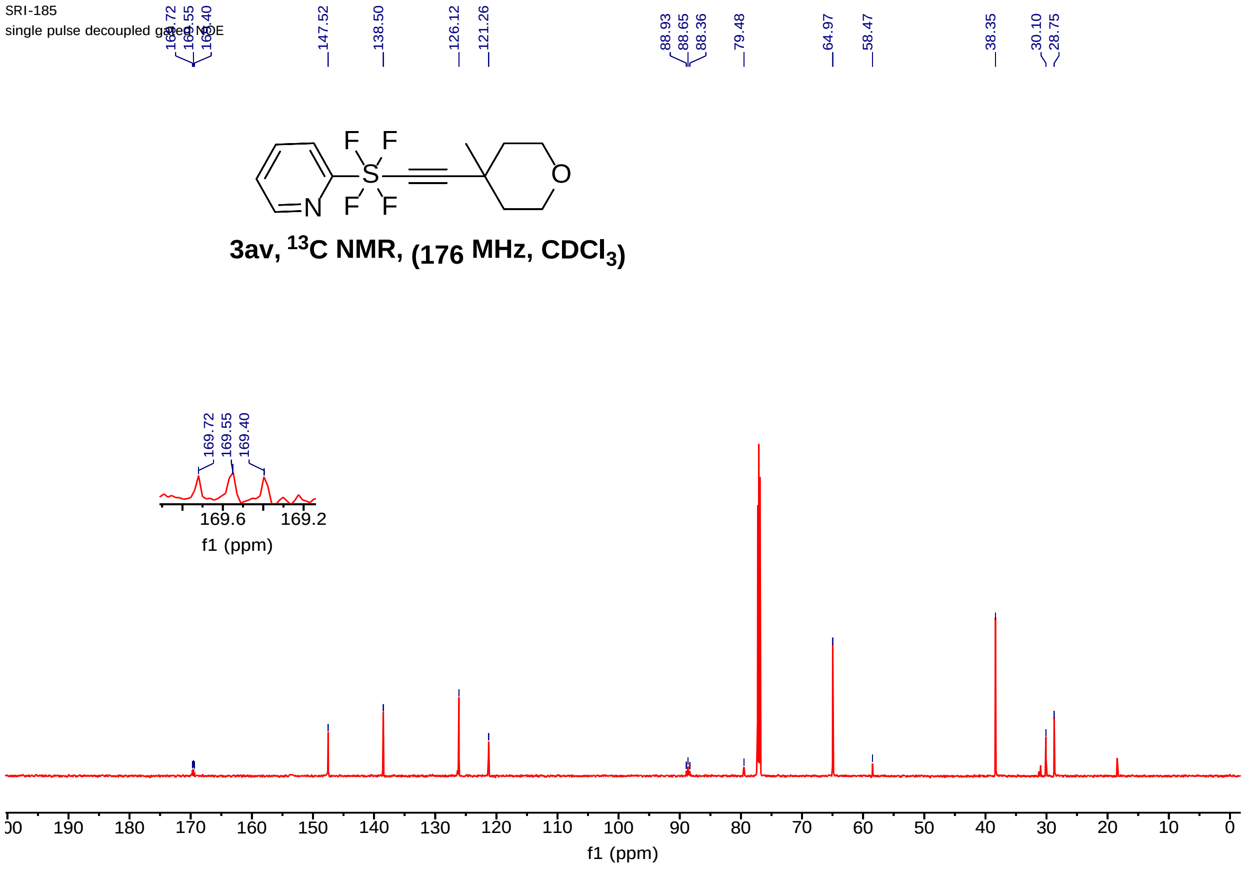
SRI-185
single pulse decoupled gated NOE
169.72
169.55
169.40
147.52
138.50
126.12
121.26
88.93
88.65
88.36
79.48
64.97
58.47
38.35
30.10
28.75
F F
S
N F F
O
3av, 13C NMR, (176 MHz, CDCl3)
169.72
169.55
169.40
169.6 169.2
f1 (ppm)
190 180 170 160 150 140 130 120 110 100 90 80 70 60 50 40 30 20 10 0
f1 (ppm)

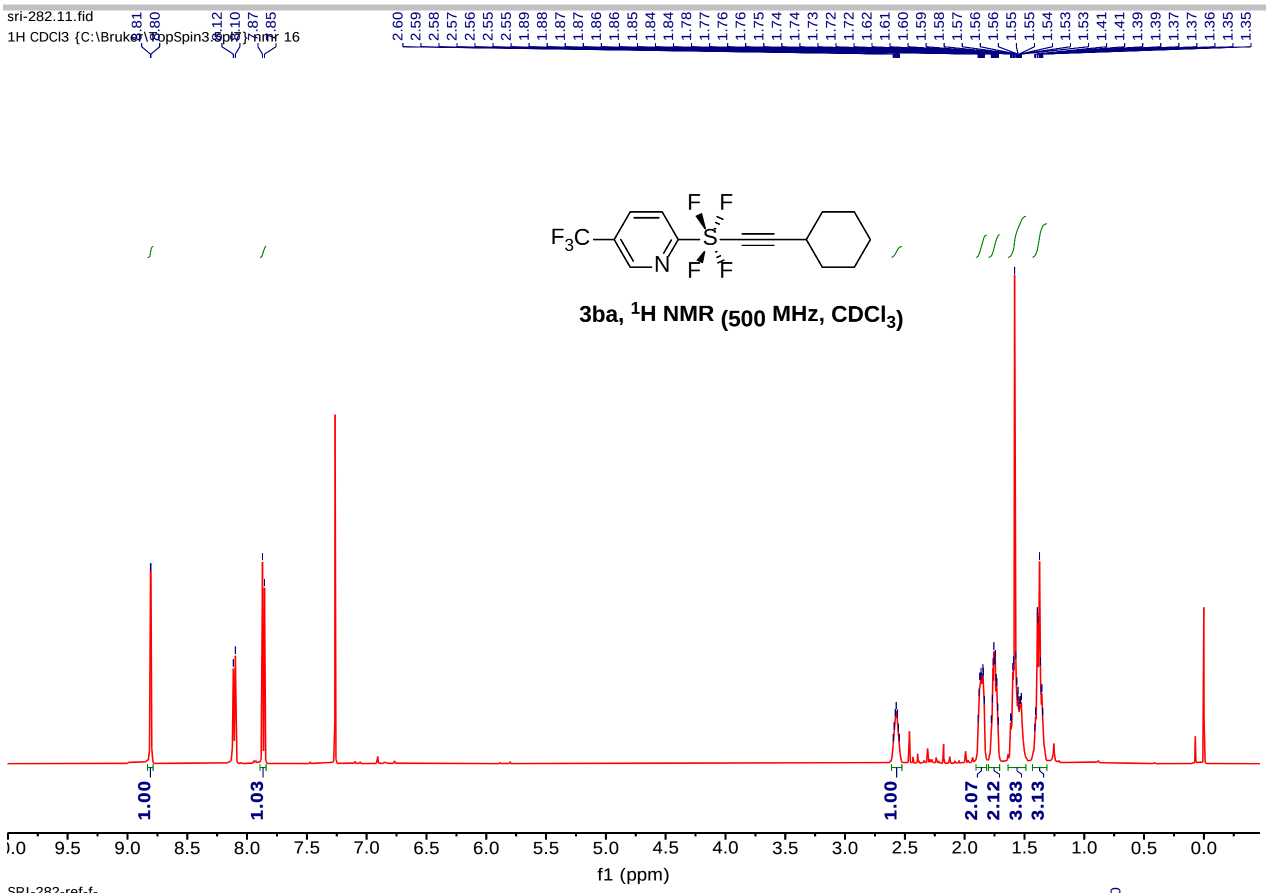
sri-282.11.fid
1H CDCl3 {C:\Bruker\TopSpin3.5pl7} nmr 16
3ba, 1H NMR (500 MHz, CDCl3)
f1 (ppm)

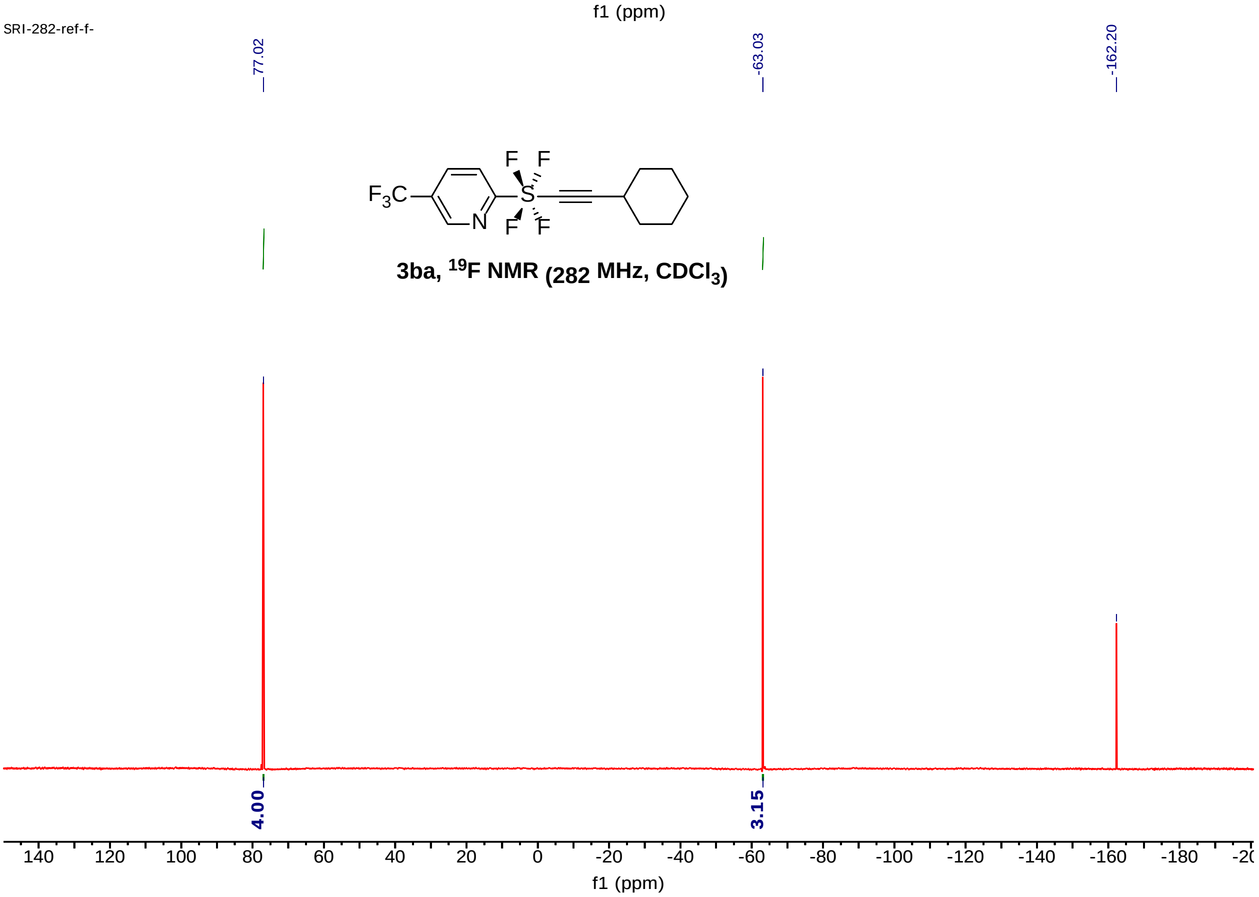
SRI-282-ref-f-
-77.02
-63.03
-162.20
3ba, 19F NMR (282 MHz, CDCl3)
4.00
3.15
f1 (ppm)

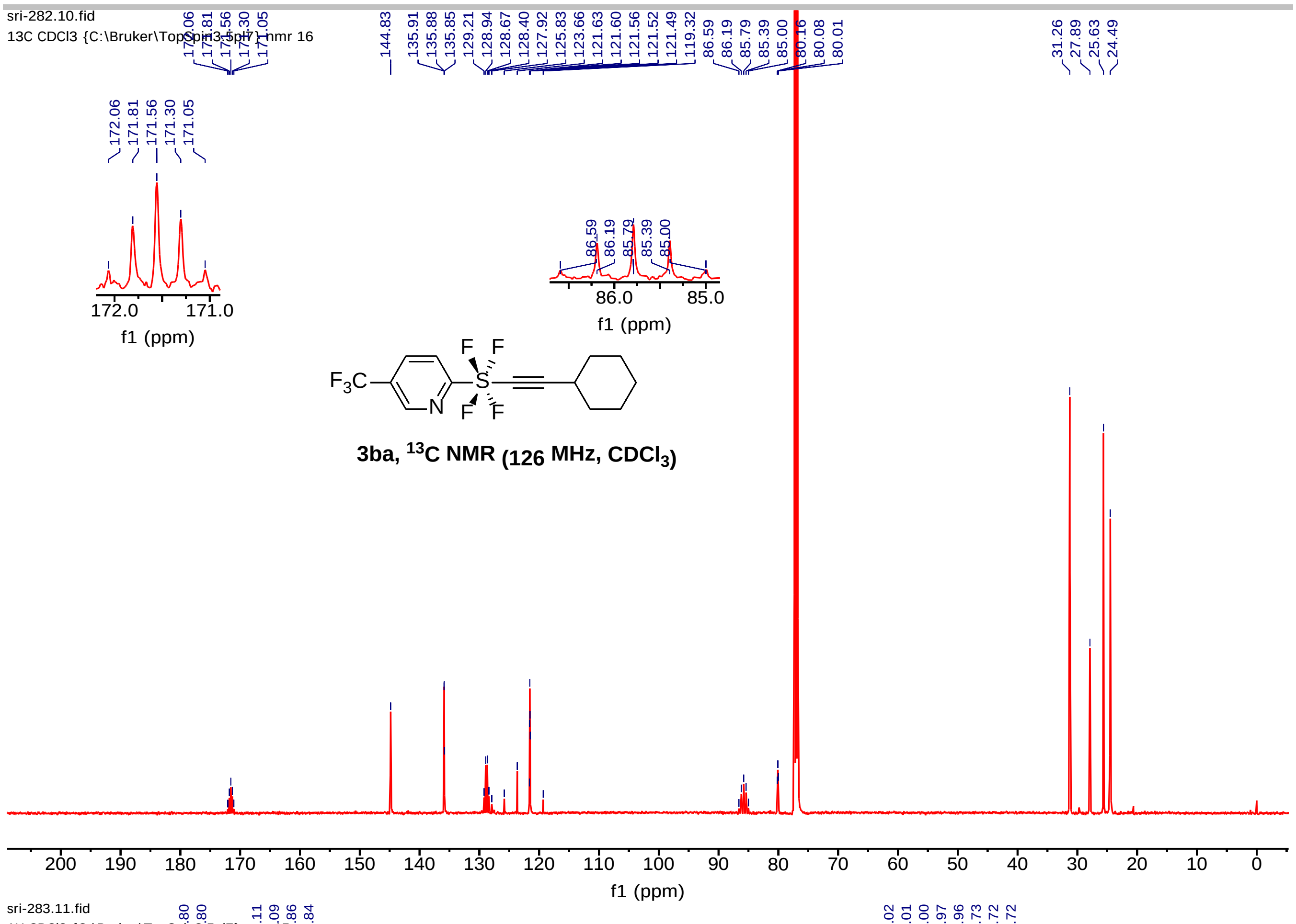
sri-282.10.fid
13C CDCl3 {C:\Bruker\TopSpin3.5pl7} nmr 16
f1 (ppm)
3ba, ^{13}C NMR (126 MHz, $CDCl_3$)

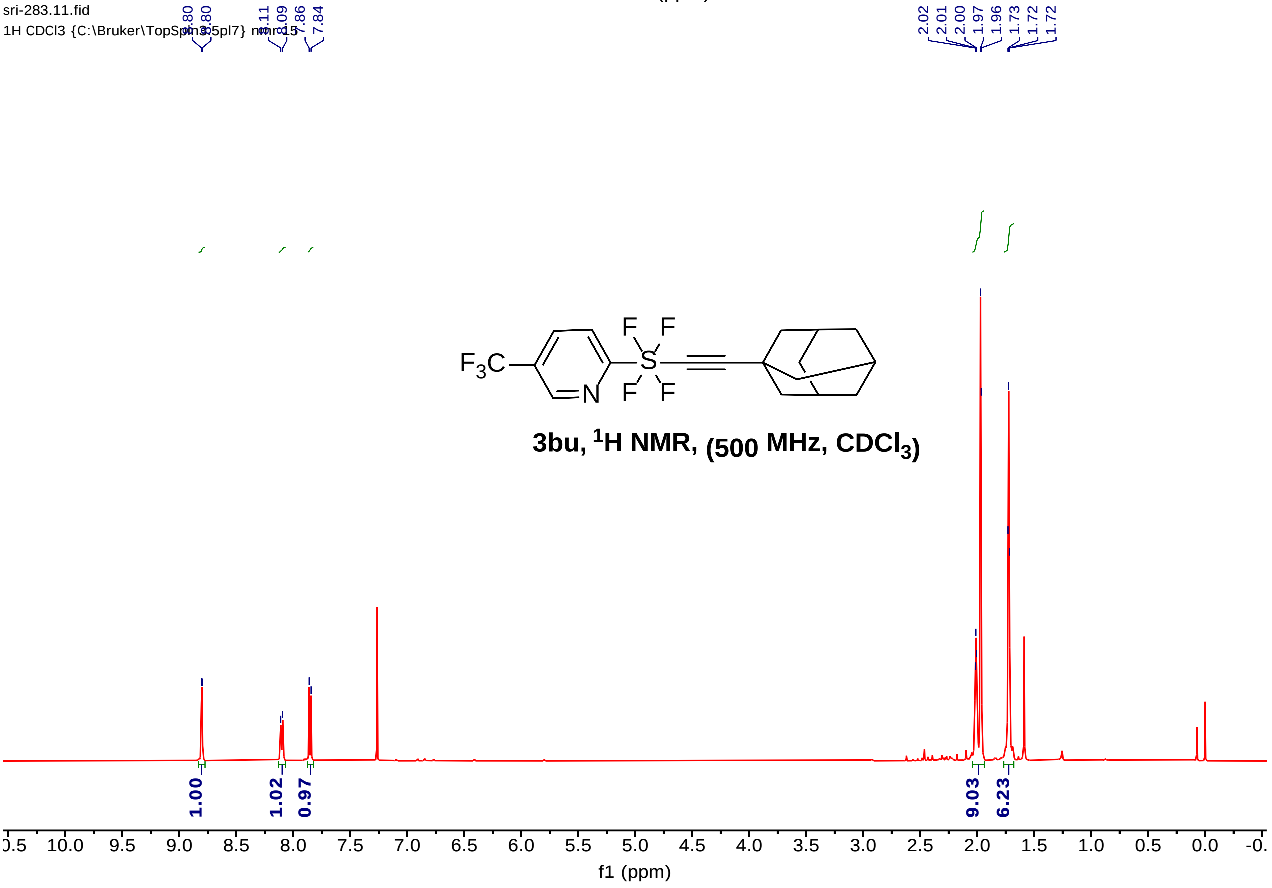
sri-283.11.fid
1H CDCl3 {C:\Bruker\TopSpin3.5pl7} nmr 15
1.00
1.02
0.97
9.03
6.23
f1 (ppm)
3bu, ^{1}H NMR, (500 MHz, $CDCl_3$)

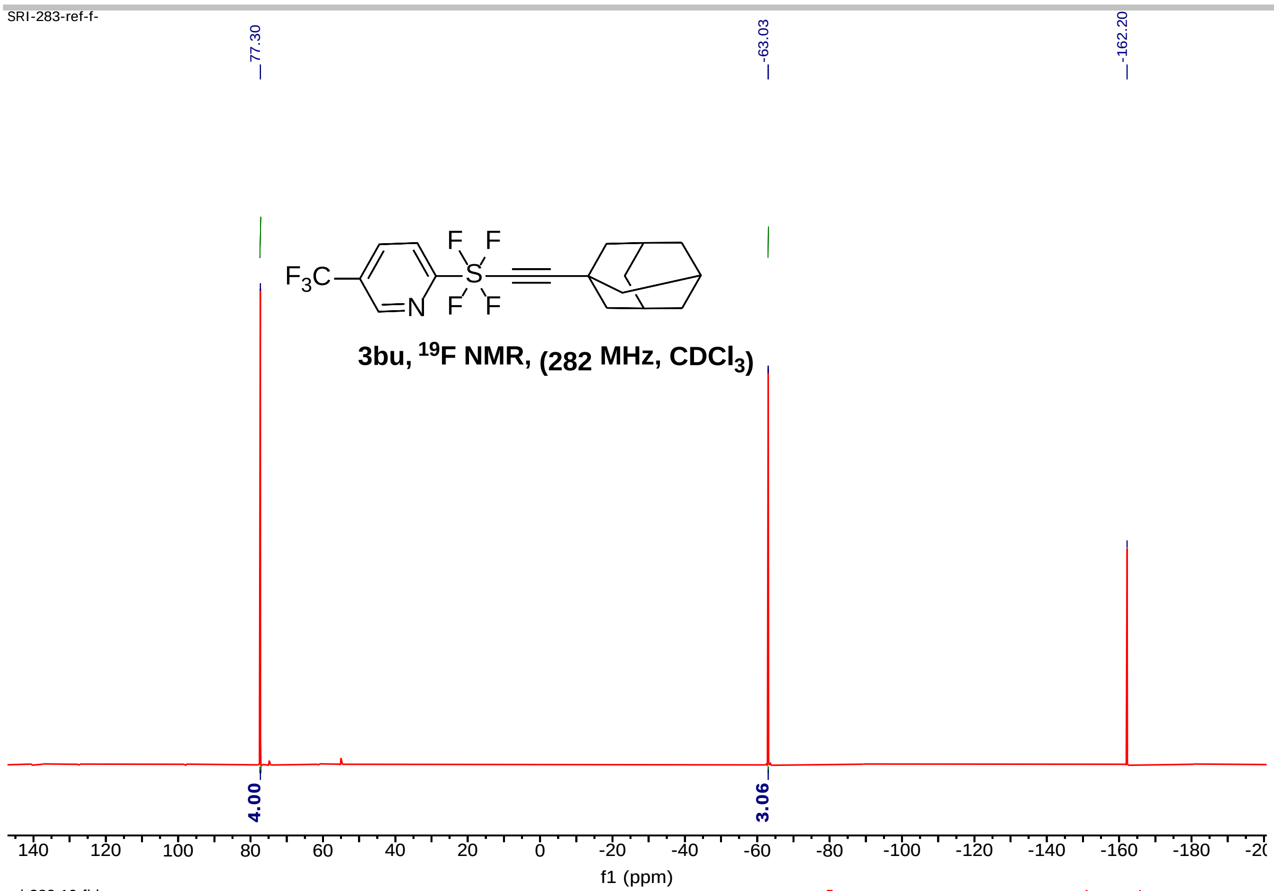

3bu, ^{19}F NMR, (282 MHz, $CDCl_3$)

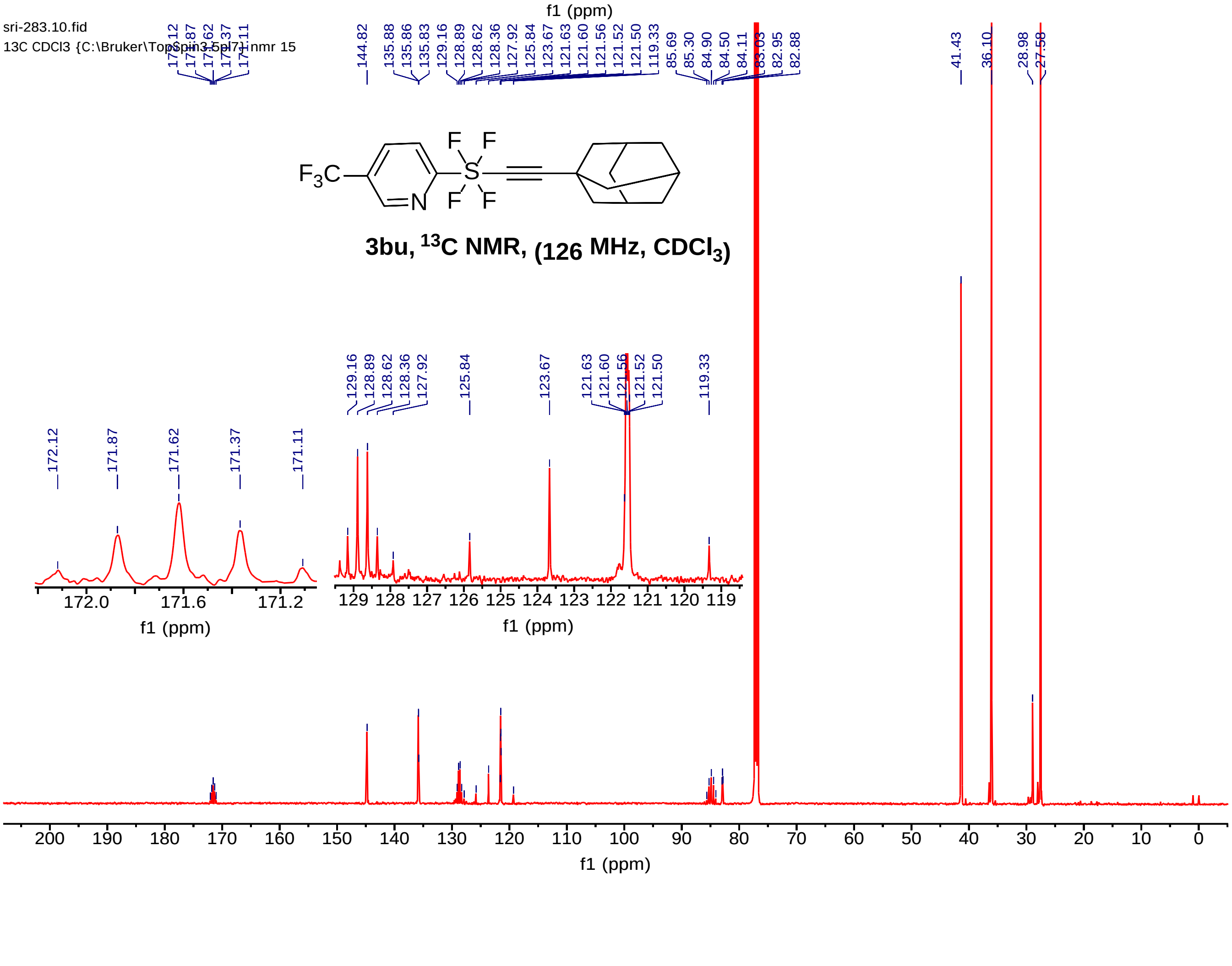

3bu, ^{13}C NMR, (126 MHz, $CDCl_3$)

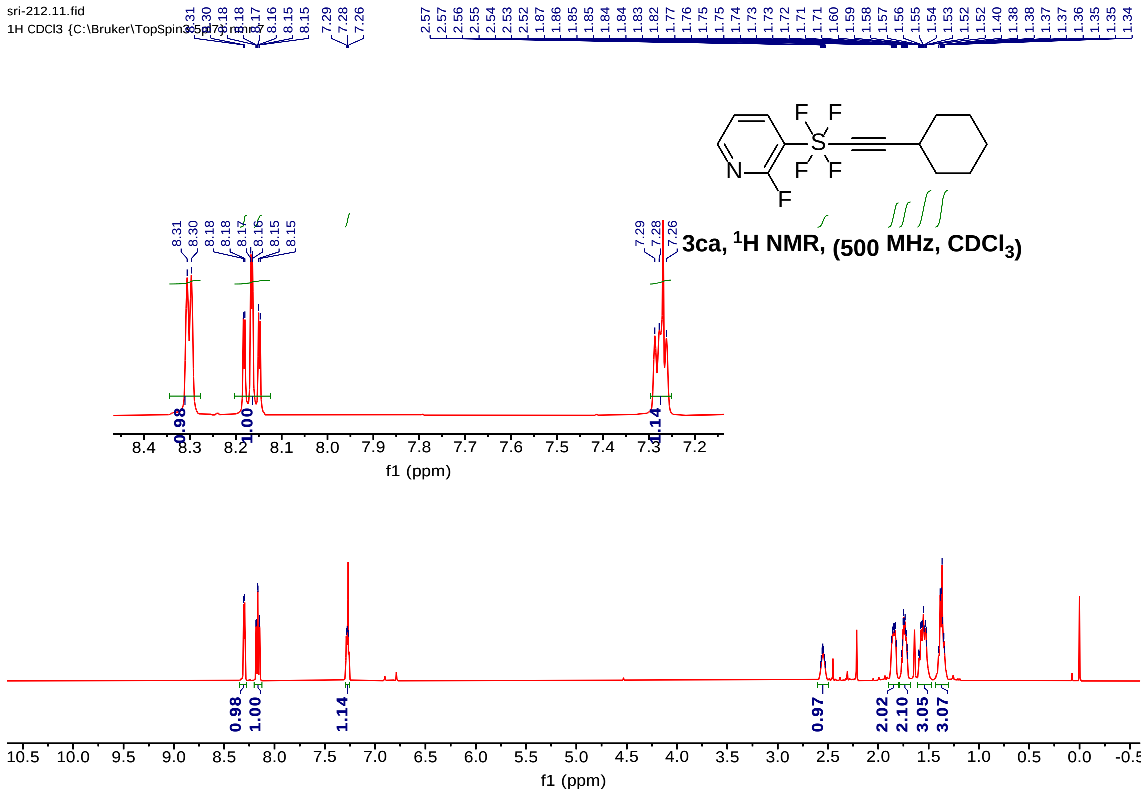
sri-212.11.fid
1H CDCl3 {C:\Bruker\TopSpin3.5pl7\nmr}
3ca, ¹H NMR, (500 MHz, CDCl3)
f1 (ppm)

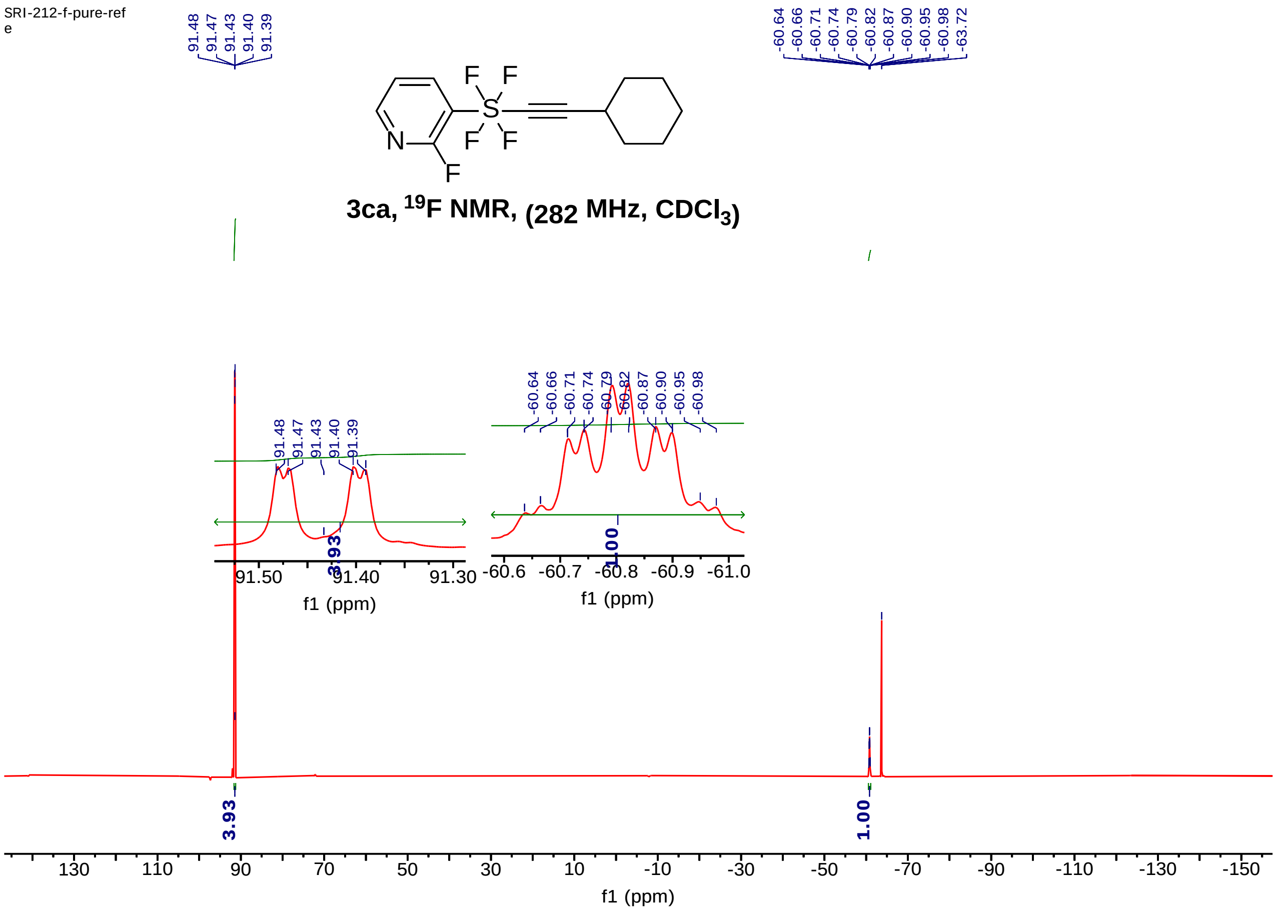
SRI-212-f-pure-ref
e
3ca, ¹⁹F NMR, (282 MHz, CDCl3)
f1 (ppm)

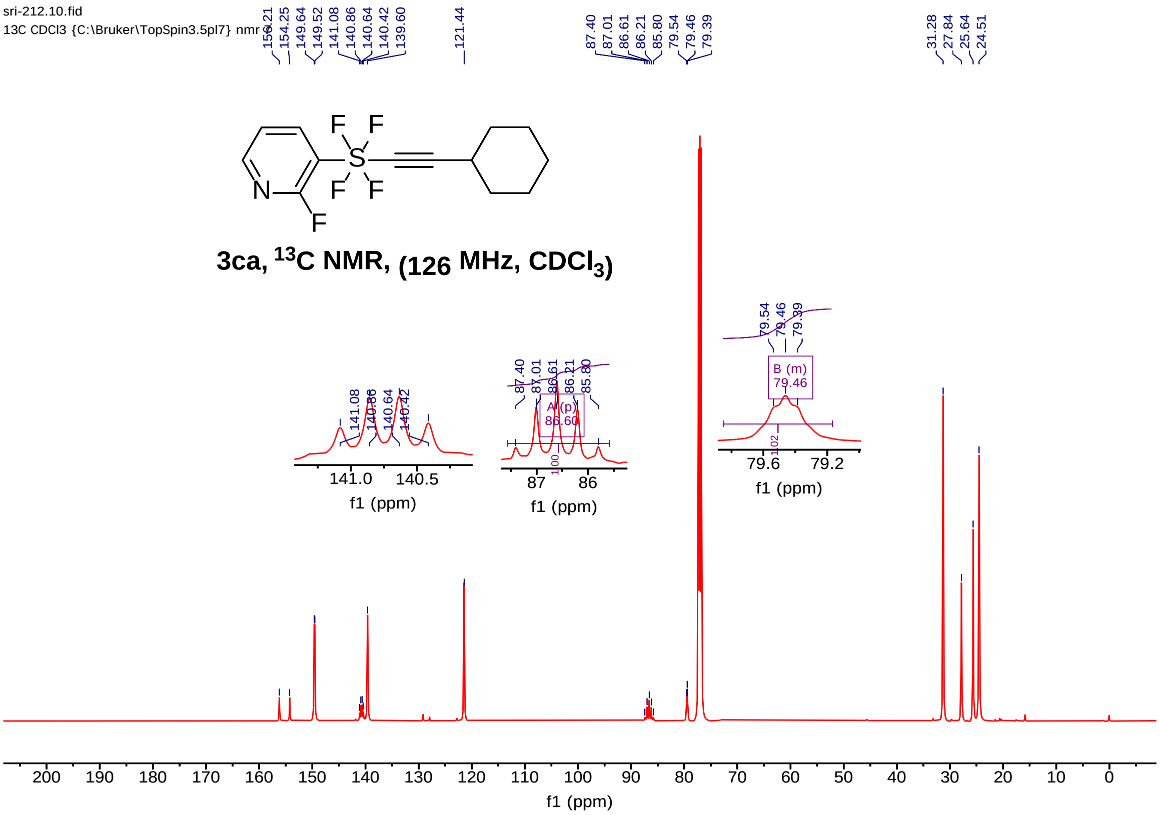
sri-212.10.fid
13C CDCl3 {C:\Bruker\TopSpin3.5pl7} nmr
3ca, ^{13}C NMR, (126 MHz, $CDCl_3$)
f1 (ppm)

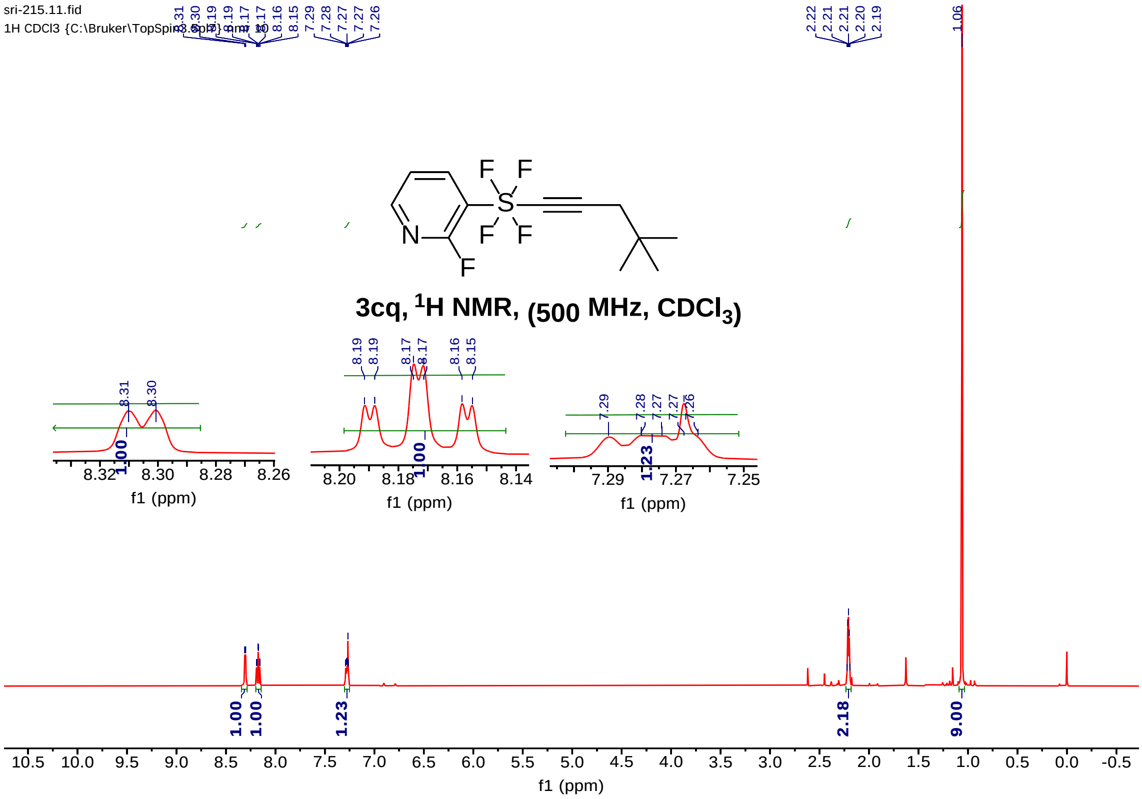
sri-215.11.fid
1H CDCl3 {C:\Bruker\TopSpin3.5pl7} nmr
3cq, ^{1}H NMR, (500 MHz, $CDCl_3$)
f1 (ppm)

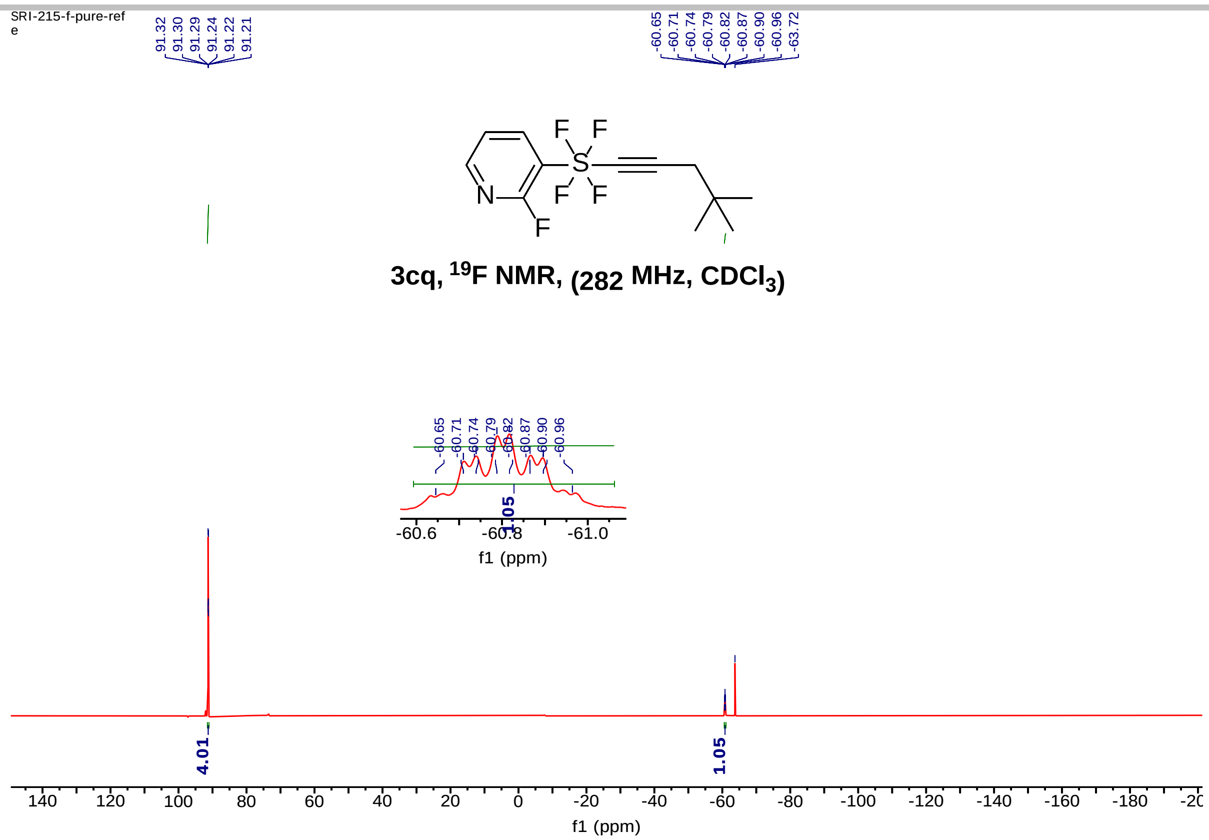
SRI-215-f-pure-ref
e
91.32
91.30
91.29
91.24
91.22
91.21
-60.65
-60.71
-60.74
-60.79
-60.82
-60.87
-60.90
-60.96
-63.72
N
F
F
F
F
F
S
3cq, 19F NMR, (282 MHz, CDCl3)
4.01
1.05
f1 (ppm)

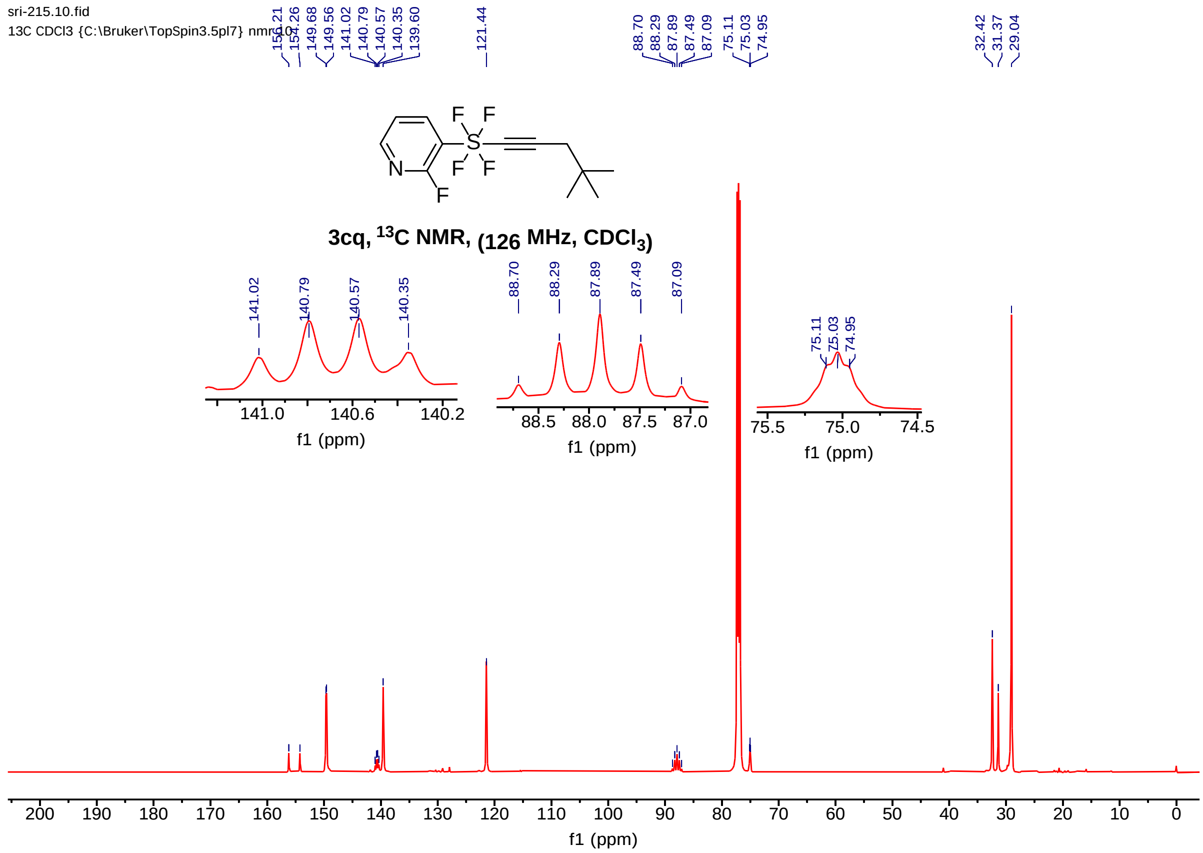
sri-215.10.fid
13C CDCl3 {C:\Bruker\TopSpin3.5pl7} nmr10
121.44
88.70
88.29
87.89
87.49
87.09
75.11
75.03
74.95
32.42
31.37
29.04
3cq, 13C NMR, (126 MHz, CDCl3)
141.02
140.79
140.57
140.35
f1 (ppm)

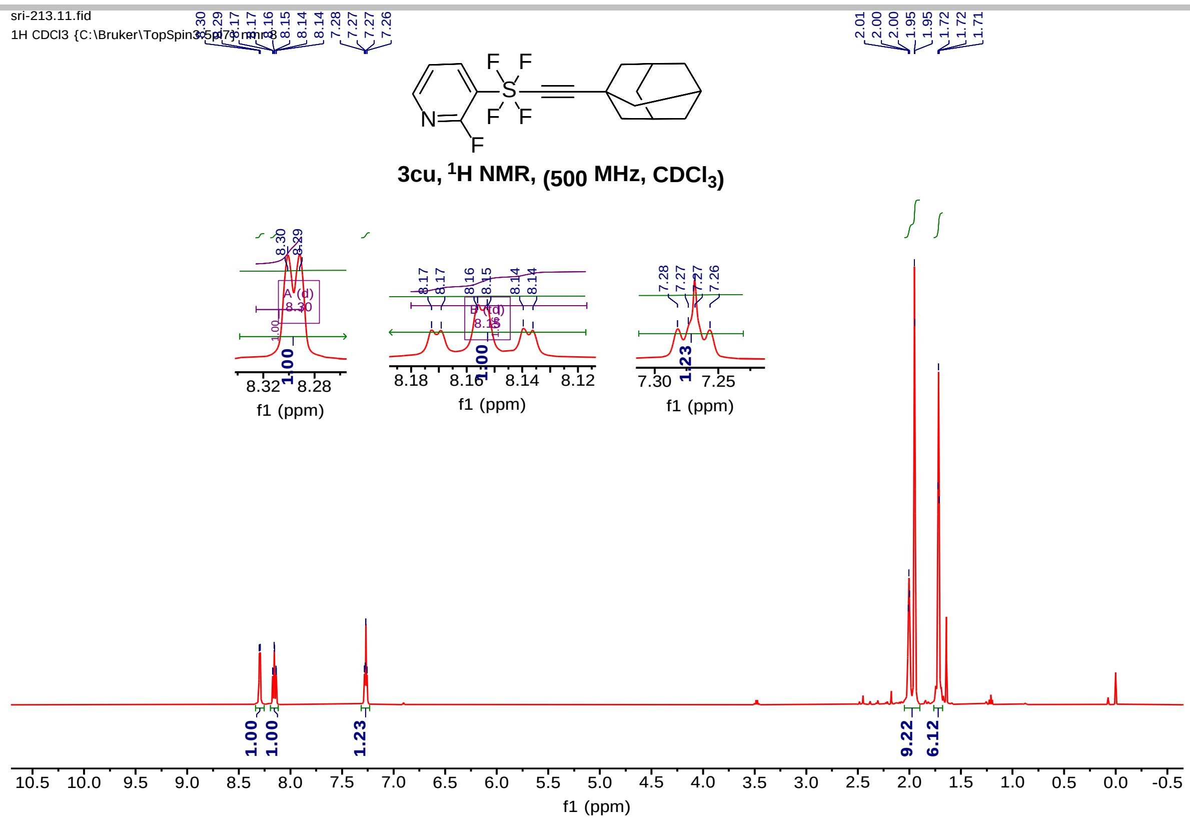
sri-213.11.fid
1H CDCl3 {C:\Bruker\TopSpin3.5pl7} nmr38
3cu, 1H NMR, (500 MHz, CDCl3)
f1 (ppm)

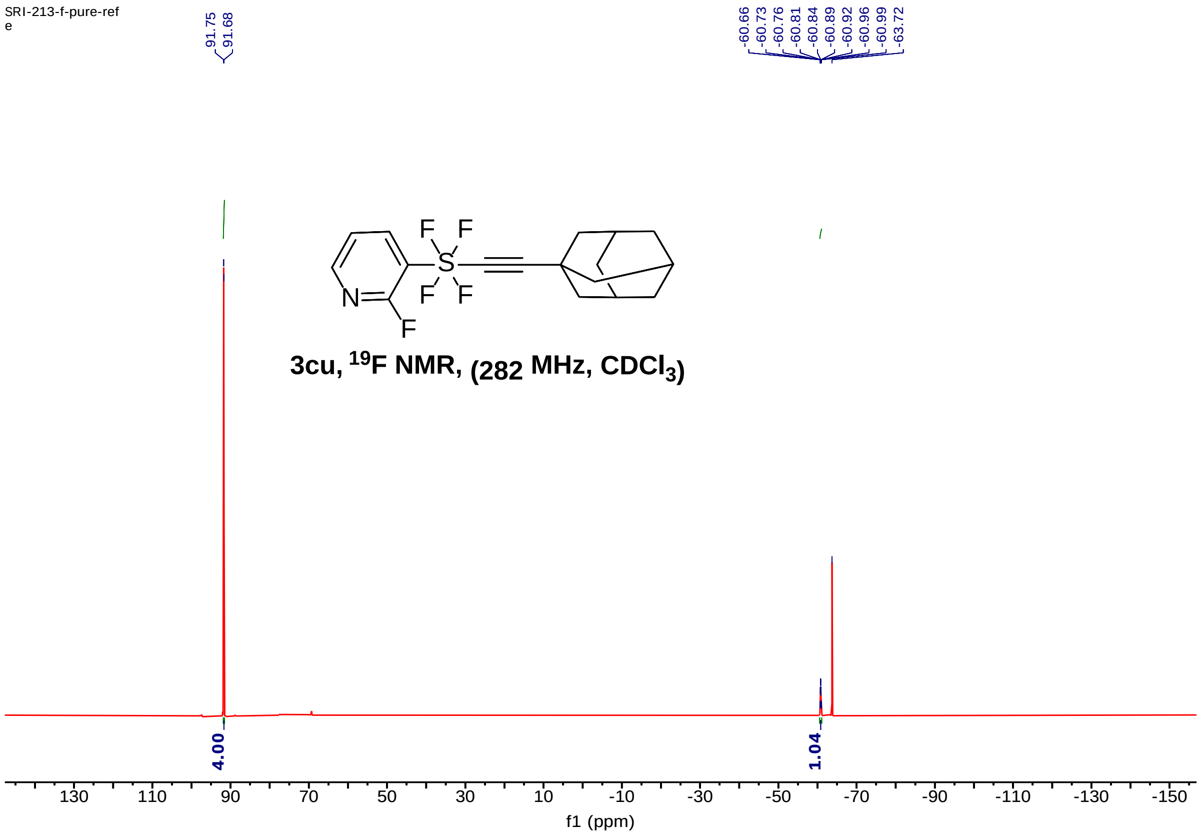
SRI-213-f-pure-ref
e
3cu, 19F NMR, (282 MHz, CDCl3)
f1 (ppm)

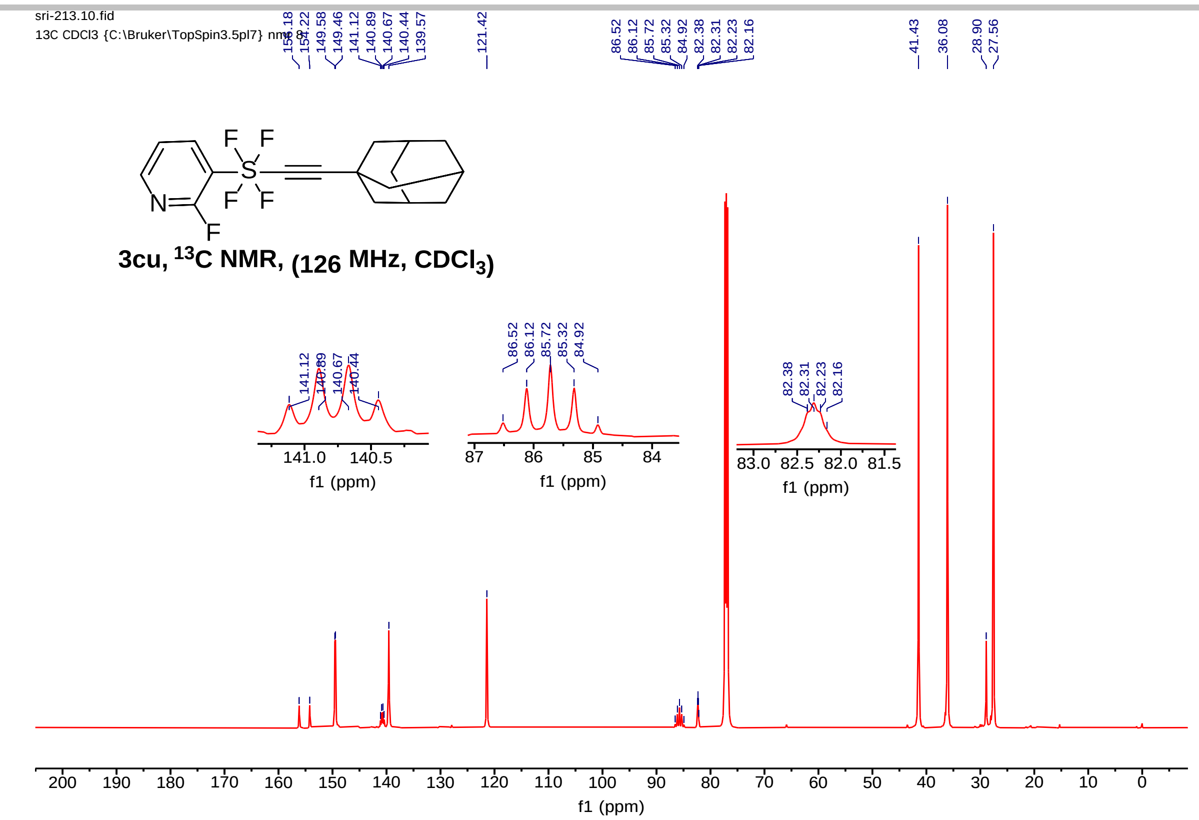
sri-213.10.fid
13C CDCl3 {C:\Bruker\TopSpin3.5pl7} nmr 8
155.18
154.22
149.58
149.46
141.12
140.89
140.67
140.44
139.57
121.42
86.52
86.12
85.72
85.32
84.92
82.38
82.31
82.23
82.16
41.43
36.08
28.90
27.56
3cu, 13C NMR, (126 MHz, CDCl3)
f1 (ppm)

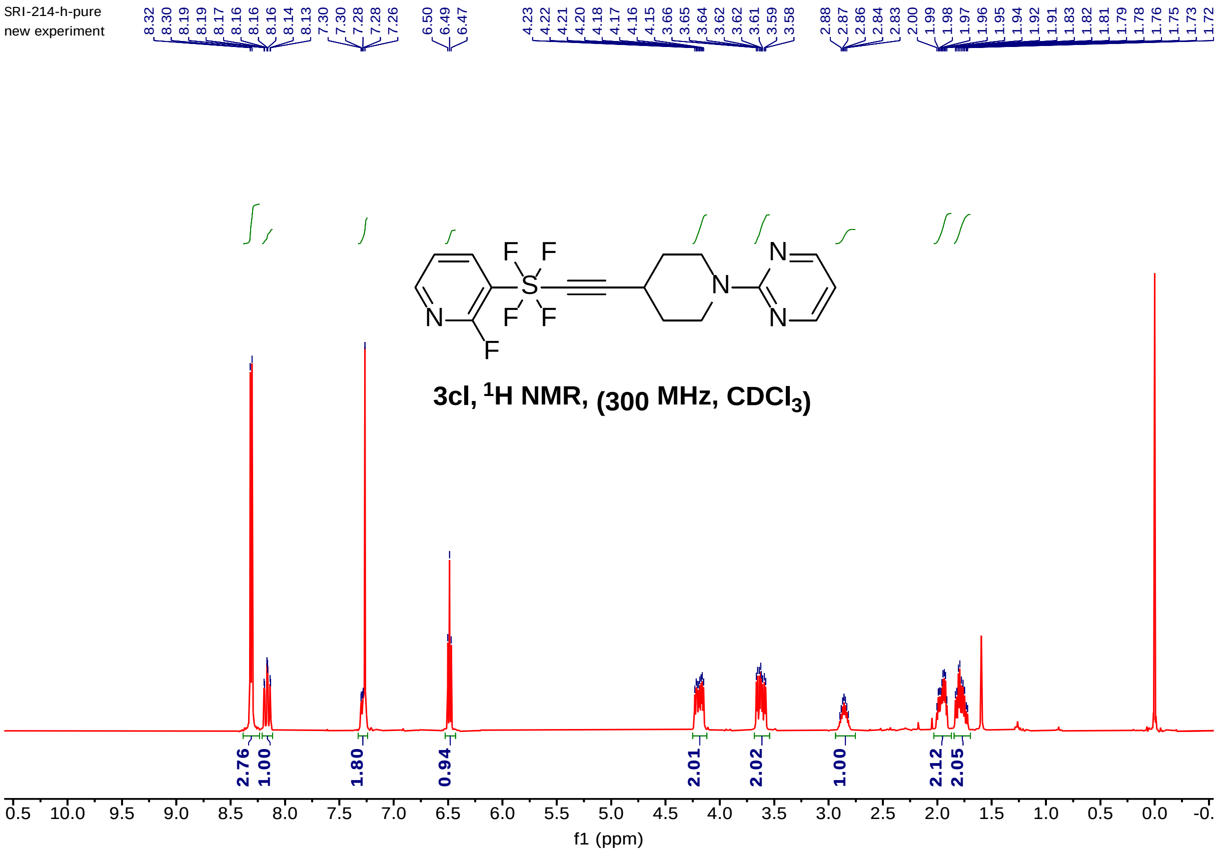
SRI-214-h-pure
new experiment
3cl, 1H NMR, (300 MHz, CDCl3)
2.76
1.00
1.80
0.94
2.01
2.02
1.00
2.12
2.05
f1 (ppm)

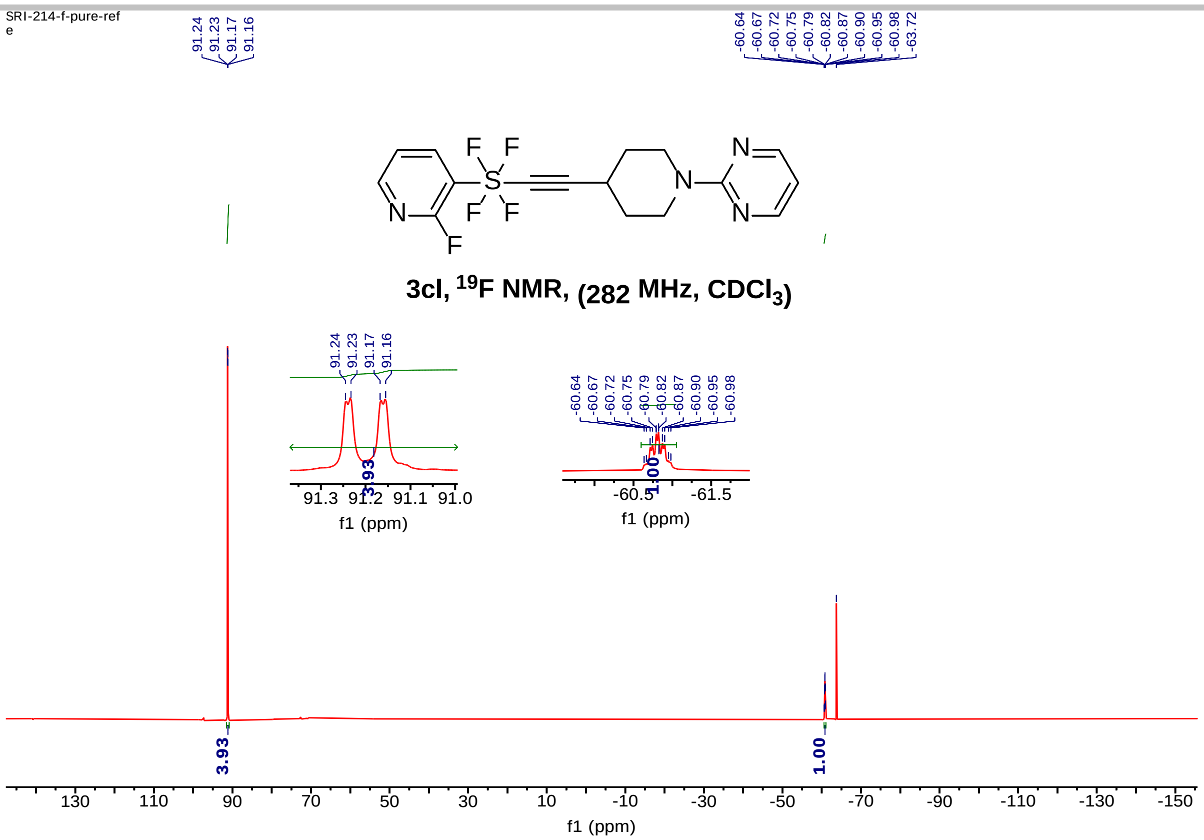
SRI-214-f-pure-ref
e
91.24
91.23
91.17
91.16
-60.64
-60.67
-60.72
-60.75
-60.79
-60.82
-60.87
-60.90
-60.95
-60.98
-63.72
3cl, ^{19}F NMR, (282 MHz, $CDCl_3$)
3.93
1.00
f1 (ppm)

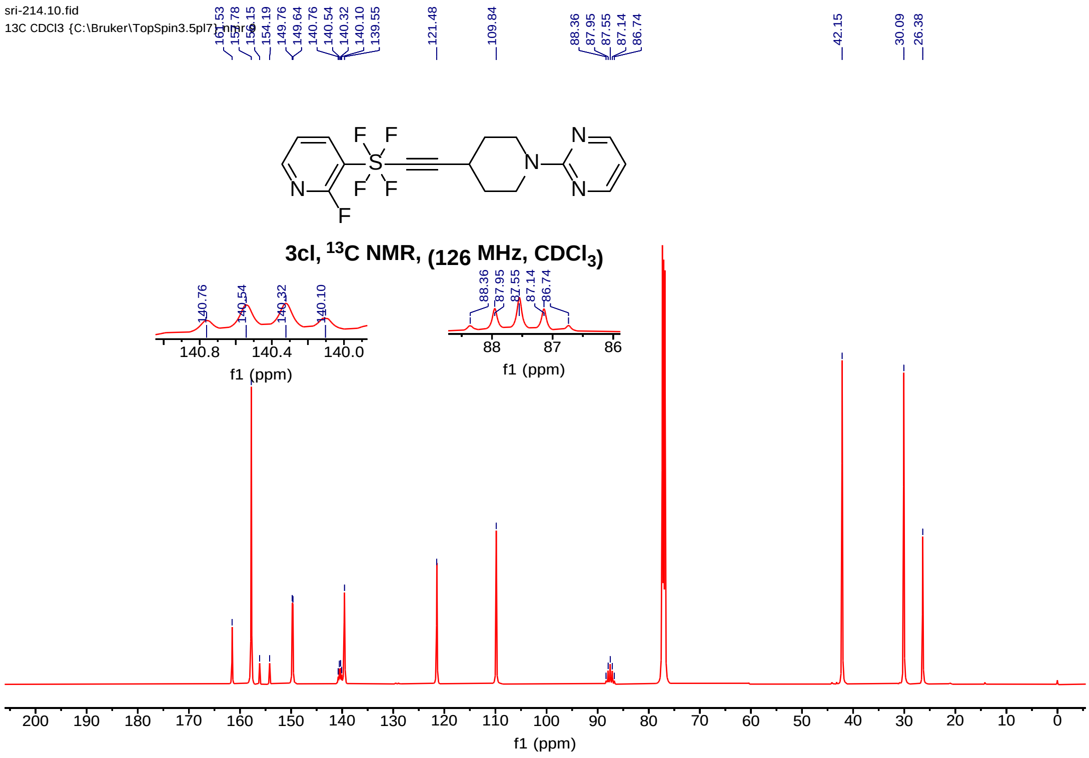
sri-214.10.fid
13C CDCl3 {C:\Bruker\TopSpin3.5pl7} nmr
154.19
149.76
149.64
140.76
140.54
140.32
140.10
139.55
121.48
109.84
88.36
87.95
87.55
87.14
86.74
42.15
30.09
26.38
3cl, ^{13}C NMR, (126 MHz, $CDCl_3$)
f1 (ppm)

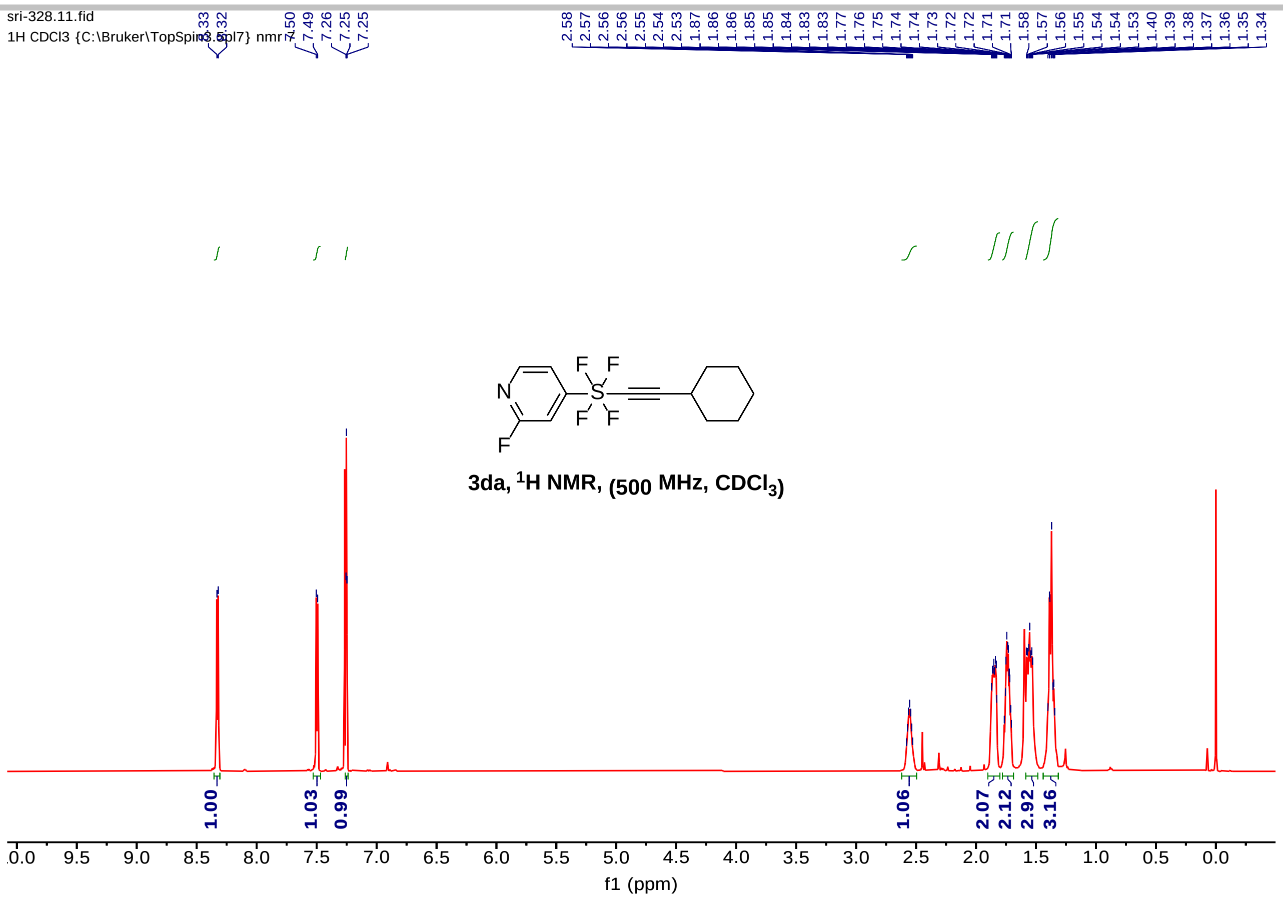

3da, ^{1}H NMR, (500 MHz, $CDCl_3$)

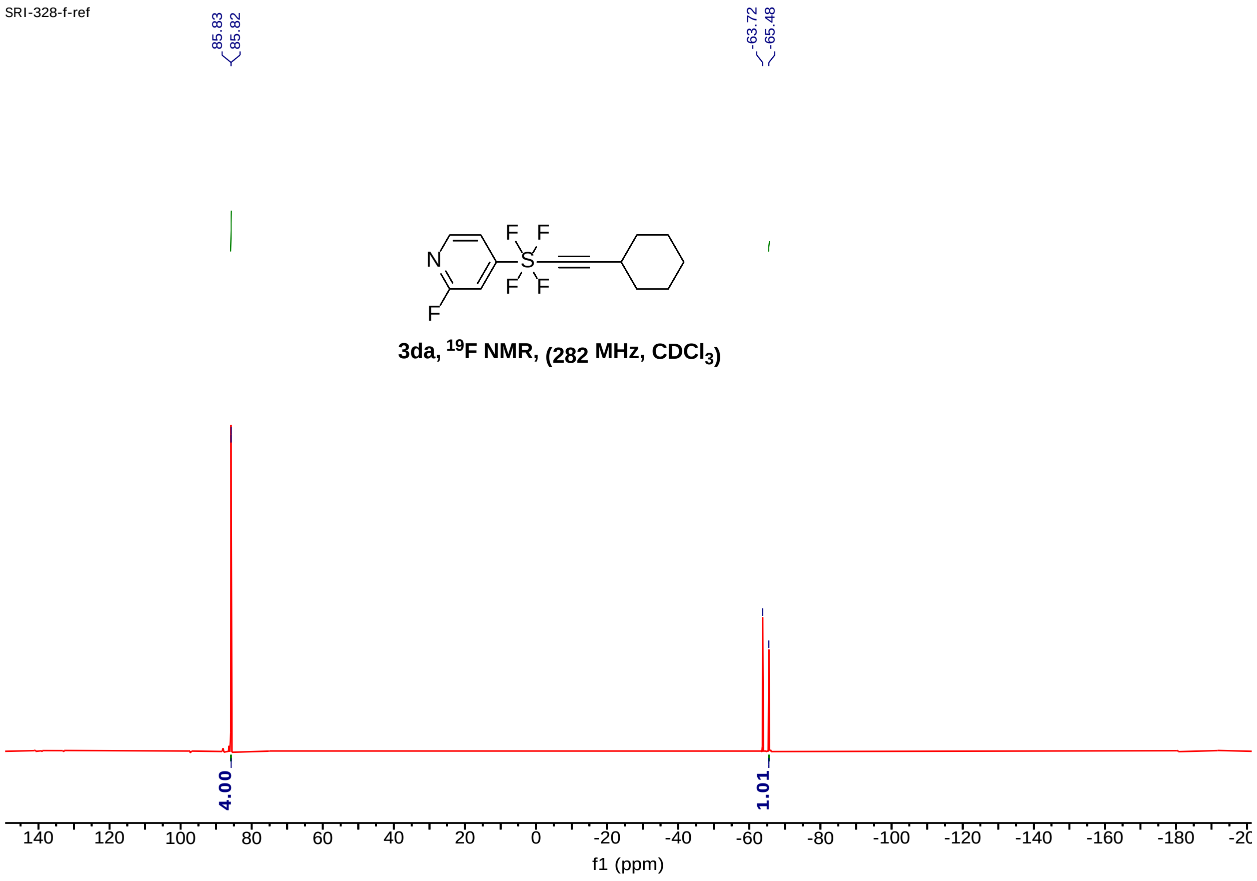

3da, ^{19}F NMR, (282 MHz, $CDCl_3$)

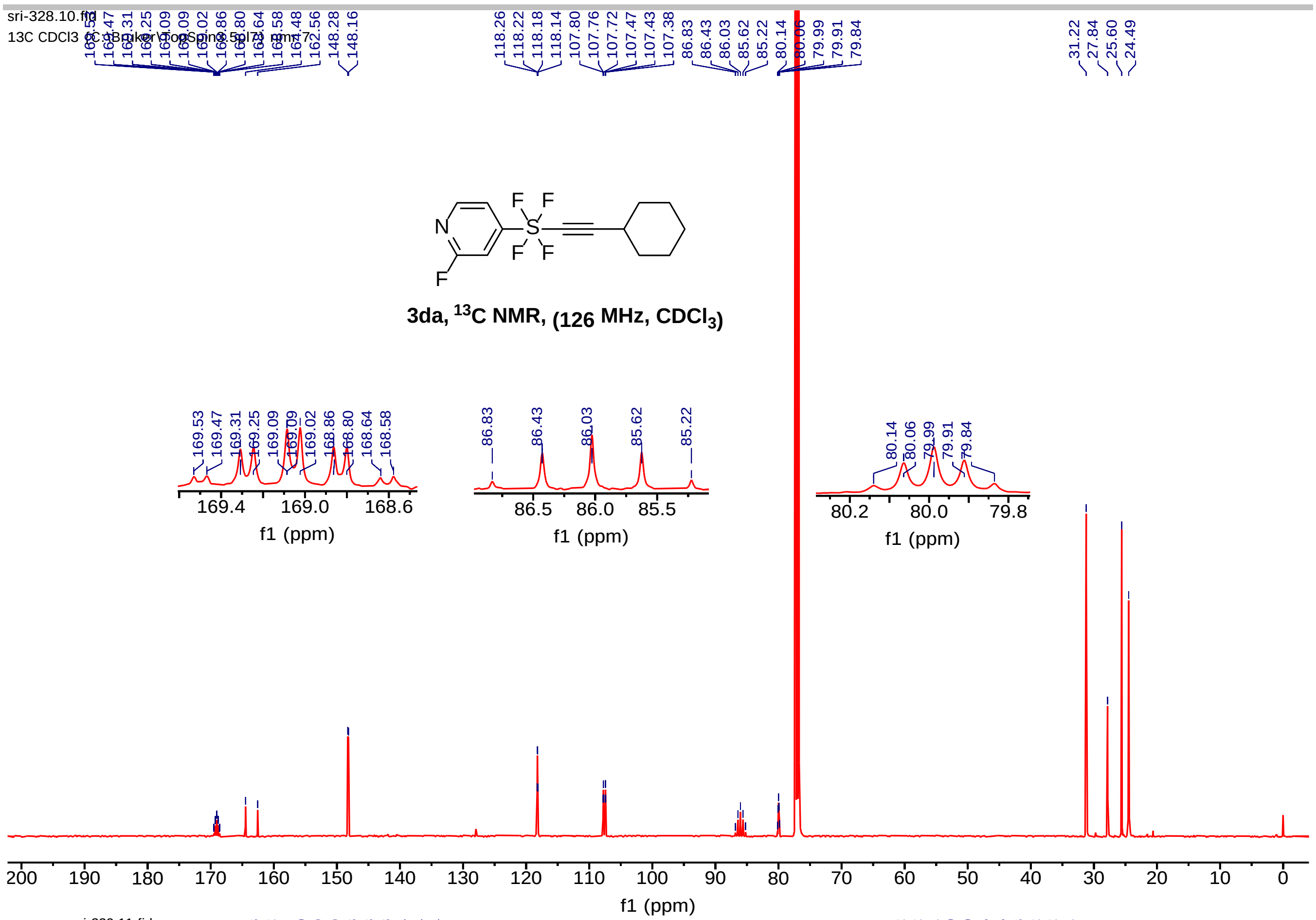
sri-328.10.fid
13C CDCl3 {C:\Bruker\TopSpin3.5pl7} nmr 7
3da, ^{13}C NMR, (126 MHz, $CDCl_3$)
f1 (ppm)

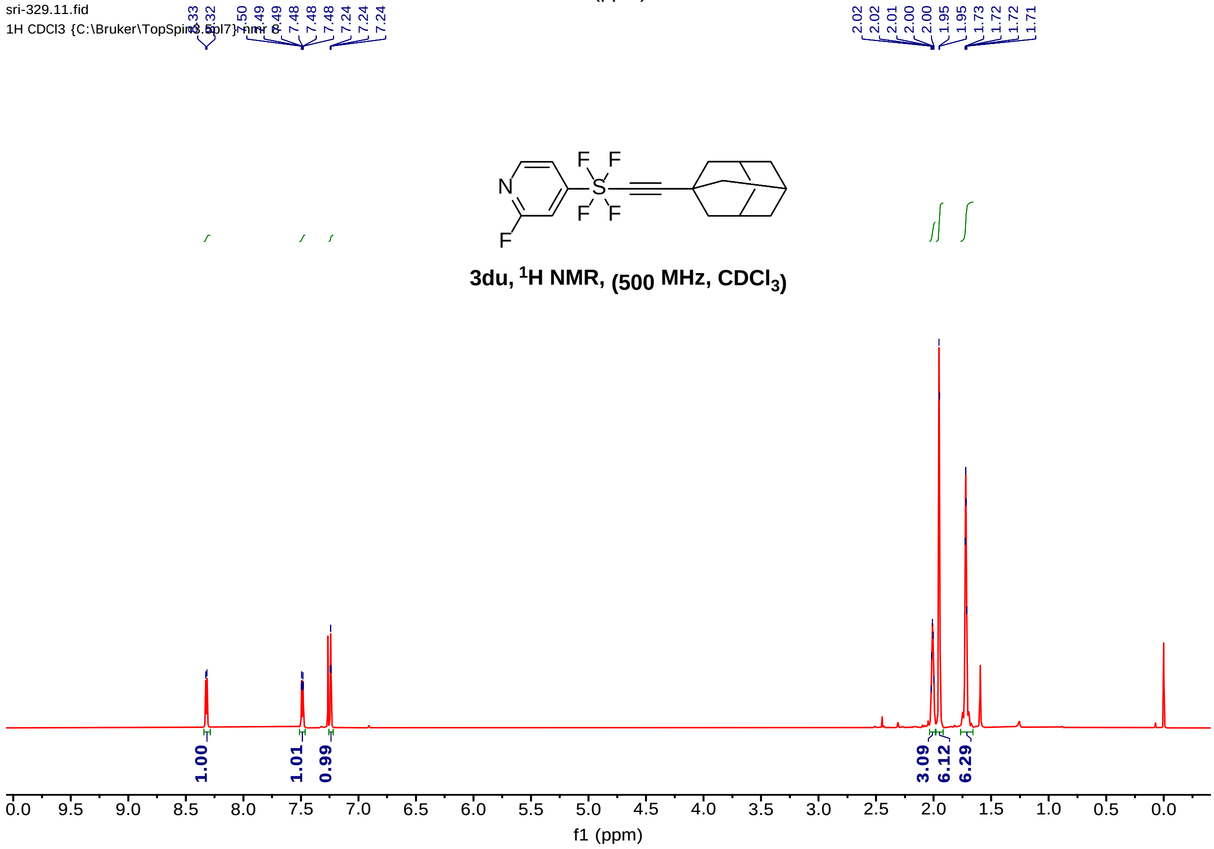
sri-329.11.fid
1H CDCl3 {C:\Bruker\TopSpin3.5pl7} nmr 8
3du, ^{1}H NMR, (500 MHz, $CDCl_3$)
1.00
1.01
0.99
3.09
6.12
6.29
f1 (ppm)

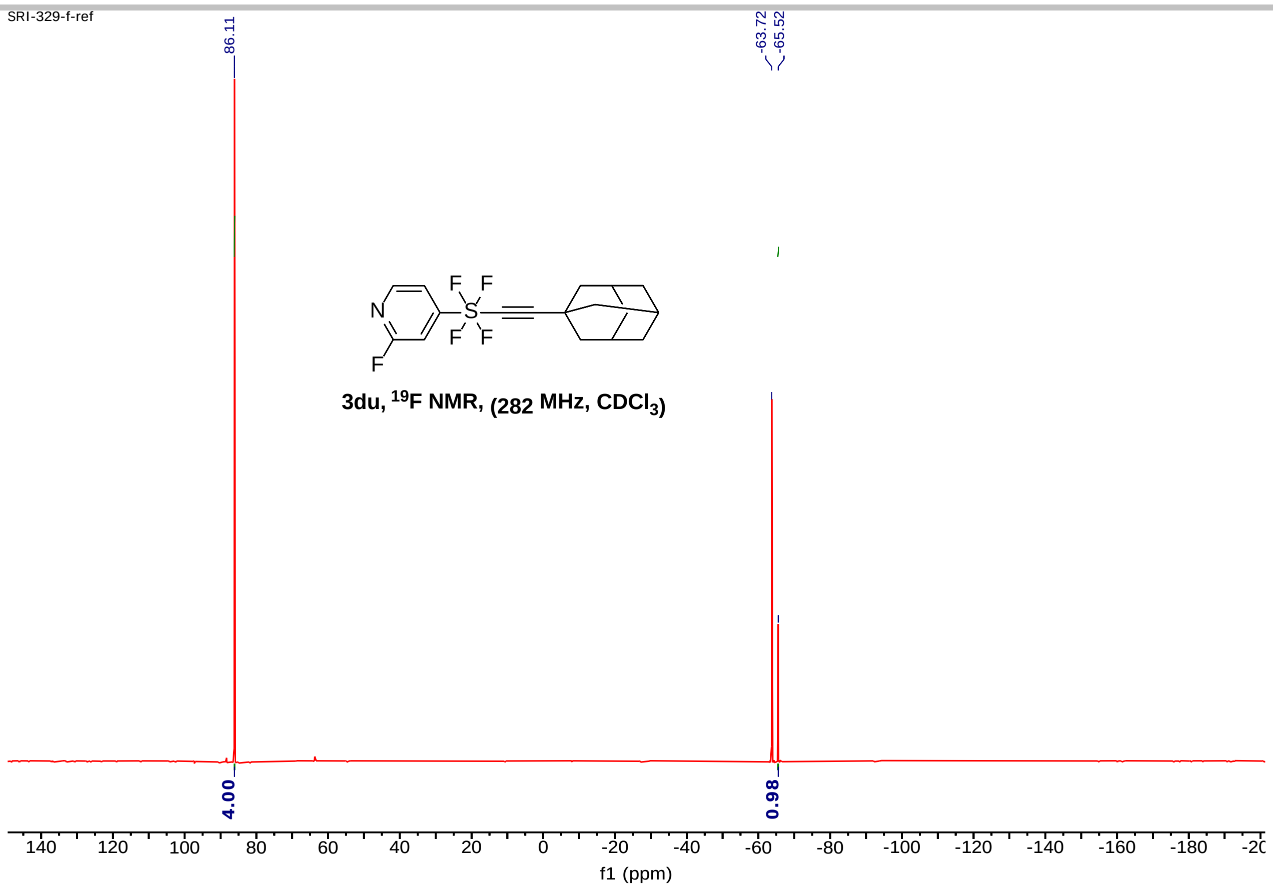
SRI-329-f-ref
86.11
-63.72
-65.52
3du, ^{19}F NMR, (282 MHz, CDCl$_3$)
4.00
0.98
f1 (ppm)

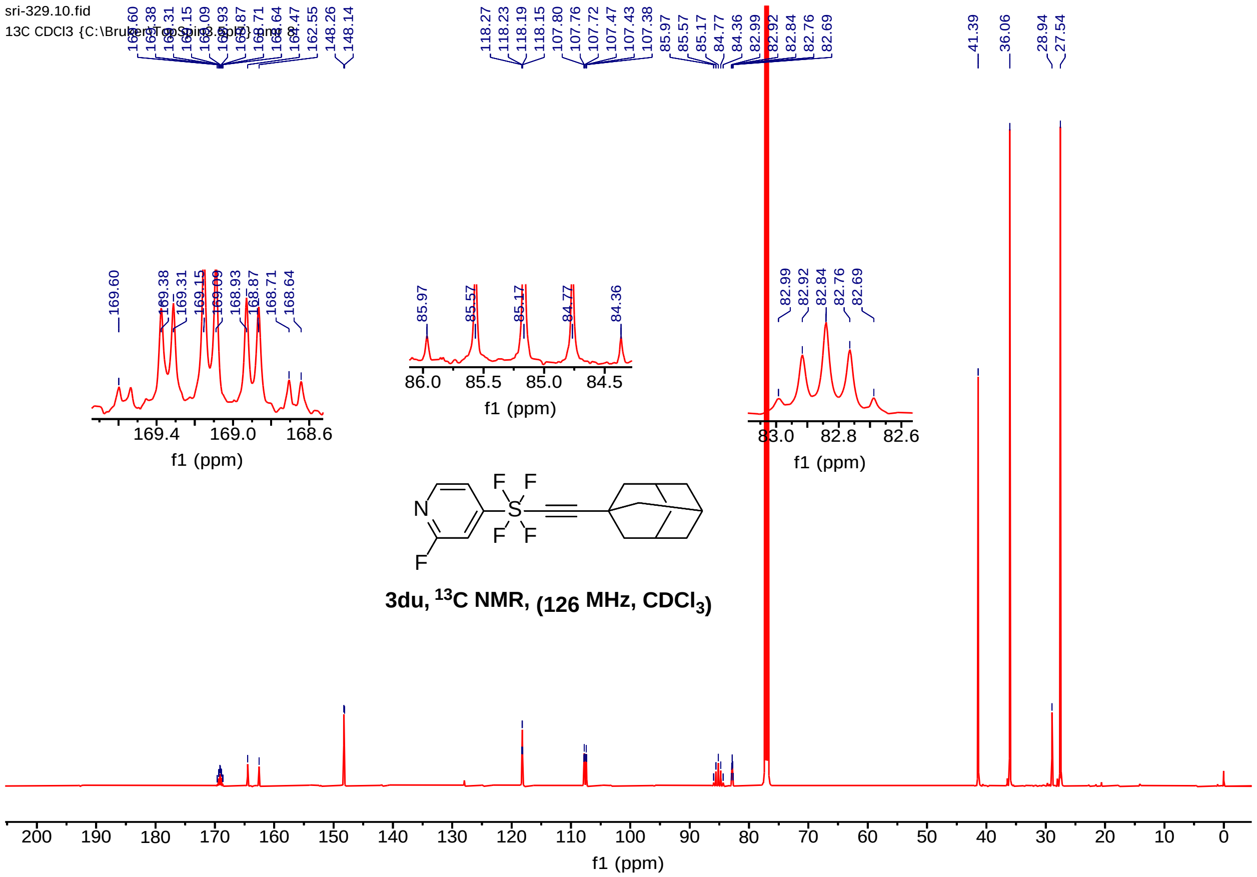
sri-329.10.fid
13C CDCl3 {C:\Bruker\TopSpin3.5pl7} cms 6
148.26
148.14
118.27
118.23
118.19
118.15
107.80
107.76
107.72
107.47
107.43
107.38
85.97
85.57
85.17
84.77
84.36
82.99
82.92
82.84
82.76
82.69
41.39
36.06
28.94
27.54
3du, ^{13}C NMR, (126 MHz, CDCl$_3$)
f1 (ppm)

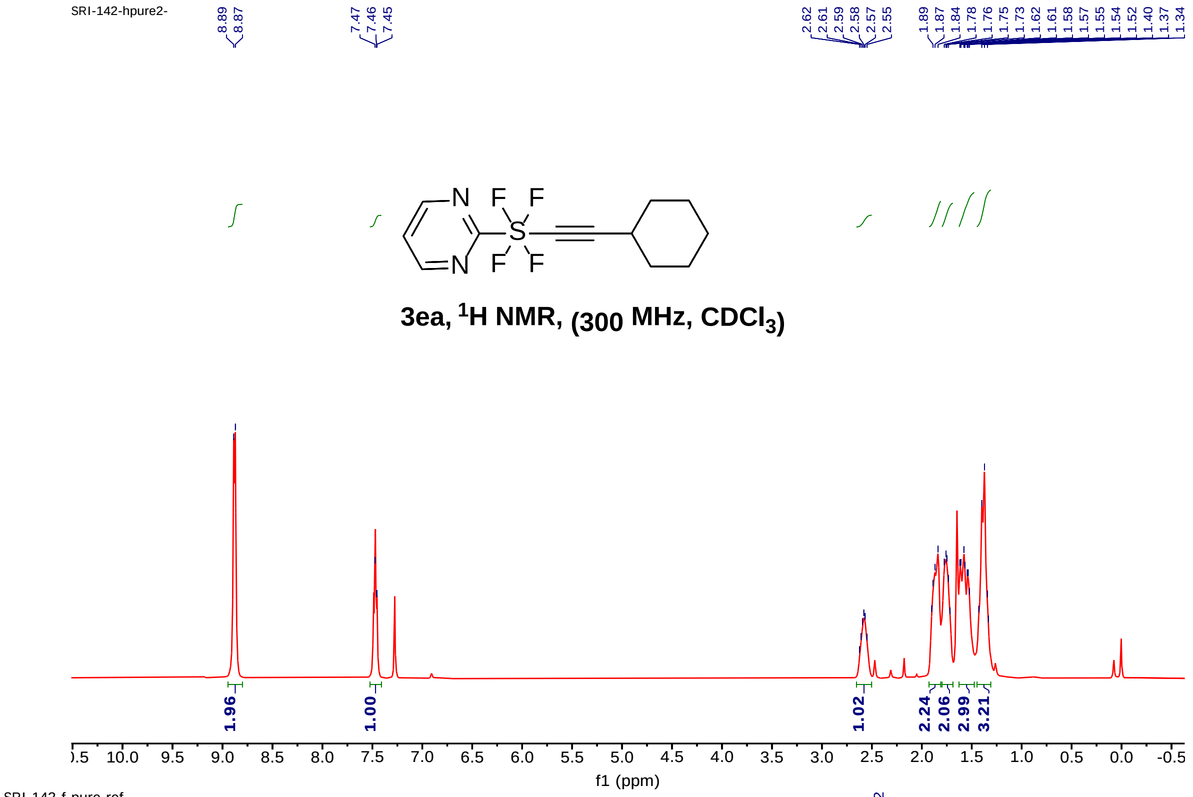

3ea, ^{1}H NMR, (300 MHz, $CDCl_3$)

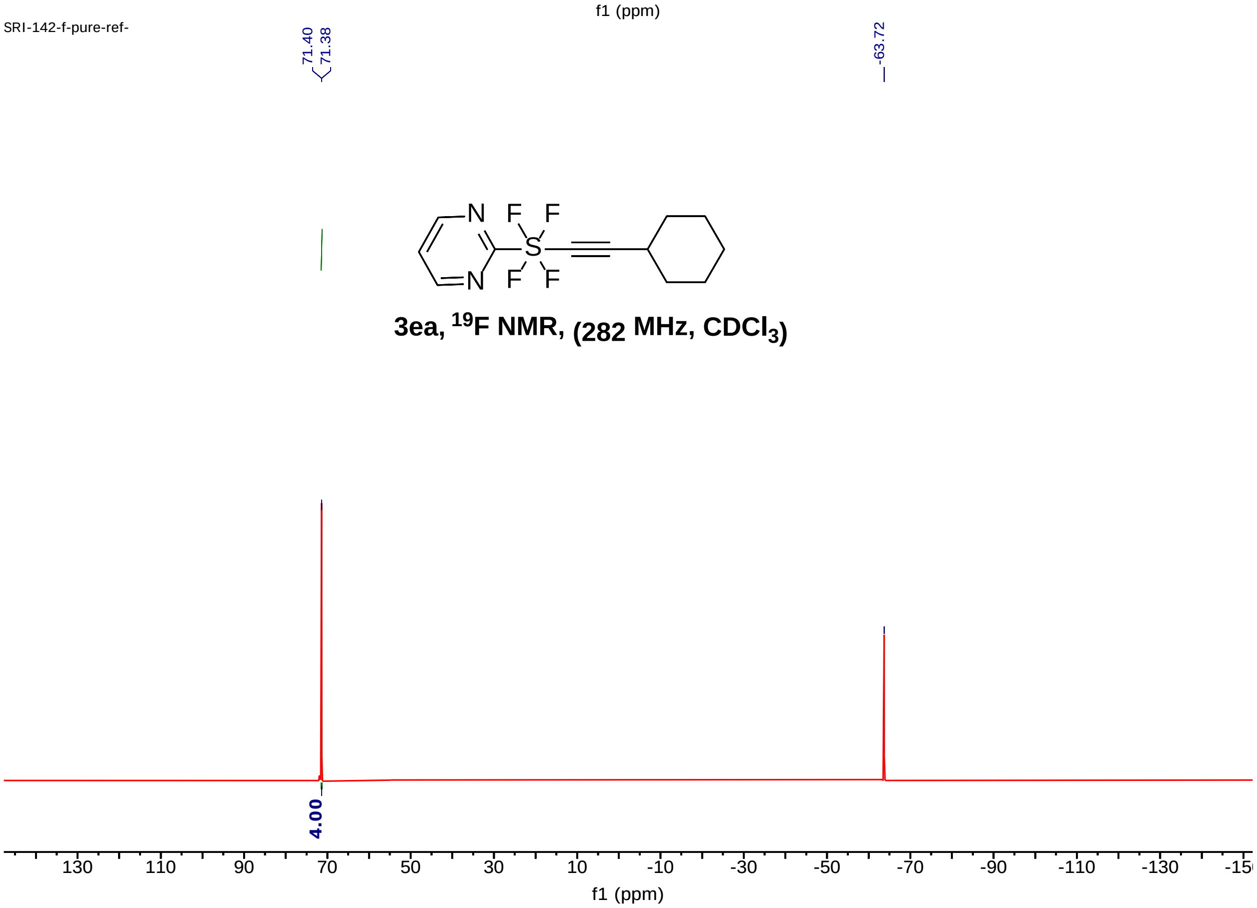

3ea, ^{19}F NMR, (282 MHz, $CDCl_3$)

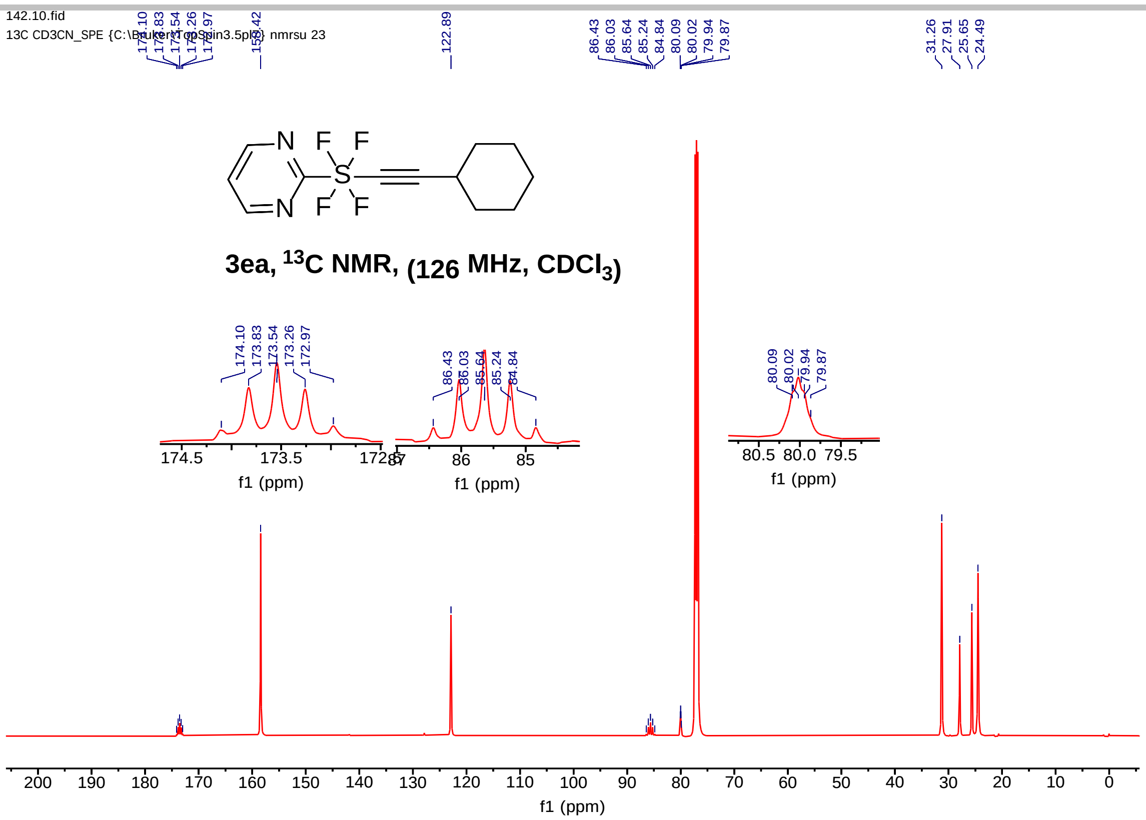
142.10.fid
13C CD3CN_SPE {C:\Bruker\TopSpin3.5pl2} nmrsu 23
3ea, 13C NMR, (126 MHz, CDCl3)
f1 (ppm)

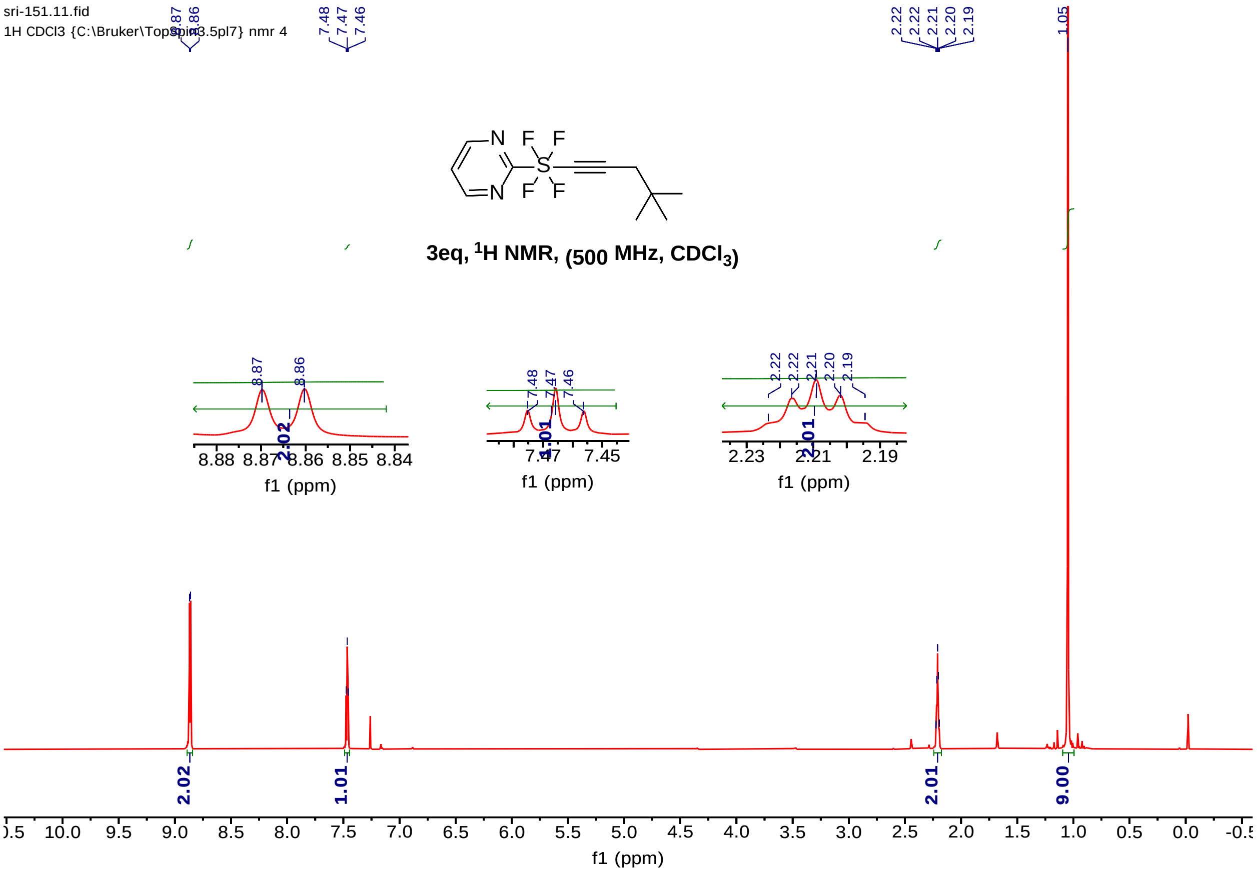
sri-151.11.fid
1H CDCl3 {C:\Bruker\TopSpin3.5pl7} nmr 4
3eq, 1H NMR, (500 MHz, CDCl3)
f1 (ppm)

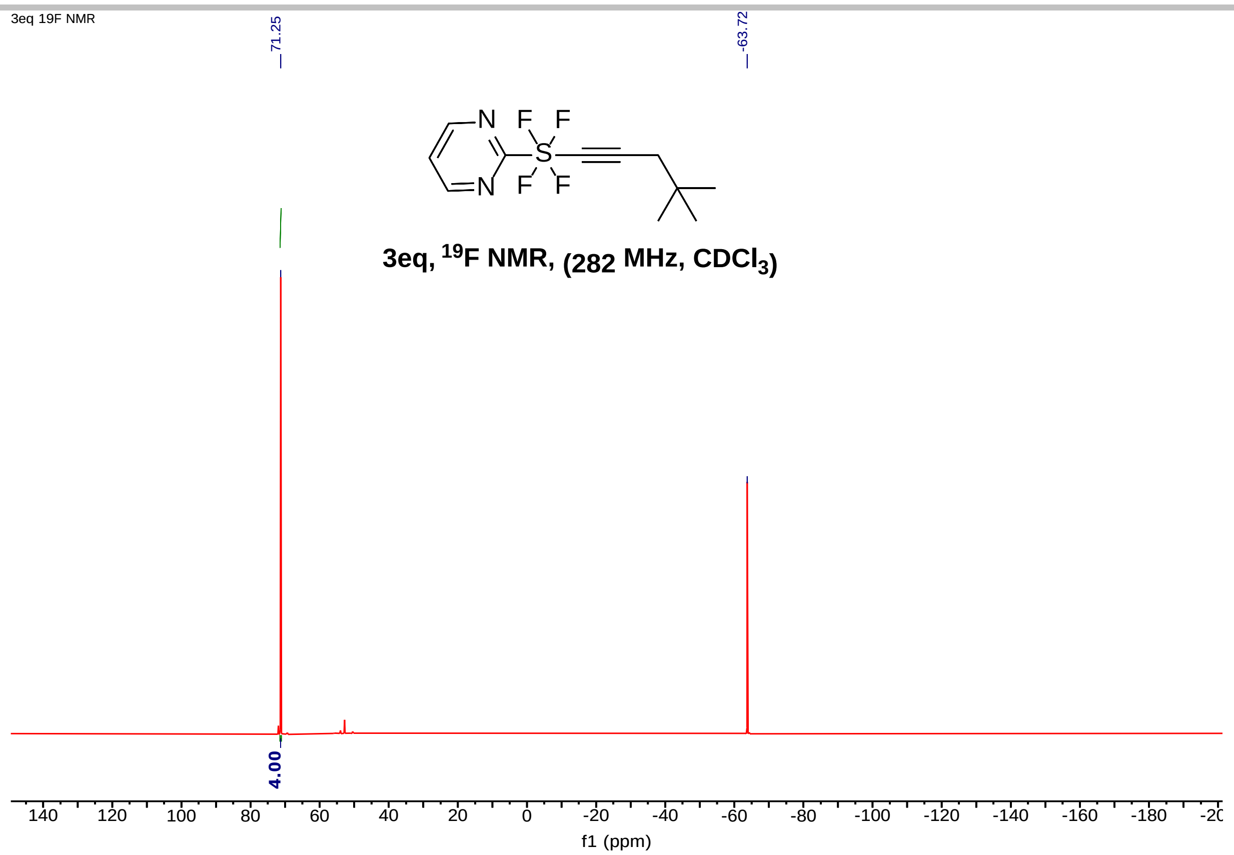
3eq 19F NMR
71.25
-63.72
3eq, 19F NMR, (282 MHz, CDCl3)
4.00
f1 (ppm)

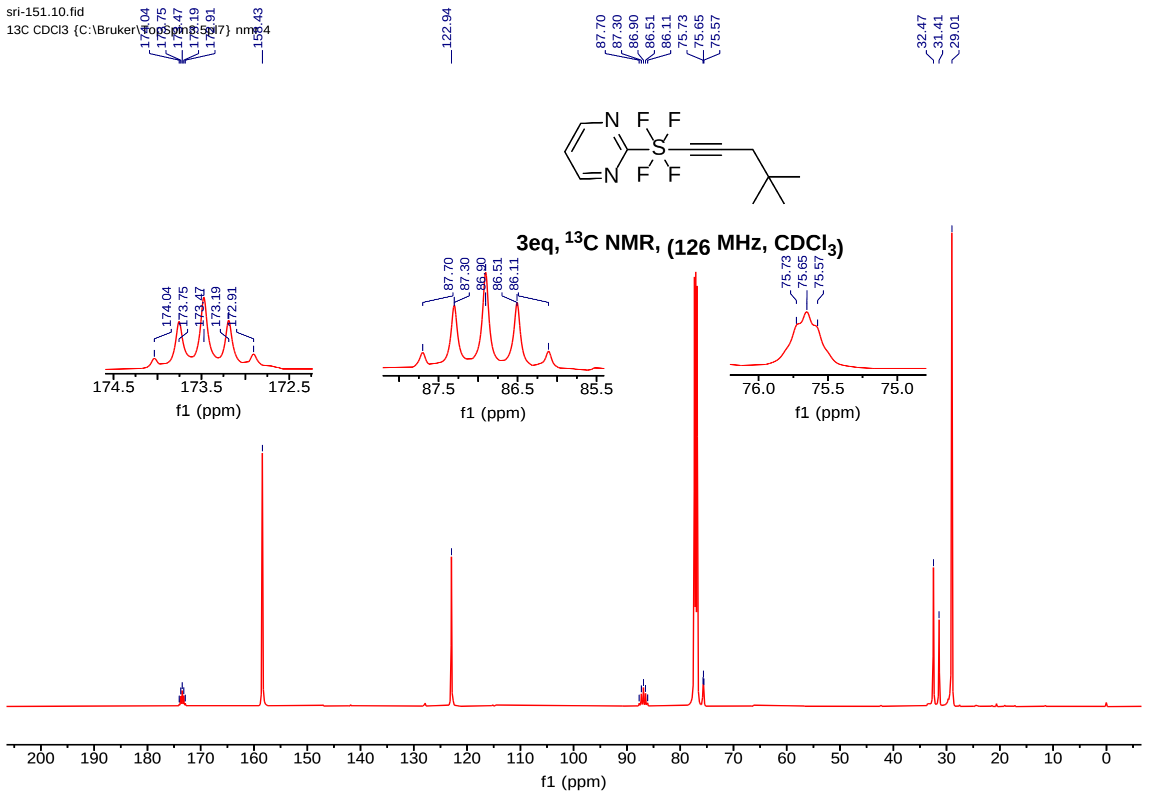
sri-151.10.fid
13C CDCl3
174.04
173.75
173.47
173.19
172.91
158.43
122.94
87.70
87.30
86.90
86.51
86.11
75.73
75.65
75.57
32.47
31.41
29.01
3eq, 13C NMR, (126 MHz, CDCl3)
f1 (ppm)

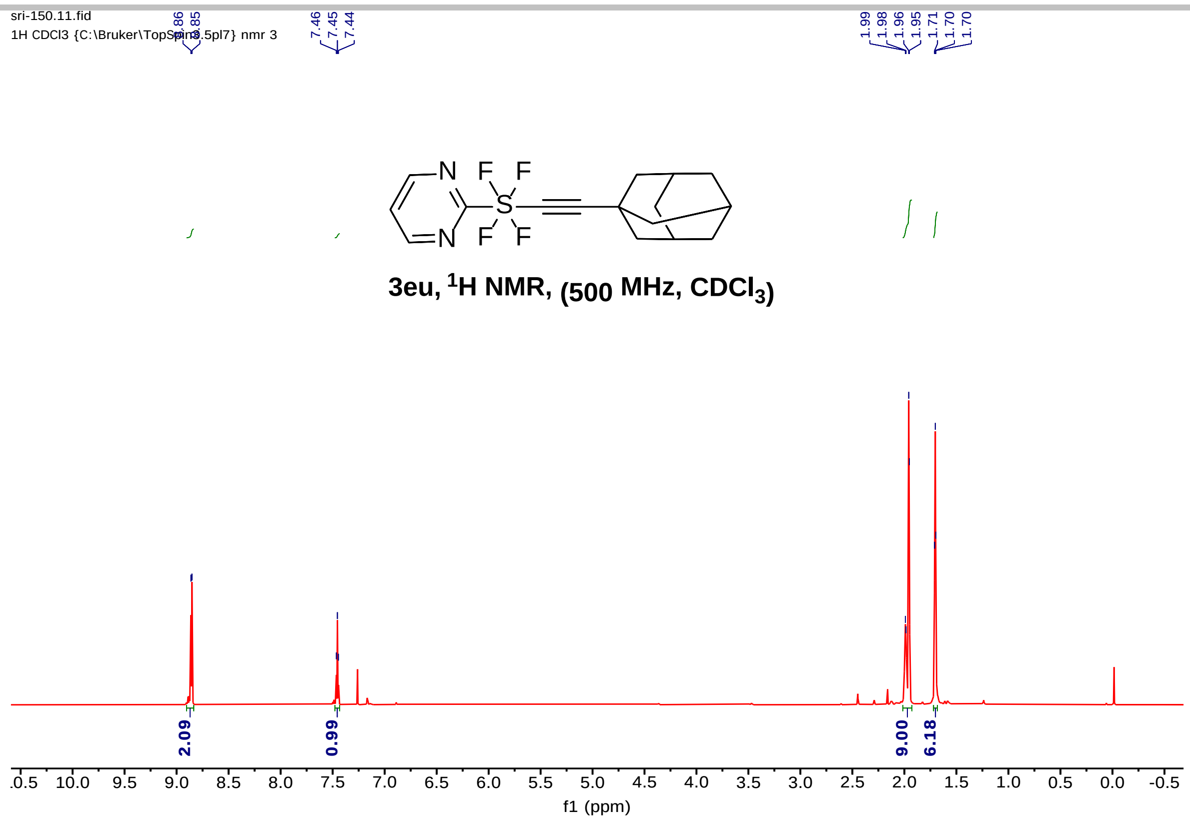
sri-150.11.fid
1H CDCl3 {C:\Bruker\TopSpin3.5pl7} nmr 3
3eu, 1H NMR, (500 MHz, CDCl3)
f1 (ppm)

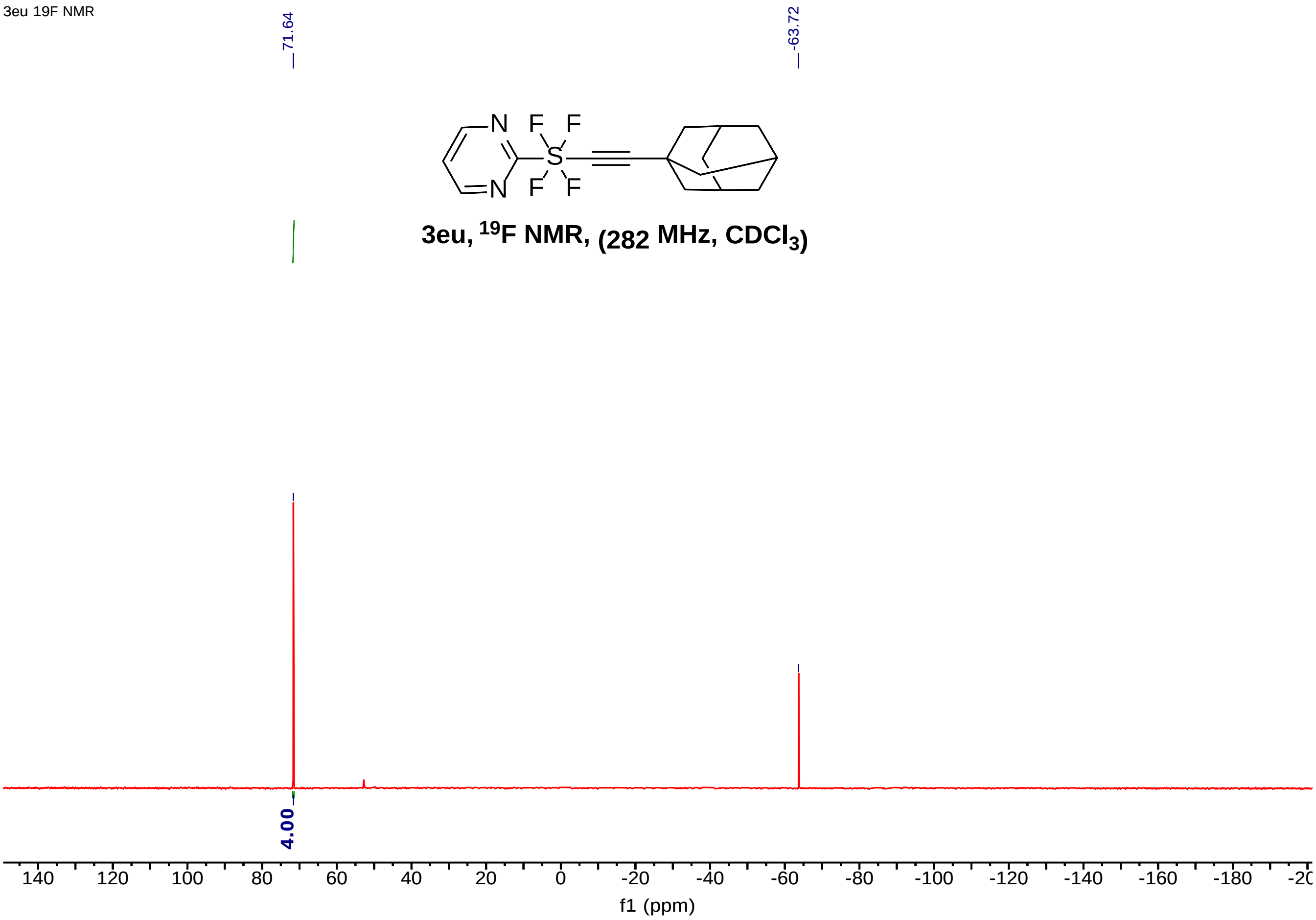
3eu 19F NMR
3eu, 19F NMR, (282 MHz, CDCl3)
f1 (ppm)

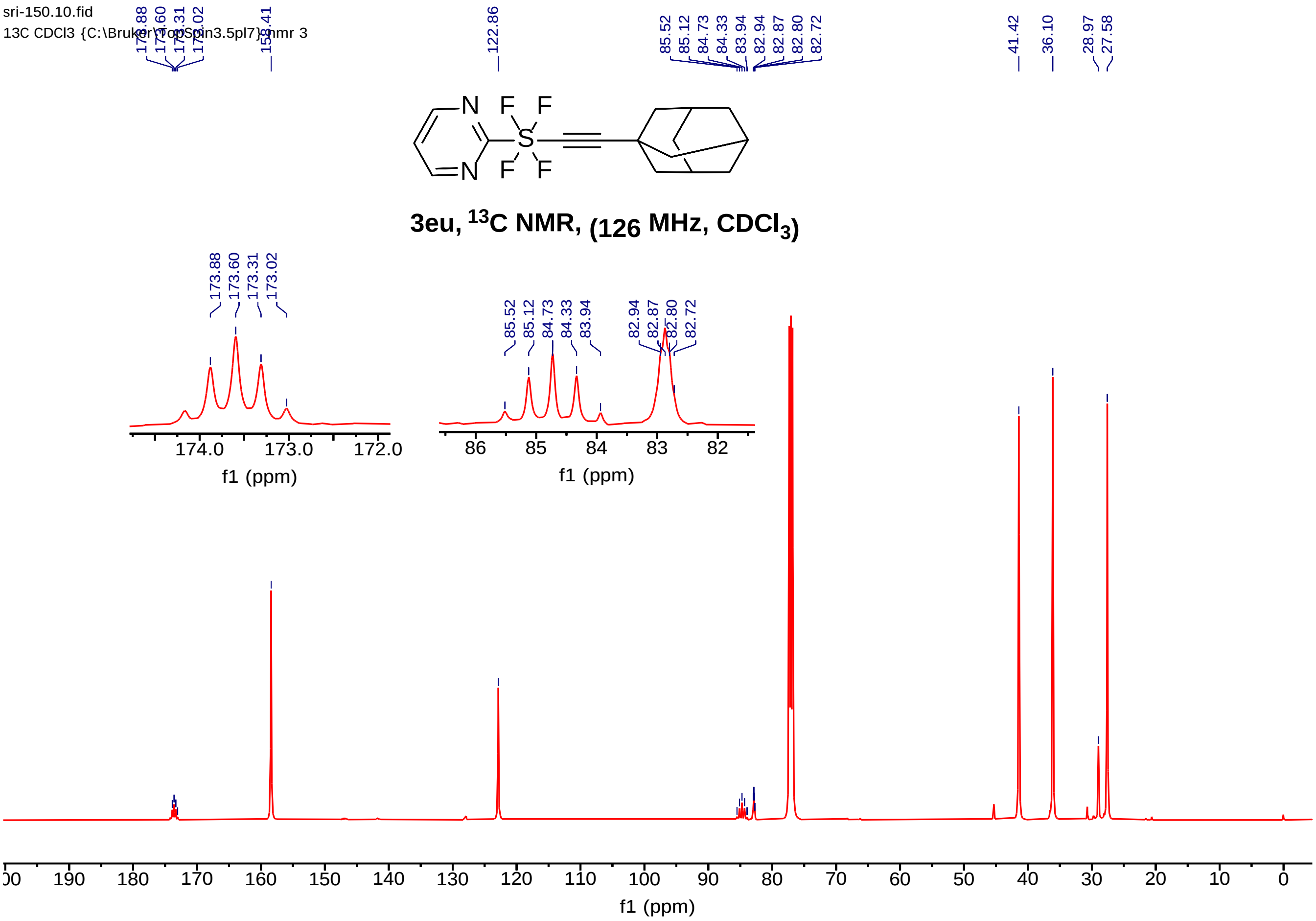

3eu, ^{13}C NMR, (126 MHz, $CDCl_3$)

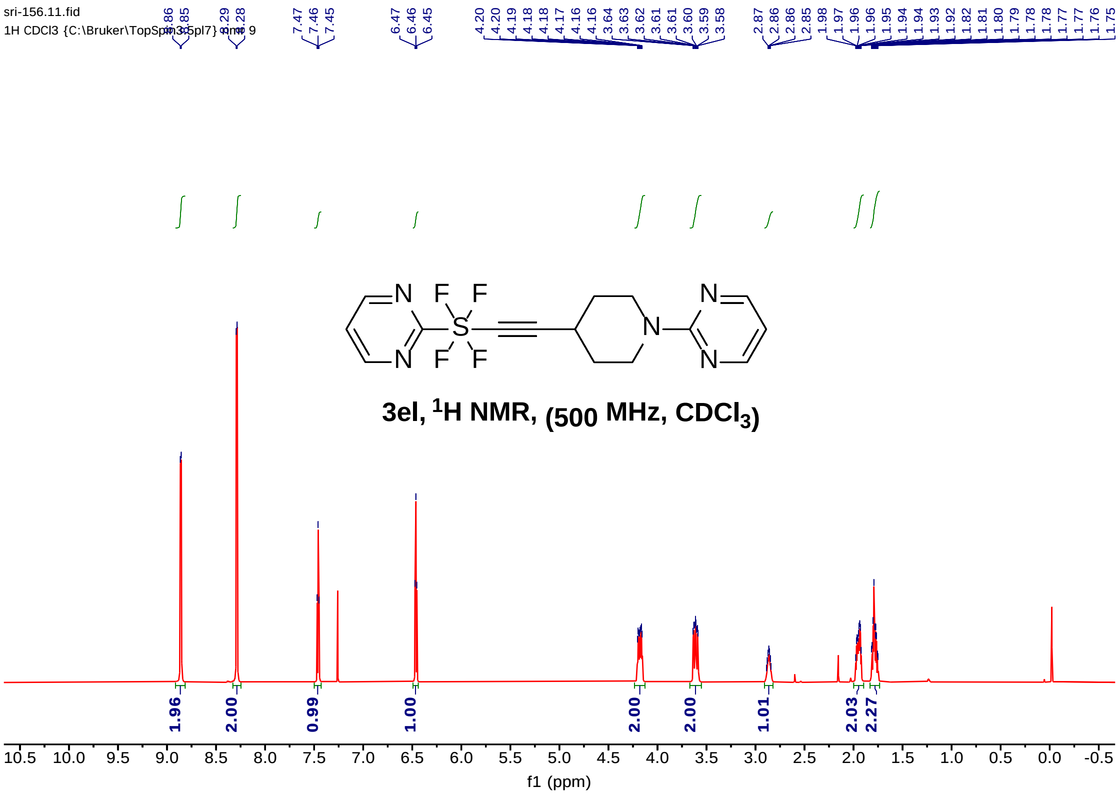

3el, ^{1}H NMR, (500 MHz, $CDCl_3$)

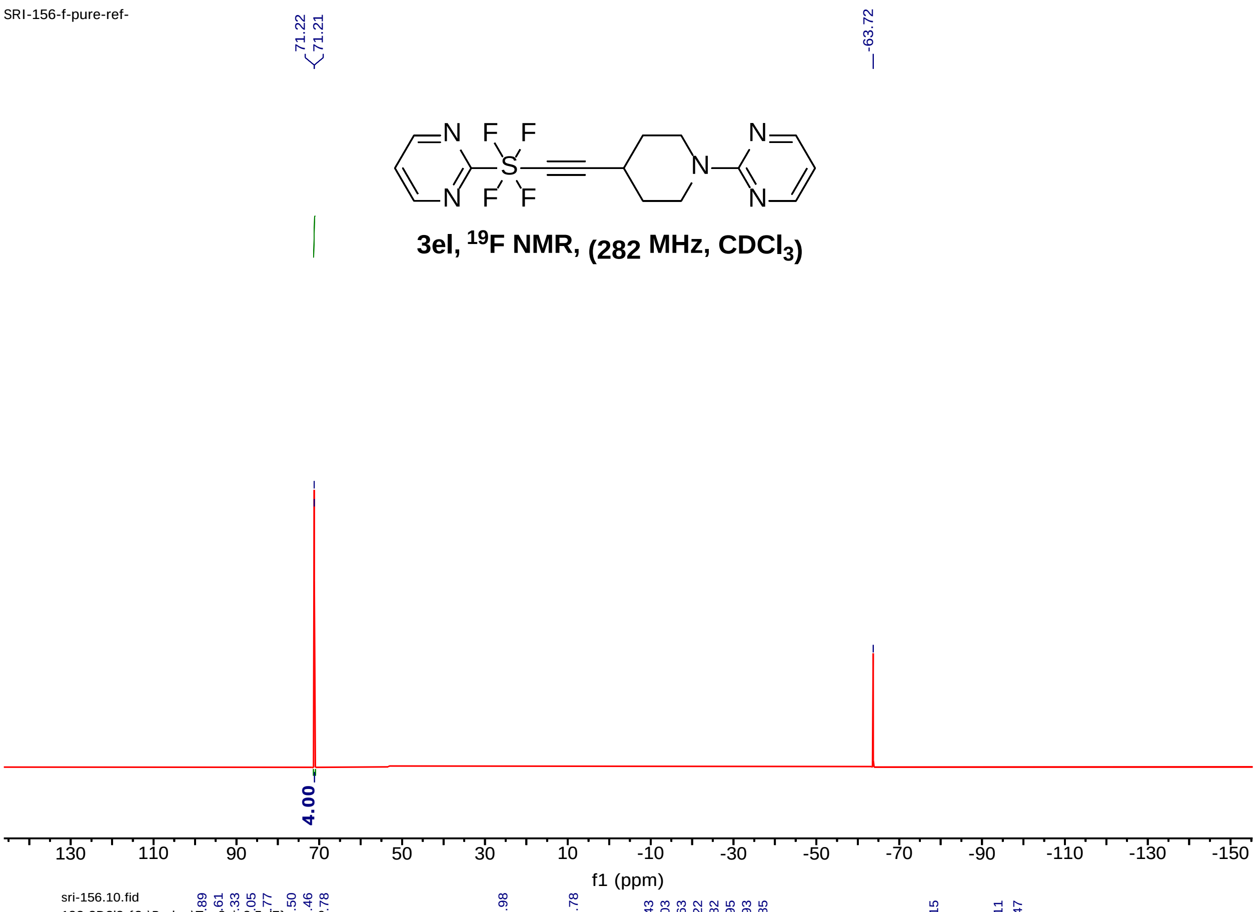

3el, ^{19}F NMR, (282 MHz, $CDCl_3$)

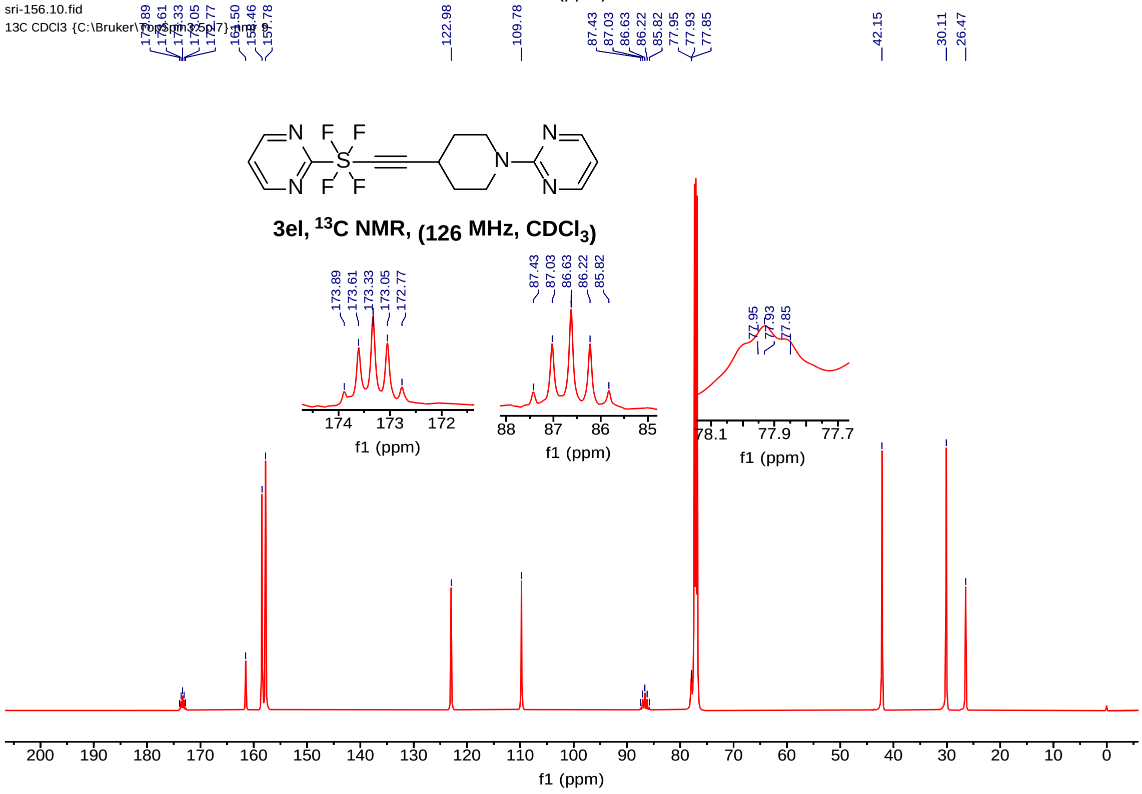

3el, ^{13}C NMR, (126 MHz, $CDCl_3$)

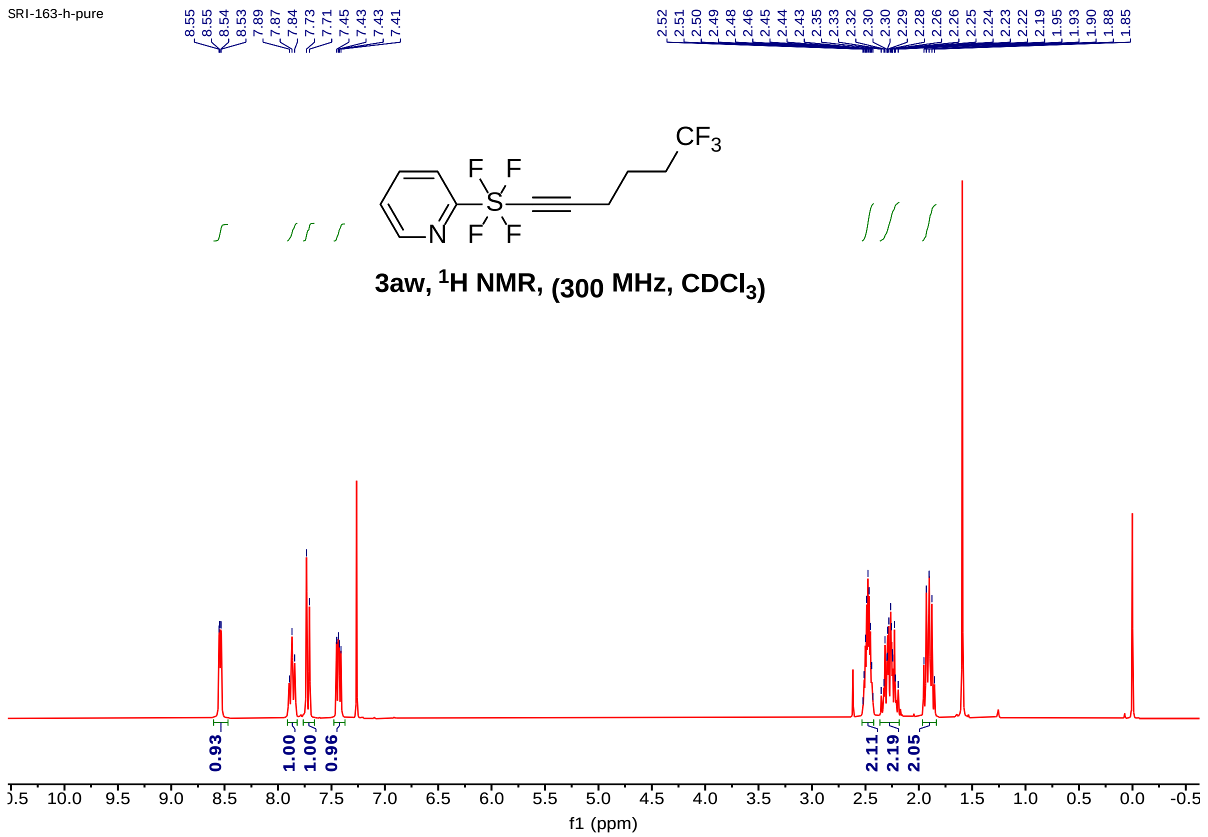
SRI-163-h-pure
CF3
F F
S
N F F
3aw, 1H NMR, (300 MHz, CDCl3)
0.93
1.00
1.00
0.96
2.11
2.19
2.05
f1 (ppm)

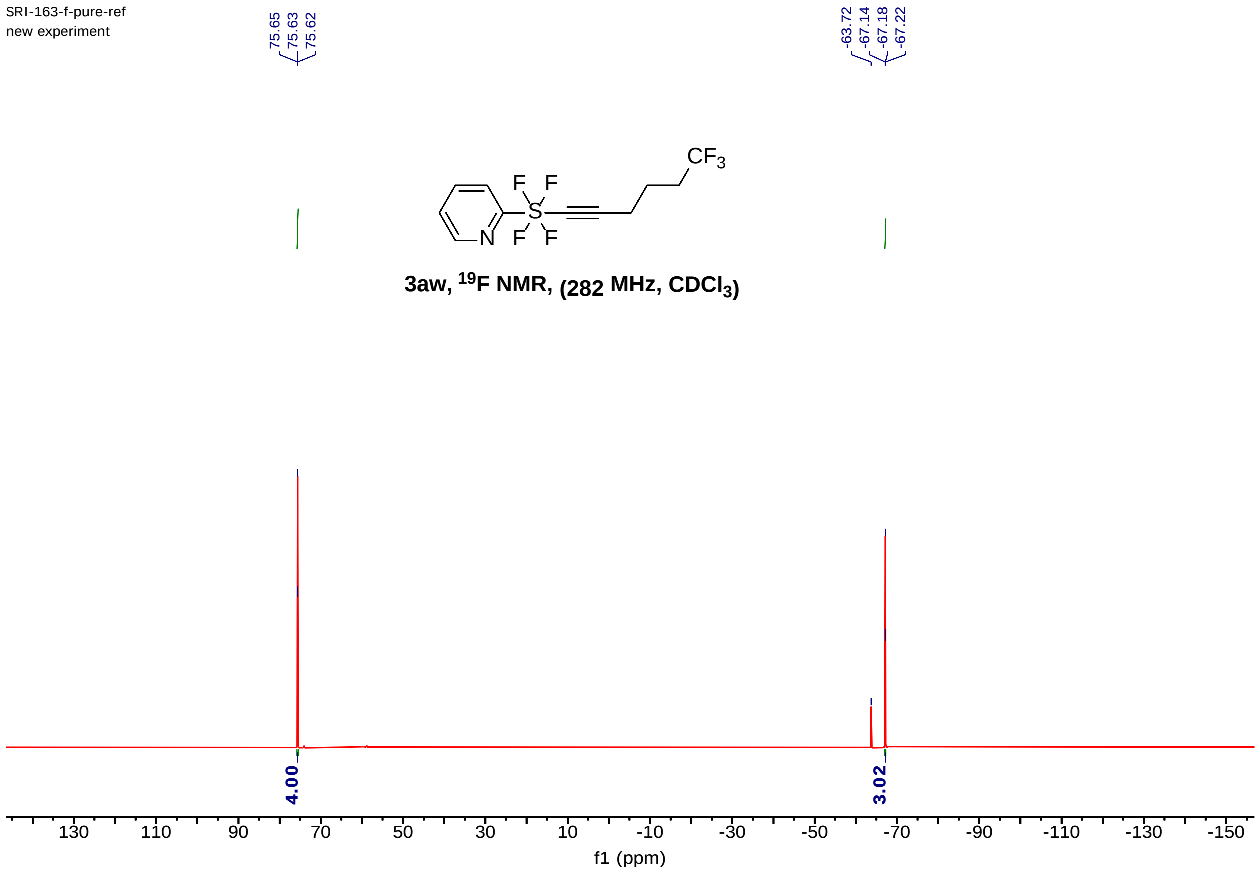
SRI-163-f-pure-ref
new experiment
75.65
75.63
75.62
-63.72
-67.14
-67.18
-67.22
CF3
F F
S
N F F
3aw, 19F NMR, (282 MHz, CDCl3)
4.00
3.02
f1 (ppm)

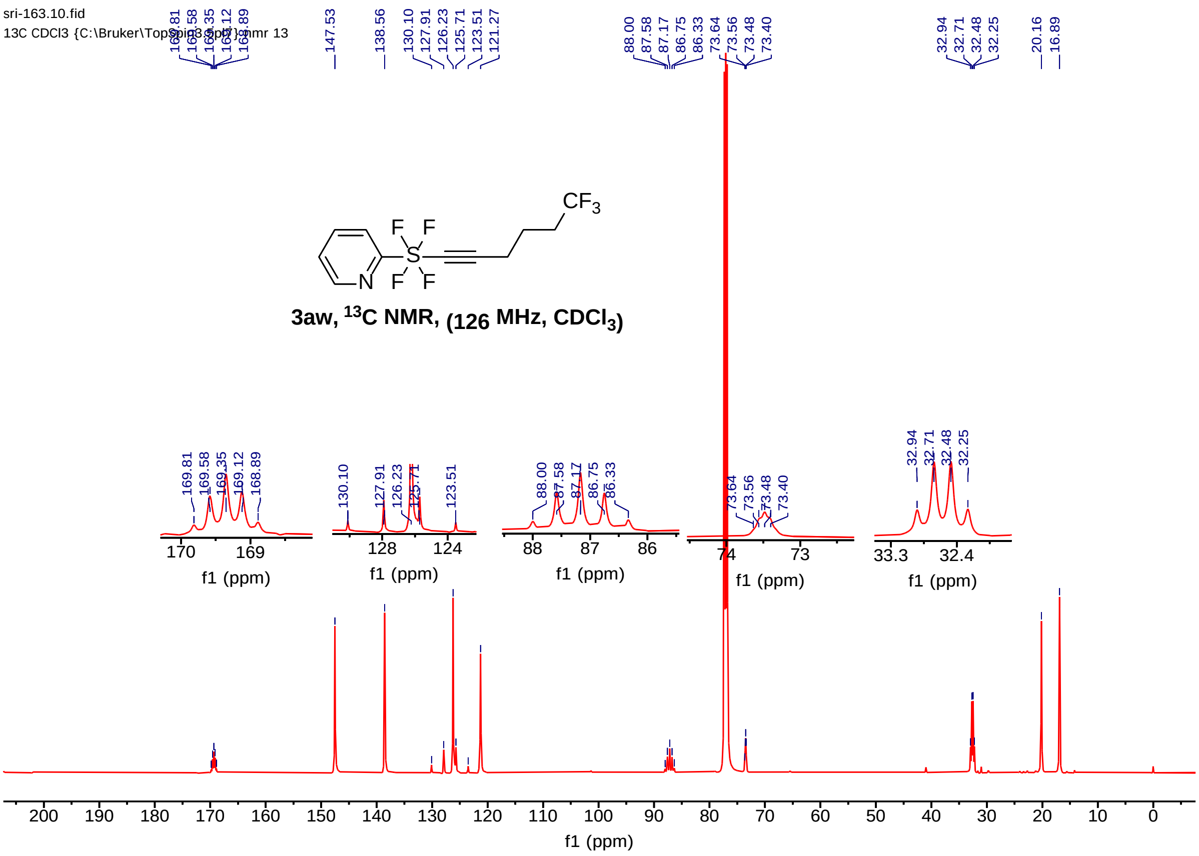
sri-163.10.fid
13C CDCl3 {C:\Bruker\TopSpin3.5pl7} nmr 13
3aw, ^{13}C NMR, (126 MHz, $CDCl_3$)
f1 (ppm)

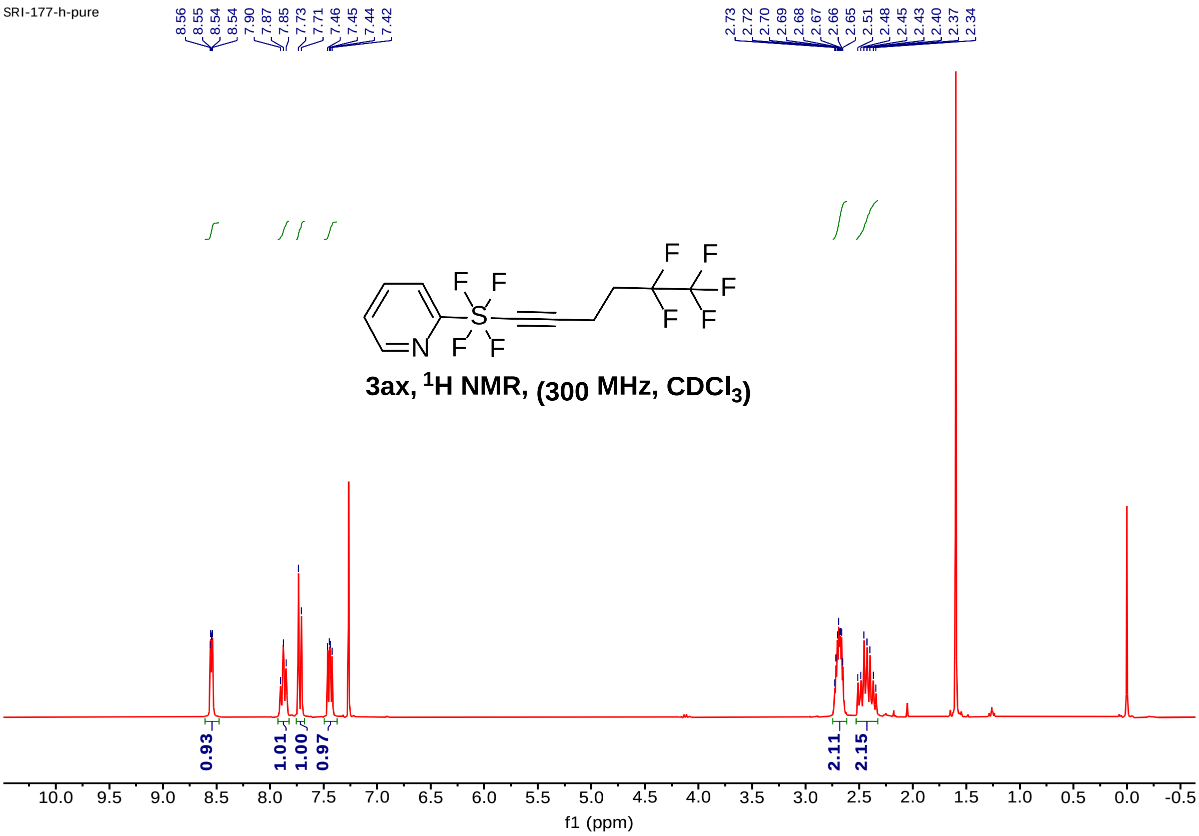
SRI-177-h-pure
3ax, ^{1}H NMR, (300 MHz, $CDCl_3$)
f1 (ppm)

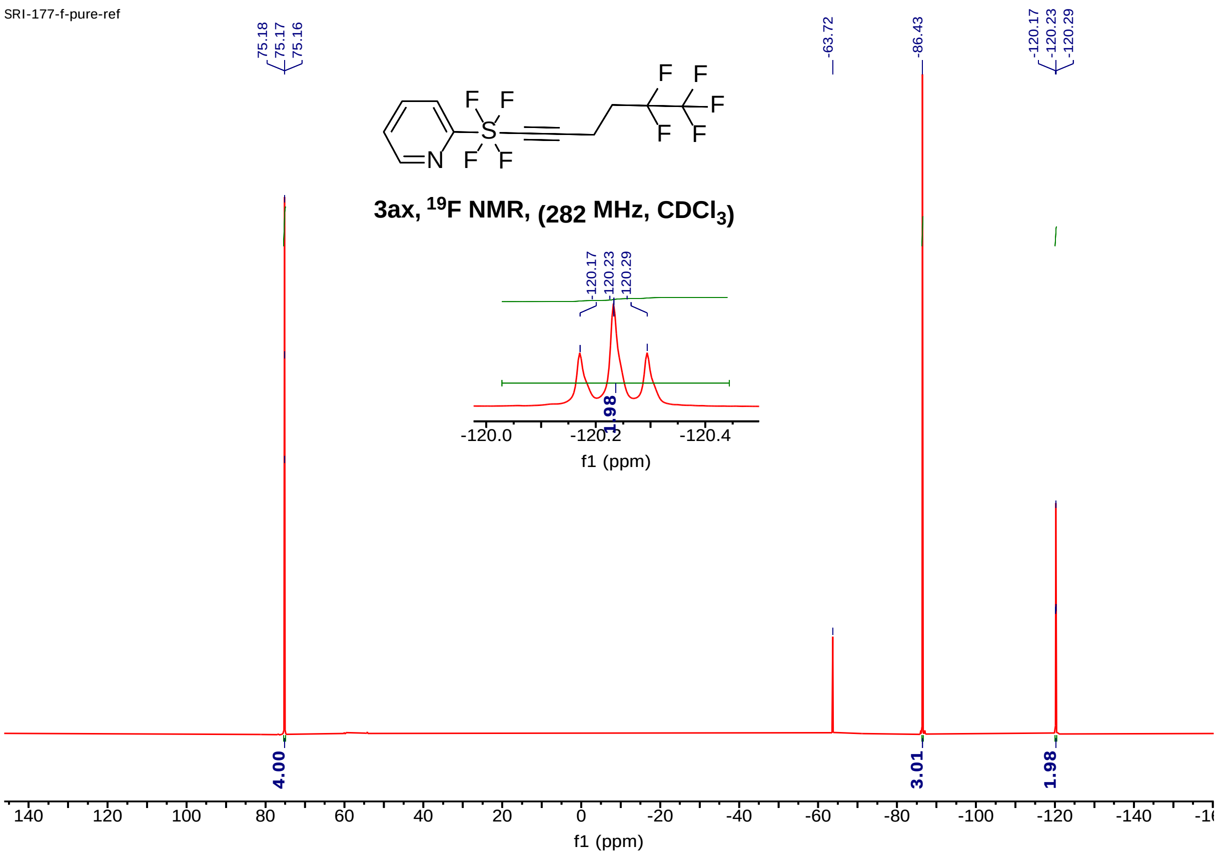
SRI-177-f-pure-ref
3ax, ^{19}F NMR, (282 MHz, CDCl$_3$)
75.18
75.17
75.16
-63.72
-86.43
-120.17
-120.23
-120.29
4.00
3.01
1.98
f1 (ppm)

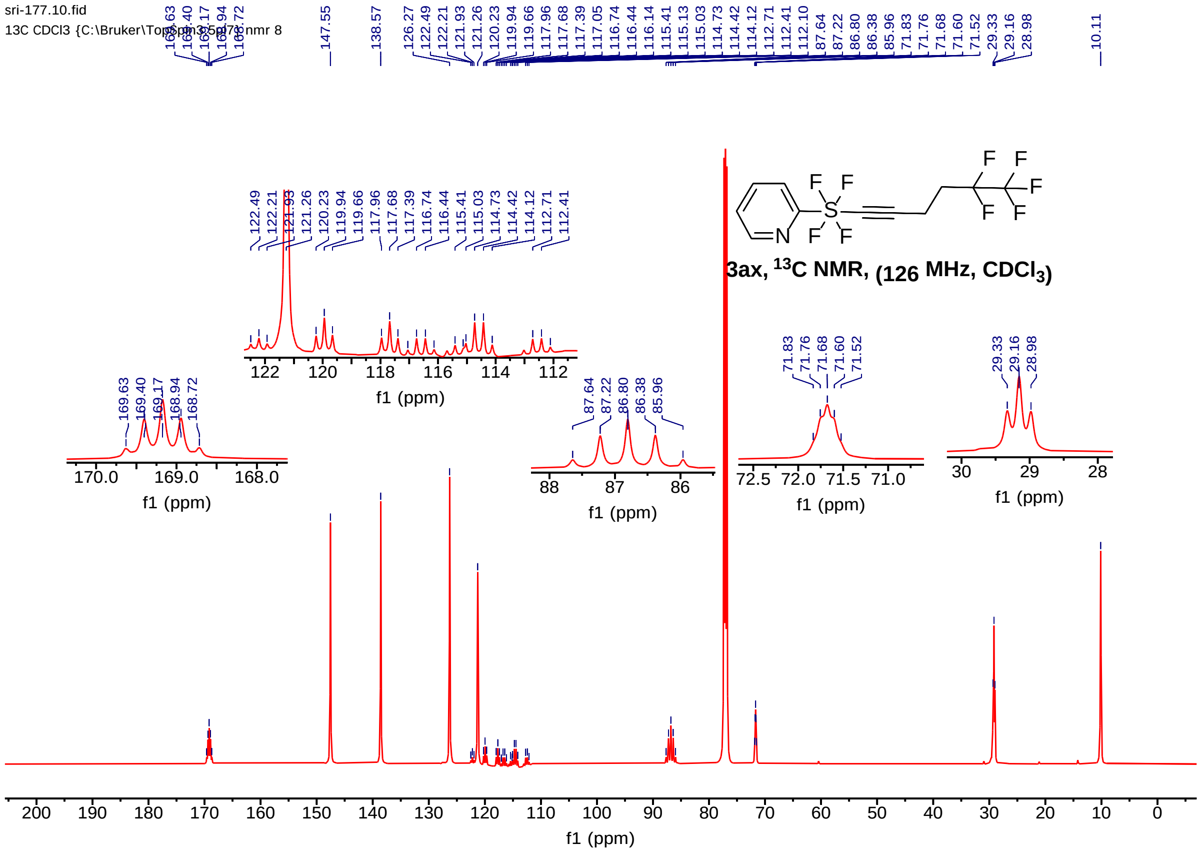
sri-177.10.fid
13C CDCl3 {C:\Bruker\TopSpin3.5pl7} nmr 8
3ax, ^{13}C NMR, (126 MHz, CDCl$_3$)
147.55
138.57
10.11
f1 (ppm)

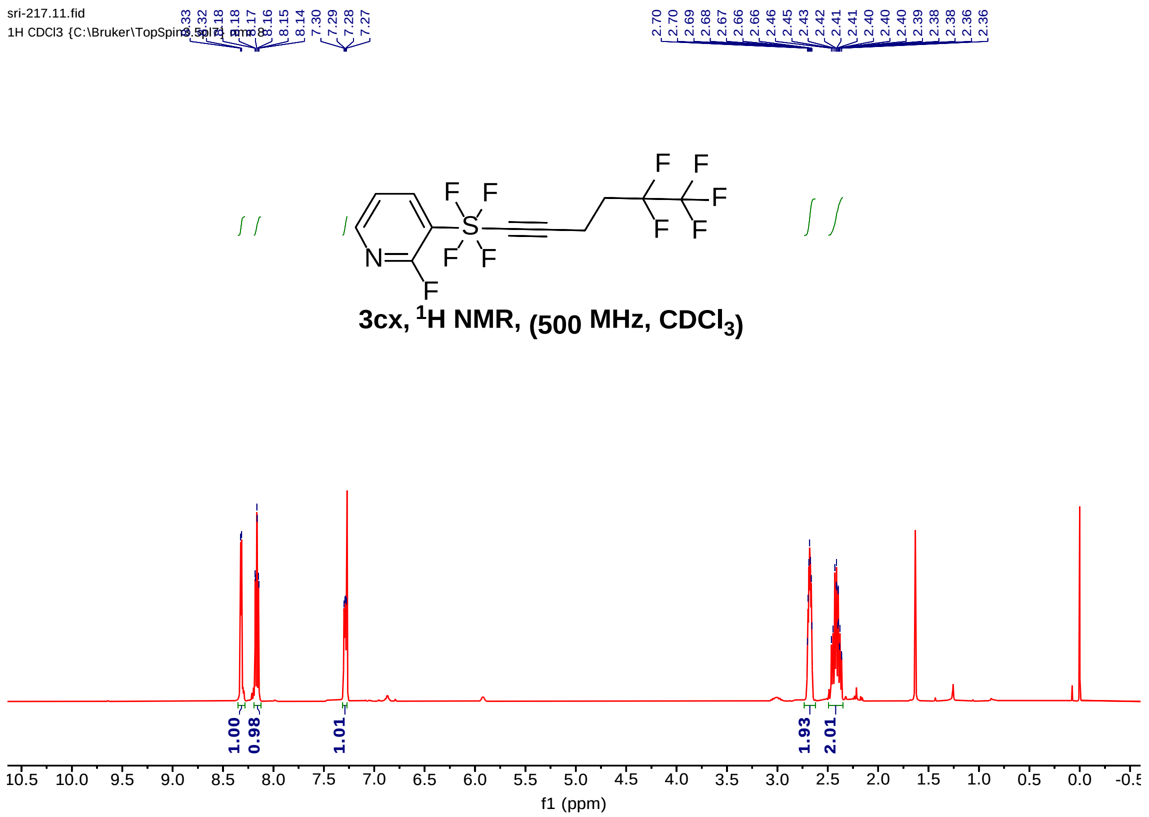
sri-217.11.fid
1H CDCl3 {C:\Bruker\TopSpin3.5pl7} nmr 8
3cx, 1H NMR, (500 MHz, CDCl3)
f1 (ppm)

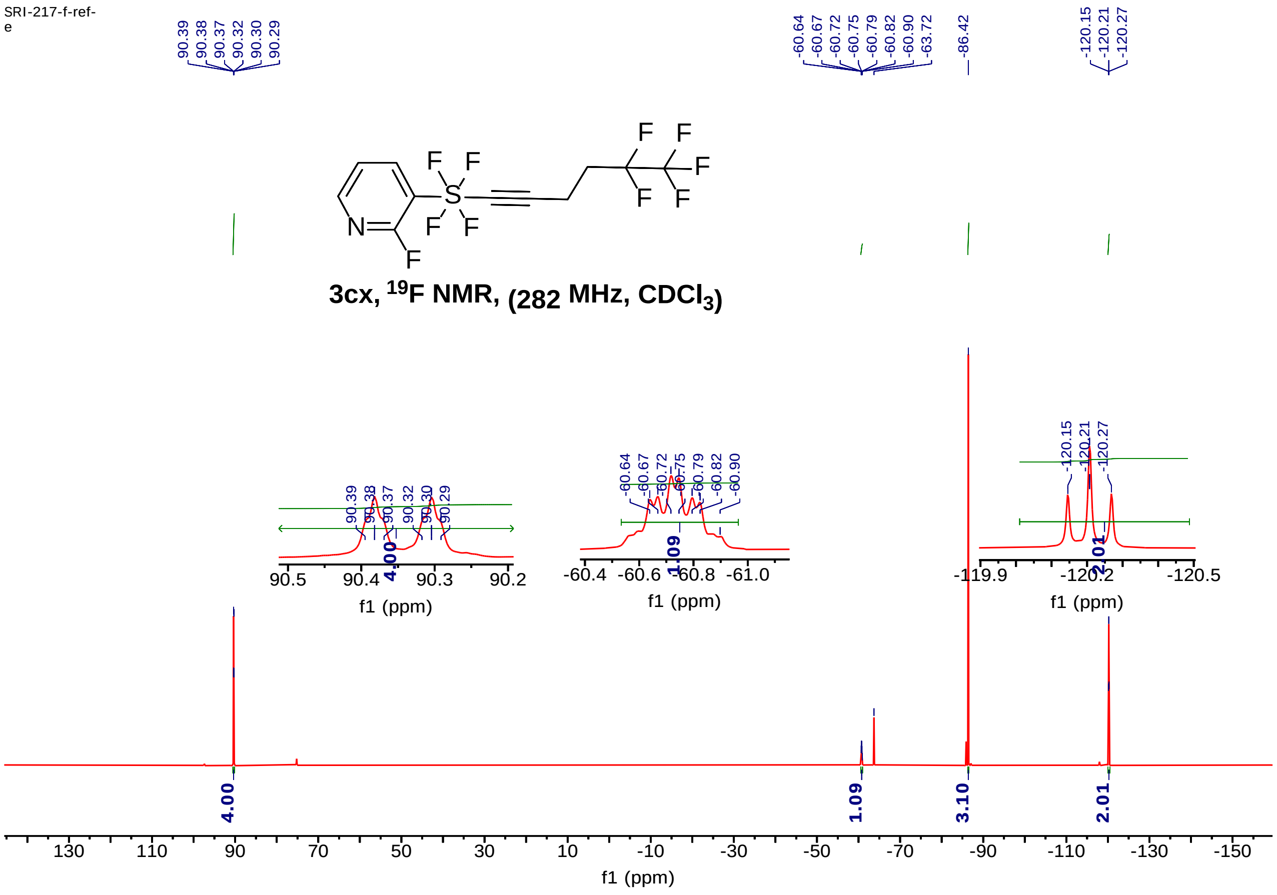
SRI-217-f-ref-e
3cx, 19F NMR, (282 MHz, CDCl3)
f1 (ppm)

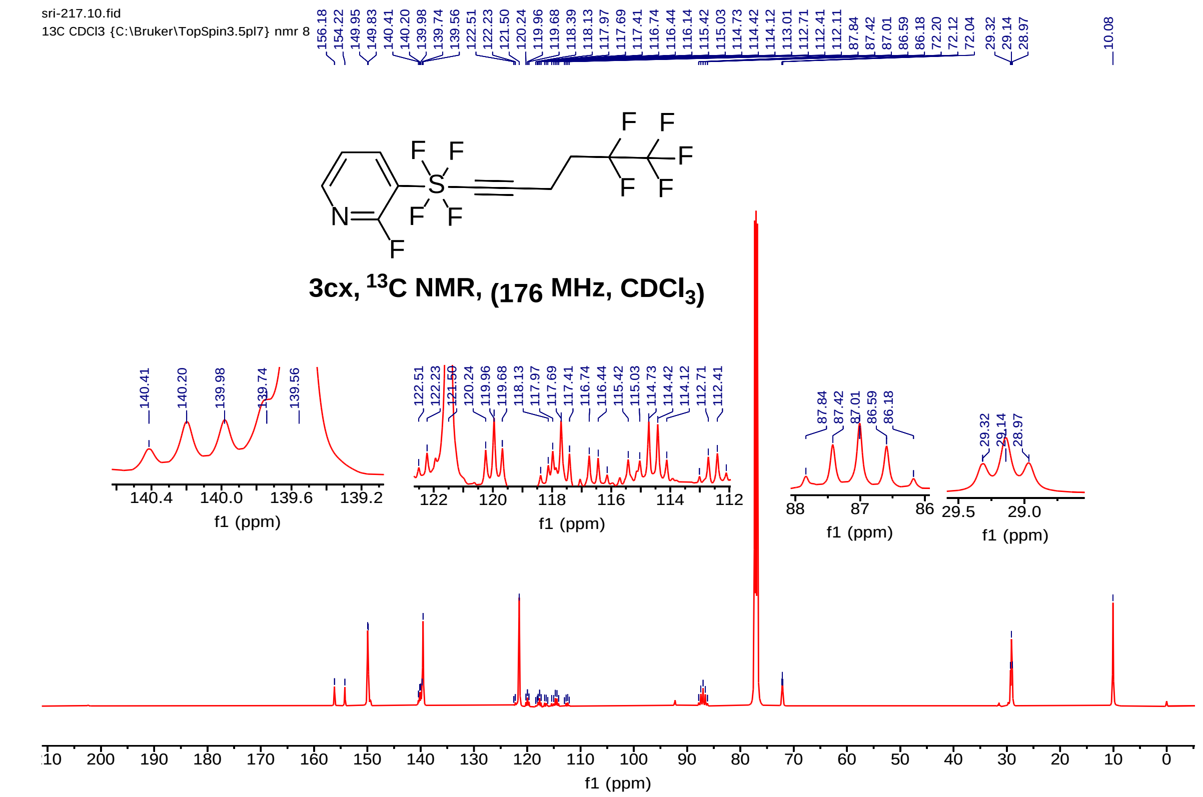
sri-217.10.fid
13C CDCl3 {C:\Bruker\TopSpin3.5pl7} nmr 8
156.18 154.22 149.95 149.83 140.41 140.20 139.98 139.74 139.56 122.51 122.23 121.50 120.24 119.96 119.68 118.39 118.13 117.97 117.69 117.41 116.74 116.44 116.14 115.42 115.03 114.73 114.42 114.12 113.01 112.71 112.41 112.11 87.84 87.42 87.01 86.59 86.18 72.20 72.12 72.04 29.32 29.14 28.97 10.08
3cx, ^{13}C NMR, (176 MHz, $CDCl_3$)
f1 (ppm)

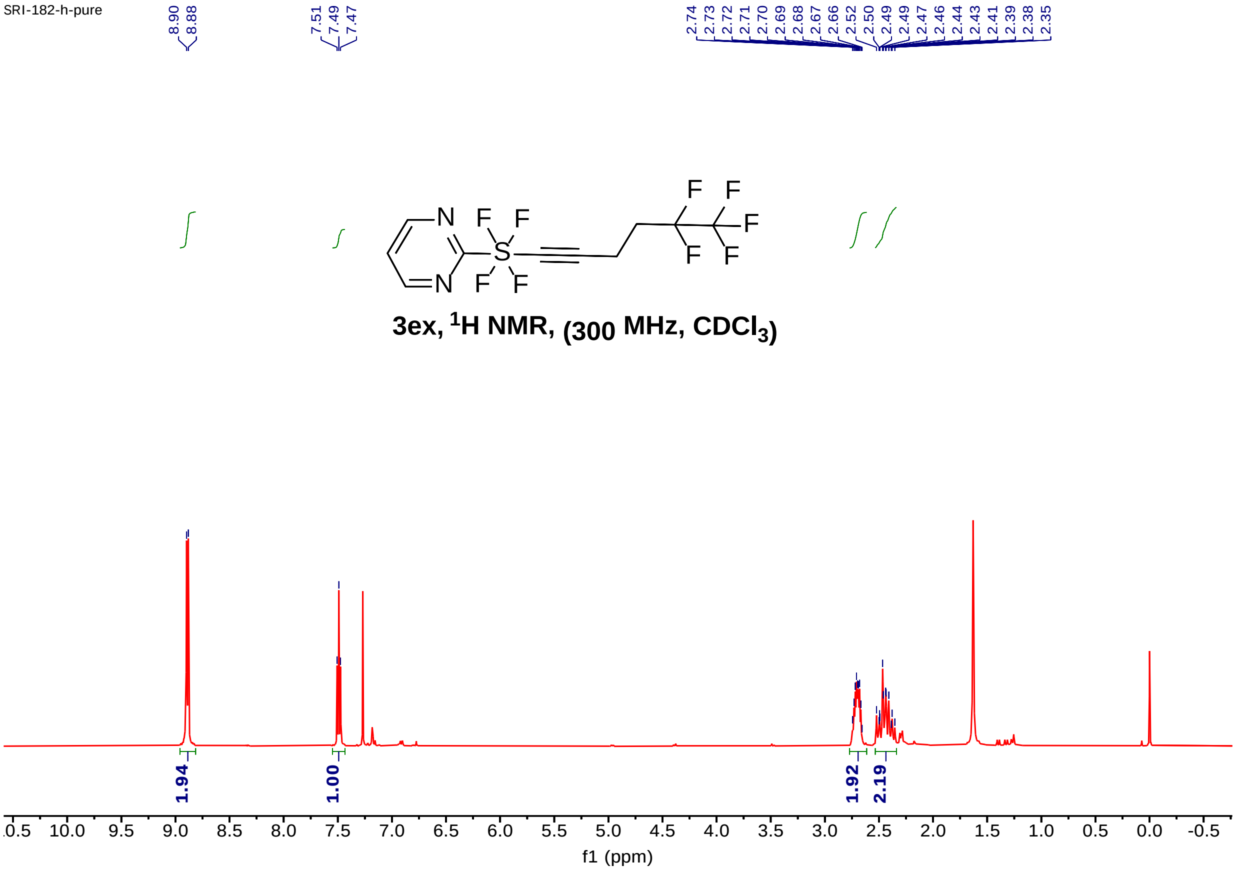
SRI-182-h-pure
8.90 8.88
7.51 7.49 7.47
2.74 2.73 2.72 2.71 2.70 2.69 2.68 2.67 2.66 2.52 2.50 2.49 2.49 2.47 2.46 2.44 2.43 2.41 2.39 2.38 2.35
3ex, ^{1}H NMR, (300 MHz, $CDCl_3$)
1.94
1.00
1.92
2.19
f1 (ppm)

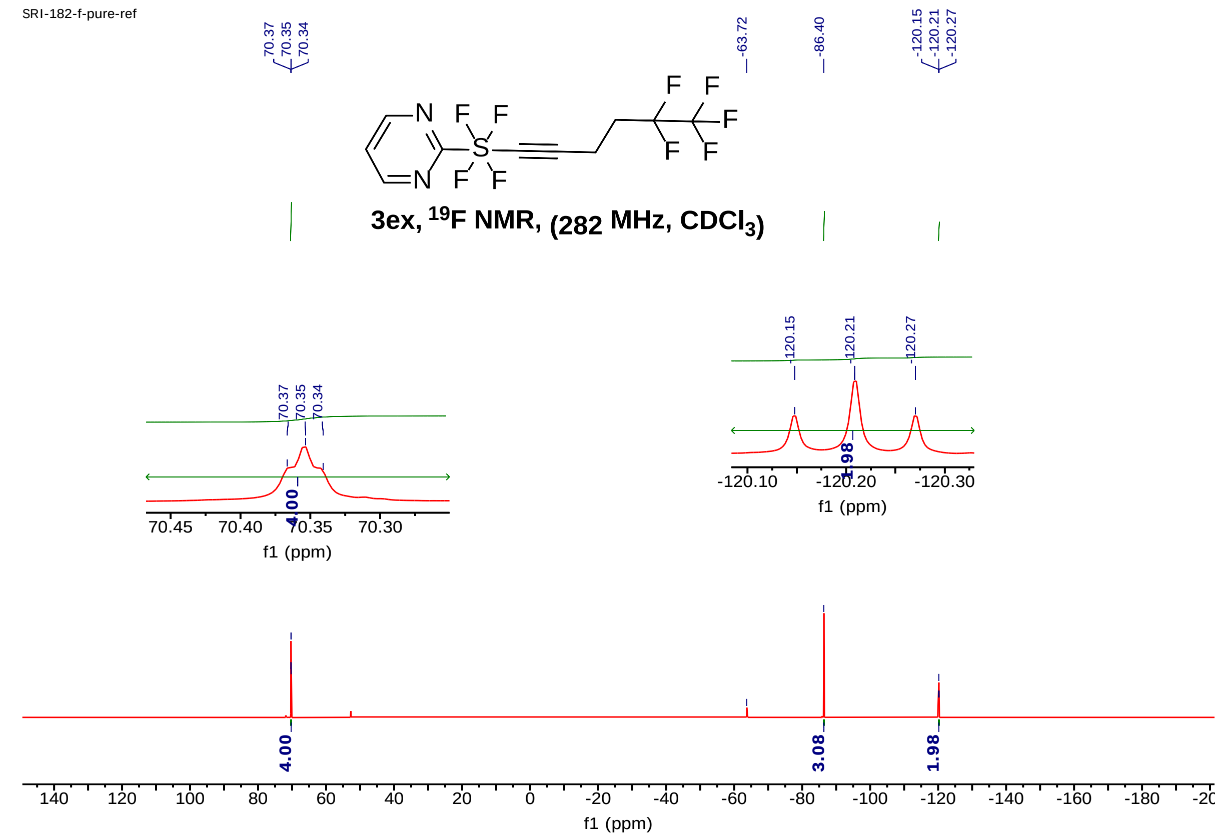
SRI-182-f-pure-ref
70.37
70.35
70.34
-63.72
-86.40
-120.15
-120.21
-120.27
3ex, 19F NMR, (282 MHz, CDCl3)
4.00
3.08
1.98
f1 (ppm)

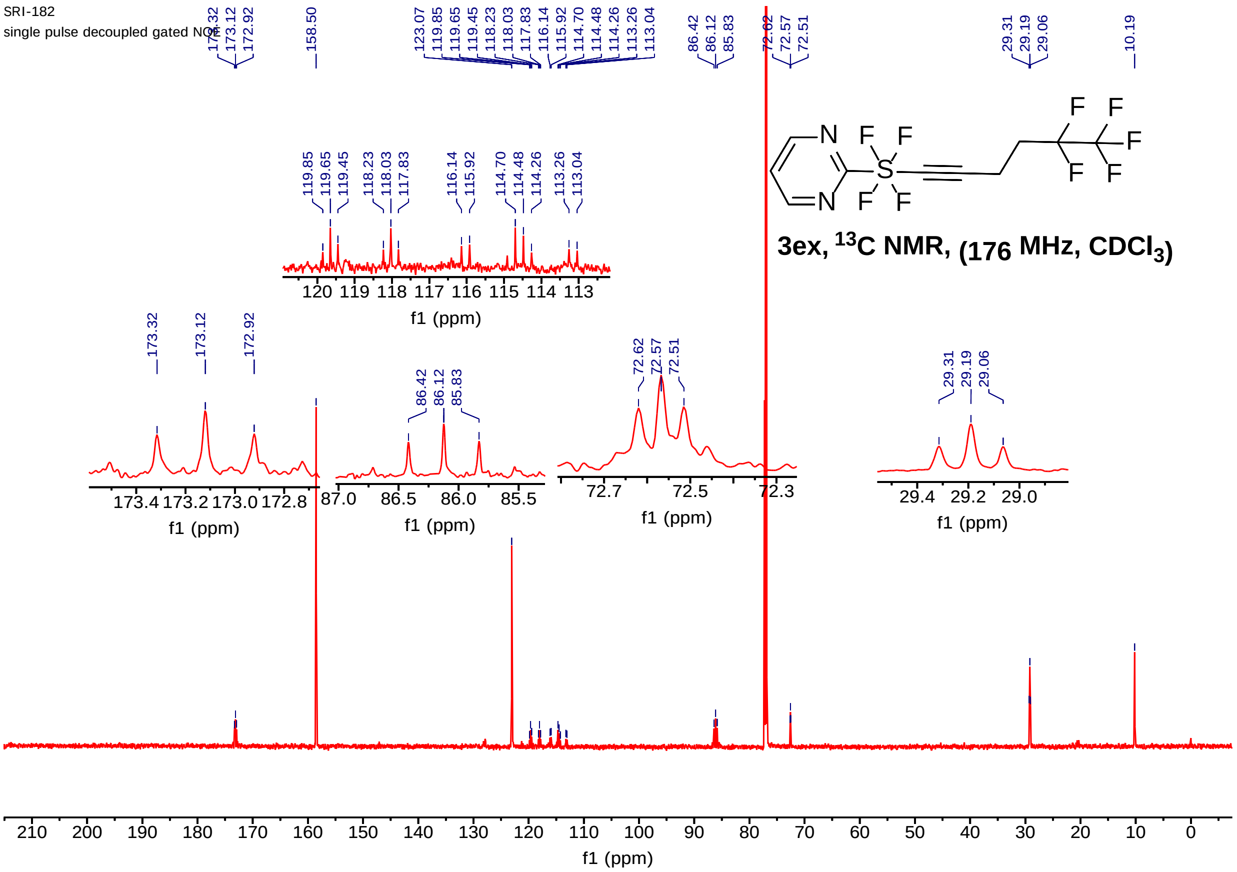
SRI-182
single pulse decoupled gated NOE
173.32
173.12
172.92
158.50
123.07
119.85
119.65
119.45
118.23
118.03
117.83
116.14
115.92
114.70
114.48
114.26
113.26
113.04
86.42
86.12
85.83
72.62
72.57
72.51
29.31
29.19
29.06
10.19
3ex, 13C NMR, (176 MHz, CDCl3)
f1 (ppm)

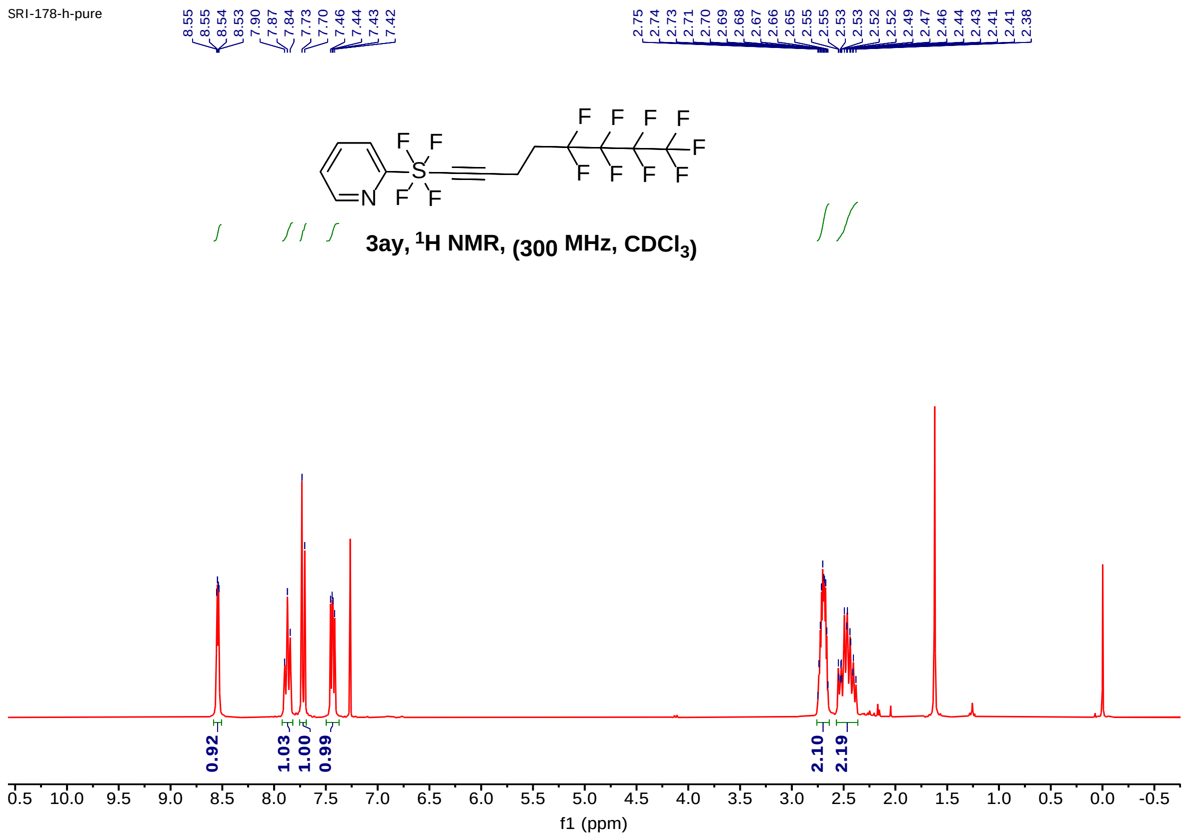
SRI-178-h-pure
3ay, ^{1}H NMR, (300 MHz, $CDCl_3$)
f1 (ppm)

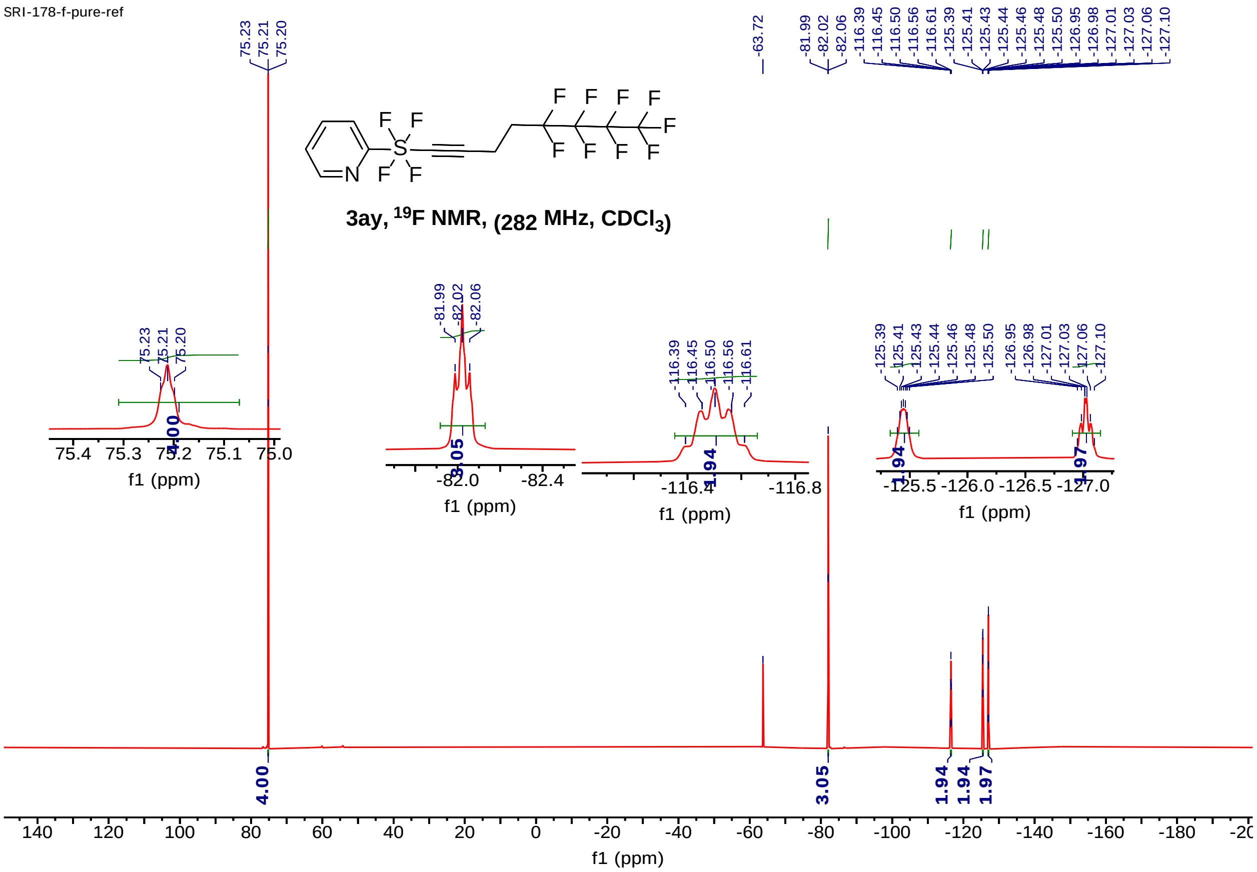
SRI-178-f-pure-ref
3ay, ^{19}F NMR, (282 MHz, $CDCl_3$)
f1 (ppm)

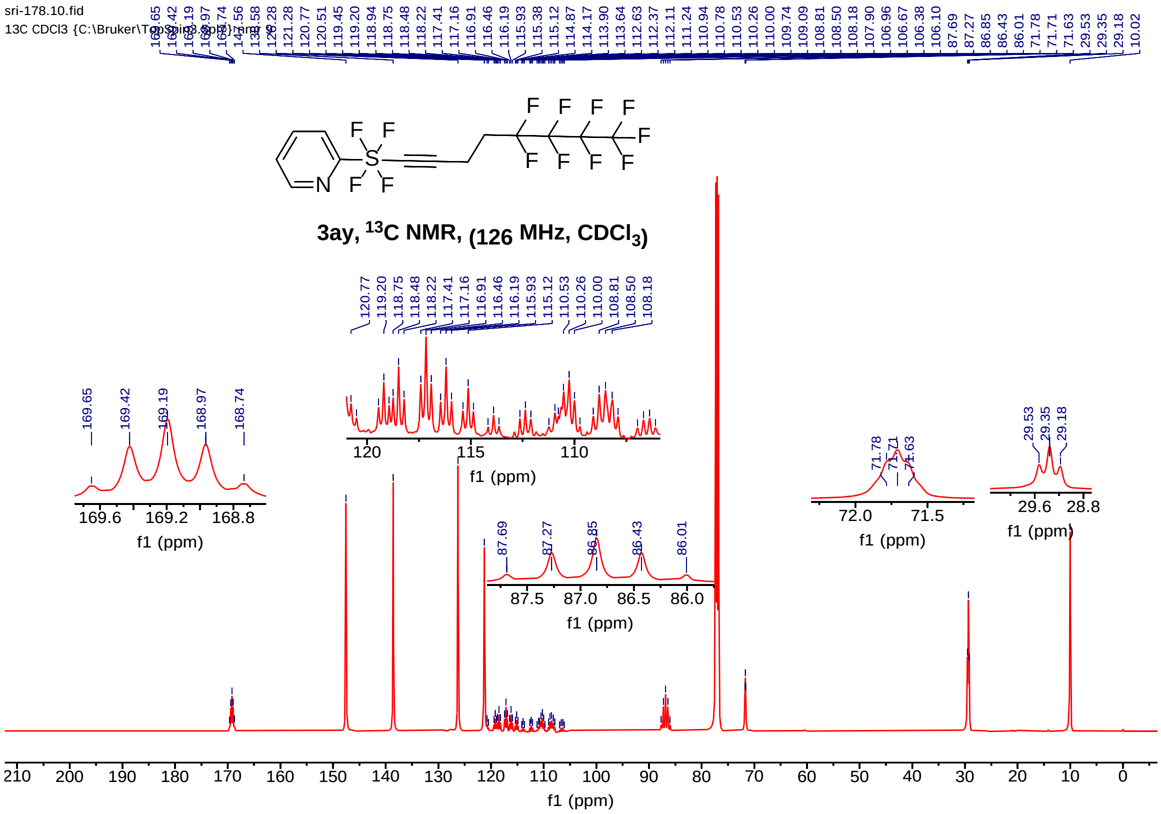
sri-178.10.fid
13C CDCl3 {C:\Bruker\TopSpin3.5pl7} nmr 10
3ay, ^{13}C NMR, (126 MHz, CDCl$_3$)
f1 (ppm)

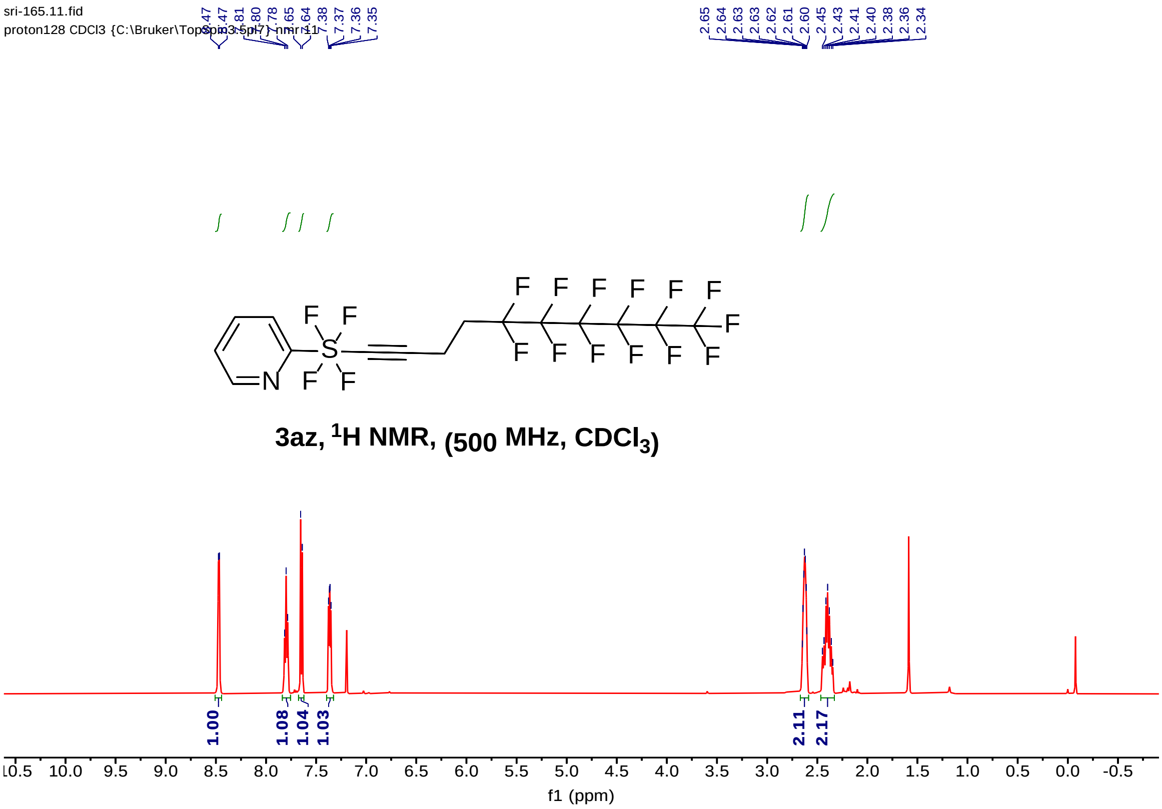
sri-165.11.fid
proton128 CDCl3 {C:\Bruker\TopSpin3.5pl7} nmr 11
3az, ^{1}H NMR, (500 MHz, CDCl$_3$)
1.00
1.08
1.04
1.03
2.11
2.17
f1 (ppm)

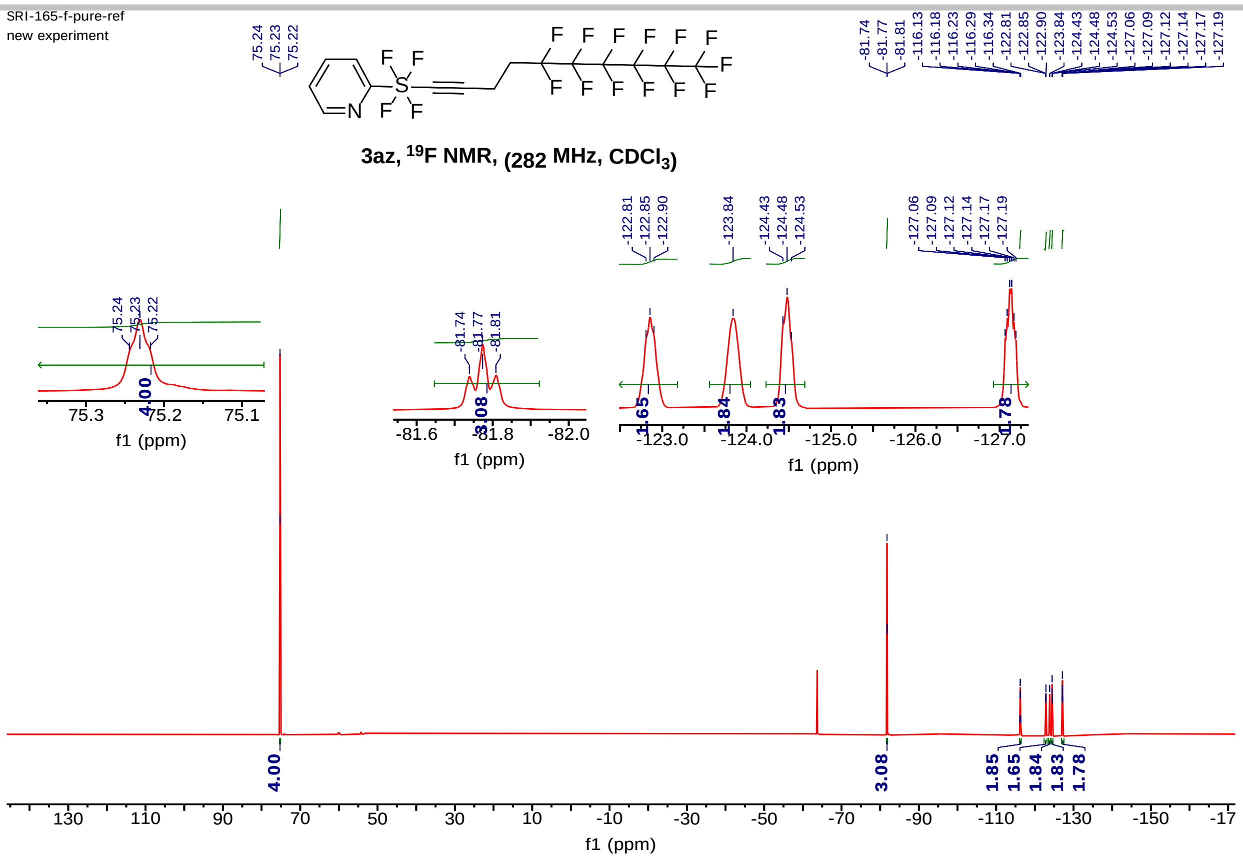
SRI-165-f-pure-ref
new experiment
3az, 19F NMR, (282 MHz, CDCl3)
f1 (ppm)

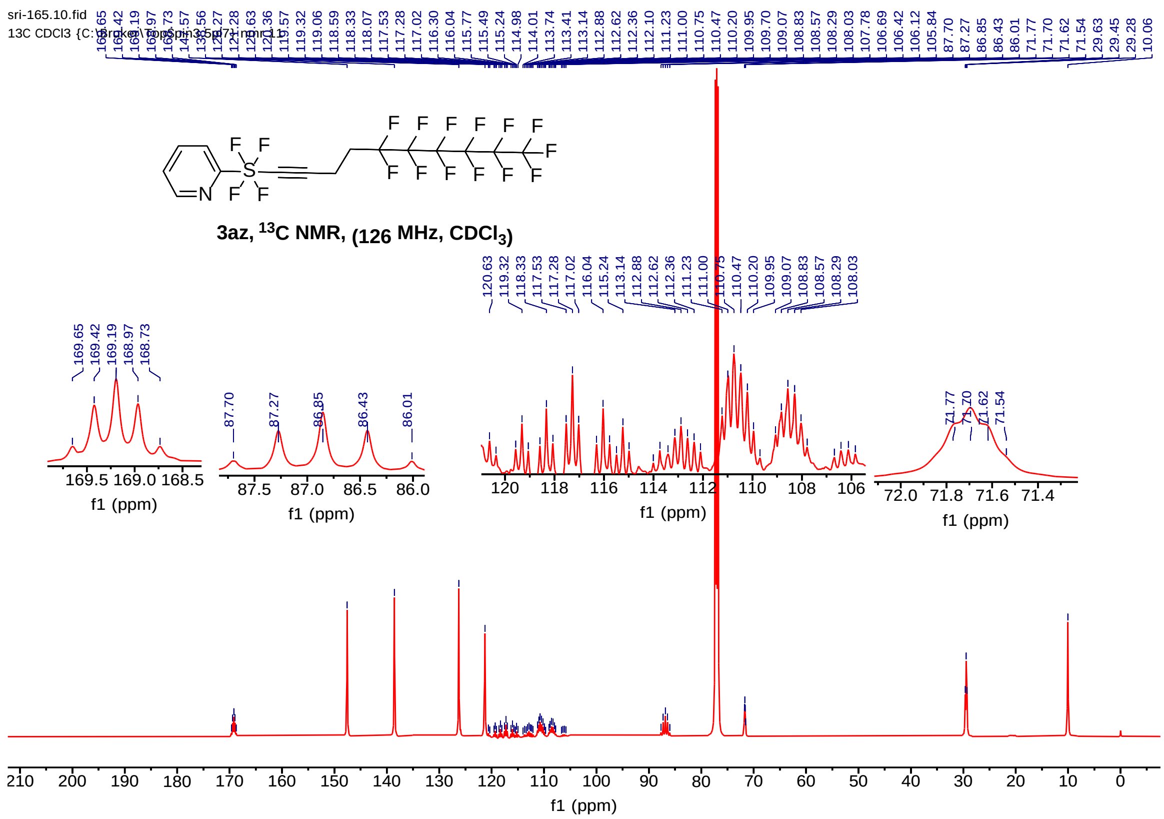
sri-165.10.fid
3az, 13C NMR, (126 MHz, CDCl3)
f1 (ppm)

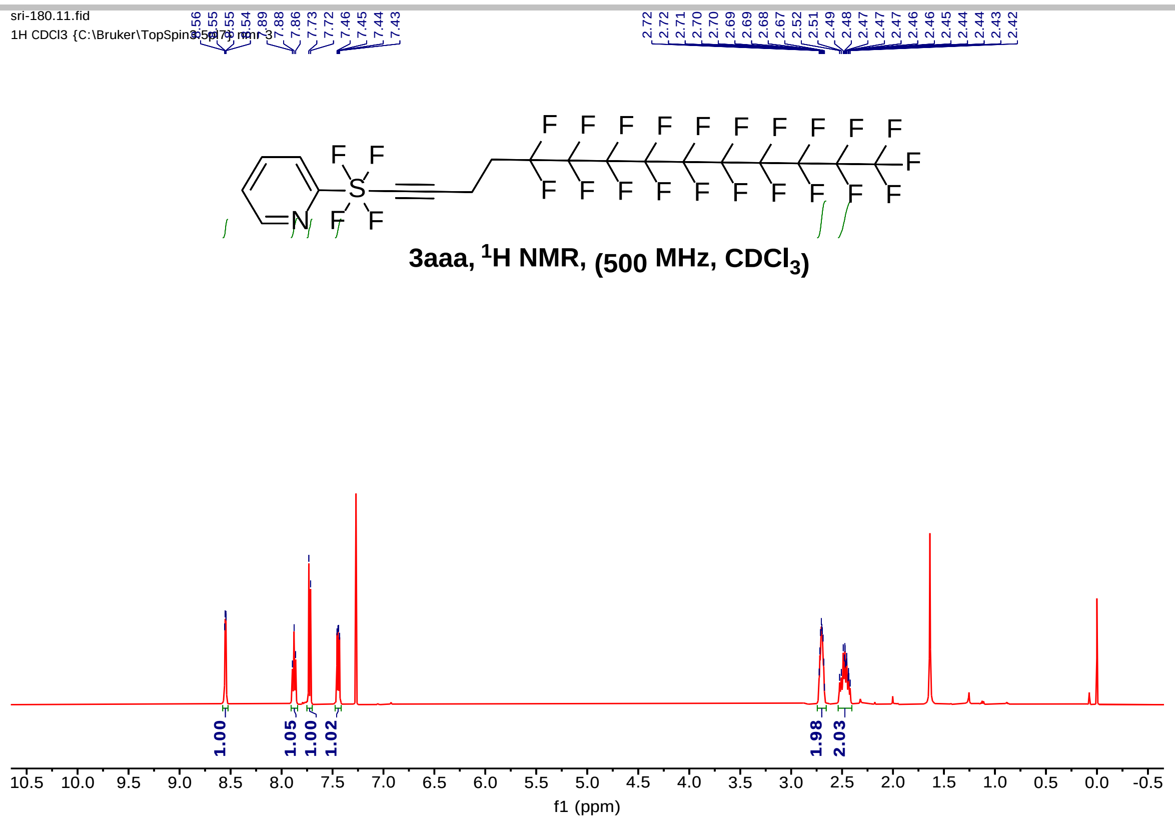

3aaa, ^{1}H NMR, (500 MHz, $CDCl_3$)

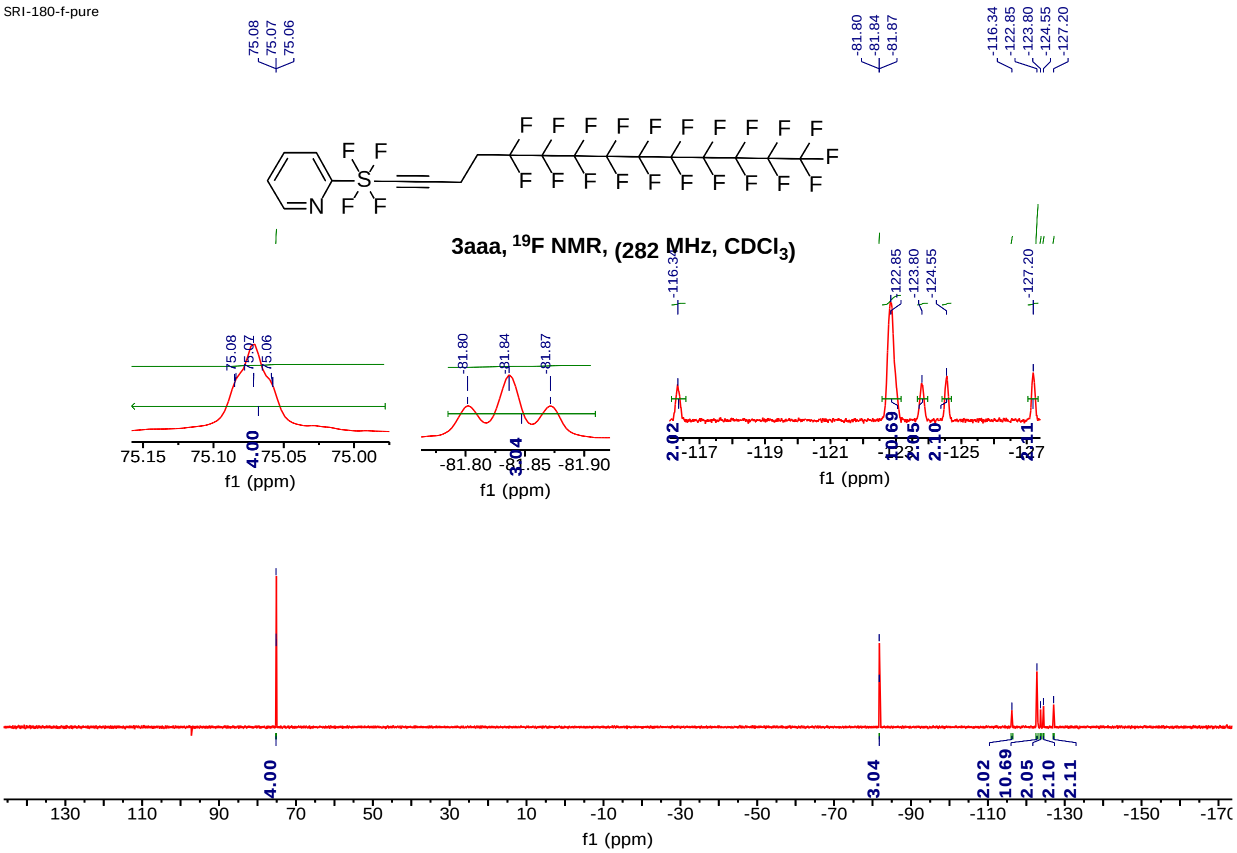

3aaa, ^{19}F NMR, (282 MHz, $CDCl_3$)

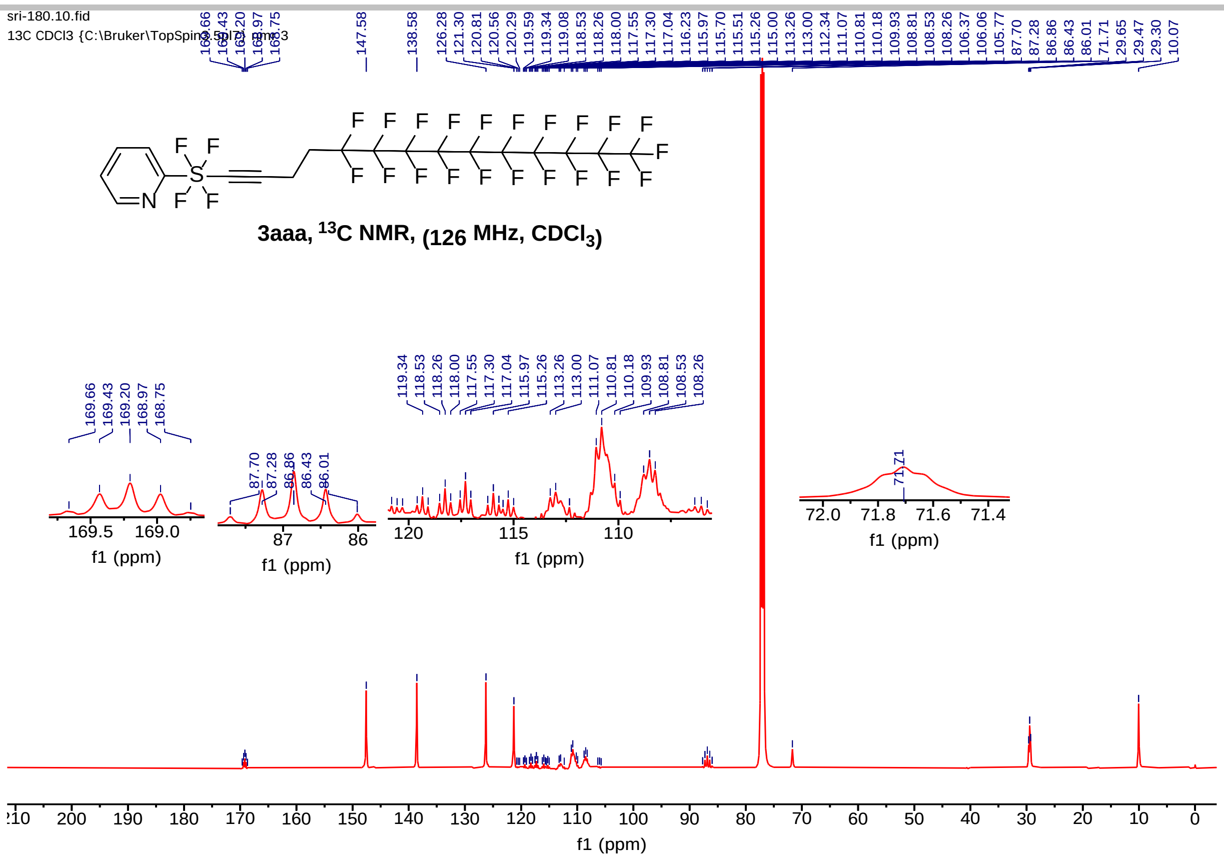

3aaa, ^{13}C NMR, (126 MHz, $CDCl_3$)

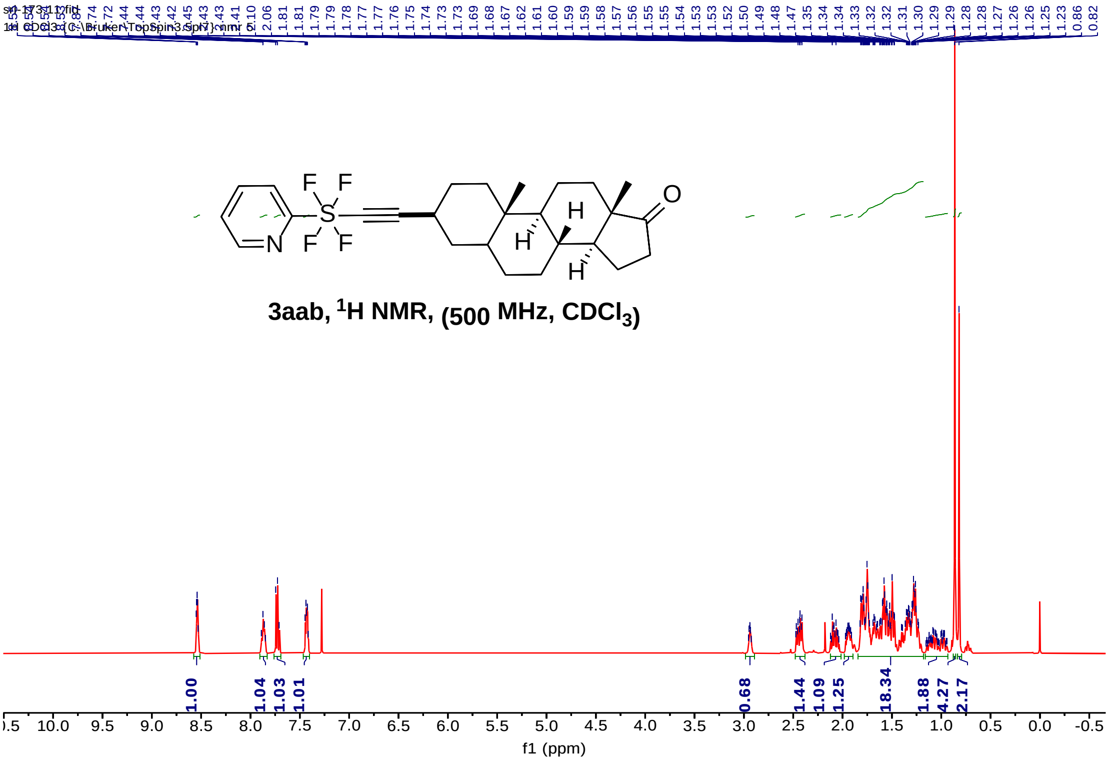

3aab, ^{1}H NMR, (500 MHz, $CDCl_3$)

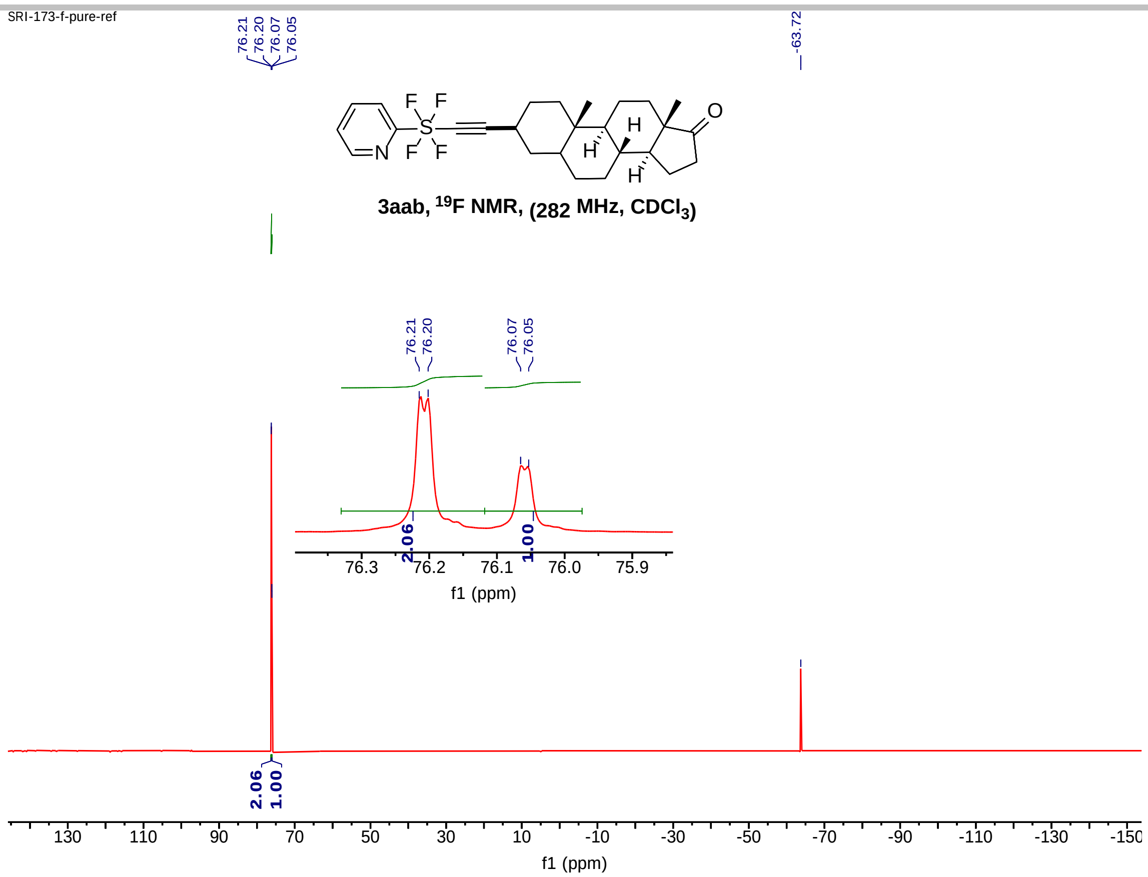
SRI-173-f-pure-ref
76.21
76.20
76.07
76.05
-63.72
3aab, 19F NMR, (282 MHz, CDCl3)
2.06
1.00
76.3
76.2
76.1
76.0
75.9
f1 (ppm)
130
110
90
70
50
30
10
-10
-30
-50
-70
-90
-110
-130
-150
f1 (ppm)

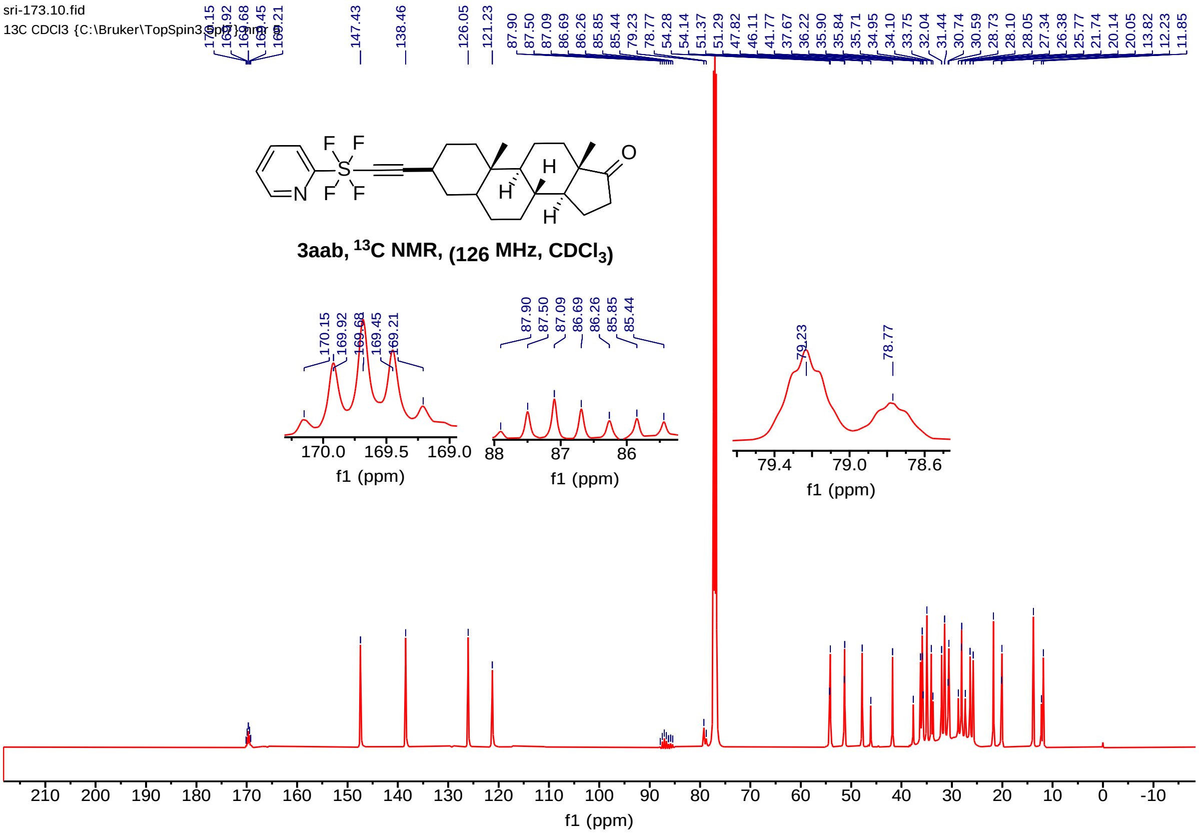
sri-173.10.fid
13C CDCl3 {C:\Bruker\TopSpin3.5pl7}
170.15
169.92
169.68
169.45
169.21
147.43
138.46
126.05
121.23
87.90
87.50
87.09
86.69
86.26
85.85
85.44
79.23
78.77
54.28
54.14
51.37
51.29
47.82
46.11
41.77
37.67
36.22
35.90
35.84
35.71
34.95
34.10
33.75
32.04
31.44
30.74
30.59
28.73
28.10
28.05
27.34
26.38
25.77
21.74
20.14
20.05
13.82
12.23
11.85
3aab, 13C NMR, (126 MHz, CDCl3)
170.0
169.5
169.0
f1 (ppm)
88
87
86
f1 (ppm)
79.4
79.0
78.6
f1 (ppm)
210
200
190
180
170
160
150
140
130
120
110
100
90
80
70
60
50
40
30
20
10
0
-10
f1 (ppm)

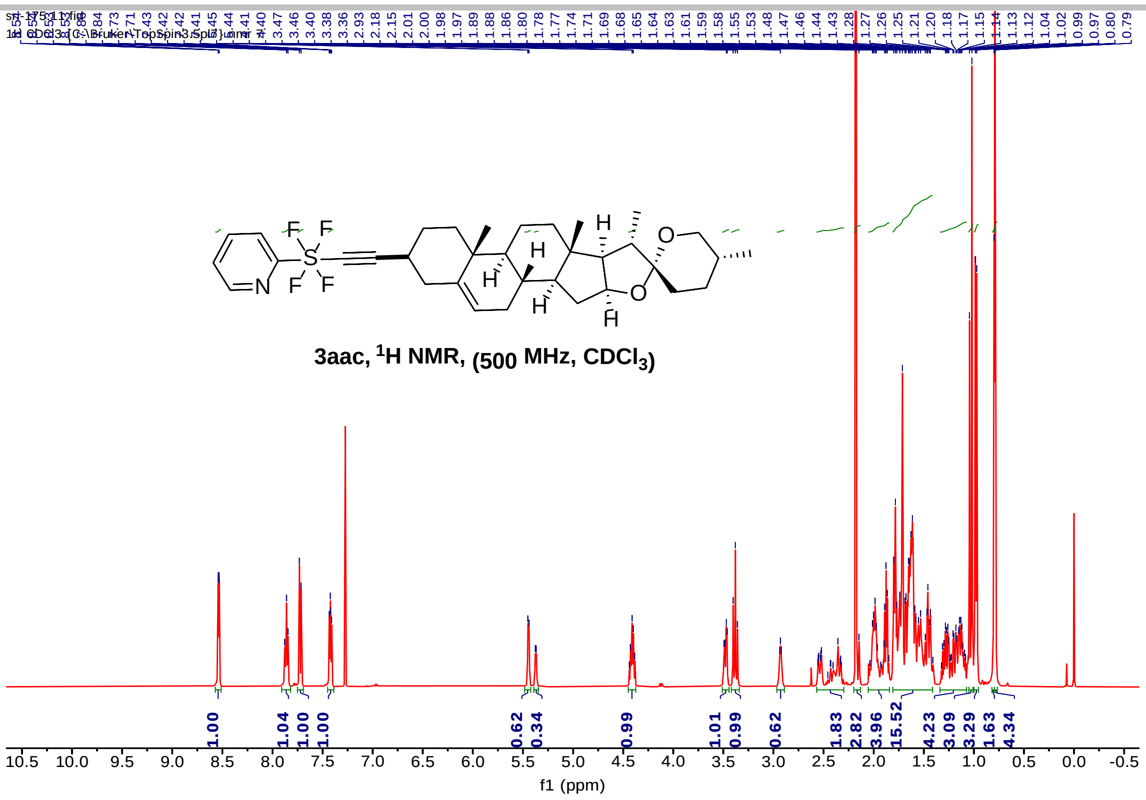
3aac, 1H NMR, (500 MHz, CDCl3)
f1 (ppm)

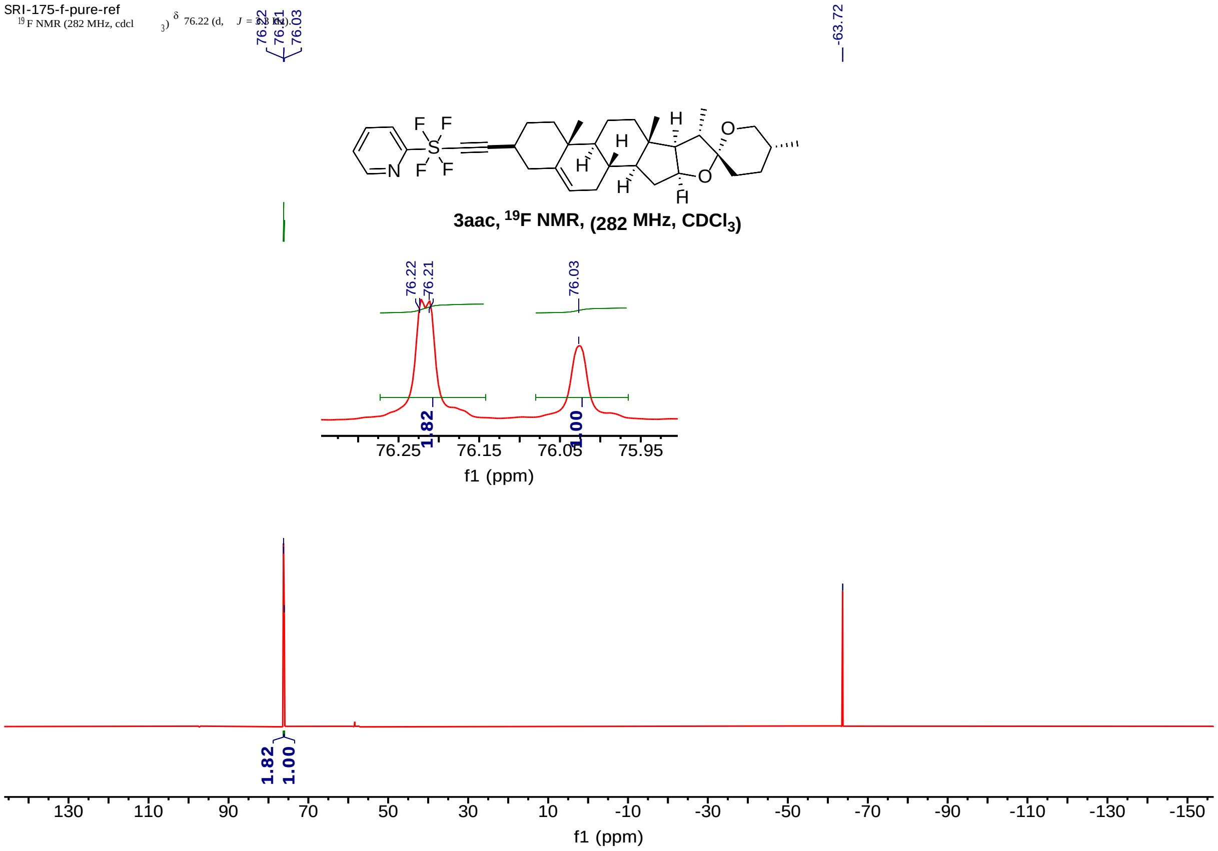
SRI-175-f-pure-ref
3aac, 19F NMR, (282 MHz, CDCl3)
f1 (ppm)

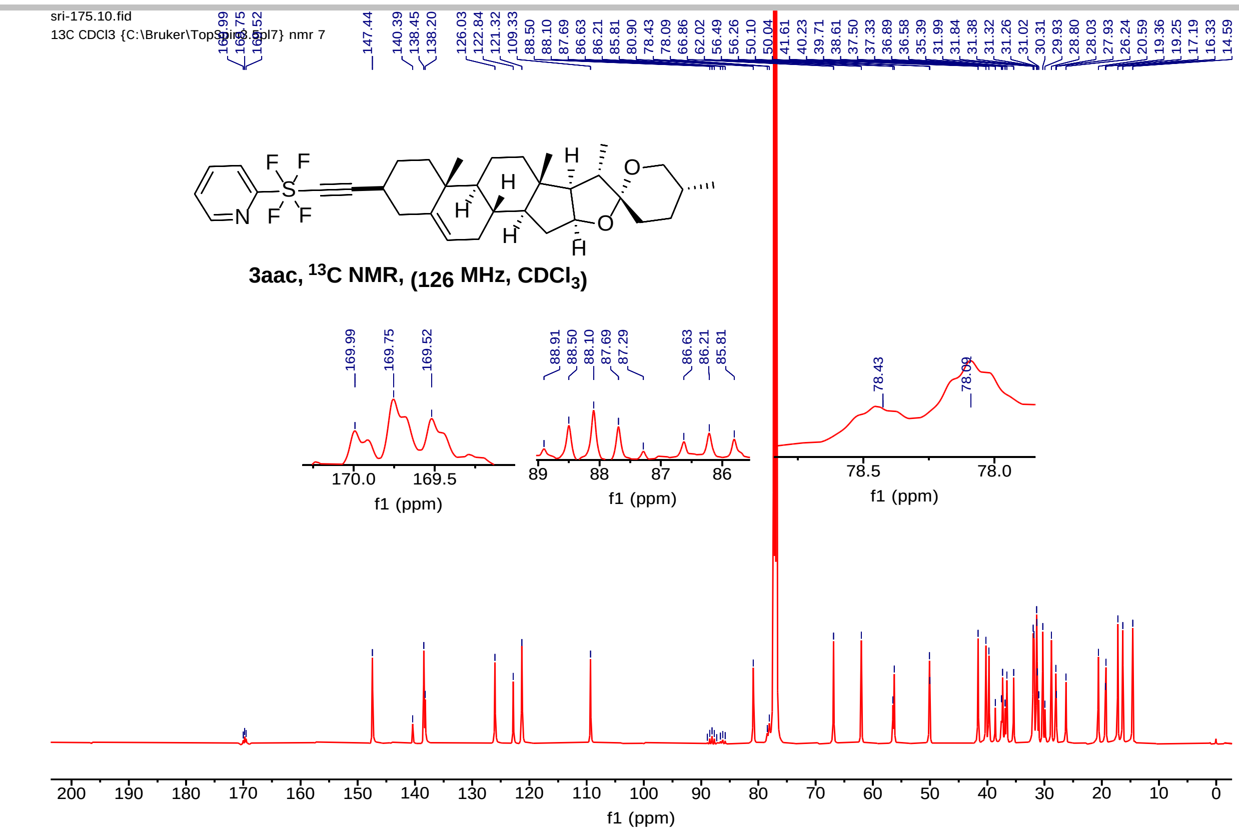
sri-175.10.fid
13C CDCl3 {C:\Bruker\TopSpin3.5pl7} nmr 7
3aac, ^{13}C NMR, (126 MHz, CDCl$_3$)
f1 (ppm)

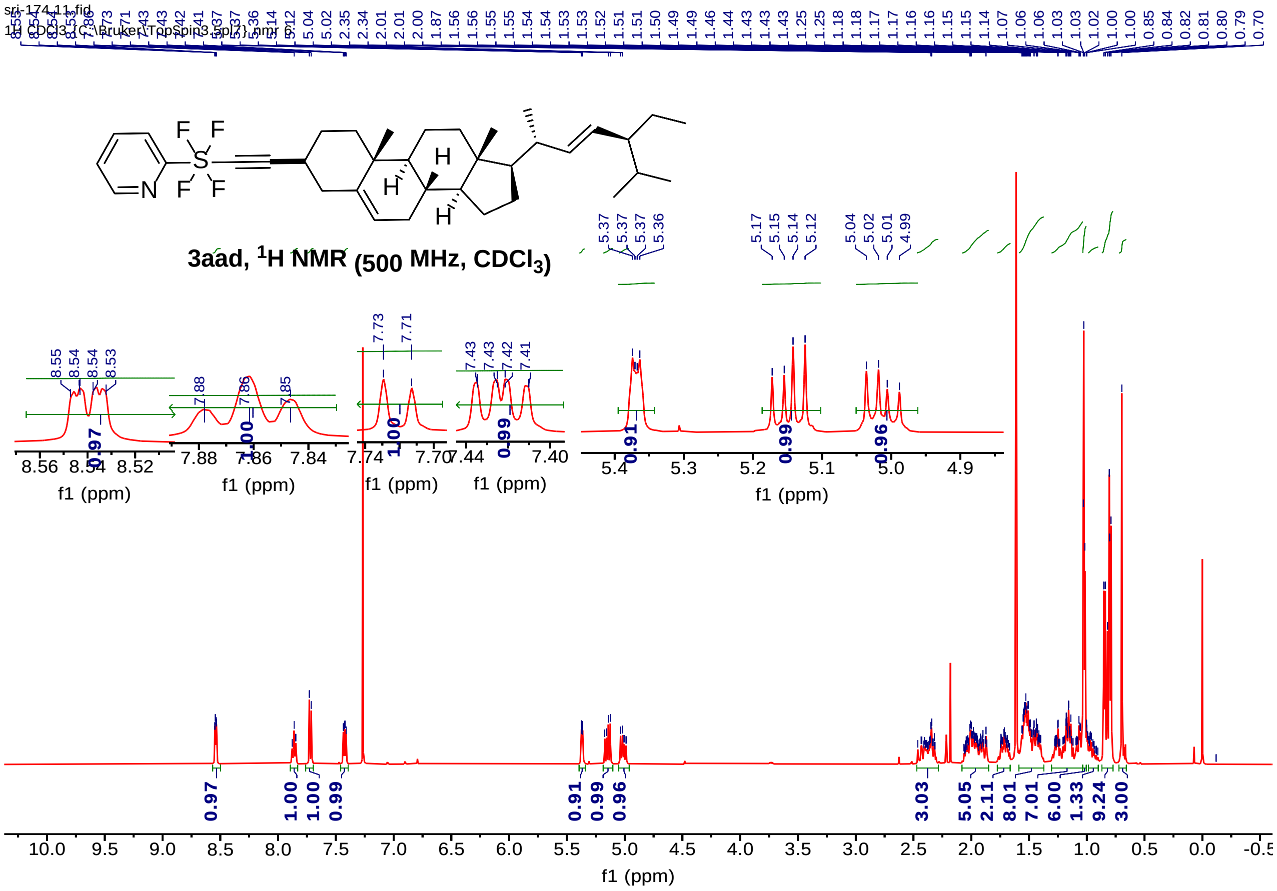
sri-174.11.fid
1H CDCl3 {C:\Bruker\TopSpin3.5pl7} nmr 5
3aad, ^{1}H NMR (500 MHz, CDCl$_3$)
f1 (ppm)

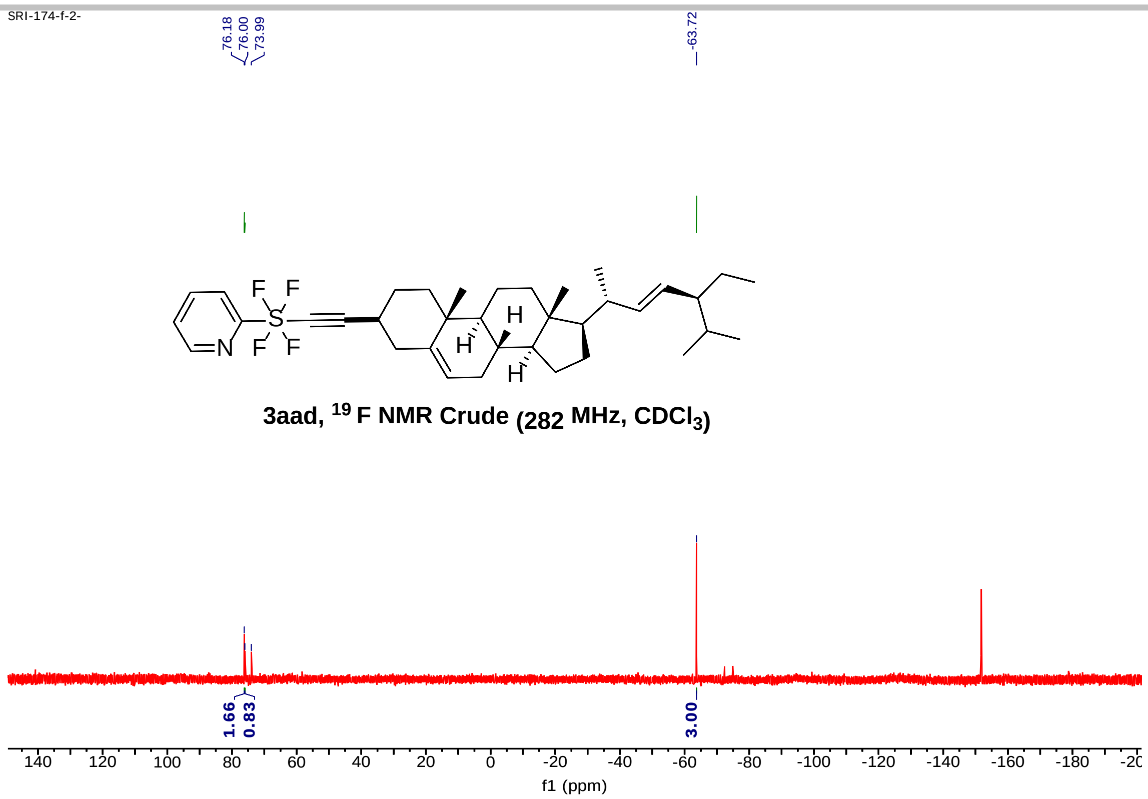
SRI-174-f-2-
76.18
76.00
73.99
-63.72
3aad, 19 F NMR Crude (282 MHz, CDCl3)
1.66
0.83
3.00
140 120 100 80 60 40 20 0 -20 -40 -60 -80 -100 -120 -140 -160 -180
f1 (ppm)

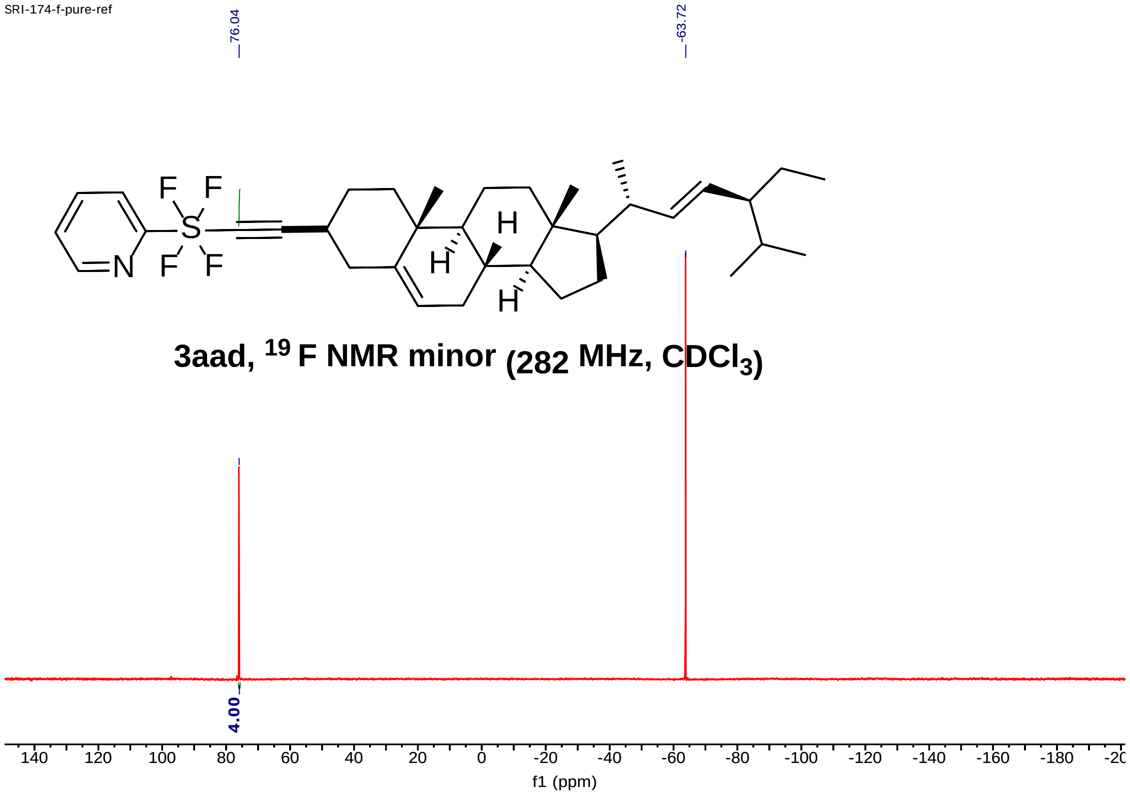
SRI-174-f-pure-ref
76.04
-63.72
3aad, 19 F NMR minor (282 MHz, CDCl3)
4.00
140 120 100 80 60 40 20 0 -20 -40 -60 -80 -100 -120 -140 -160 -180
f1 (ppm)

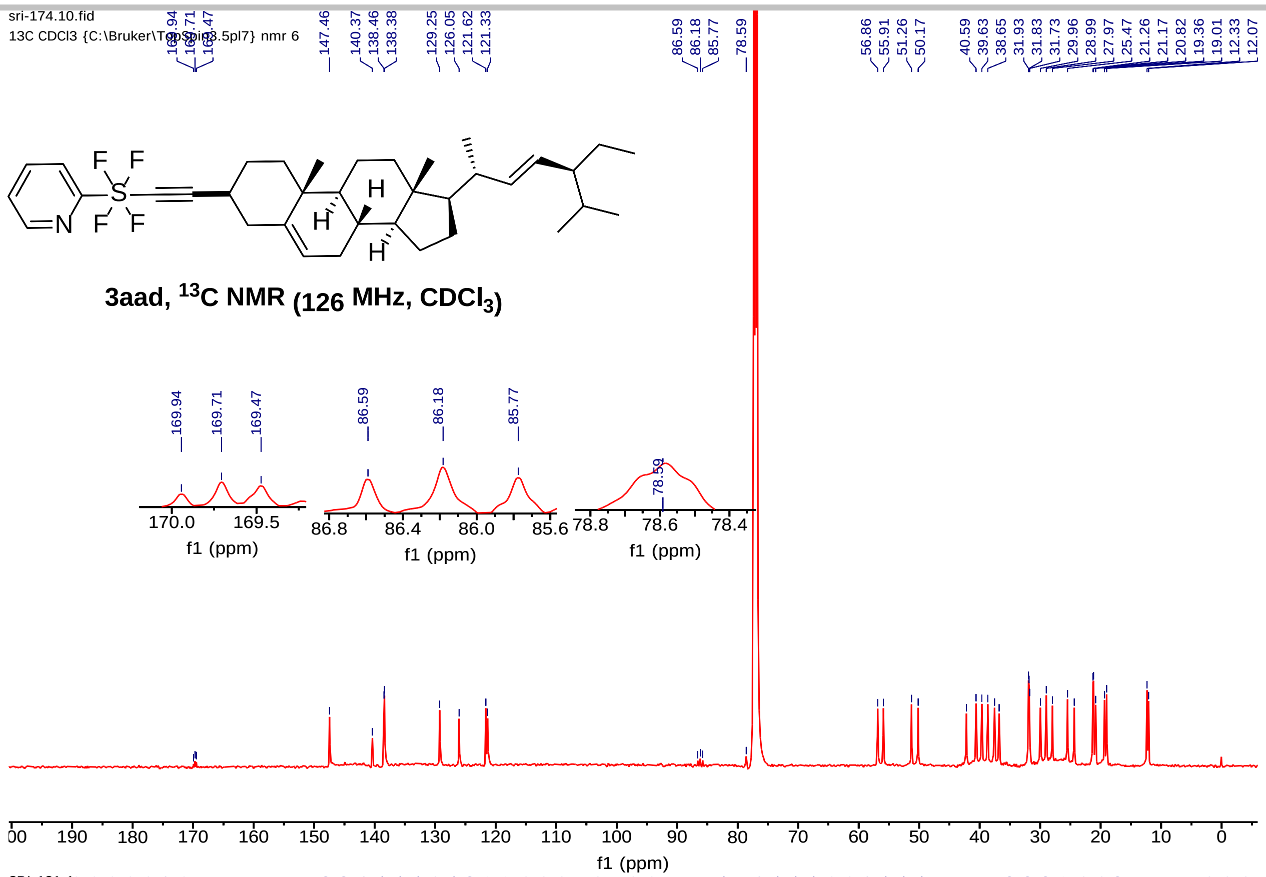
sri-174.10.fid
13C CDCl3 {C:\Bruker\TopSpin3.5pl7} nmr 6
3aad, ^{13}C NMR (126 MHz, $CDCl_3$)
f1 (ppm)

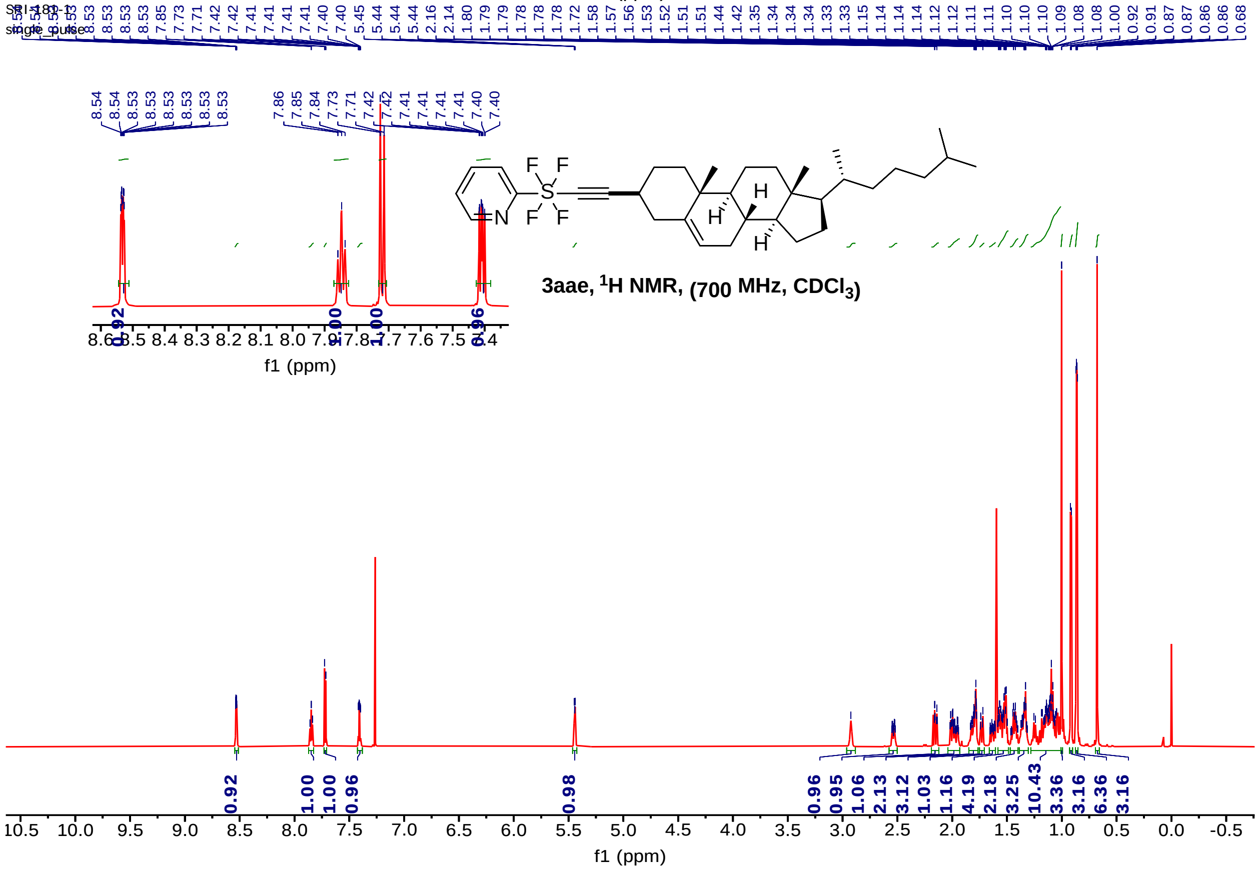
SRI-181-1
single_pulse
3aae, ^{1}H NMR, (700 MHz, $CDCl_3$)
f1 (ppm)

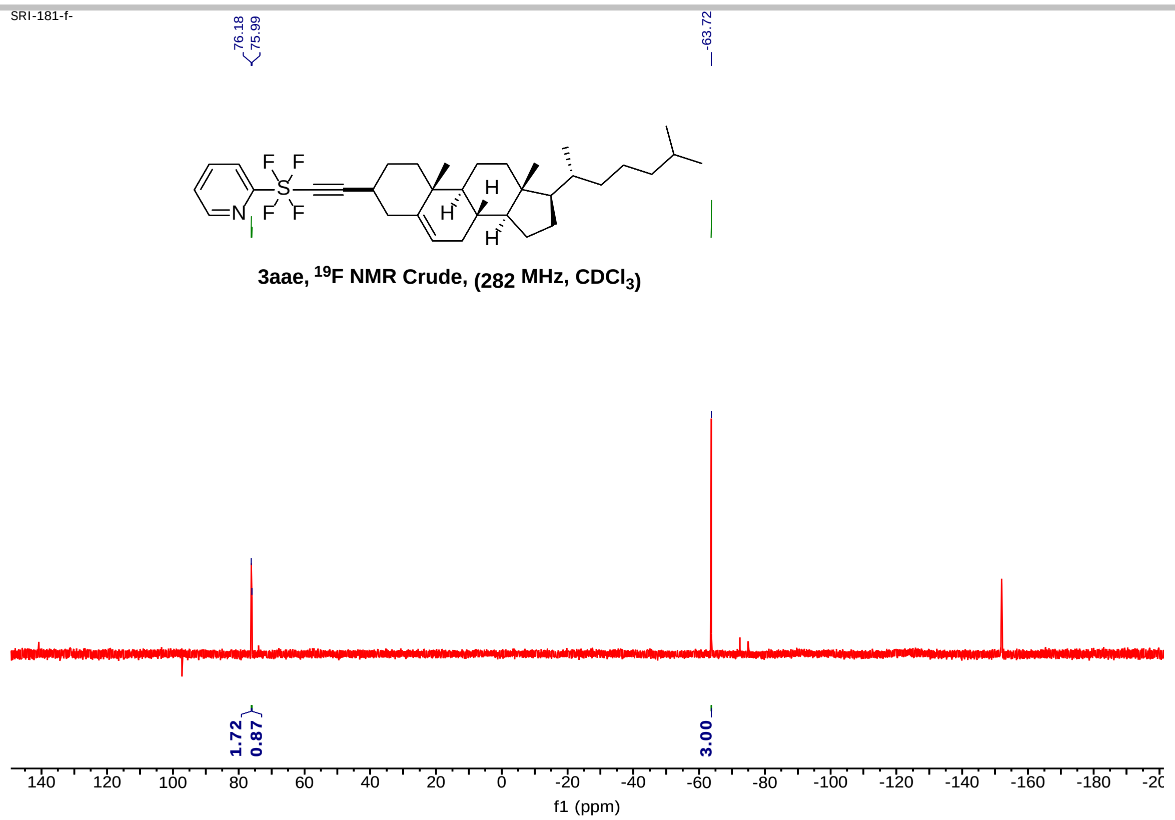

3aae, ^{19}F NMR Crude, (282 MHz, $CDCl_3$)

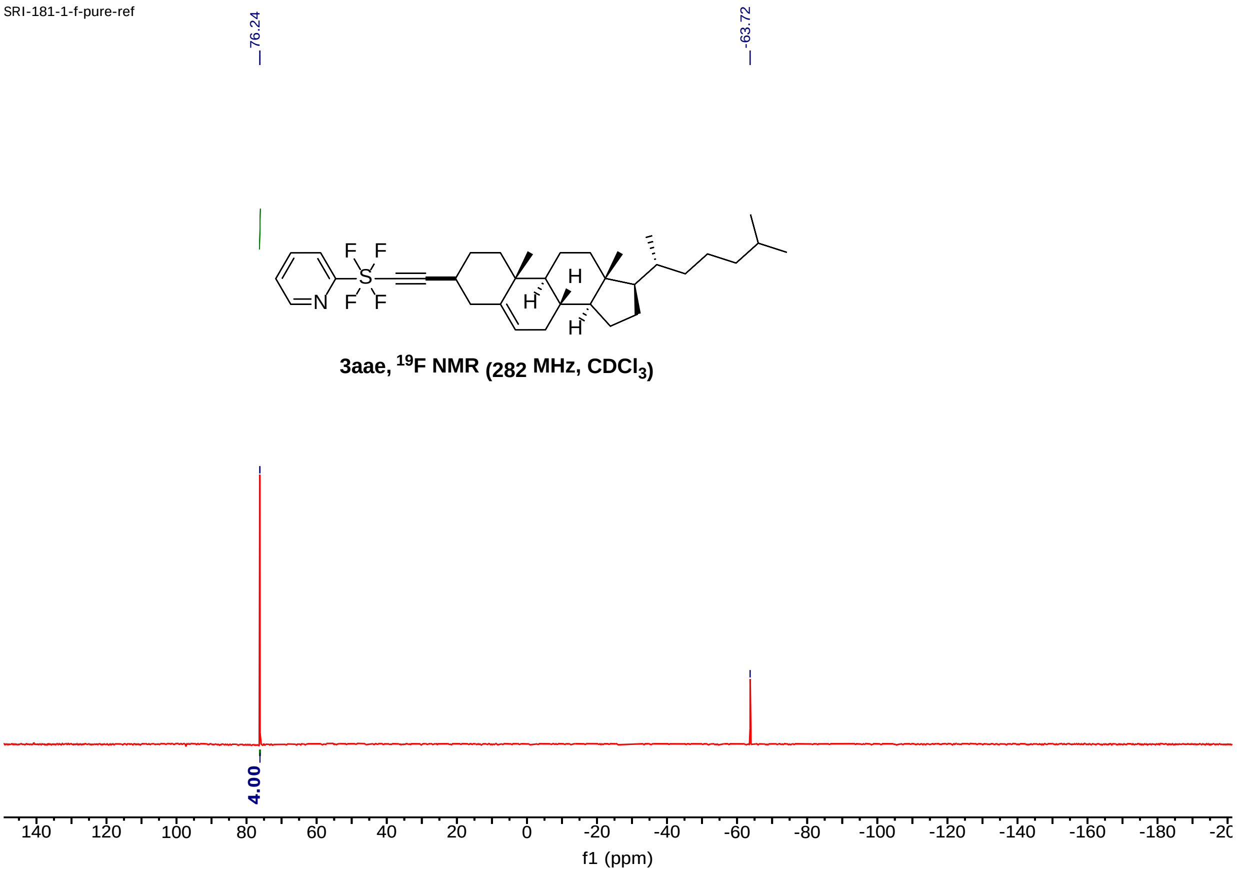

3aae, ^{19}F NMR (282 MHz, $CDCl_3$)

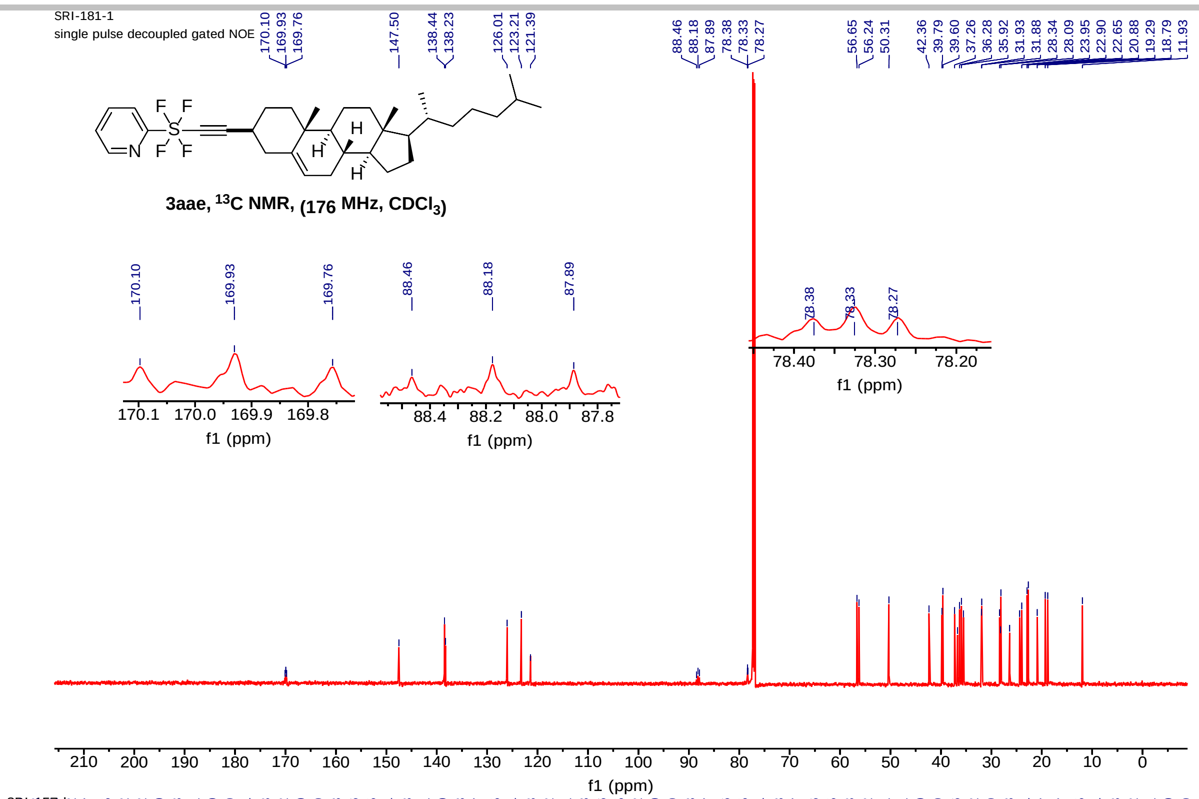
SRI-181-1
single pulse decoupled gated NOE
170.10
169.93
169.76
147.50
138.44
138.23
126.01
123.21
121.39
88.46
88.18
87.89
78.38
78.33
78.27
56.65
56.24
50.31
42.36
39.79
39.60
37.26
36.28
35.92
31.93
31.88
28.34
28.09
23.95
22.90
22.65
20.88
19.29
18.79
11.93
3aae, ¹³C NMR, (176 MHz, CDCl₃)
f1 (ppm)

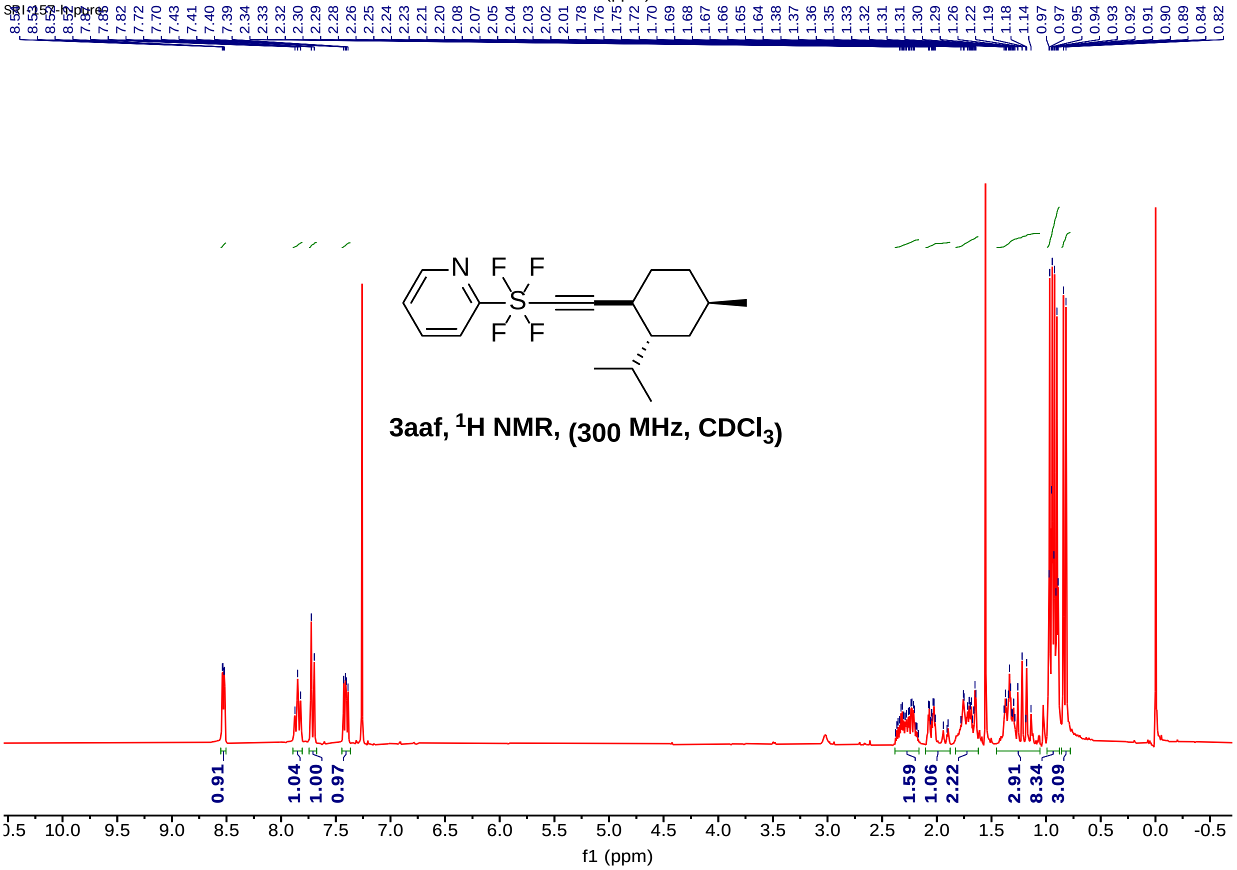
7.82
7.72
7.70
7.43
7.41
7.40
7.39
2.34
2.33
2.32
2.30
2.29
2.28
2.26
2.25
2.24
2.23
2.21
2.20
2.08
2.07
2.05
2.04
2.03
2.02
2.01
1.78
1.76
1.75
1.72
1.70
1.69
1.68
1.67
1.66
1.65
1.64
1.38
1.37
1.36
1.35
1.33
1.32
1.31
1.31
1.30
1.29
1.26
1.22
1.19
1.18
1.14
0.97
0.97
0.95
0.94
0.93
0.92
0.91
0.90
0.89
0.84
0.82
3aaf, ¹H NMR, (300 MHz, CDCl₃)
0.91
1.04
1.00
0.97
1.59
1.06
2.22
2.91
8.34
3.09
f1 (ppm)

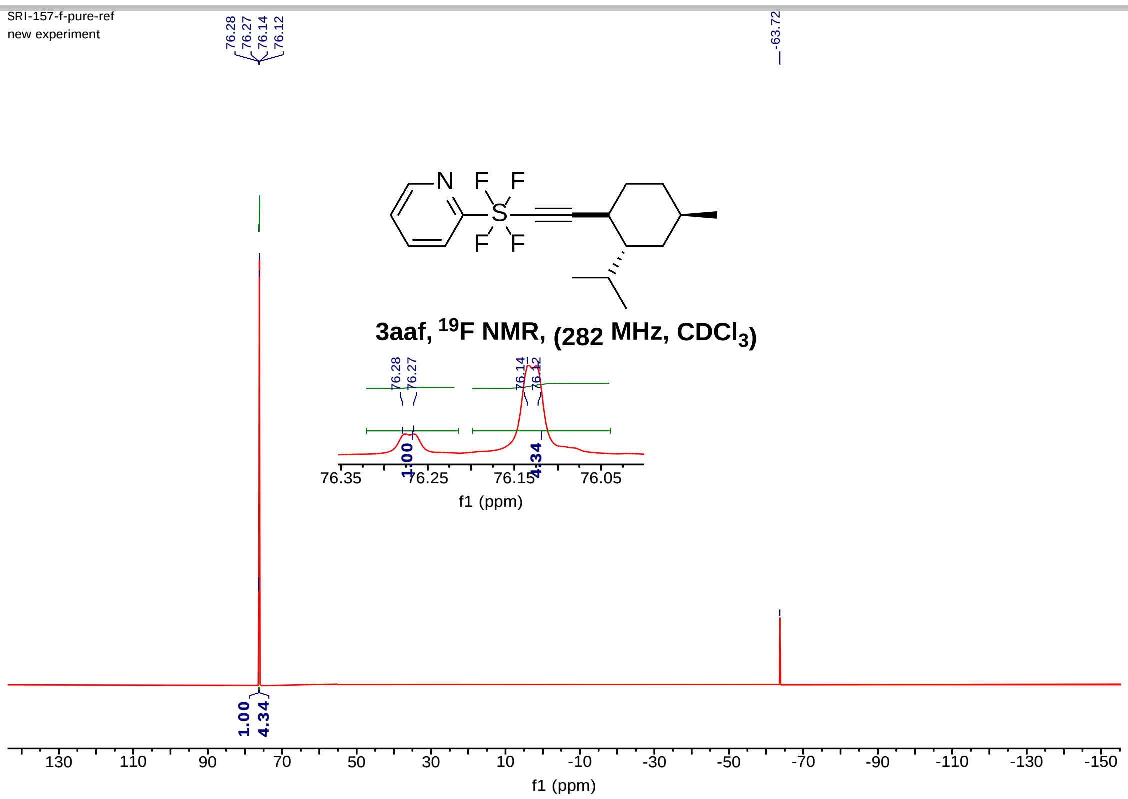
SRI-157-f-pure-ref
new experiment
3aaf, ^{19}F NMR, (282 MHz, CDCl$_3$)
f1 (ppm)

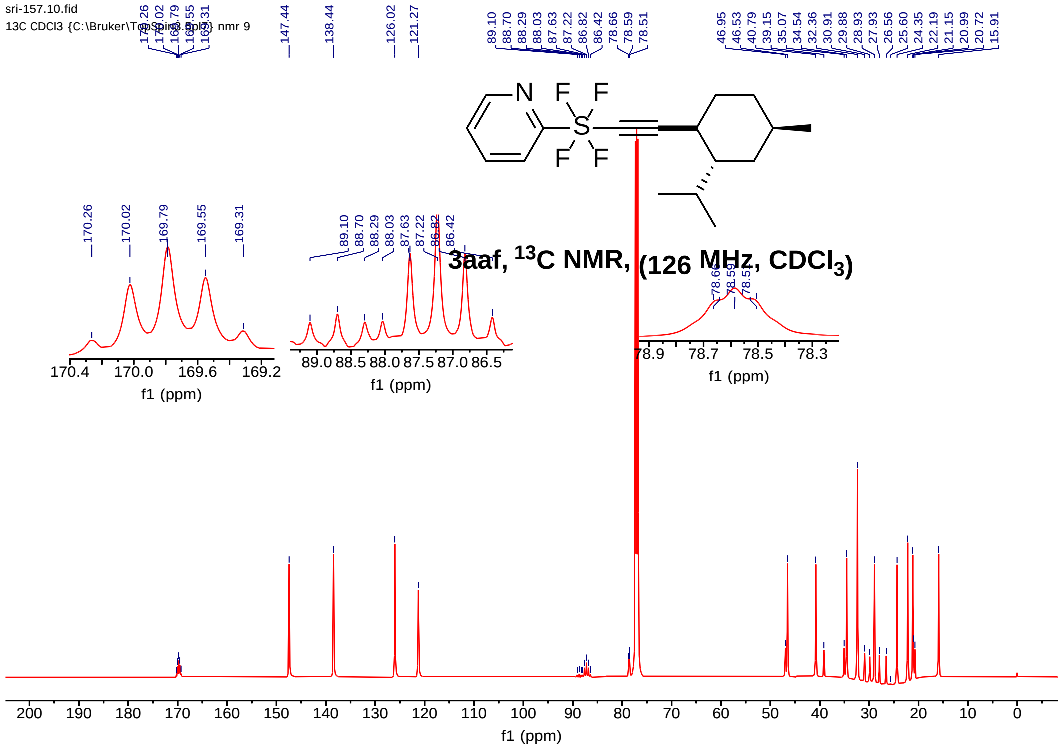
sri-157.10.fid
3aaf, ^{13}C NMR, (126 MHz, CDCl$_3$)
f1 (ppm)

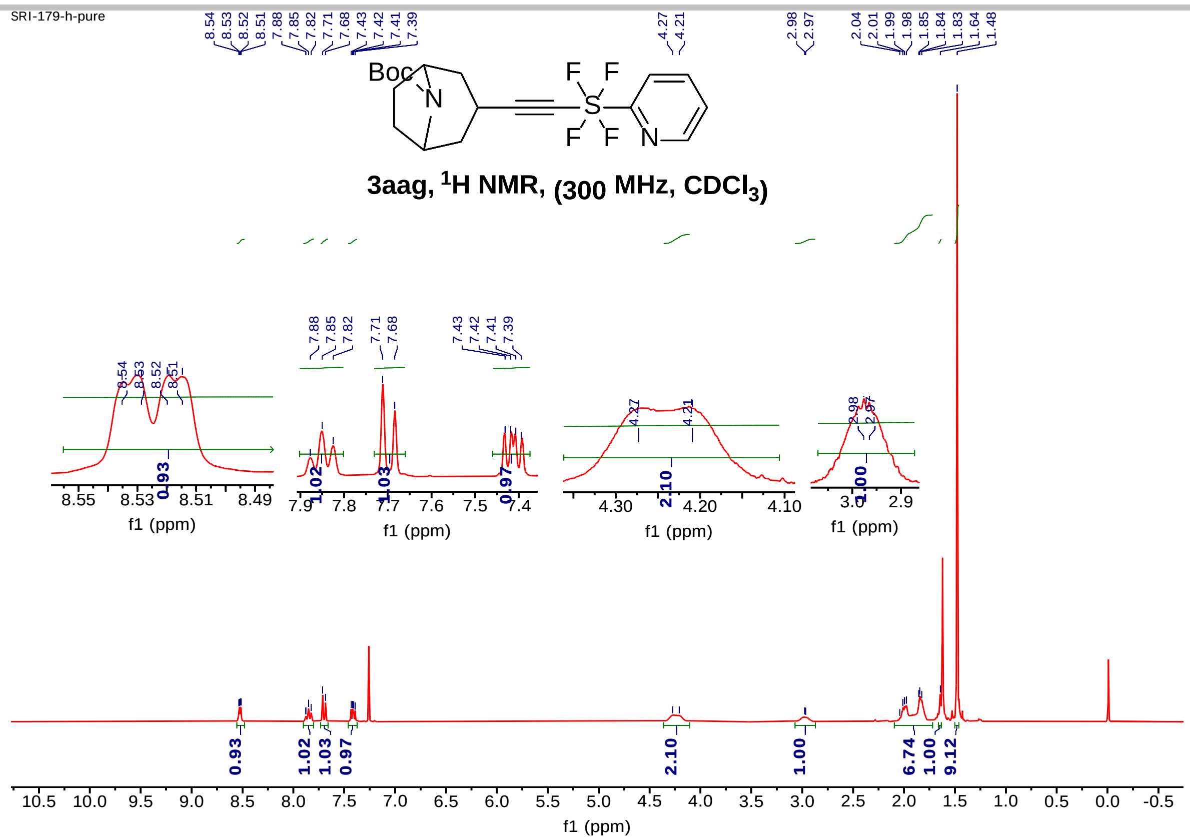
SRI-179-h-pure
3aag, 1H NMR, (300 MHz, CDCl3)
f1 (ppm)

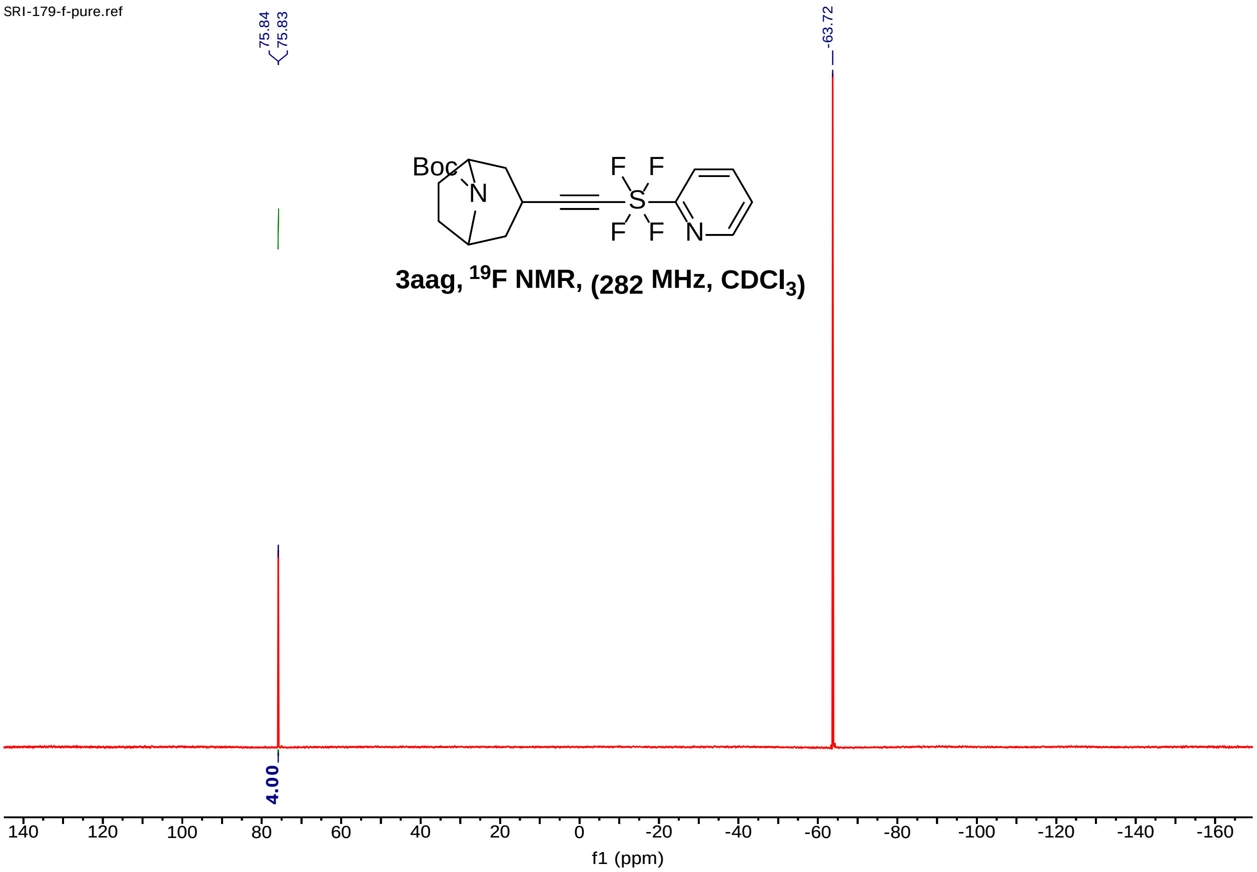
SRI-179-f-pure.ref
75.84
75.83
-63.72
3aag, 19F NMR, (282 MHz, CDCl3)
4.00
f1 (ppm)

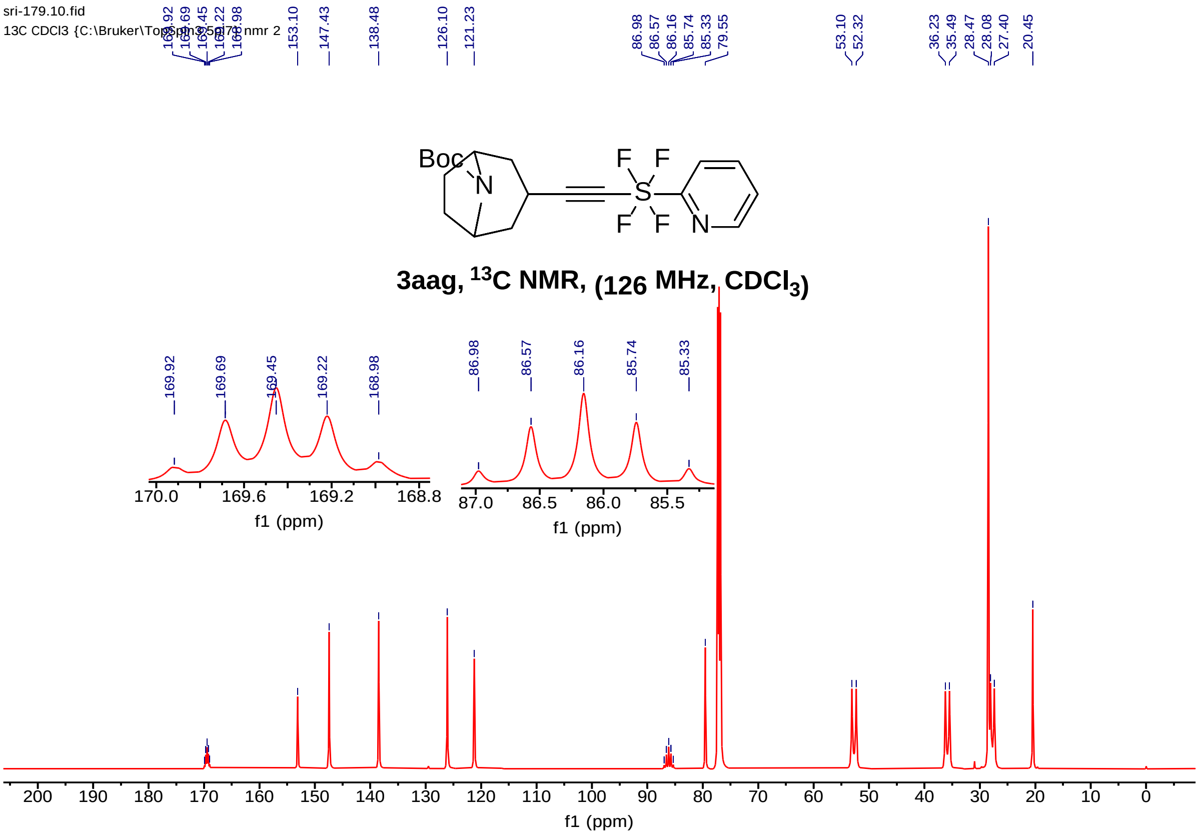
sri-179.10.fid
13C CDCl3 {C:\Bruker\TopSpin3.5pl7} nmr 2
169.92 169.69 169.45 169.22 168.98 153.10 147.43 138.48 126.10 121.23 86.98 86.57 86.16 85.74 85.33 79.55 53.10 52.32 36.23 35.49 28.47 28.08 27.40 20.45
Boc
N
F F
S
F F
N
3aag, 13C NMR, (126 MHz, CDCl3)
f1 (ppm)

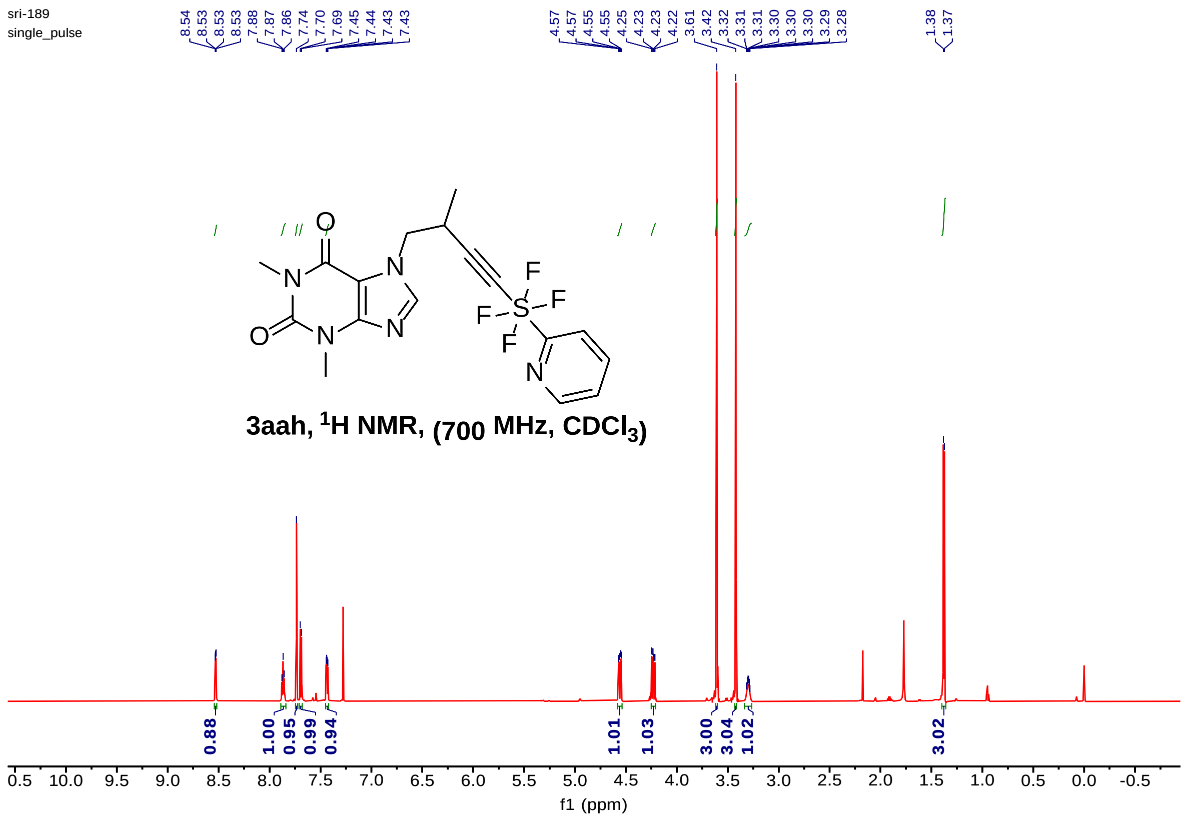
sri-189
single_pulse
8.54 8.53 8.53 8.53 7.88 7.87 7.86 7.74 7.70 7.69 7.45 7.44 7.43 7.43 4.57 4.57 4.55 4.55 4.25 4.23 4.23 4.22 3.61 3.42 3.32 3.31 3.31 3.30 3.30 3.29 3.28 1.38 1.37
0.88 1.00 0.95 0.99 0.94 1.01 1.03 3.00 3.04 1.02 3.02
3aah, 1H NMR, (700 MHz, CDCl3)
f1 (ppm)

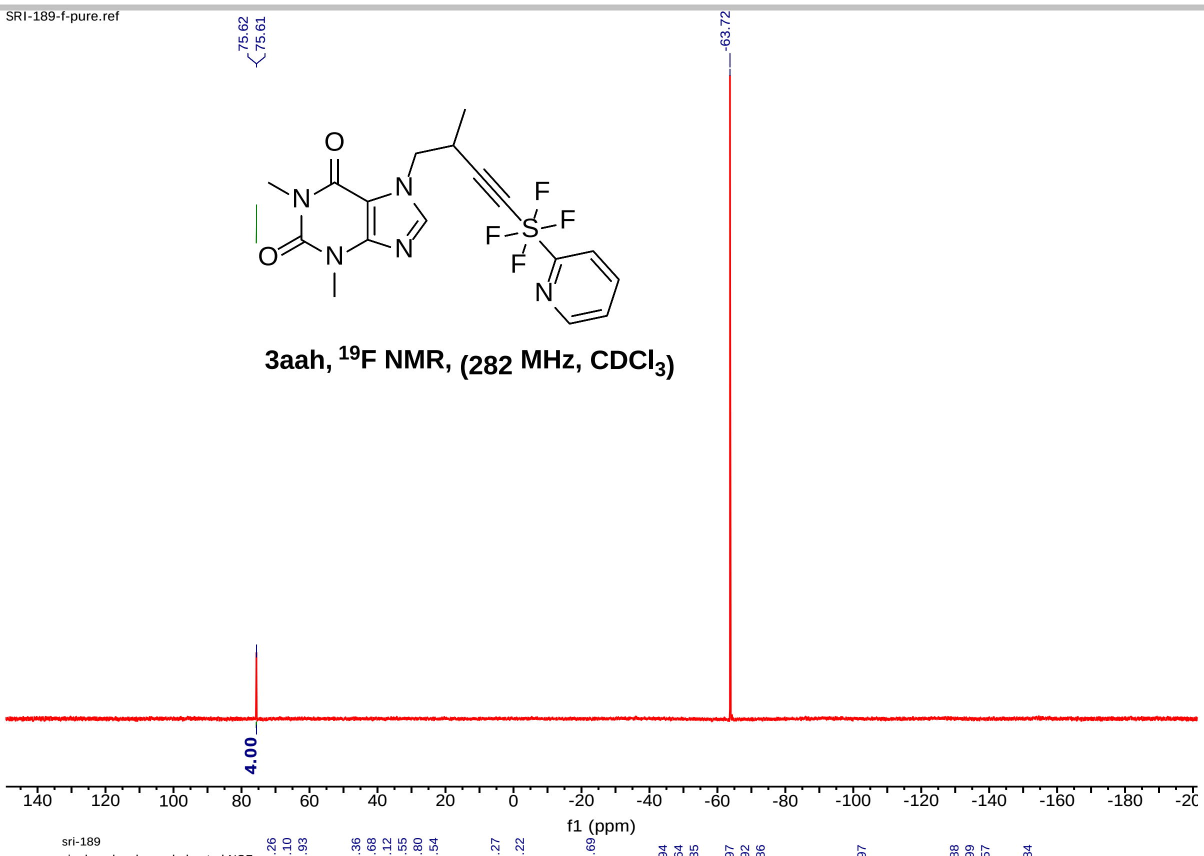
SRI-189-f-pure.ref
75.62
75.61
-63.72
3aah, ¹⁹F NMR, (282 MHz, CDCl₃)
4.00
f1 (ppm)

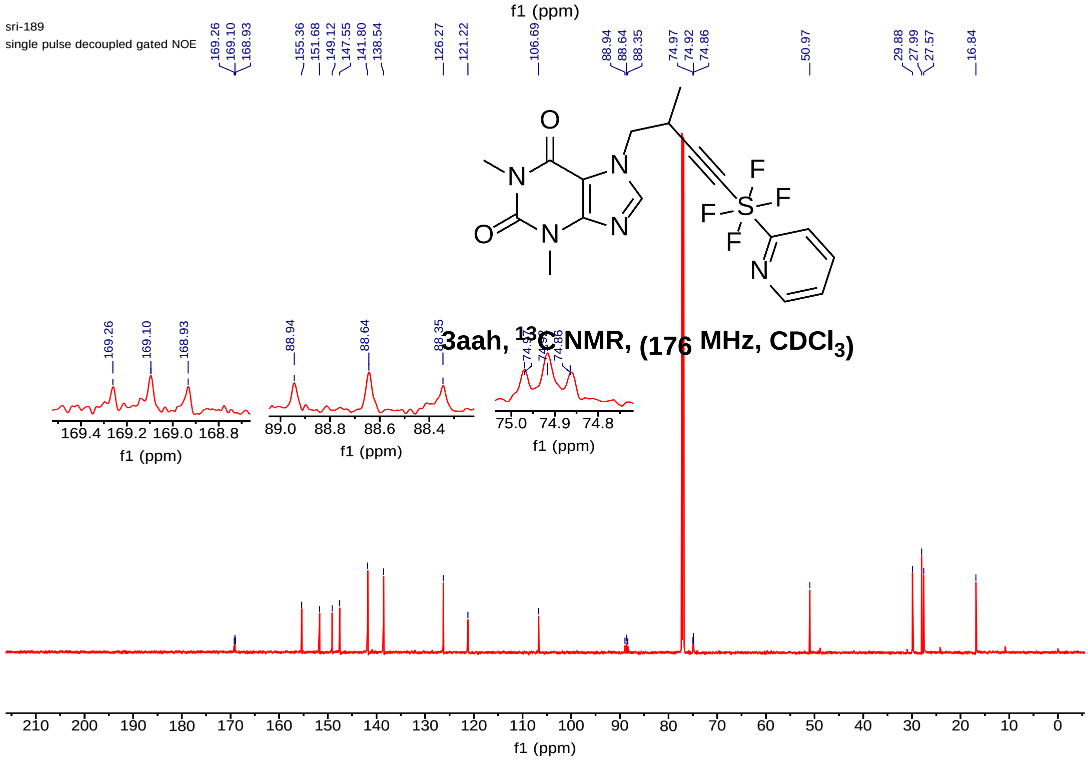
sri-189
single pulse decoupled gated NOE
169.26
169.10
168.93
155.36
151.68
149.12
147.55
141.80
138.54
126.27
121.22
106.69
88.94
88.64
88.35
74.97
74.92
74.86
50.97
29.88
27.99
27.57
16.84
3aah, ¹³C NMR, (176 MHz, CDCl₃)
f1 (ppm)

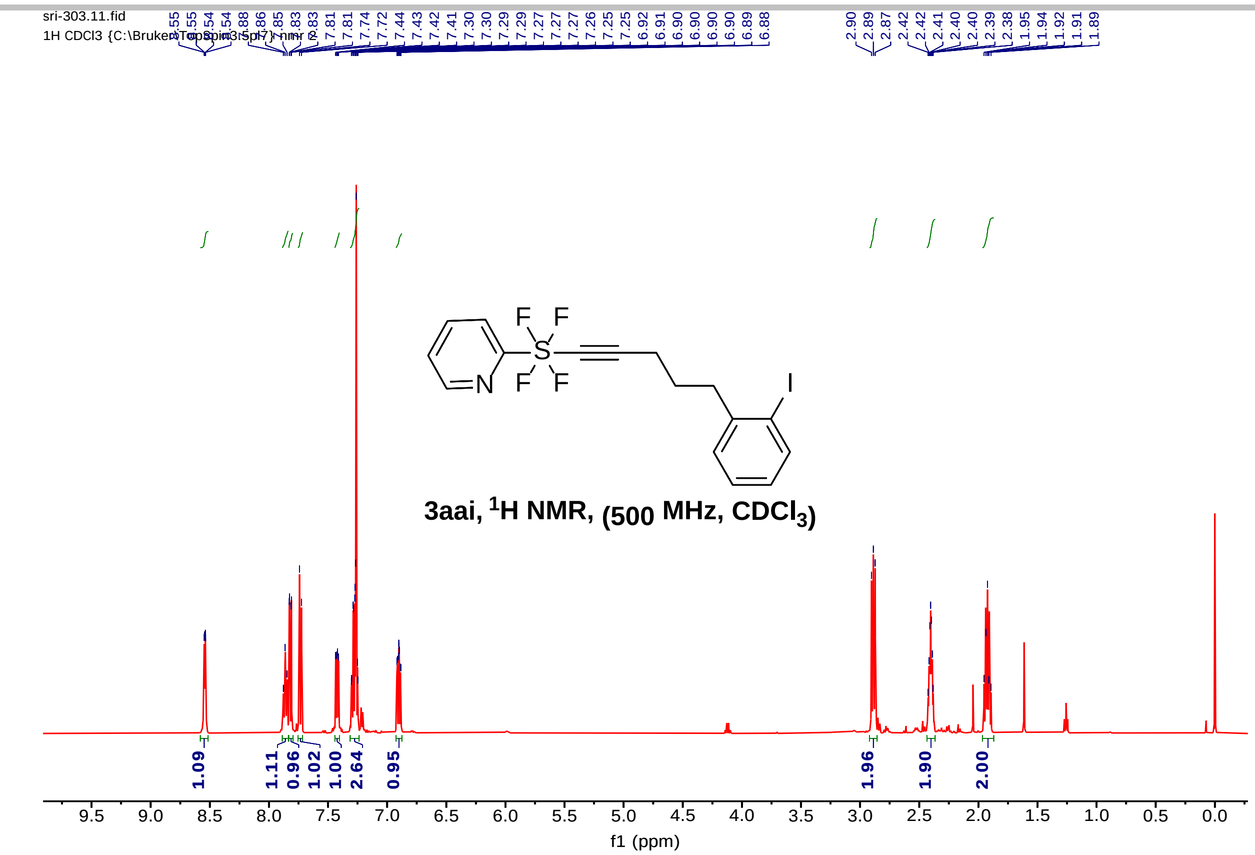
sri-303.11.fid
1H CDCl3 {C:\Bruker\TopSpin3.5pl7} nmr 2
8.55
8.55
8.54
8.54
7.88
7.86
7.85
7.83
7.83
7.81
7.81
7.74
7.72
7.44
7.43
7.42
7.41
7.30
7.30
7.29
7.29
7.27
7.27
7.27
7.26
7.25
7.25
6.92
6.91
6.90
6.90
6.90
6.90
6.89
6.88
2.90
2.89
2.87
2.42
2.42
2.41
2.40
2.40
2.39
2.38
1.95
1.94
1.92
1.91
1.89
3aai, ^{1}H NMR, (500 MHz, $CDCl_3$)
1.09
1.11
0.96
1.02
1.00
2.64
0.95
1.96
1.90
2.00
f1 (ppm)

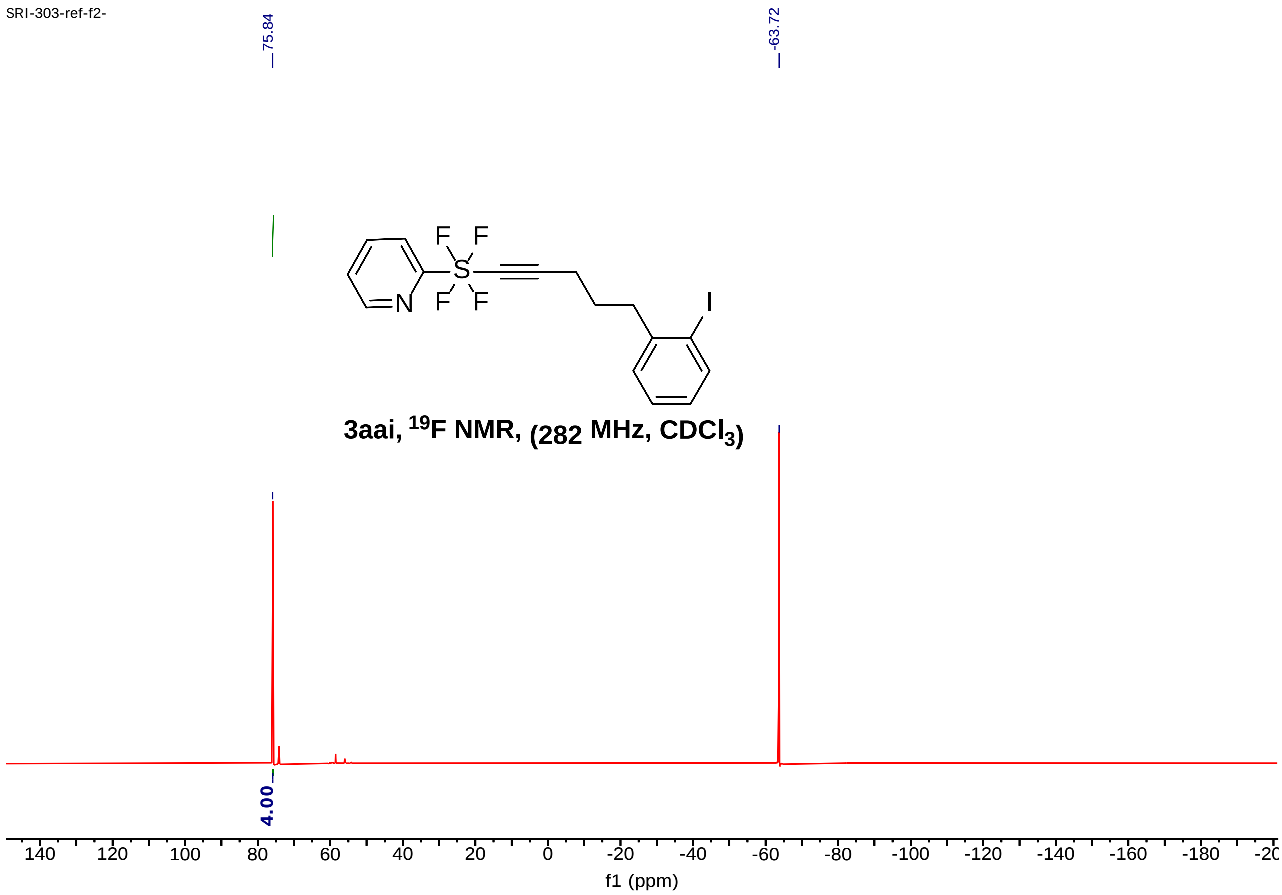
SRI-303-ref-f2-
-75.84
-63.72
3aai, ^{19}F NMR, (282 MHz, $CDCl_3$)
4.00
f1 (ppm)

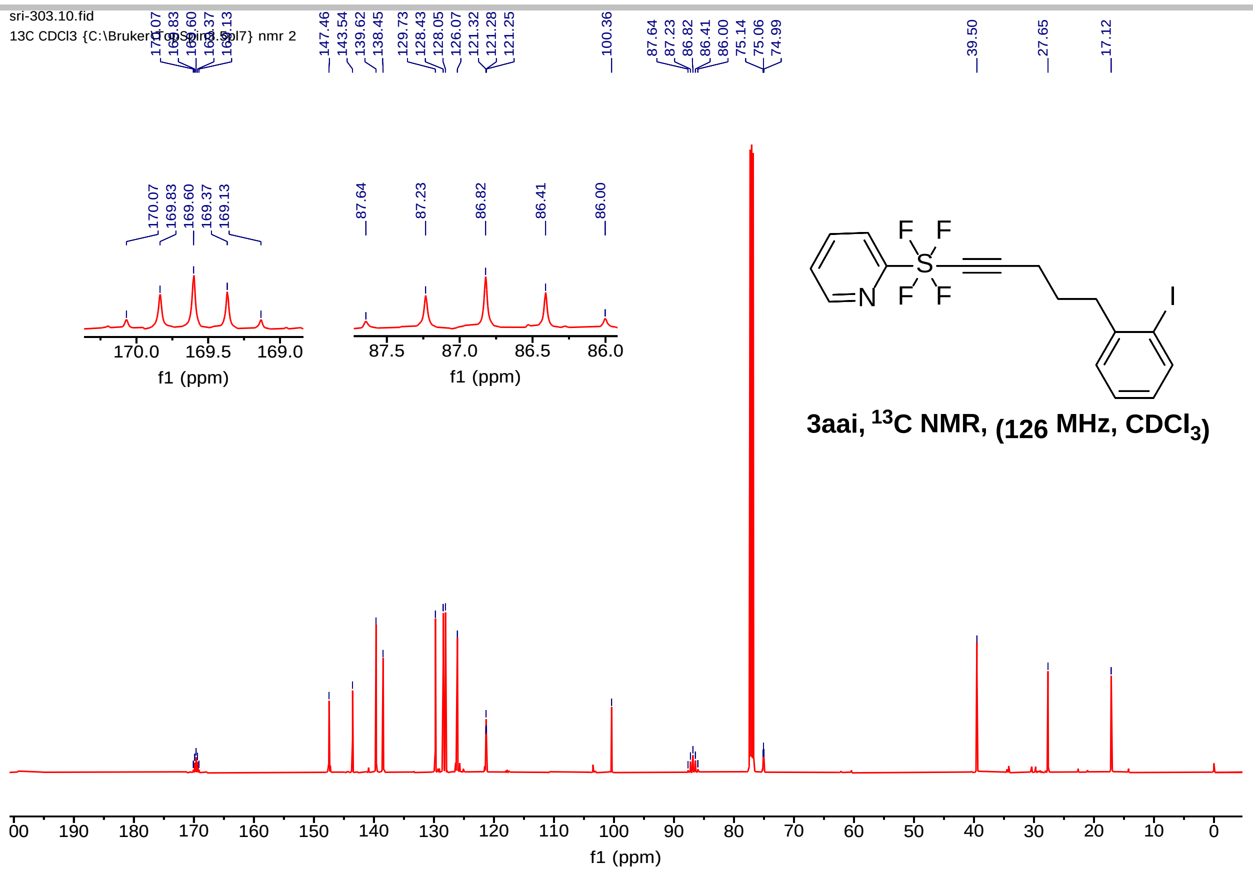
sri-303.10.fid
13C CDCl3 {C:\Bruker\TopSpin3.5pl7} nmr 2
3aai, 13C NMR, (126 MHz, CDCl3)
f1 (ppm)

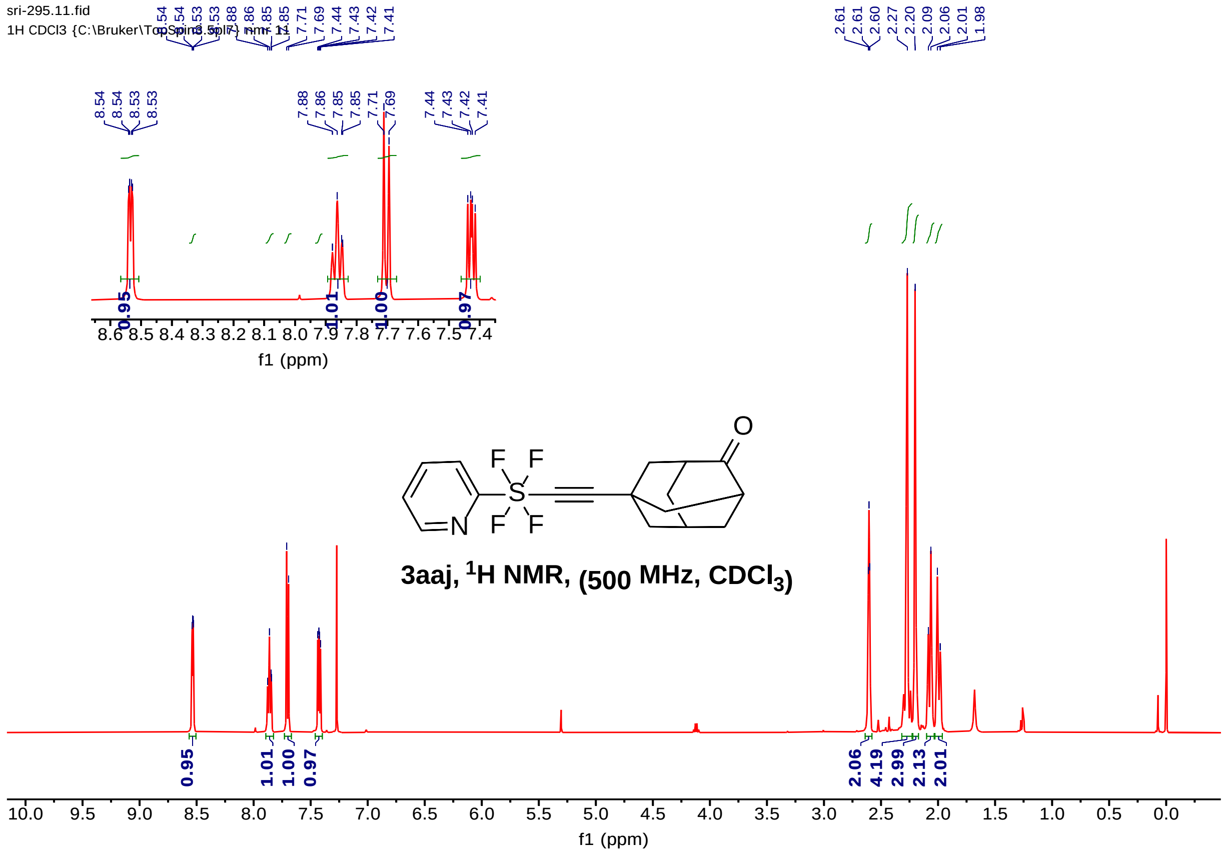
sri-295.11.fid
1H CDCl3 {C:\Bruker\TopSpin3.5pl7} nmr 11
3aaj, 1H NMR, (500 MHz, CDCl3)
f1 (ppm)

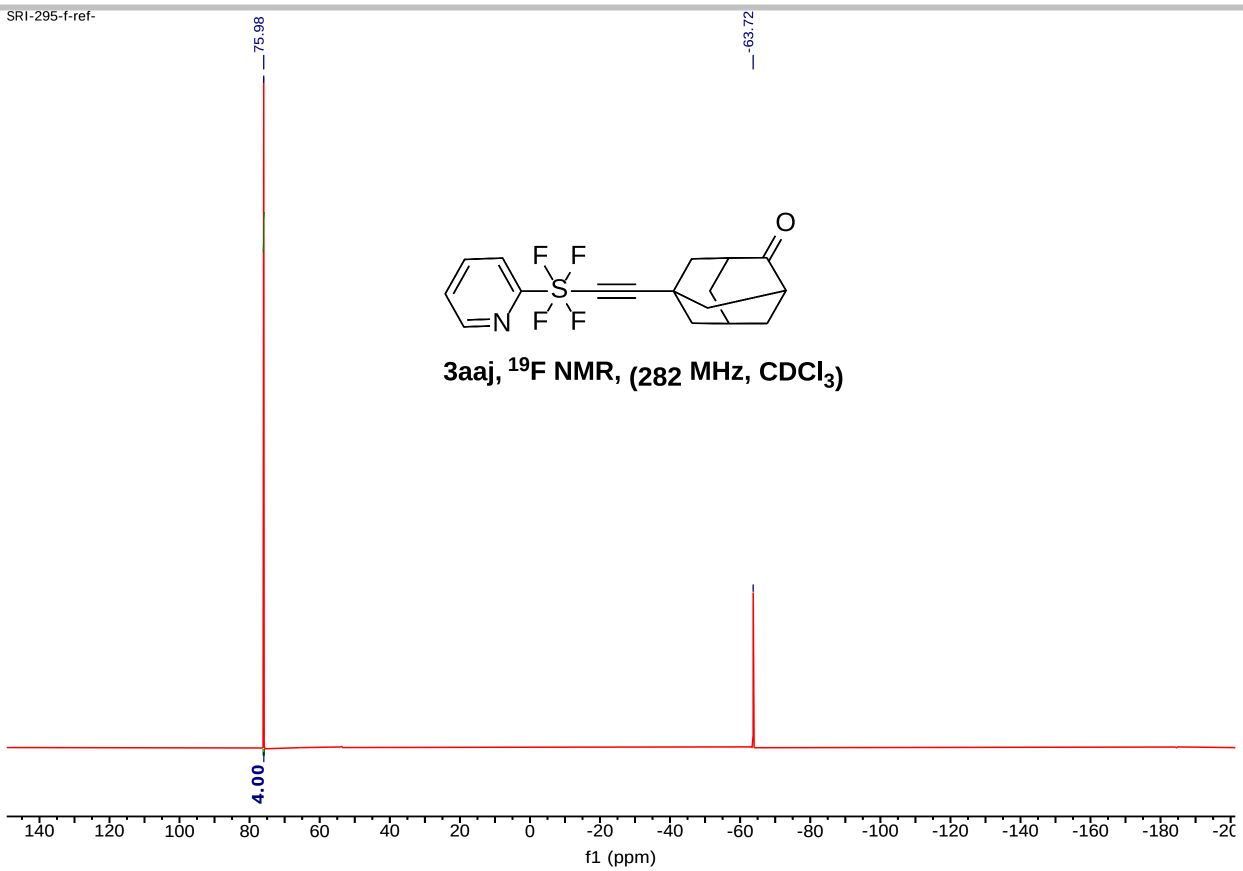
SRI-295-f-ref-
75.98
-63.72
3aaj, ^{19}F NMR, (282 MHz, $CDCl_3$)
4.00
f1 (ppm)

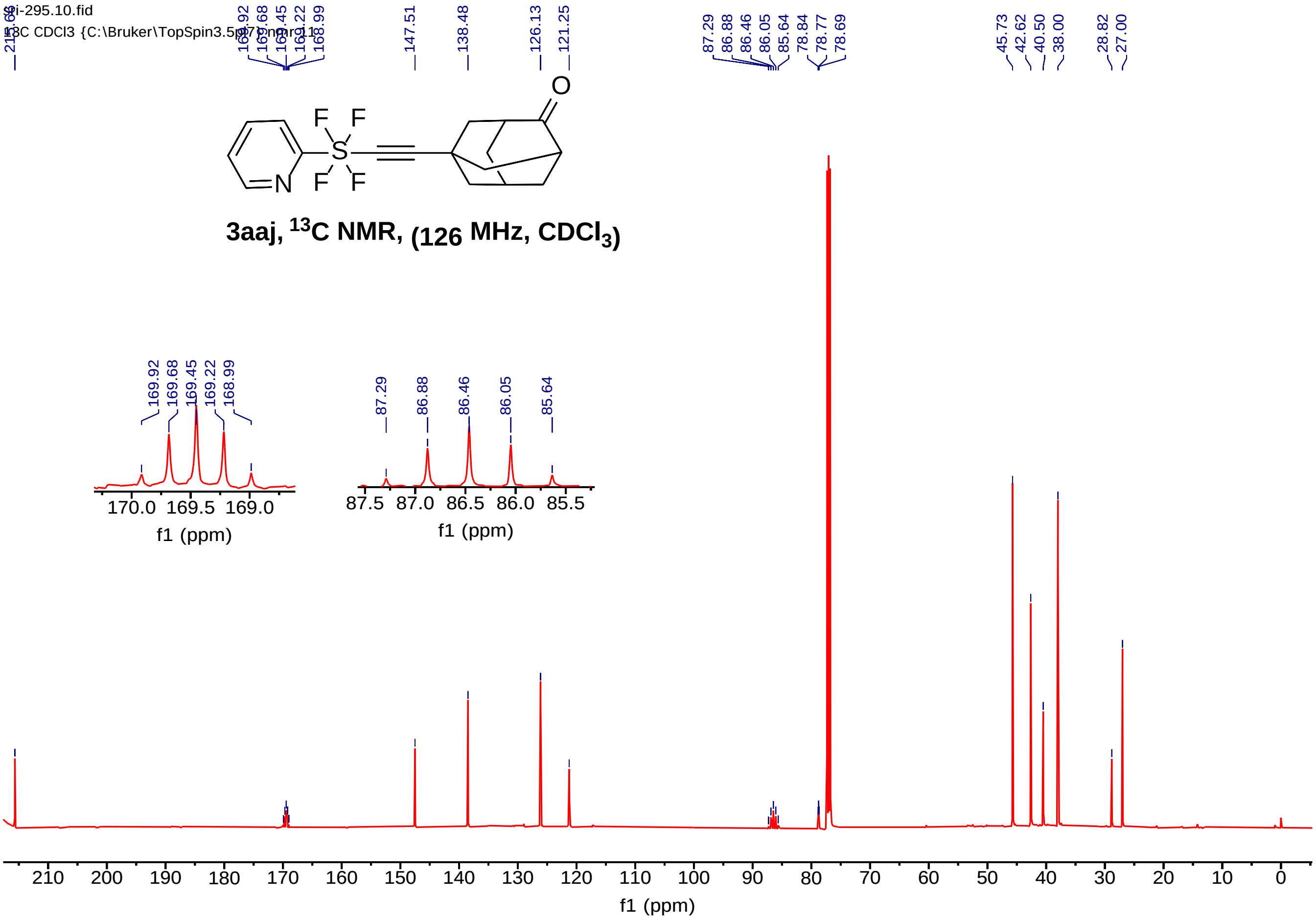
sri-295.10.fid
13C CDCl3 {C:\Bruker\TopSpin3.5pl7\nmr}11
169.92
169.68
169.45
169.22
168.99
147.51
138.48
126.13
121.25
87.29
86.88
86.46
86.05
85.64
78.84
78.77
78.69
45.73
42.62
40.50
38.00
28.82
27.00
3aaj, ^{13}C NMR, (126 MHz, $CDCl_3$)
f1 (ppm)

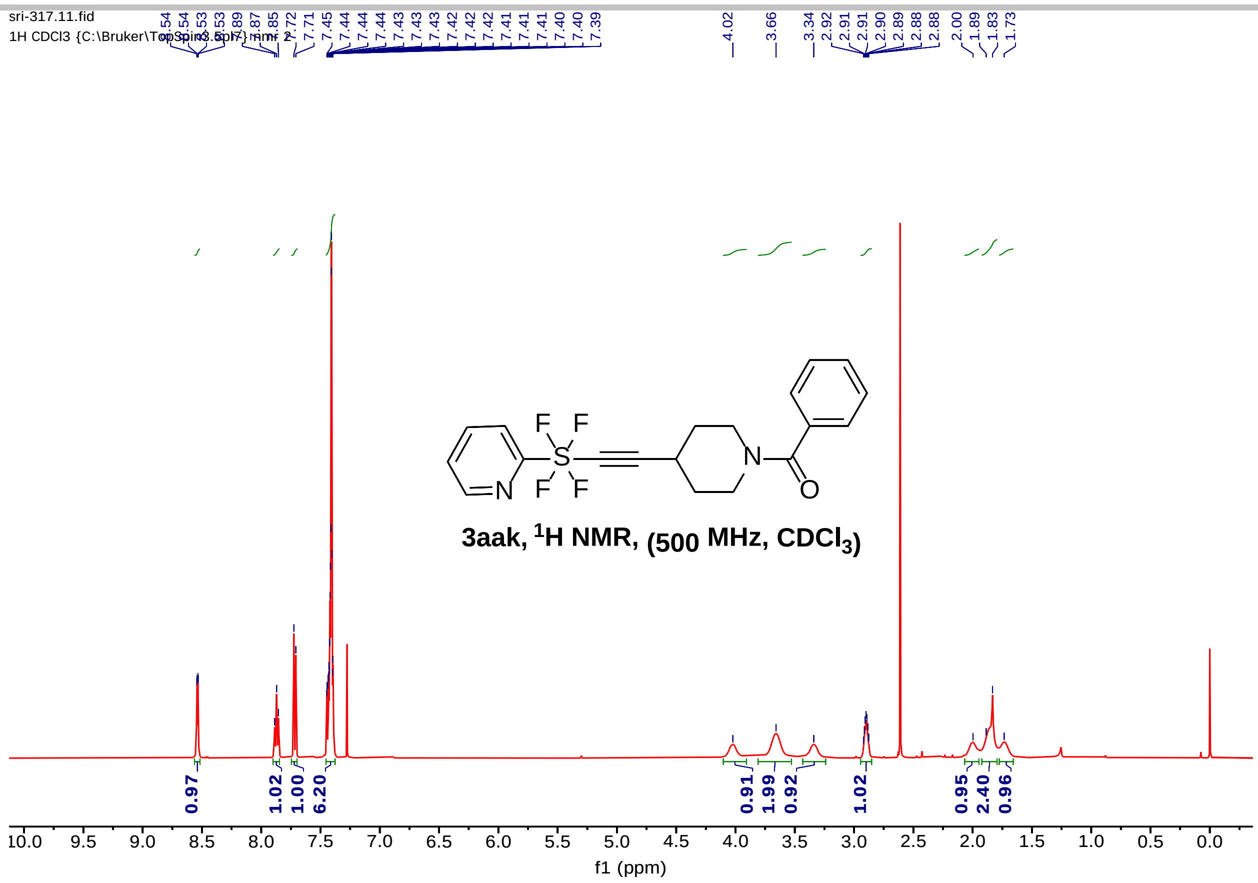
sri-317.11.fid
1H CDCl3 {C:\Bruker\TopSpin3.5pl7} nmr 2
3aak, 1H NMR, (500 MHz, CDCl3)
f1 (ppm)

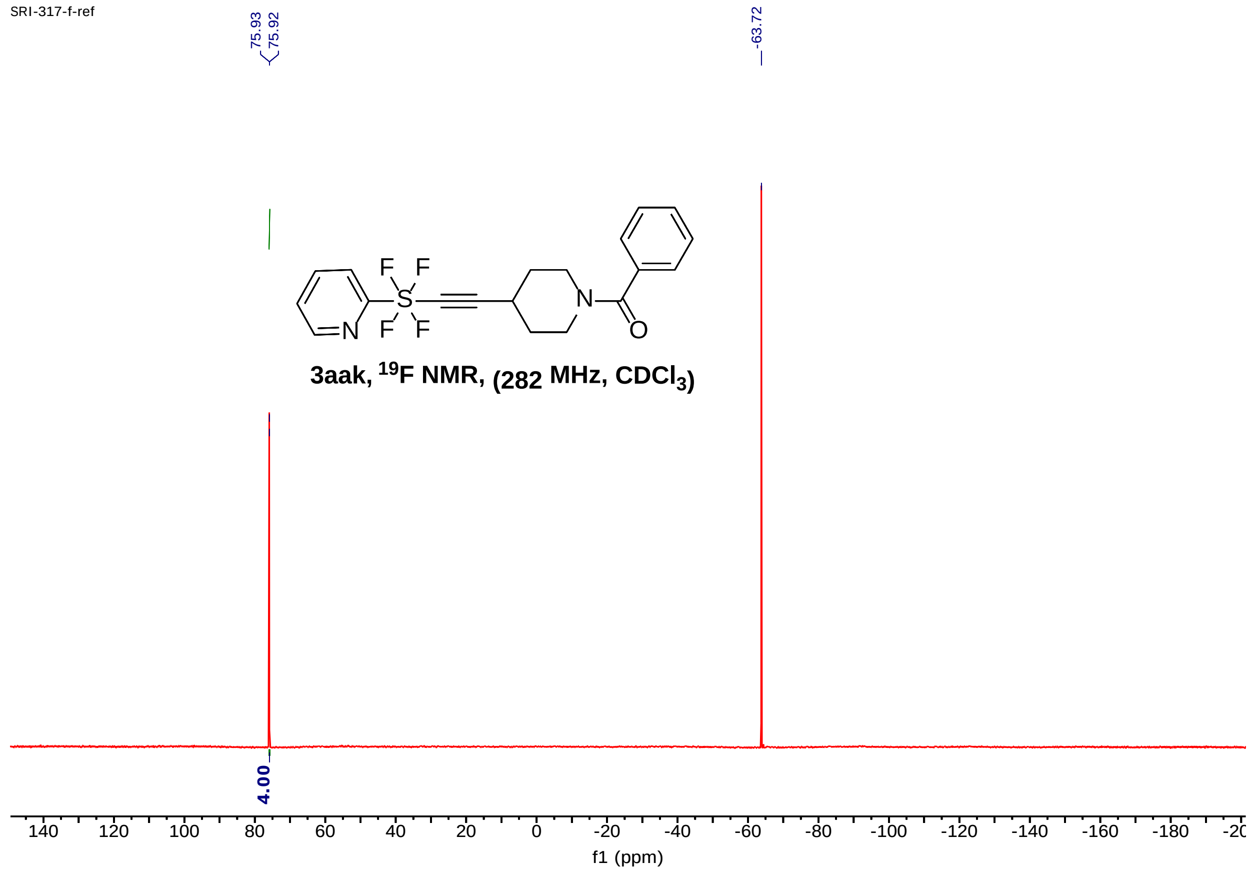
SRI-317-f-ref
3aak, 19F NMR, (282 MHz, CDCl3)
f1 (ppm)

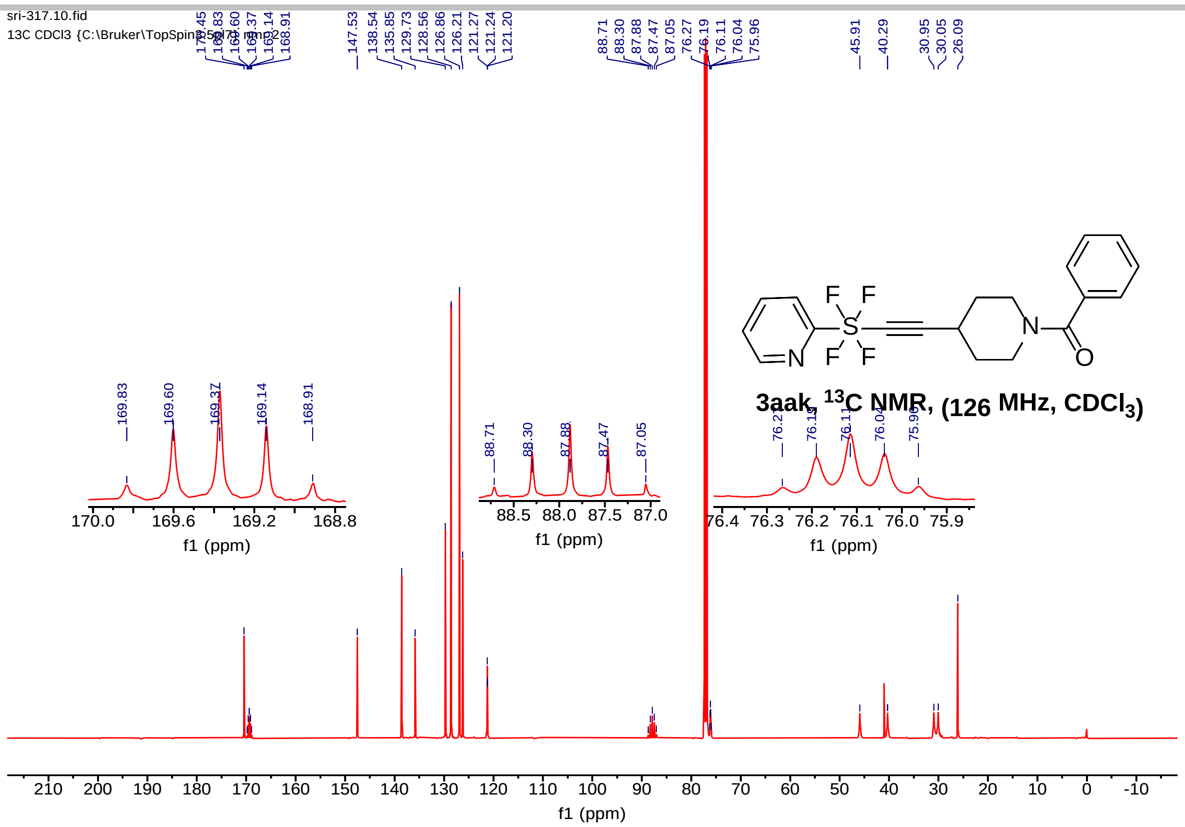
sri-317.10.fid
13C CDCl3 {C:\Bruker\TopSpin3.5pl7} nmr
3aak, 13C NMR, (126 MHz, CDCl3)
f1 (ppm)

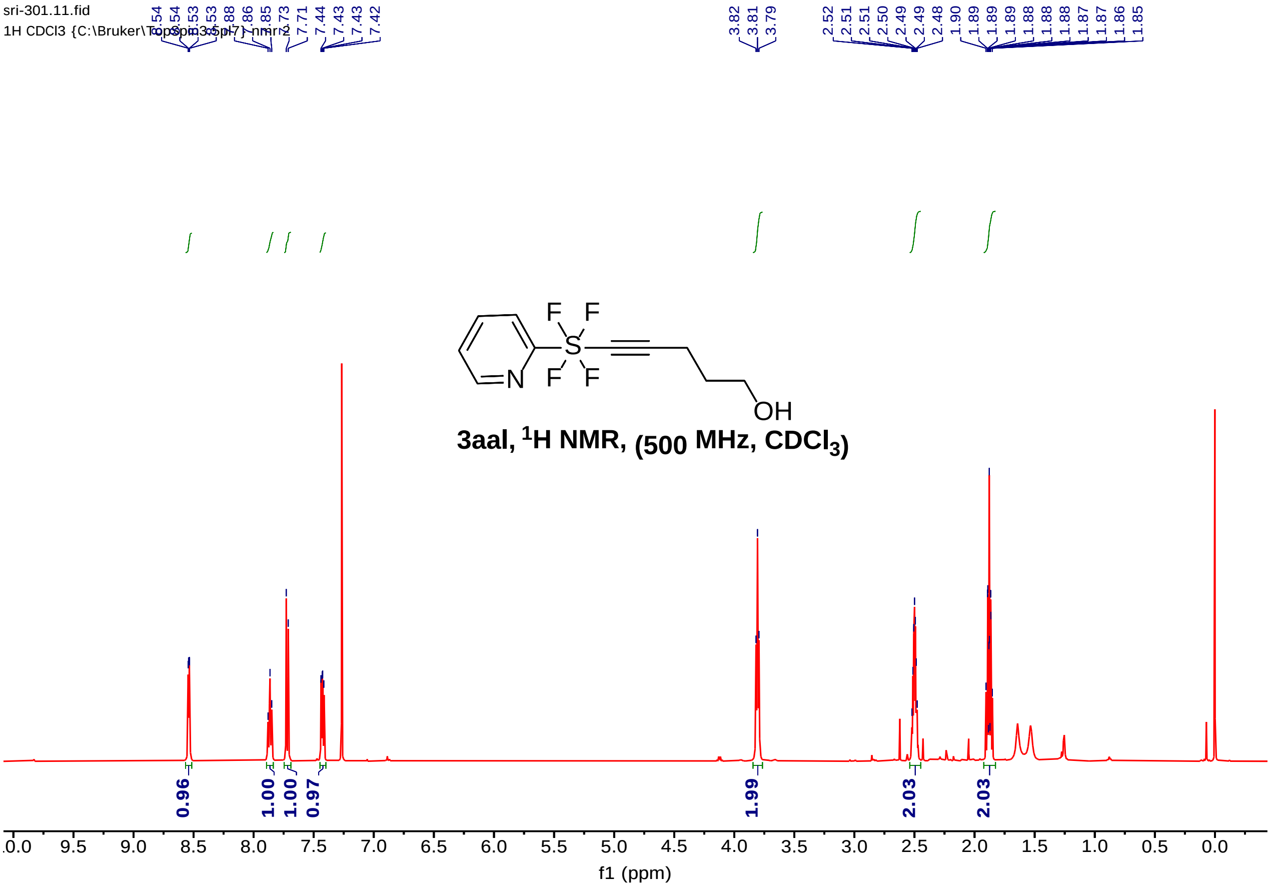
sri-301.11.fid
1H CDCl3 {C:\Bruker\TopSpin3.5pl7} nmr
3aal, 1H NMR, (500 MHz, CDCl3)
f1 (ppm)

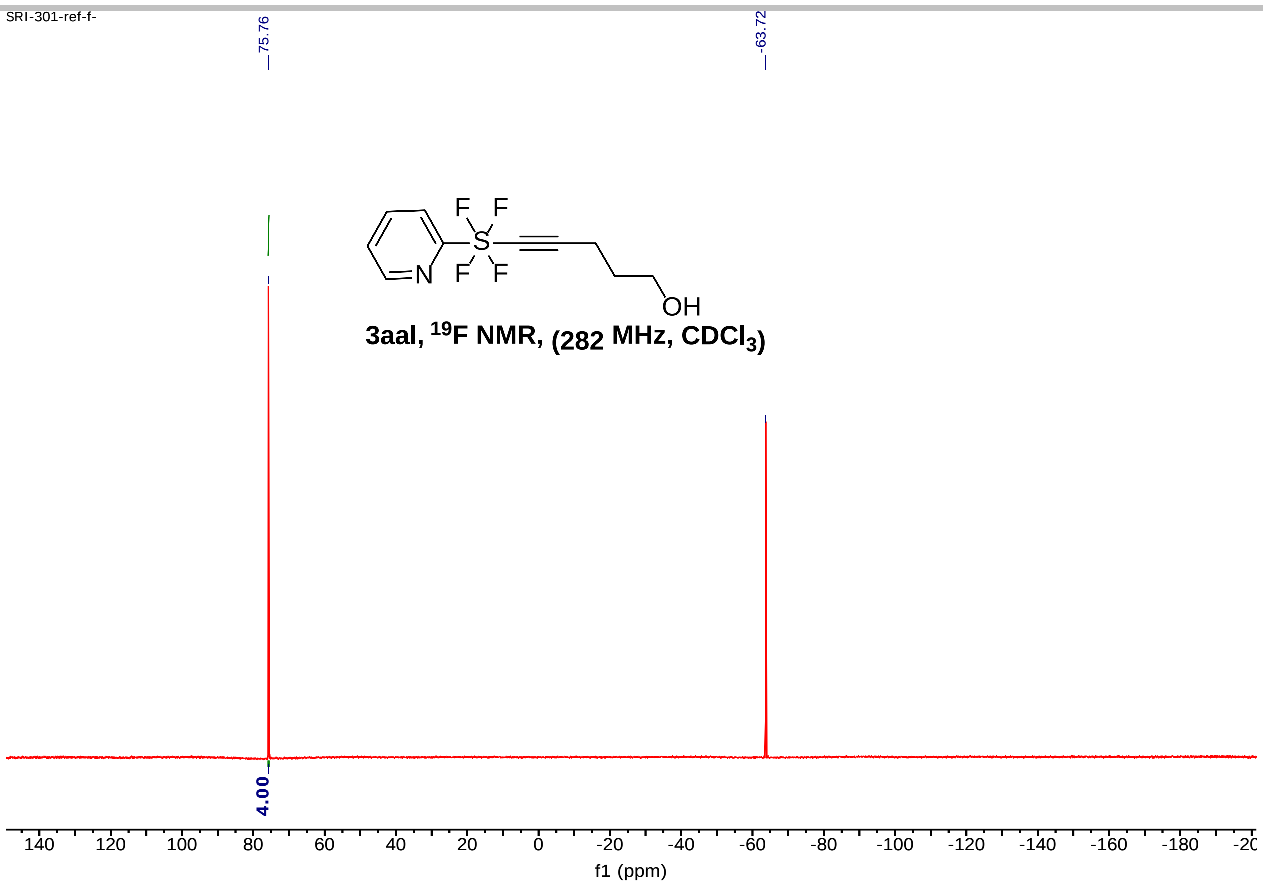
SRI-301-ref-f-
75.76
-63.72
F F
S
N F F
OH
3aal, 19F NMR, (282 MHz, CDCl3)
4.00
140 120 100 80 60 40 20 0 -20 -40 -60 -80 -100 -120 -140 -160 -180 -20
f1 (ppm)

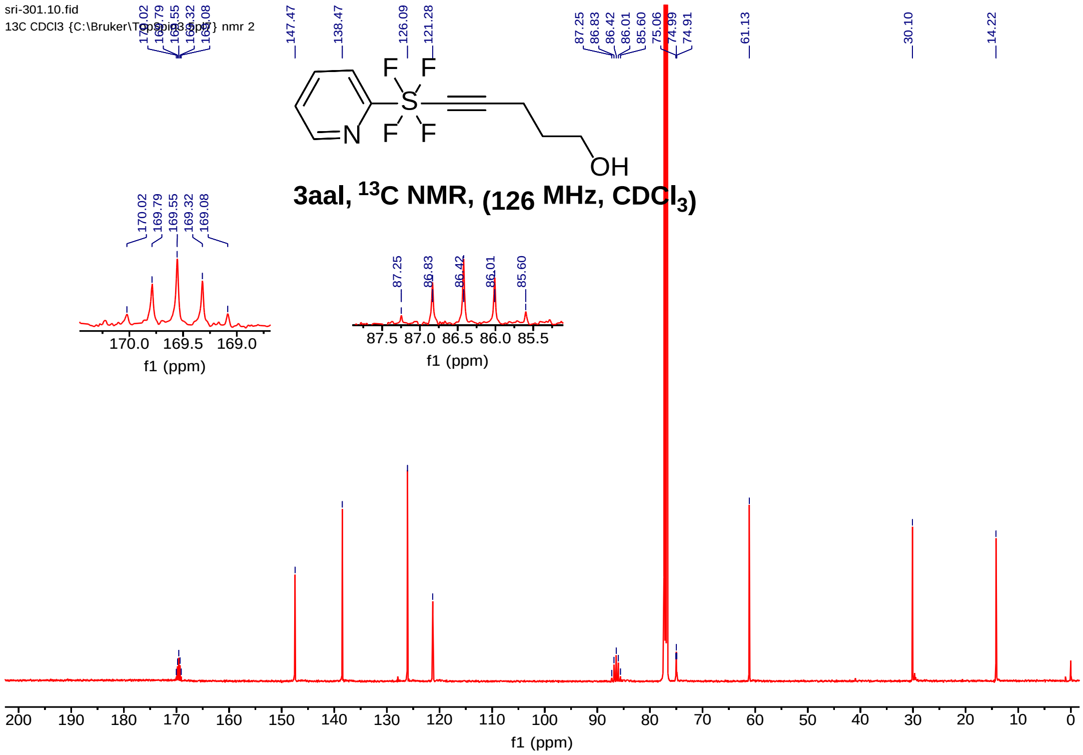
sri-301.10.fid
13C CDCl3 {C:\Bruker\TopSpin3.5pl7} nmr 2
170.02
169.79
169.55
169.32
169.08
147.47
138.47
126.09
121.28
87.25
86.83
86.42
86.01
85.60
75.06
74.99
74.91
61.13
30.10
14.22
F F
S
N F F
OH
3aal, 13C NMR, (126 MHz, CDCl3)
170.0 169.5 169.0
f1 (ppm)
87.5 87.0 86.5 86.0 85.5
f1 (ppm)
200 190 180 170 160 150 140 130 120 110 100 90 80 70 60 50 40 30 20 10 0
f1 (ppm)

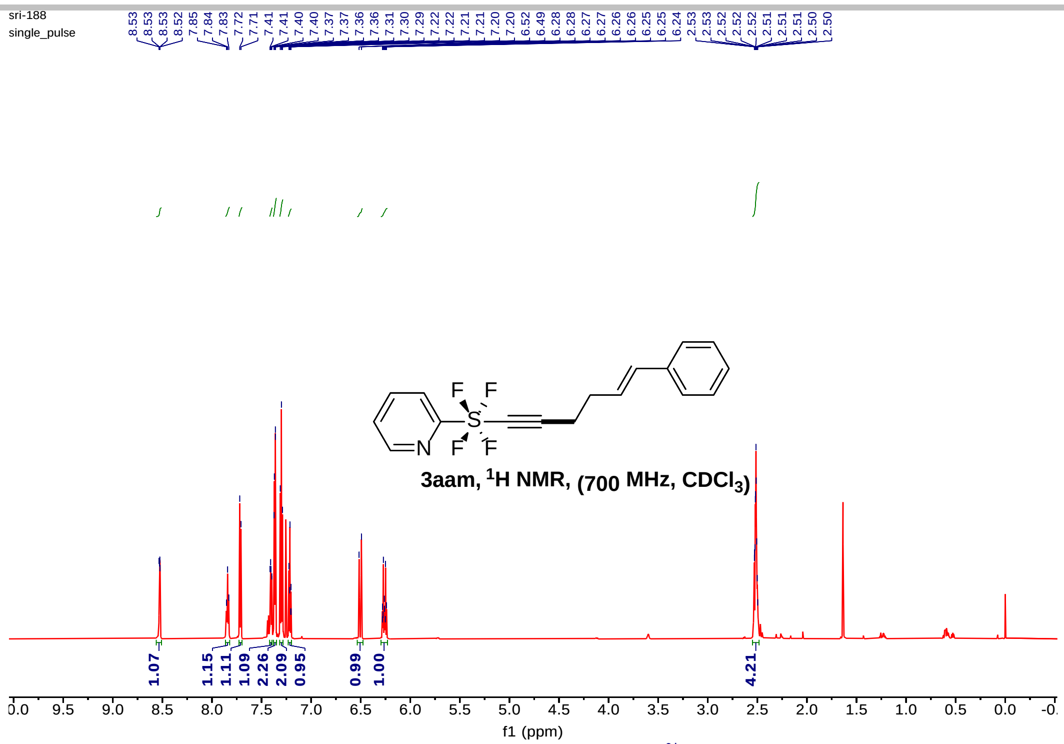
sri-188
single_pulse
8.53 8.53 8.53 8.52 7.85 7.84 7.83 7.72 7.71 7.41 7.41 7.40 7.40 7.37 7.37 7.36 7.36 7.31 7.30 7.29 7.22 7.22 7.21 7.21 7.20 7.20 6.52 6.49 6.28 6.28 6.27 6.27 6.26 6.26 6.25 6.25 6.24 2.53 2.53 2.52 2.52 2.52 2.51 2.51 2.51 2.50 2.50
F F S N F F
3aam, 1H NMR, (700 MHz, CDCl3)
1.07 1.15 1.11 1.09 2.26 2.09 0.95 0.99 1.00 4.21
f1 (ppm)

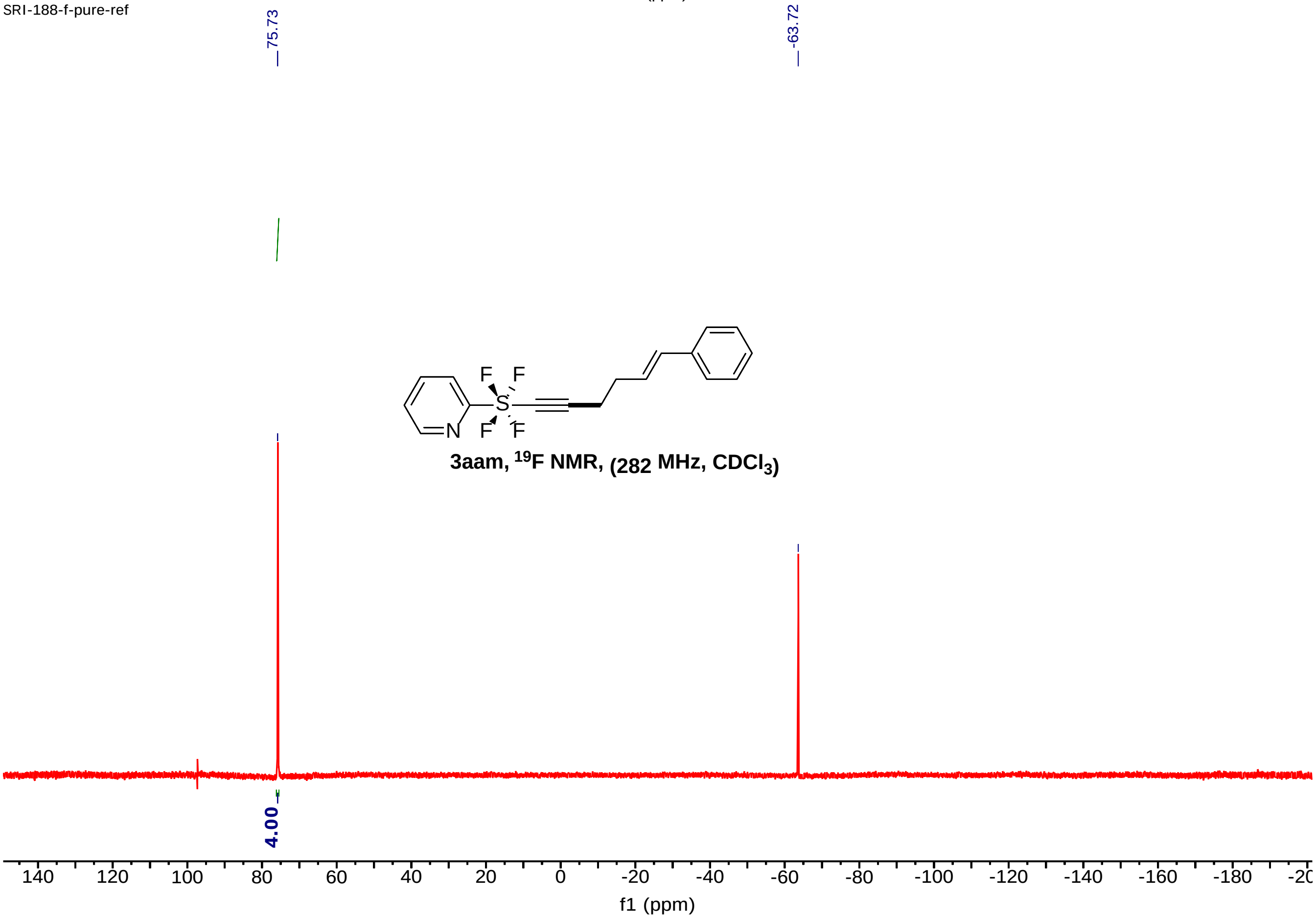
SRI-188-f-pure-ref
75.73
-63.72
F F S N F F
3aam, 19F NMR, (282 MHz, CDCl3)
4.00
f1 (ppm)

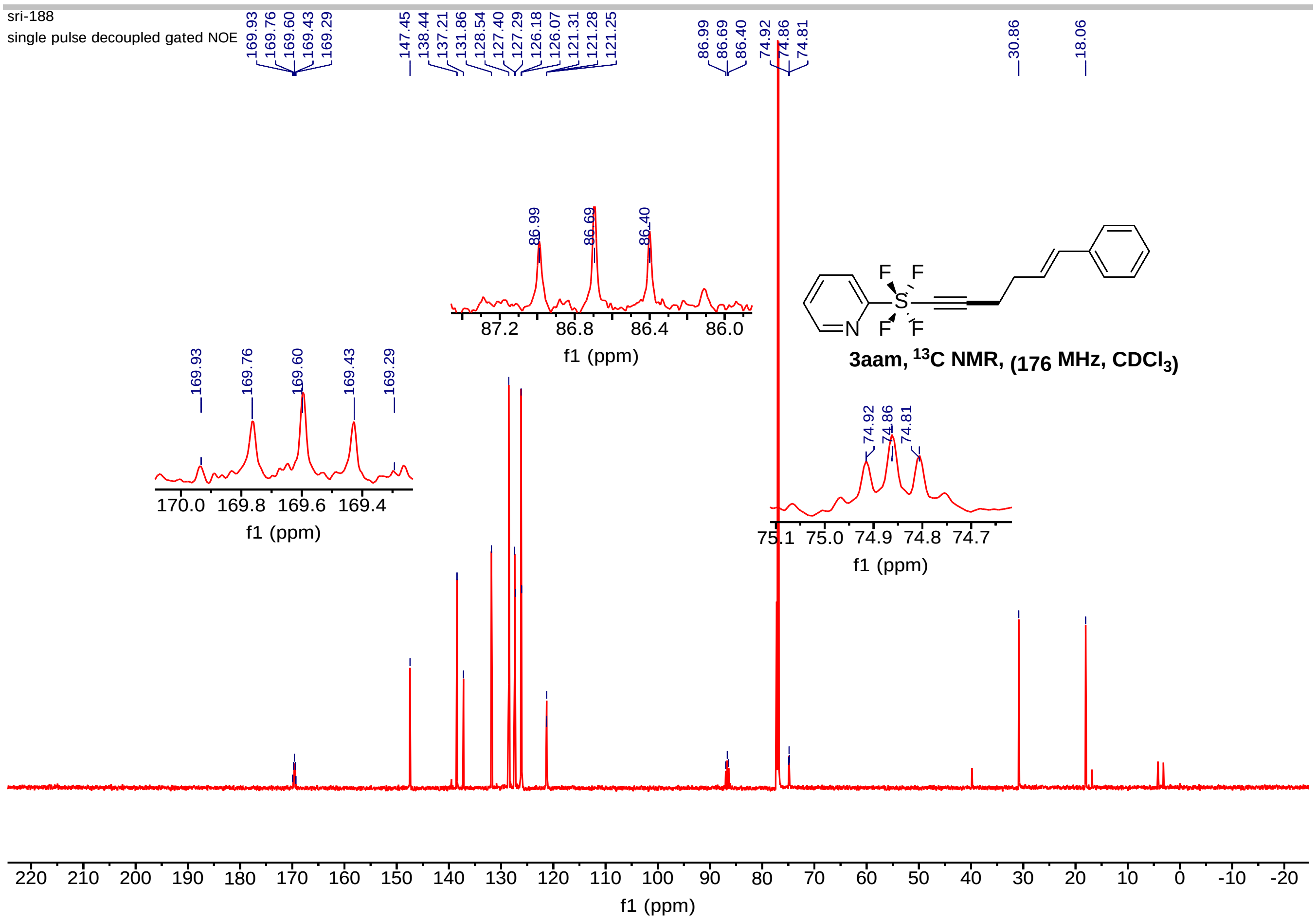

3aam, ^{13}C NMR, (176 MHz, $CDCl_3$)

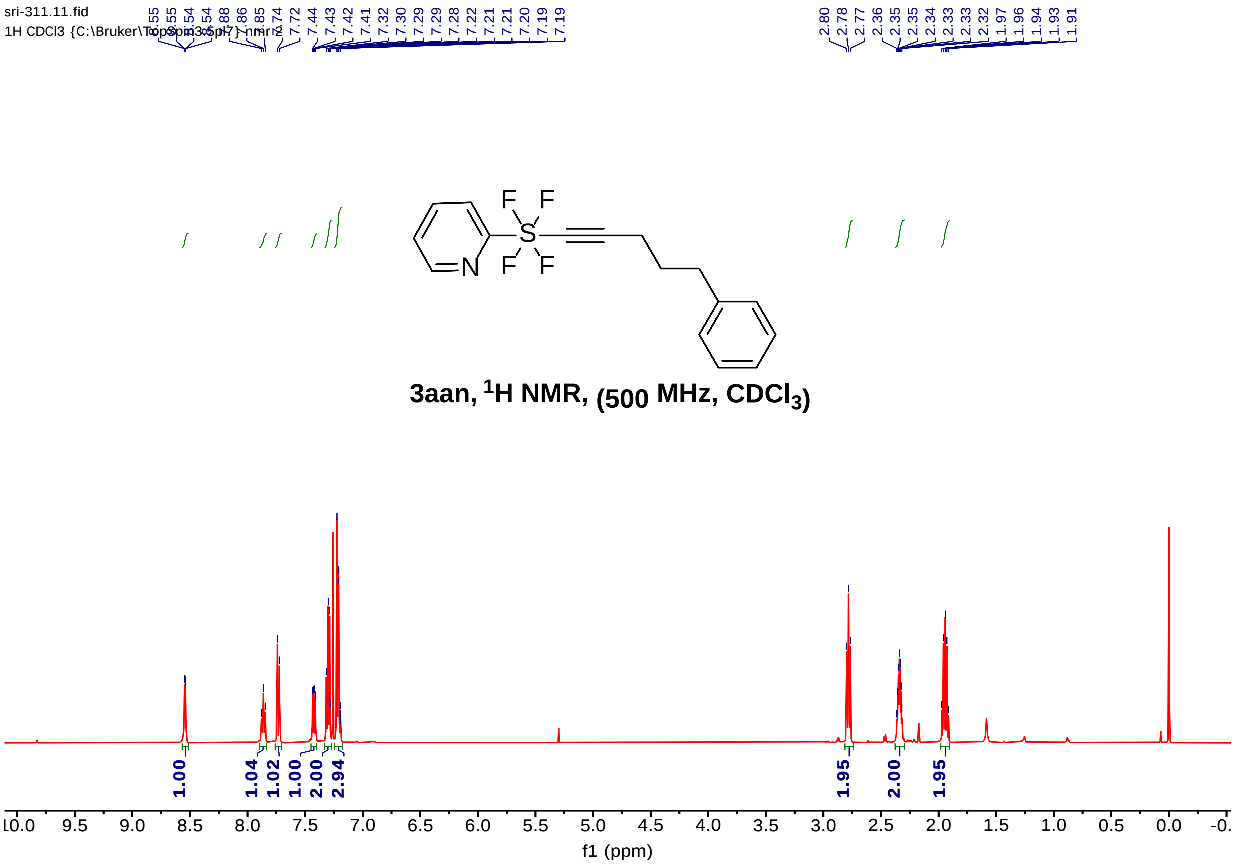

3aan, ^{1}H NMR, (500 MHz, $CDCl_3$)

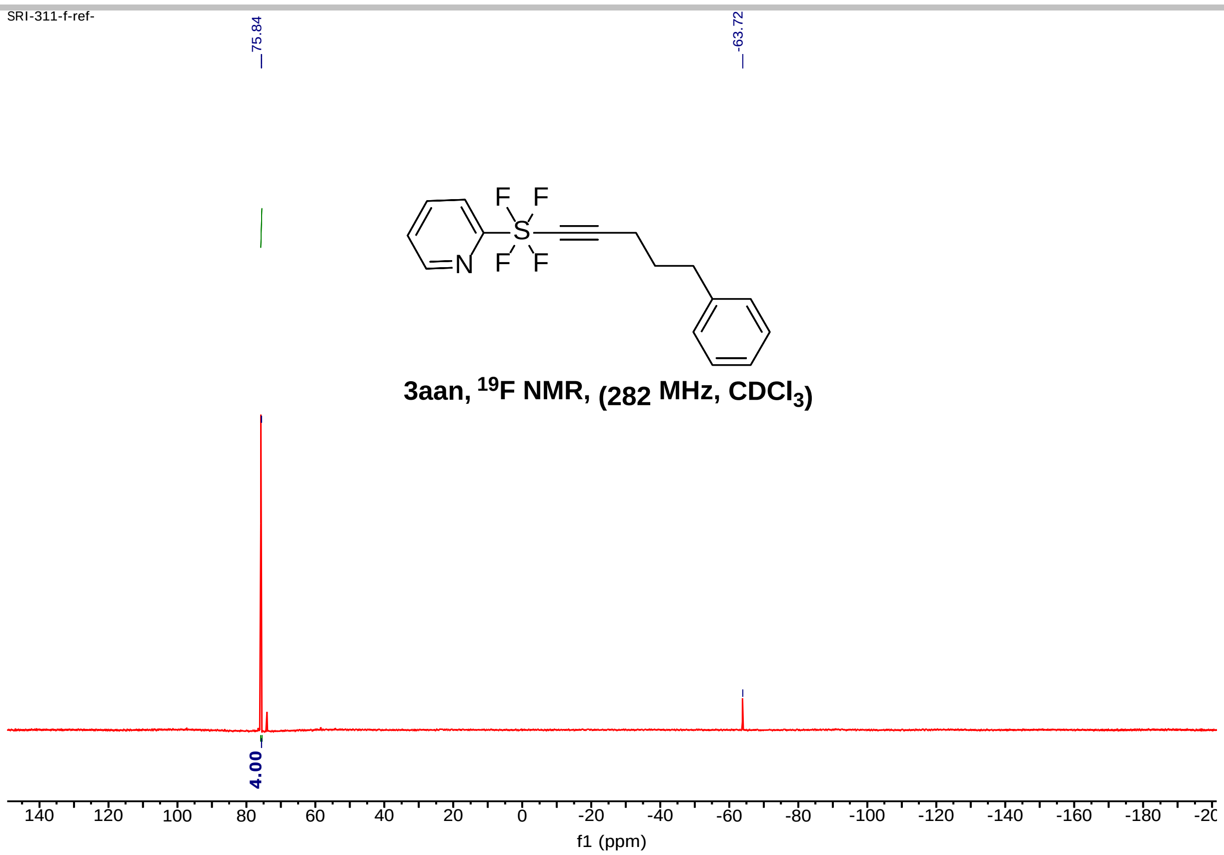
SRI-311-f-ref-
75.84
-63.72
3aan, 19F NMR, (282 MHz, CDCl3)
4.00
f1 (ppm)

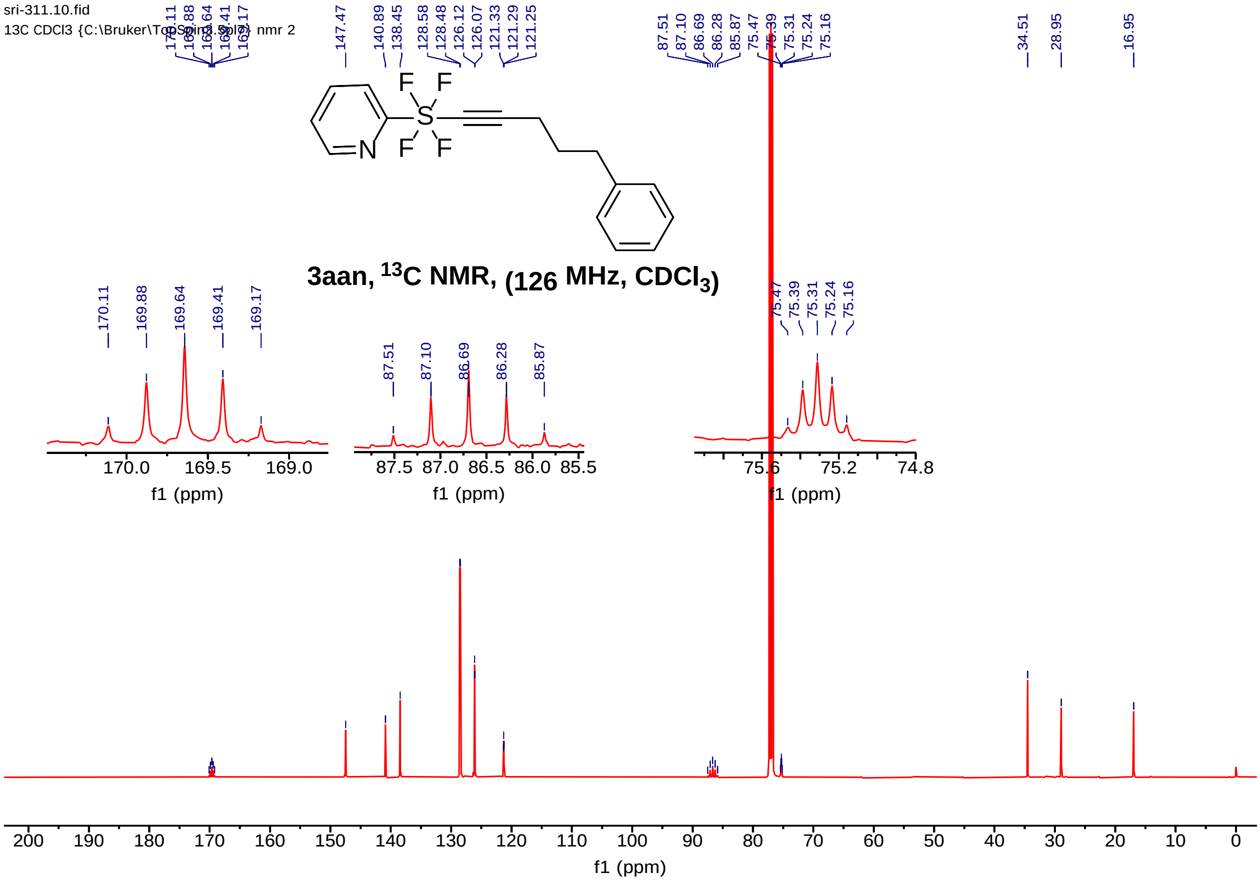
sri-311.10.fid
13C CDCl3 {C:\Bruker\TopSpin3.5pl7} nmr 2
3aan, 13C NMR, (126 MHz, CDCl3)
170.11
169.88
169.64
169.41
169.17
147.47
140.89
138.45
128.58
128.48
126.12
126.07
121.33
121.29
121.25
87.51
87.10
86.69
86.28
85.87
75.47
75.39
75.31
75.24
75.16
34.51
28.95
16.95
f1 (ppm)

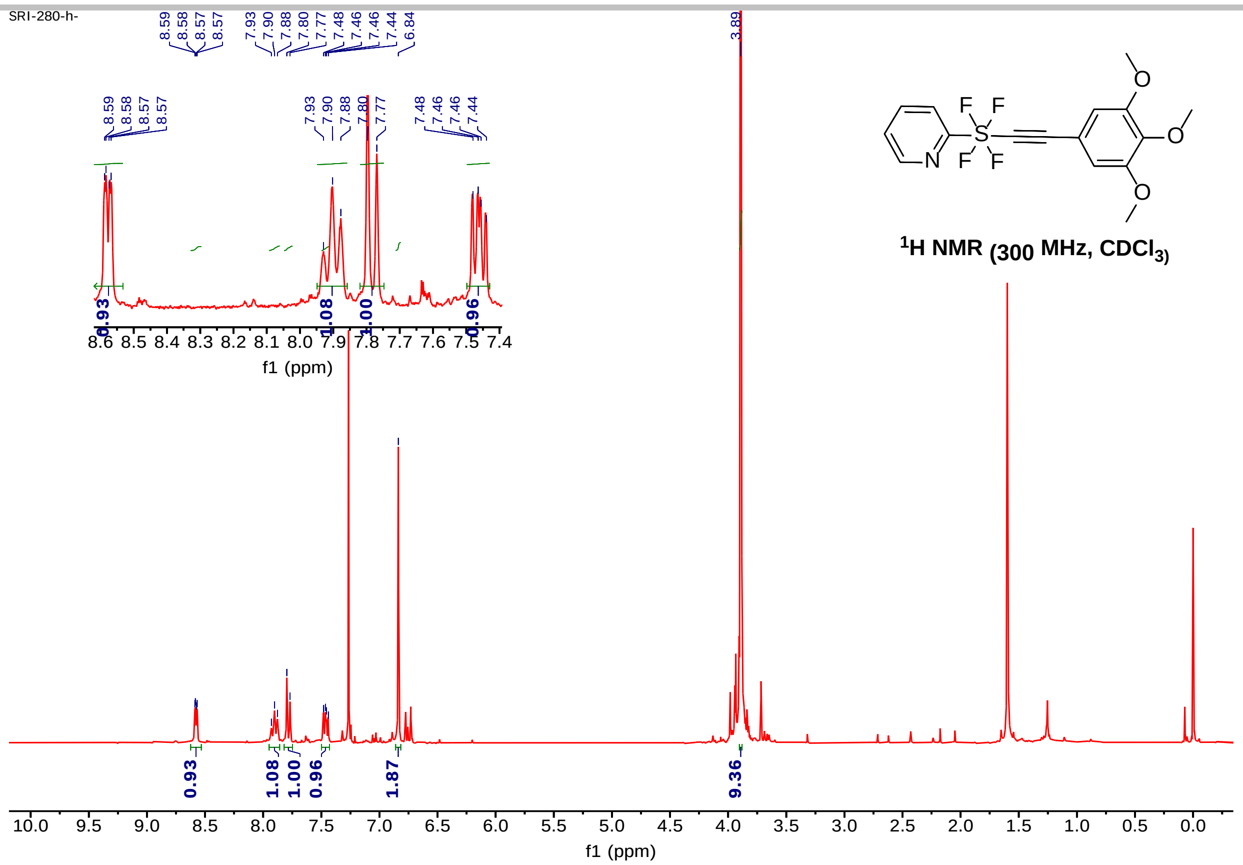
SRI-280-h-
^{1}H NMR (300 MHz, CDCl$_3$)
f1 (ppm)

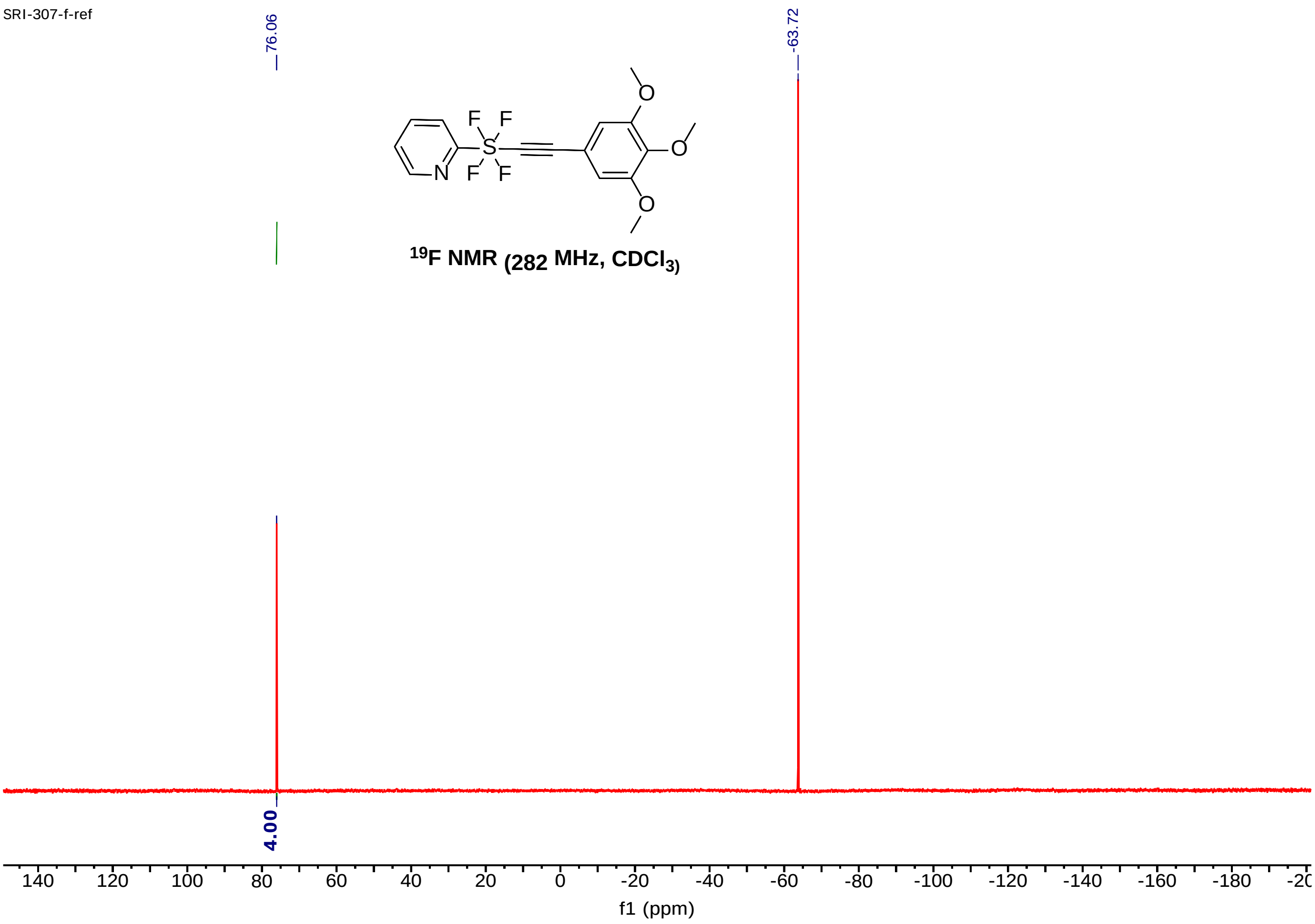
SRI-307-f-ref
^{19}F NMR (282 MHz, CDCl$_3$)
f1 (ppm)

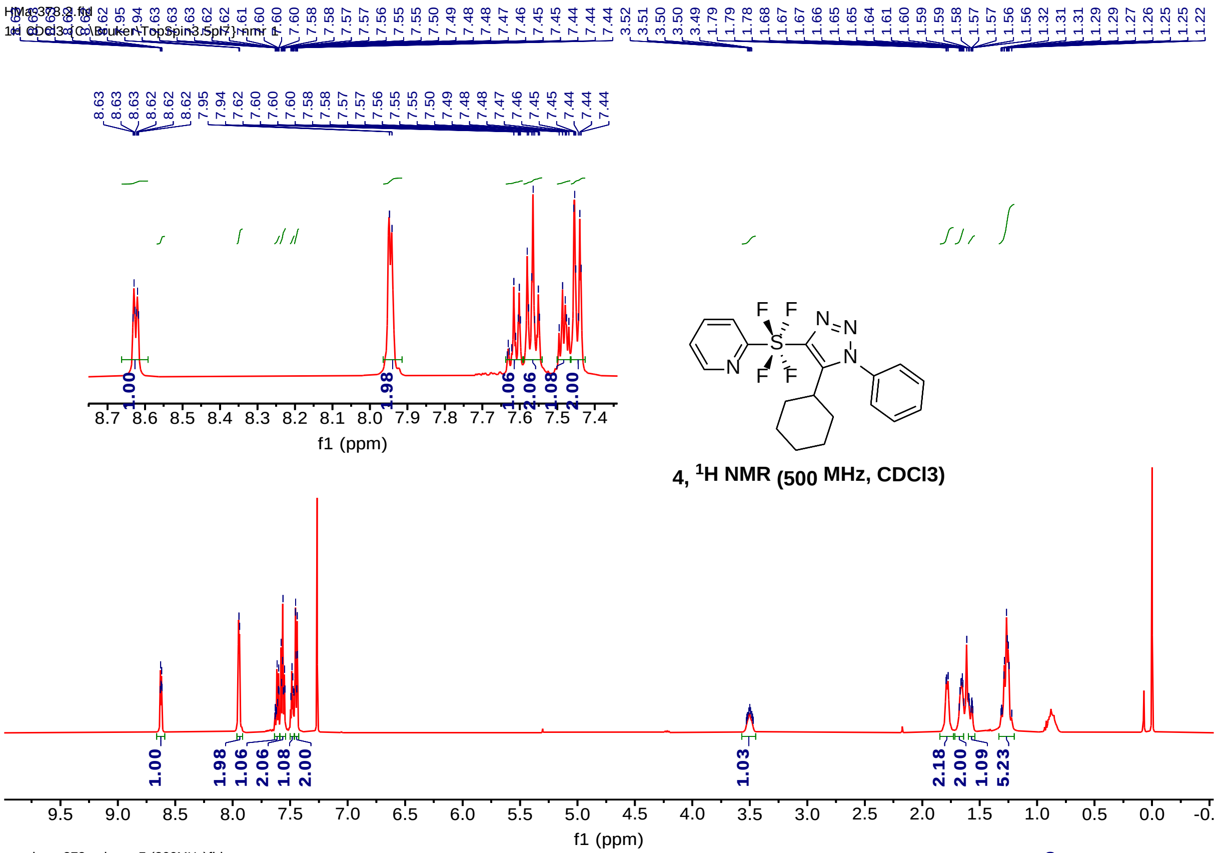

4, ^{1}H NMR (500 MHz, CDCl3)

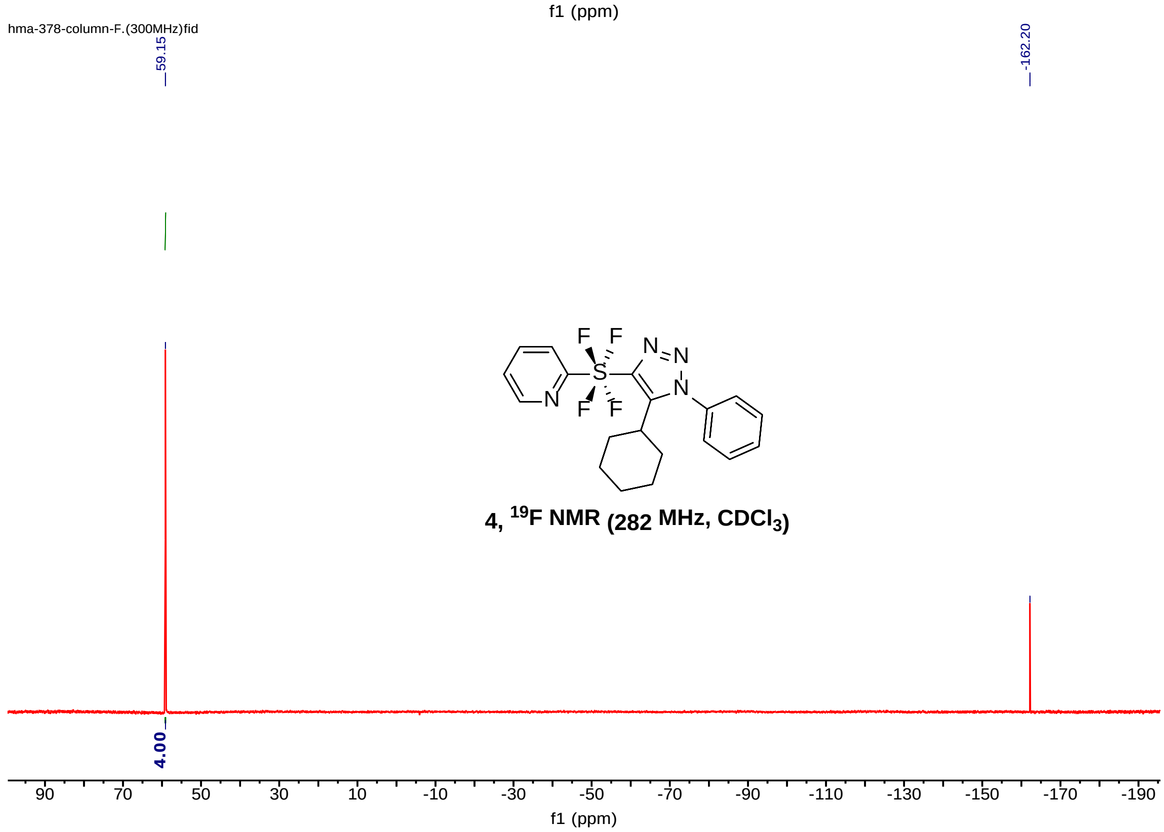

4, ^{19}F NMR (282 MHz, $CDCl_3$)

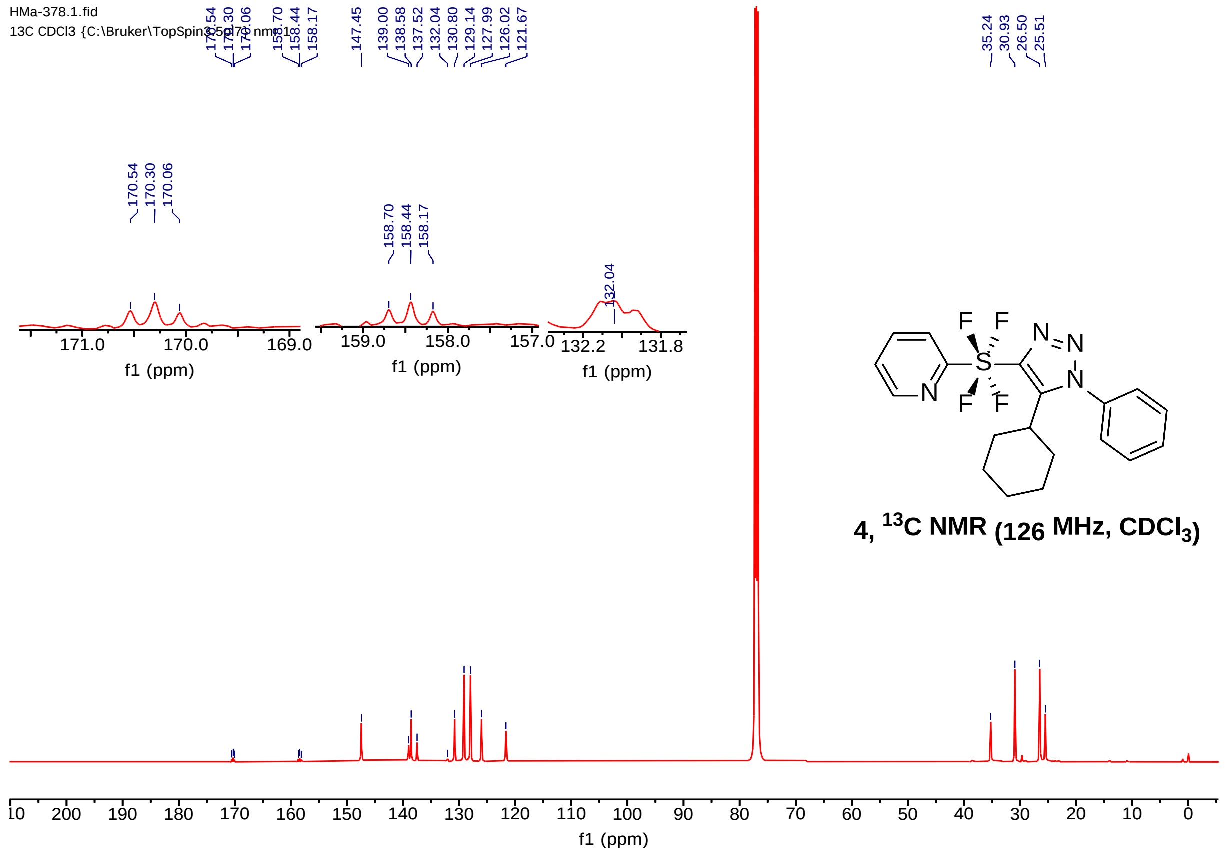

4, ^{13}C NMR (126 MHz, $CDCl_3$)

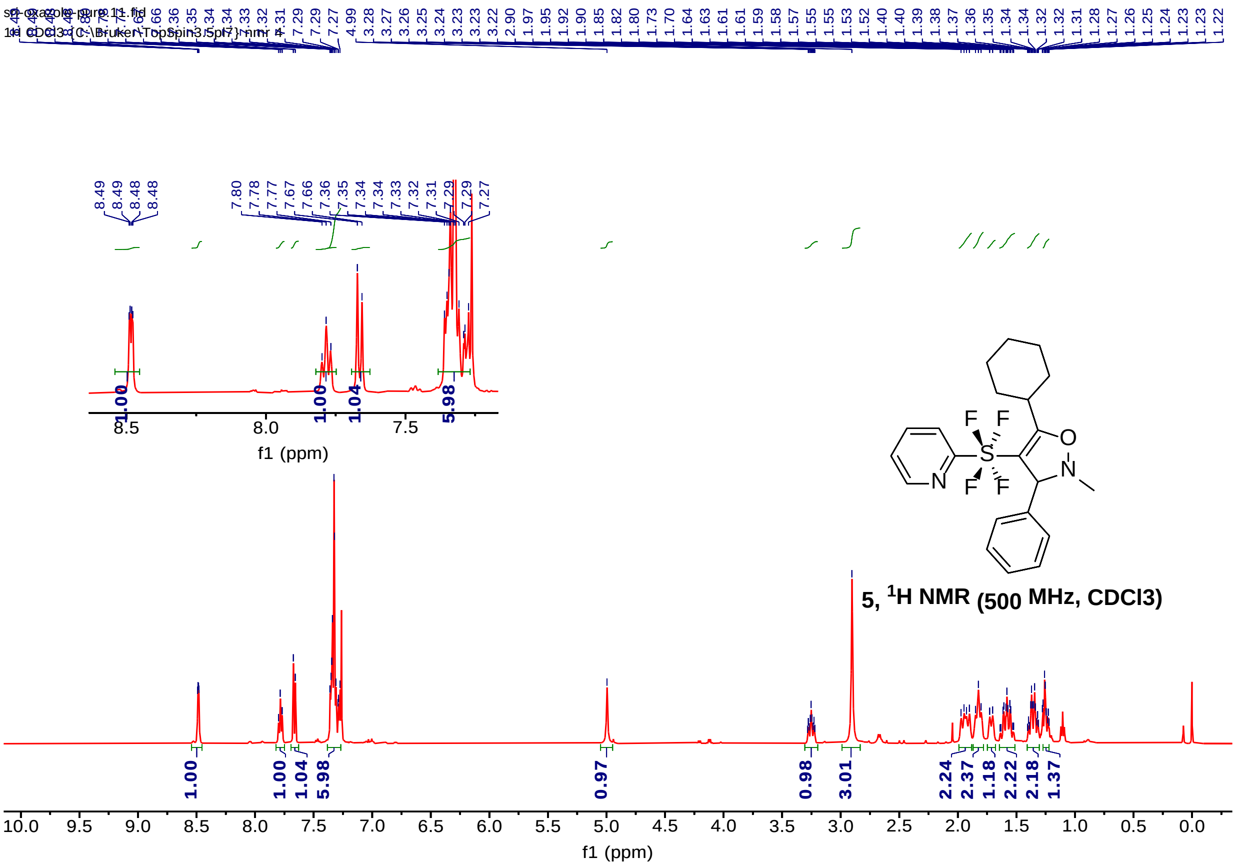

5, ^{1}H NMR (500 MHz, CDCl3)

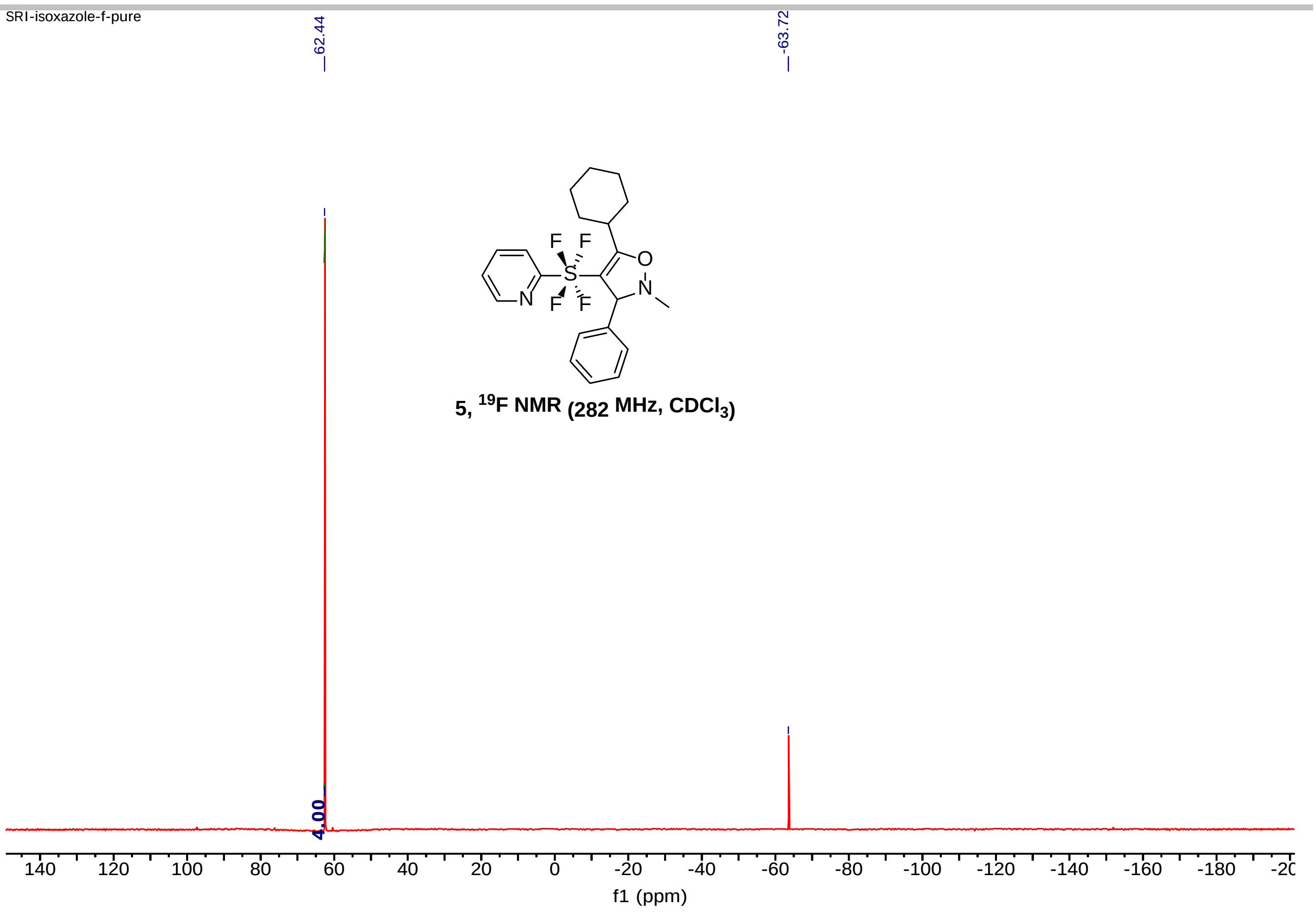

5, ^{19}F NMR (282 MHz, $CDCl_3$)

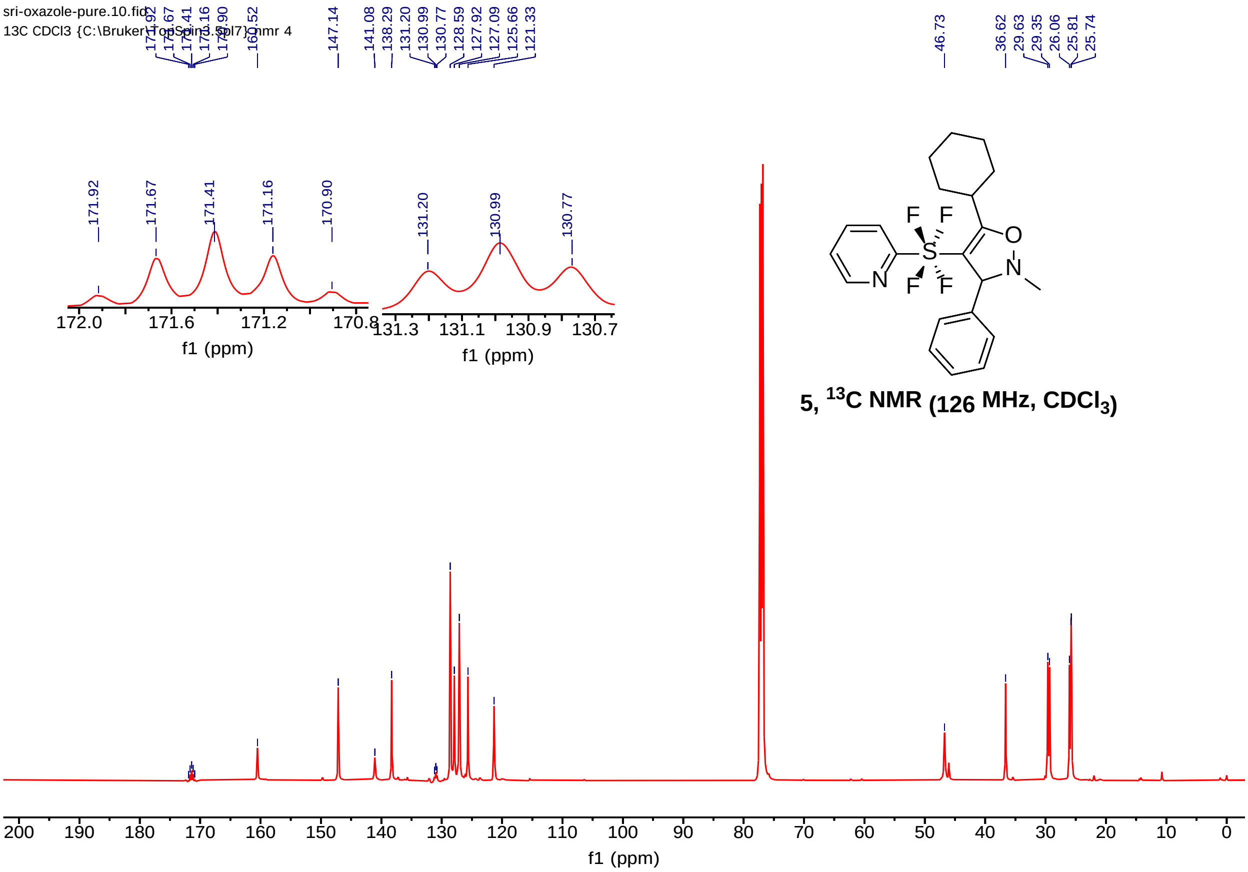

5, ^{13}C NMR (126 MHz, $CDCl_3$)
